# Quinoline Functionalized Schiff Base Silver (I) Complexes: Interactions with Biomolecules and In Vitro Cytotoxicity, Antioxidant and Antimicrobial Activities

**DOI:** 10.3390/molecules26051205

**Published:** 2021-02-24

**Authors:** Adesola A. Adeleke, Sizwe J. Zamisa, Md. Shahidul Islam, Kolawole Olofinsan, Veronica F. Salau, Chunderika Mocktar, Bernard Omondi

**Affiliations:** 1 School of Chemistry and Physics, Pietermaritzburg Campus, University of Kwazulu-Natal, Private Bag X01, Scottsville 3209, South Africa; 217080311@stu.ukzn.ac.za; 2 Department of Chemical Sciences, Olabisi Onabanjo University, P. M. B. 2002, Ago-Iwoye 120107, Nigeria; 3 School of Chemistry and Physics, Westville Campus, University of Kwazulu-Natal, Private Bag X54001, Westville 4001, South Africa; zamisas@ukzn.ac.za; 4 Discipline of Biochemistry, School of Life Sciences, Westville Campus, University of Kwazulu-Natal, Private Bag X54001, Durban 4000, South Africa; islamd@ukzn.ac.za (M.S.I.); 219017967@stu.ukzn.ac.za (K.O.); 218087036@stu.ukzn.ac.za (V.F.S.); 5 Discipline of Pharmaceutical Sciences, School of Health Sciences, Westville Campus, University of Kwazulu-Natal, Private Bag X54001, Durban 4000, South Africa; mocktarc@ukzn.ac.za

**Keywords:** Ag(I), quinolines, cytotoxicity, antimicrobial, antioxidant, CT-DNA, BSA

## Abstract

A series of fifteen silver (I) quinoline complexes **Q1**–**Q15** have been synthesized and studied for their biological activities. **Q1**–**Q15** were synthesized from the reactions of quinolinyl Schiff base derivatives **L1**–**L5** (obtained by condensing 2-quinolinecarboxaldehyde with various aniline derivatives) with AgNO_3_, AgClO_4_ and AgCF_3_SO_3_. **Q1**–**Q15** were characterized by various spectroscopic techniques and the structures of [Ag(**L1**)_2_]NO_3_ **Q1**, [Ag(**L1**)_2_]ClO_4_ **Q6**, [Ag(**L2**)_2_]ClO_4_ **Q7**, [Ag(**L2**)_2_]CF_3_SO_3_ **Q12** and [Ag(**L4**)_2_]CF_3_SO_3_ **Q14** were unequivocally determined by single crystal X-ray diffraction analysis. In vitro antimicrobial tests against Gram-positive and Gram-negative bacteria revealed the influence of structure and anion on the complexes′ moderate to excellent antibacterial activity. In vitro antioxidant activities of the complexes showed their good radical scavenging activity in ferric reducing antioxidant power (FRAP). Complexes with the fluorine substituent or the thiophene or benzothiazole moieties are more potent with IC_50_ between 0.95 and 2.22 mg/mL than the standard used, ascorbic acid (2.68 mg/mL). The compounds showed a strong binding affinity with calf thymus-DNA via an intercalation mode and protein through a static quenching mechanism. Cytotoxicity activity was examined against three carcinoma cell lines (HELA, MDA-MB231, and SHSY5Y). [Ag(**L2**)_2_]ClO_4_ **Q7** with a benzothiazole moiety and [Ag(**L4**)_2_]ClO_4_ **Q9** with a methyl substituent had excellent cytotoxicity against HELA cells.

## 1. Introduction

Schiff bases are an extensively studied important class of ligands. They are commonly synthesized by the condensation reaction of aromatic aldehydes or ketones with primary amines resulting in an azomethine moiety with at least one aryl group attached to either the carbon or nitrogen atom. The interest in Schiff base ligands is therefore as such due to their ease of synthesis, selectivity to the central metal ion [1], synthesis flexibility [2], structural diversity [3], and their broad biological applications [4].

Quinolines are derivatives of pyridine and are found in many biologically active natural [5,6] and synthetic [7,8,9] compounds. Their Schiff base derivatives have also been identified with various pharmaceutical activities [10,11]. Although some free Schiff base ligands are known to be bioactive, literature has shown that incorporating metal in such ligands enhances their activities [12,13]. This has lately driven research in metal-based drugs. Transition metal ions regarded as potential biological agents are platinum, cobalt, copper, zinc, and ruthenium [14,15,16,17]. However, there is an emerging curiosity in exploring silver(I) and its complexes as antimicrobial and antioxidant agents as well as efficient DNA and protein binders [18] with potential cytotoxicity activity. Our interest in silver(I) ion is due to its low toxicity to human at low concentration [19], high cytotoxicity [20], and good potency compared to other bioactive metal complexes such as cisplatin, known to have drawbacks which include nephrotoxicity, drug resistance, and cervical renal problems [21].

Silver(I) complexes have been reported to have significant anti-tumor activity [22,23] with minimum side effects; this is in addition to their well-known antimicrobial activities [24,25] and their effective scavenging of free radicals and reactive oxygen species (ROSs) [26].

The interaction of metal complexes with deoxyribonucleic acid (DNA) and plasma proteins are important when designing effective bioactive drugs that target the DNA since binding of small organic and inorganic molecules to DNA can influence numerous biological processes in which DNA participate, like transcription and replication [27].

The serum albumins are also considered to be one of the main molecular targets in the action of anticancer agents. Since proteins play an important role in the transportation and deposition of various endogenous and exogenous substances in the blood, their interactions with drugs lead to the formation of stable drug-protein complexes [28].

Inspired by the biological activities of pyridinyl imine reported previously by our group [29], we decided to incorporate a quinoline moiety into an imine ligand to investigate the effect of an additional phenyl ring in the ligand in biological systems. We have as such explored some ligands derived from the condensation reaction of 2-quinolinecarboxaldehyde with 2-fluoroaniline, 2-aminobenzenethiol, 4-chloroaniline, *p*-toluidine, and thiophene-2-ylmethanamine, respectively. Report herein is the synthesis, characterization, and biological studies of *N,N*-bidentate quinolinyl Schiff base ligands, and their Ag(I) -nitrate, -perchlorate, and -triflate complexes.

## 2. Results and Discussion

### 2.1. Synthesis and Characterization of **L1**–**L5**

The ligands were prepared by the condensation reaction of 2-quinolinecarboxaldehyde with five substituted aromatic anilines (2-fluoroaniline, **L1**; 2-aminobenzenethiol, **L2**; 4-chloroaniline, **L3**; *p*-toluidine, **L4**; and 2-thiophenemethylamine, **L5**) in anhydrous ethanol in excellent yields as per the method in literature [30]. All the physical and spectral data for the known ligands [31,32,33] were "in agreement" with the ones previously reported.

### 2.2. Synthesis and Characterization of the Complexes

**Scheme 1:**
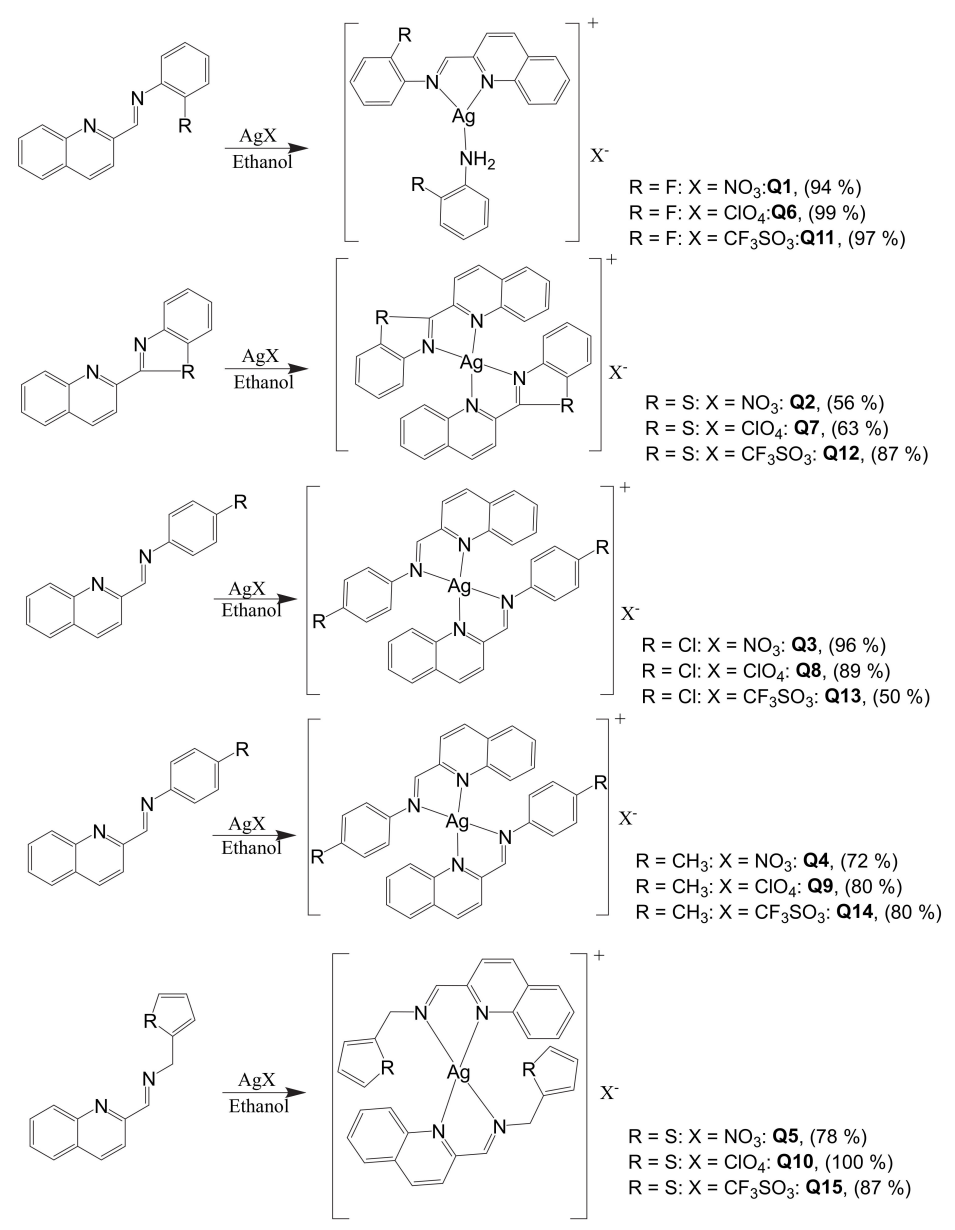
Synthesis of **Q1**–**Q15** under constant magnetic stirring in anhydrous ethanol.

Complexes **Q1**–**Q15** were obtained in good to excellent yields between 50 and 100% by reacting two equivalents of the respective ligands (**L1**–**L5**) with one equivalent of the silver(I) salts in anhydrous ethanol (Scheme 1). The air-stable solids are soluble in common organic solvents such as dichloromethane, acetonitrile, tetrahydrofuran, dimethylsulfoxide, dimethylformamide, and some in water.

The reaction of **L1**–**L5** with silver(I) metal ion in a 1:2 (Ag:L) molar ratio in anhydrous ethanol afforded fifteen complexes (**Q1**–**Q15**) and was followed using ^1^H NMR spectroscopy in DMSO-*d6*. The solution behavior of the complexes differs in the solid-state as observed in the crystal structures of **Q7**, **Q12**, and **Q14** and their ^1^H NMR spectra peaks. In the ^1^H NMR spectra of **Q1**–**Q15**, three and four coordinate geometries were observed, indicating the presence of two types of complex species in solution. In the ^1^H-NMR spectra of **Q1**–**Q15** (except **Q1**, **Q6**, and **Q11**), the integration values and the significant downfield shifts in the resonance of the protons in the vicinity of the -C=N- atoms and the alpha proton on the quinoline ring relative to their free ligand resonance peaks (Table S1) point to the formation of the complexes in 1:2 (Ag:L) stoichiometry. This suggests that **L2**, **L3**, **L4**, and **L5** are four coordinates in solution coordinating to the silver metal center via the N_im_ and N_qy_ atoms and in **L2** via N_thiazole_ and N_qy_ atoms. This mode of coordination is consistent with other reported silver(I) complexes of similar ligand [34,35,36].

The pattern of coordination is different in complexes **Q1**, **Q6**, and **Q11**. The integration values of **Q1**, **Q6**, and **Q11** showed that the complexes adopt a 3-coordinate geometry as is evident from their ^1^H-NMR spectra. A significant downfield shift in the azomethine protons and upfield shift in the alpha protons with respect to the N atom in the quinoline ring was observed in comparison with their free ligand resonance. In addition to this, protons on the carbon of aniline in **Q1**, **Q6**, and **Q11** and a singlet of amine protons at δ 5.04 ppm was seen, which shows that the silver ion coordinated to one ligand **L1** (through the N_im_ and N_qy_ atoms) and the N atom of aniline from the fraction of the second ligand resulting in a three-coordinate geometry. In the ^1^H-NMR spectrum of **Q11**, the NH_2_ proton is absent which could be attributed to the low concentration of the complex in solution. The tricoordinate observed in **Q1**, **Q6**, and **Q11** are likely due to the steric repulsions between the two Schiff bases resulting in the hydrolysis of one of the ligands. Further, the electron-withdrawing substituent in **Q1**, **Q6**, and **Q11** could have influenced this type of coordination because electron-withdrawing substituent is known for their ability to reduce electron density around the metal center and thus affects binding interaction between the metal and the coordination donor [37]. The effects of solvent on the Schiff base hydrolysis can also not be overlooked as this type of Schiff base hydrolysis during coordination has been reported [38,39].

Coordination of **L1**–**L5** to the Ag(I) centers were confirmed by comparing the FT-IR spectroscopy (Table S1). Bands observed between 1613 and 1636 cm^−1^ associated with the -C=N- bond stretching frequencies shifted to higher frequencies between 1624 and 1684 cm^−1^ in the spectra of the **L4** and **L5** upon coordination to Ag(I) while in **L1** and **L3**, the -C=N- band shifted to lower frequencies from 1618–1693 cm^−1^ to 1613–1624 cm^−1^, respectively, on coordination to Ag(I).

In the spectrum of **L2** with thiazole moiety, the -C=N- bond shifted from 1452 cm^−1^ to higher frequency on coordination to Ag(I) in the spectra of **Q2**, **Q7**, and **Q12** between ca. 1456 and 1457 cm^−1^. The absorption bands associated with the C=N quinolinyl ring in the range 1591–1593 cm^−1^ in the spectra of **L1, L2**, **L4**, and **L5** shifted to the lower frequency on coordination to 1585–1592 cm^−1^. In **L3** spectra, the C=N quinolinyl ring at 1588 cm^−1^ blue-shifted to 1591–1592 cm^−1^ on coordination in the spectra of **Q3**, **Q8**, and **Q13**. Coordination of **L2**, **L3**, **L4**, and **L5** to Ag(I) can thus be proposed to be via either N_im_ or N_thiazole_ and N_qui_ donor atoms [34,40,41], while for **L1**, coordination was via N_im_, N_qui_, and N_amine_ donor atoms.

The mass spectra of **Q1**–**Q15** were all obtained in the positive ion mode. Molecular ion peaks *m*/*z* = 468 for **Q1**, **Q6**, and **Q11** arise from [Ag(**L1**)]^+^, **Q2**, **Q7**, and **Q12** had their base peak at *m*/*z* 633 corresponding to [Ag(**L2**)_2_]^+^ while for **Q3**, **Q8**, and **Q13** and **Q4**, **Q9**, and **Q14** base peaks corresponding to [Ag(**L3**)_2_]^+^ and [Ag(**L4**)_2_]^+^ were observed at *m*/*z* 641 and *m*/*z* 601, respectively. The molecular formula [Ag(**L5**)_2_]^+^ corresponded to a prominent molecular ion peak at *m*/*z* 613 for complexes **Q5**, **Q10**, and **Q15**. All spectroscopic studies, along with the elemental analysis (see Supplementary Materials Tables S1 and S2), confirmed the formation and purity of the proposed structures in Scheme 1.

Molar conductivity of **Q1**–**Q15** was measured in dimethylformamide at 25 °C and the values range between 0.28 and 4.05 × 10^4^ S m^2^ mol^−1^ (see Supplementary Materials Table S2), indicating that the complexes act as electrolytes.

**Figure 1:**
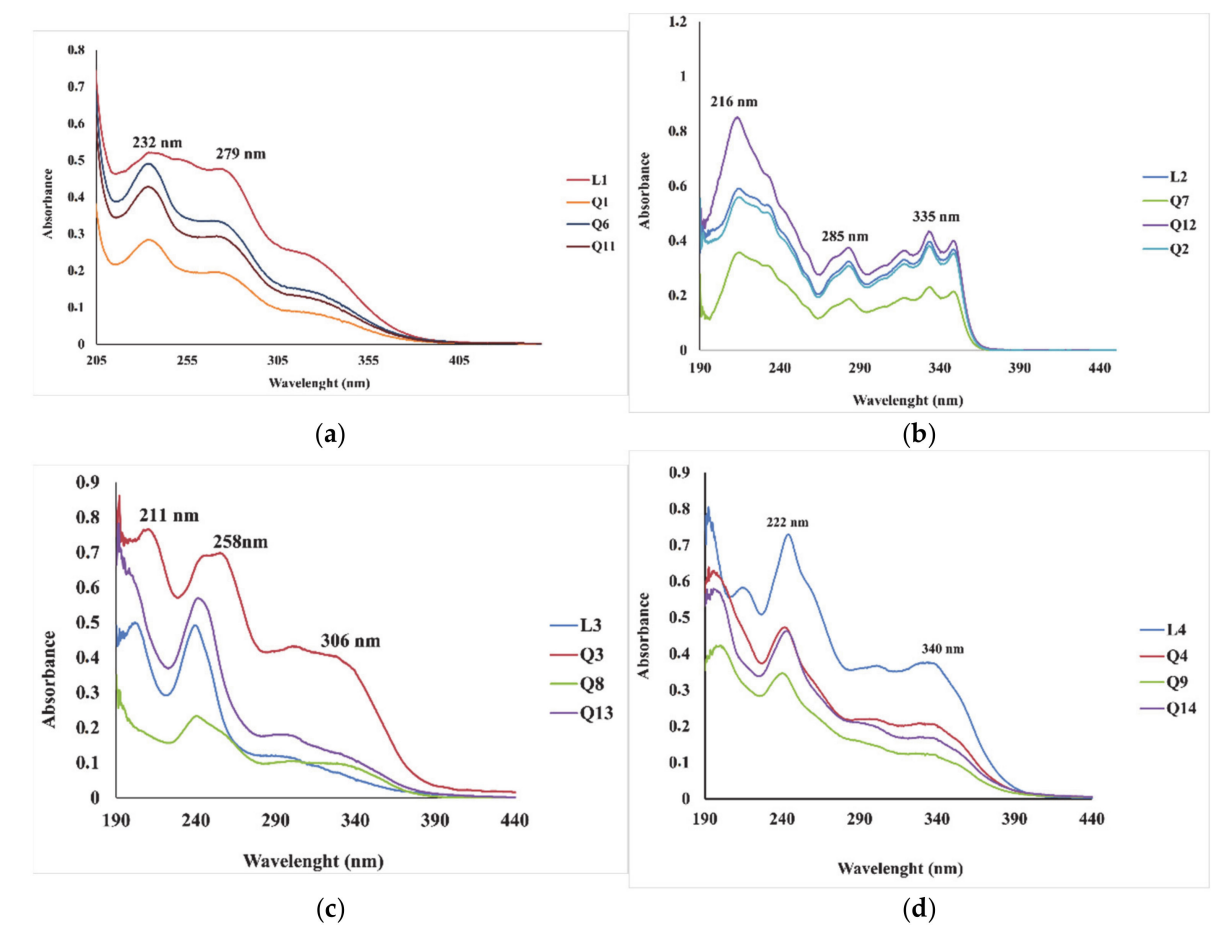

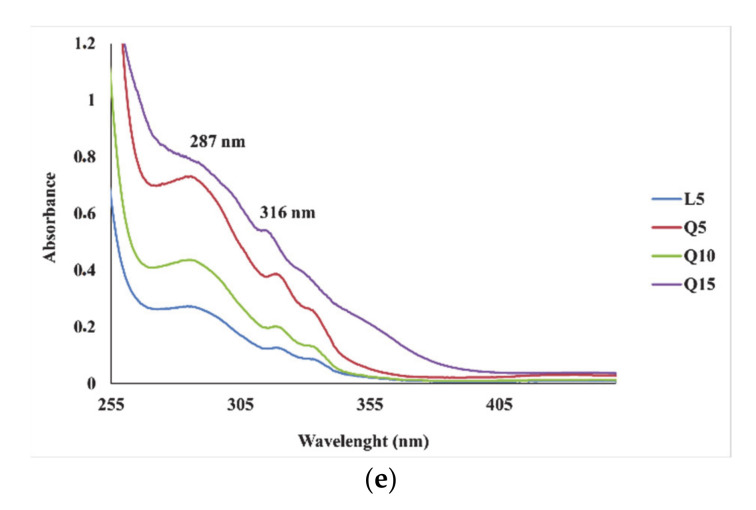
(**a**): Electronic absorption spectra of **L1** and Complexes **Q1**, **Q6** and **Q11**, (**b**) Electronic absorption spectra of **L2** and Complexes **Q2**, **Q7** and **Q12**, (**c**) Electronic absorption spectra of L3 and Complexes **Q3**, **Q8** and **Q13**, (**d**) Electronic absorption spectra of **L4** and Complexes **Q4**, **Q9** and **Q14**, (**e**) Electronic absorption spectra of **L5** and Complexes **Q5**, **Q10** and **Q15**.

Electronic absorption spectra recorded in acetonitrile at room temperature in the UV-Vis region of the free **L1**–**L5** ligands and their corresponding complexes **Q1**–**Q15** are presented in Figure 1. The absorption spectra of **L1**, **L3**, and **L4** (Figure 1a,c,d) and their respective complexes are similar having two major absorption bands in the UV region between 232–275 nm and 300–340 nm. In **L2** and its complexes absorption spectra (Figure 1b), five similar absorption bands in the UV region between 216–289 and 318–350 nm attributed to intraligand (IL) π–π * and *n*–π * transitions respectively are displayed. **L5** and its complexes showed one major absorption spectra between 287 and 291, accompanied by two shoulders between 316 and 337 nm. All the complexes′ bands can be attributed to intra-ligand *n*–π *, π–π *, and metal to ligand charge transfer transitions (MLCT) [42,43]. A general slight red-shift in the spectra of **L1**–**L5** complexes was observed, which can be associated with a decrease in energy at the excited states upon coordination to silver(I) [44,45,46]. The steric effects of the various substituents were observed to have influenced the mode of coordination in the structures of **L1**, **L2**, and **L4** complexes.

**Figure 2:**
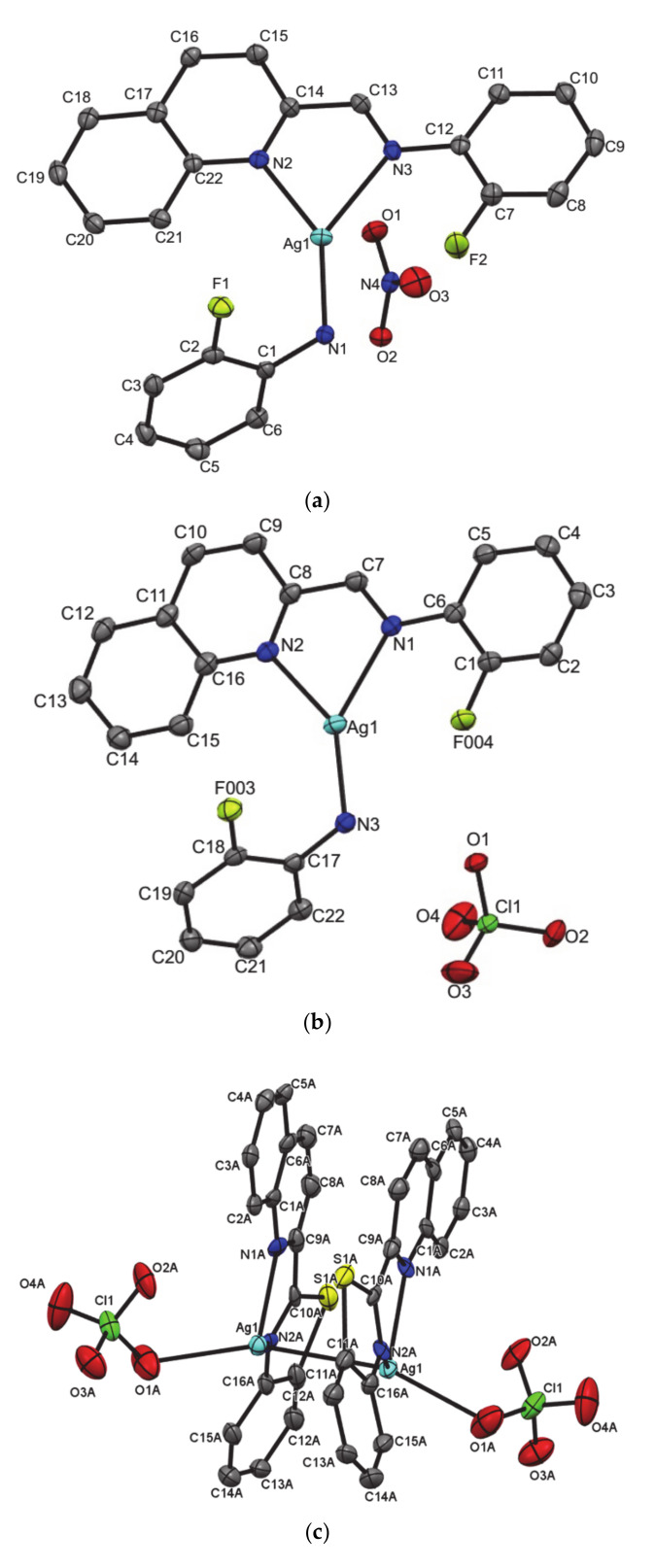

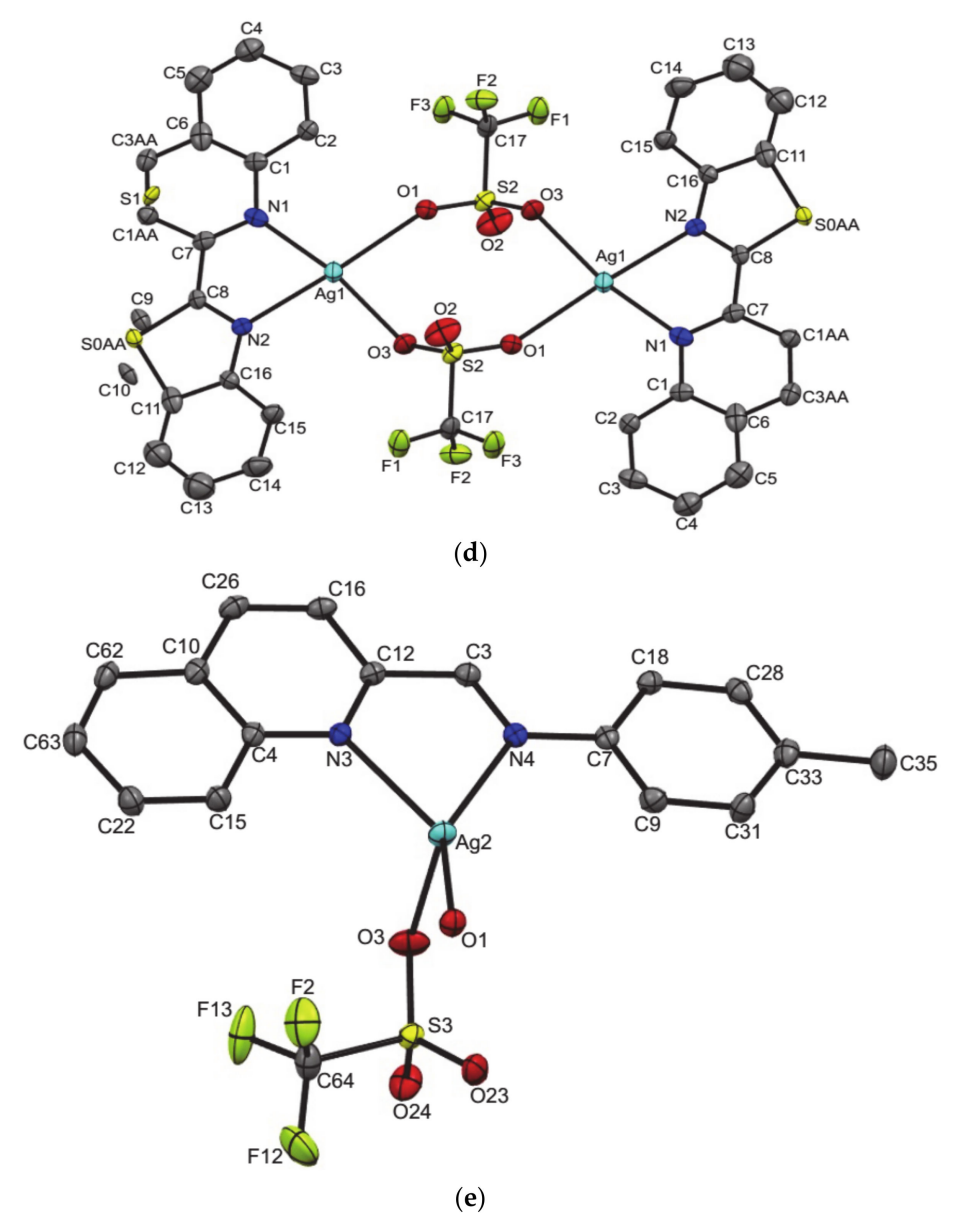
The ORTEP diagrams showing the atom numbering scheme with the thermal ellipsoids drawn at the 50% probability level for molecule of complexes (**a**) **Q1**, (**b**) **Q6**, (**c**) **Q7**, (**d**) **Q12**, and (**e**) **Q14**. Hydrogen atoms have been omitted for clarity.

In the structures of **L2** complexes, the involvement of anion in the coordination to the Ag(I) center seems to be influenced by the less steric hindrance of the benzothiazole moiety as seen in complexes **Q7** and **Q12** structures (Figure 2c,d). These types of coordination by trifluoromethanesulfonate anion have been reported [47]. In **L1** complexes, the hydrolytic cleavage of one molecule of the Schiff base ligands could be related to the steric repulsions between the two Schiff bases.

### 2.3. Crystal Structures of Complexes **Q1**, **Q6**, **Q7**, **Q12**, and **Q14**

The crystals of **Q1**, **Q6**, **Q7**, **Q12**, and **Q14** were each grown by slow evaporation of toluene diffused into dichloromethane solution of the complexes. The structures of **Q1** and **Q6** confirmed their solution behavior while that of **Q7**, **Q12**, and **Q14** are different in solution. The asymmetric units of **Q1** and **Q6** (Figure 2a,b) had a full molecule of the ligand coordinated to the metal center in a bidentate manner, an amine (from the hydrolytic cleavage of the second ligand molecule) and a molecule of the nitrate (**Q1**) or perchlorate (**Q6**), kicked out of the coordination sphere. The coordination to the metal center was via a N atom of the quinoline moiety, an imine N atom and a N atom of amine forming a three-coordinate geometry. The silver(I) centers in **Q1** and **Q6** fits in Davis et al. 2015 [48] "Extended Y" geometry description (120 ± 2° < α & β < 180 ± 2°, γ < 120 ± 2°; Σ∡′s ≥ 354°) with the sum of their angles almost 360° (**Q1**, Σ∡′s = 359.96°; **Q6**, Σ∡′s = 359.31°). The bond angles around the Ag(I) center lie between 72.96(6) and 146.71 (6)° (Table 1). The asymmetric unit of **Q7** consists of a disordered ligand, one silver(I) center with a perchlorate anion with coordination via the quinoline N atom, the imine N atom in a bidentate manner, and to an O atom of the perchlorate anion making half of the complex. The other unsymmetrically related half is generated through an Ag.Ag interaction and in the process the Ag(I) centers adopt a pseudo tetrahedral geometry. The bond angles around the Ag(I) center lie between 72.8 (4) and 105.49 (2)°. This type of coordination has been observed in related silver(I) complexes [46,49]. **Q12** has a molecule of the disordered ligand, an Ag(I) metal center and a molecule of the trifluoromethanesulfonate anion in the asymmetric unit. The other half is generated through a center of inversion lying in the center of an eight-member metallacycle in which two trifluoromethanesulfonate anions through two O atoms each bridge two metal centers. While the two sets of O atoms occupy two coordination sites of each Ag(I) center, the other two sites are occupied by two N atoms of the ligand in a bidentate manner. The resulting geometry is distorted pseudo tetrahedral with the bond angles around the Ag(I) center being 99.86 (10), 105.57 (10), 162.01 (10), and 175.69 (9)°. A similar mode of coordination has been reported [50]. The asymmetric unit of **Q14** has a molecule of the ligand, an Ag(I) metal center, a trifluoromethanesulfonate molecule and a molecule of water. Coordination in this complex is via a N atom of the quinoline moiety, an imine N atom, an O atom of a trifluoromethanesulfonate, and an O atom of a water molecule forming a pseudo tetrahedral with the bond angles around the Ag(I) center lying between 93.18 (6) and 145.17 (6)°.

All the complexes feature two sets of planes described by a five-member metallacycle including the quinoline imine unit (Plane 1) and by substituted phenyl ring for **Q1**, **Q6**, and **Q14** and by the thiazole moiety in the structures of complexes **Q7** and **Q12**.

The Ag–N_im_ bond distances in **Q1**, **Q6**, **Q7**, and **Q12** range between 2.317 (18) and 2.465 (3) Å and are generally longer than the Ag–N_qy_ bond distance between the range 2.240 (16) and 2.346 (3) Å but shorter in **Q14** (Table 1). The Ag–O bond distances range from 2.233 (16) and 2.609 Å. The Ag–N and Ag–O bond distances in complexes **Q1**, **Q6**, **Q7**, **Q12**, and **Q14** are within expected ranges and are comparable with those reported in the literature [50,51]. Complexes **Q7** has Ag Ag interaction where the bond distance is 3.0563 (11) Å.

### 2.4. In Vitro Antimicrobial Studies

All the ligands and complexes were tested against two Gram-positive bacteria, Methicillin-resistant *Staphylococcus aureus* (MRSA) and *Staphylococcus aureus* (*S. aureus)*, and four Gram-negative bacteria, *Escherichia coli* (*E. coli)*, *Salmonella typhimurium* (*S. typhi)*, *Klebsiella pneumoniae* (*K. pneumoniae),* and *Pseudomonas aeruginosa* (P. *aeruginosa)*. The minimum inhibitory concentrations (MIC) values were compared to Ciprofloxacin and the three silver(I) salts as standards (Table 2). The aim was to investigate the effect of introducing the silver metal to the ligands in the bacteria strains. **Q1**–**Q15** showed better antibacterial activity compared to **L1**–**L5** in general an indication of enhanced activity of the ligands on complexation to Ag(I). The general belief is that complexation of the ligands leads to high lipophilicity which in turn can lead to the rupturing of the cell membranes of the microorganisms [52]. In addition, silver(I) ion is well known for its bactericidal effects, mechanistically acting by obstruction of bacteria cell functions through direct interaction with the bacteria enzymes, DNA and the negatively charged peptidoglycan from the bacterial cell wall. This interrupts cell division and replication and cell breathing leading to cell death through the release of their fluid and electrolyte content into the environment [53].

Only **L1** showed some activity against *S. aureus* (100 µg/mL) while **L3** also had some activity against MRSA (1000 µg/mL). Against the Gram-negative bacteria, the ligands showed selective activity. L1 is active against *E. coli* with an MIC value of 1000 µg/mL, **L2** and **L3** against *P. aeruginosa* and *K. pneumoniae* with MIC values between 50 and 100 µg/mL, **L4** against *S. typhi* and *E. coli* and **L5** against *P. aeruginosa*, *S. typhi,* and *K. pneumoniae* with MIC values of 100 µg/mL, 50 µg/mL, and 100 µg/mL, respectively. **Q7** and **Q8** showed the highest antimicrobial activity against all bacteria strains. The ligands of each of **Q7** and **Q8** possess either the benzothiazole moiety or the *p*-chloro substituent. Enhanced antimicrobial activity of compounds containing either benzothiazole moiety or the chlorine substituent in the para-position is in accordance with the literature report [55,56] making them targets for drug design. In addition, both **Q7** and **Q8** have the perchlorate anion. Enhance antimicrobial activity was also observed in other complexes with perchlorate anion against most of the bacteria strains.

All the complexes with the exception of **Q4**, **Q6**, and **Q10**, were selectively active against *K. pneumoniae* (in comparison to the other strains) with MIC values between 0.0125 and 0.4 µg/mL, lower than that of Ciprofloxacin (0.8 µg/mL). The activity of **Q7** against *S. aureus* with an MIC value of 1.6 µg/mL can be singled out as it was better than that of the standard ciprofloxacin, AgNO_3_, AgClO_4_, or AgCF_3_SO_3_. Similarly, **Q4**, **Q6**, **Q11**, **Q12**, and **Q15** with an MIC value below 25 µg/mL signified their better activity than ciprofloxacin against *S. aureus*. Those of **Q1**, **Q2**, **Q10**, and **Q13** have the same MIC value as the standard reference against *S. aureus*. All the complexes are fairly active against MRSA with only **Q6** and **Q8** with MIC value 25 µg/mL showing the same activities as ciprofloxacin. **Q6** and **Q8** have fluorine and chlorine respectively, as their substituent and perchlorate as their counter anion. This revealed the influence of electron-withdrawing functional groups on antimicrobial activity. This type of compound with an electron-withdrawing group has been reported to have excellent antimicrobial activity [57]. Against *P. aeruginosa*, **Q8** having chlorine as its substituent, showed excellent activity higher than the reference standards while **Q7** has the same MIC value of 0.2 µg/mL as ciprofloxacin. **Q14** and **Q15** have similar MIC value as AgClO_4_ (0.8 µg/mL), **Q5**, **Q6**, **Q9**, **Q10**, have MIC values within 1.6–6.25 µg/mL lower than AgNO_3_ and AgCF_3_SO_3_ while **Q2**, **Q3**, **Q4**, **Q12**, and **Q13** (6.25 µg/mL) have similar MIC value as AgNO_3_. **Q6**, **Q10**, **Q14**, and **Q15** having either thiophene moiety, fluorine, methyl substituent in their ligand performed better than the standard against *P. aeruginosa*, these complexes also have either perchlorate or triflate as their anion.

The MIC of all the complexes except **Q15** and AgNO_3_ (1000 µg/mL) against *S. typhi* is within 12.5–100 µg/mL, these values are higher than the ciprofloxacin standard (0.4 µg/mL), only AgClO_4_ (0.2 µg/mL) had better activity than the standard. **Q5** having thiophene moiety and nitrate ion as counter anion has the same MIC value as AgCF_3_SO_3_ (12.5 µg/mL). This shows complexes **Q1**–**Q15** fair activity against *S. typhi*. The activities of **Q5** (with thiophene) and **Q8** (with chlorine) against *E. coli* are the same as the standard (1.6 µg/mL). Complexes **Q6** and **Q9** (12.5 µg/mL) were half of their salt (25 µg/mL), while **Q10** (6.25 µg/mL) was four times lower than the salt against *E. coli*. The enhanced activity of **Q8** with chlorine substituent and **Q5** (with thiophene) are 64 and 32 times higher than the standard respectively against *K. pneumoniae*. All the complexes showed excellent antibacterial activities against *K. pneumoniae*. **Q4** has the same MIC value as the standard (0.8 µg/mL).

In summary, the antimicrobial activities of the complexes exhibited a structural activity relationship because those with the chlorine substituents, benzothiazole, and thiophene moieties and with perchlorate anion displayed a remarkable antimicrobial activity. Enhanced antimicrobial activities of compounds containing similar electron-withdrawing groups [58,59], benzothiazole [60], and thiophene [61] moieties have been reported. Reports on the effects of different counter anions are also known [62].

**Figure 3:**
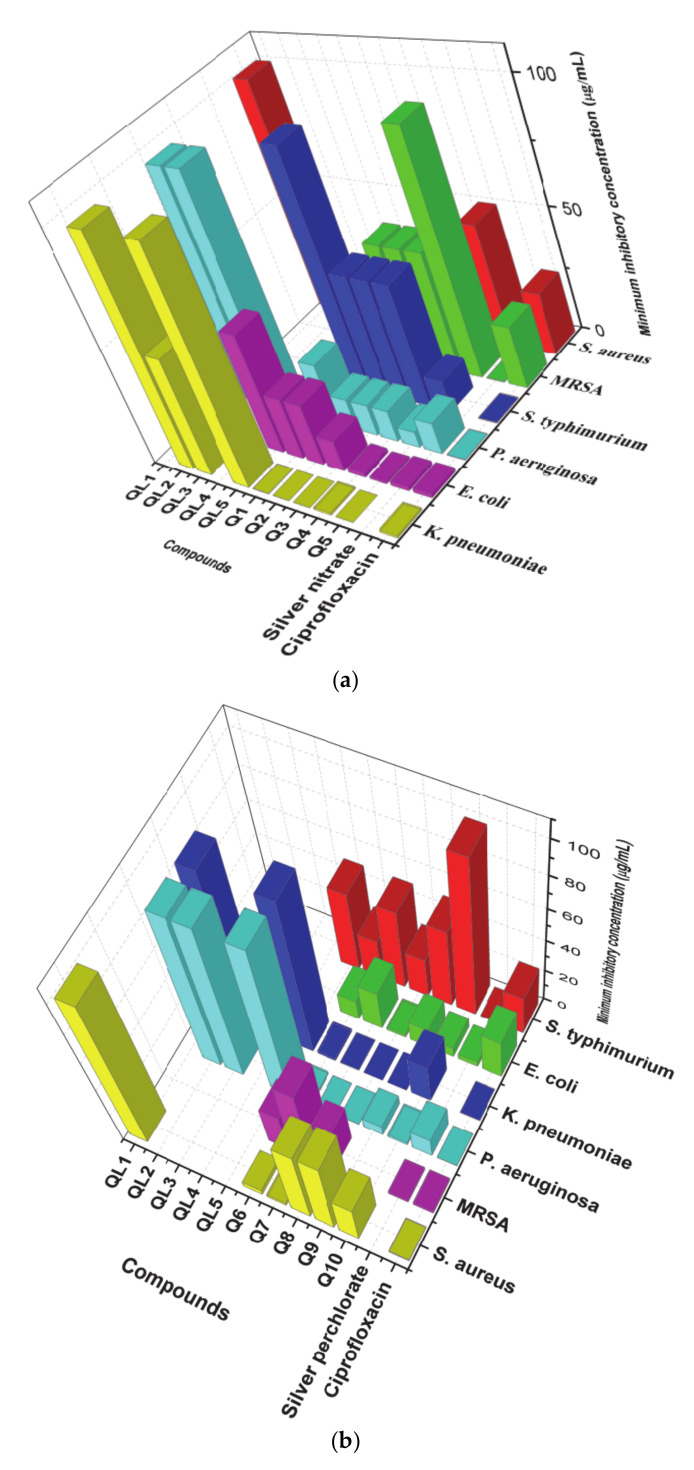

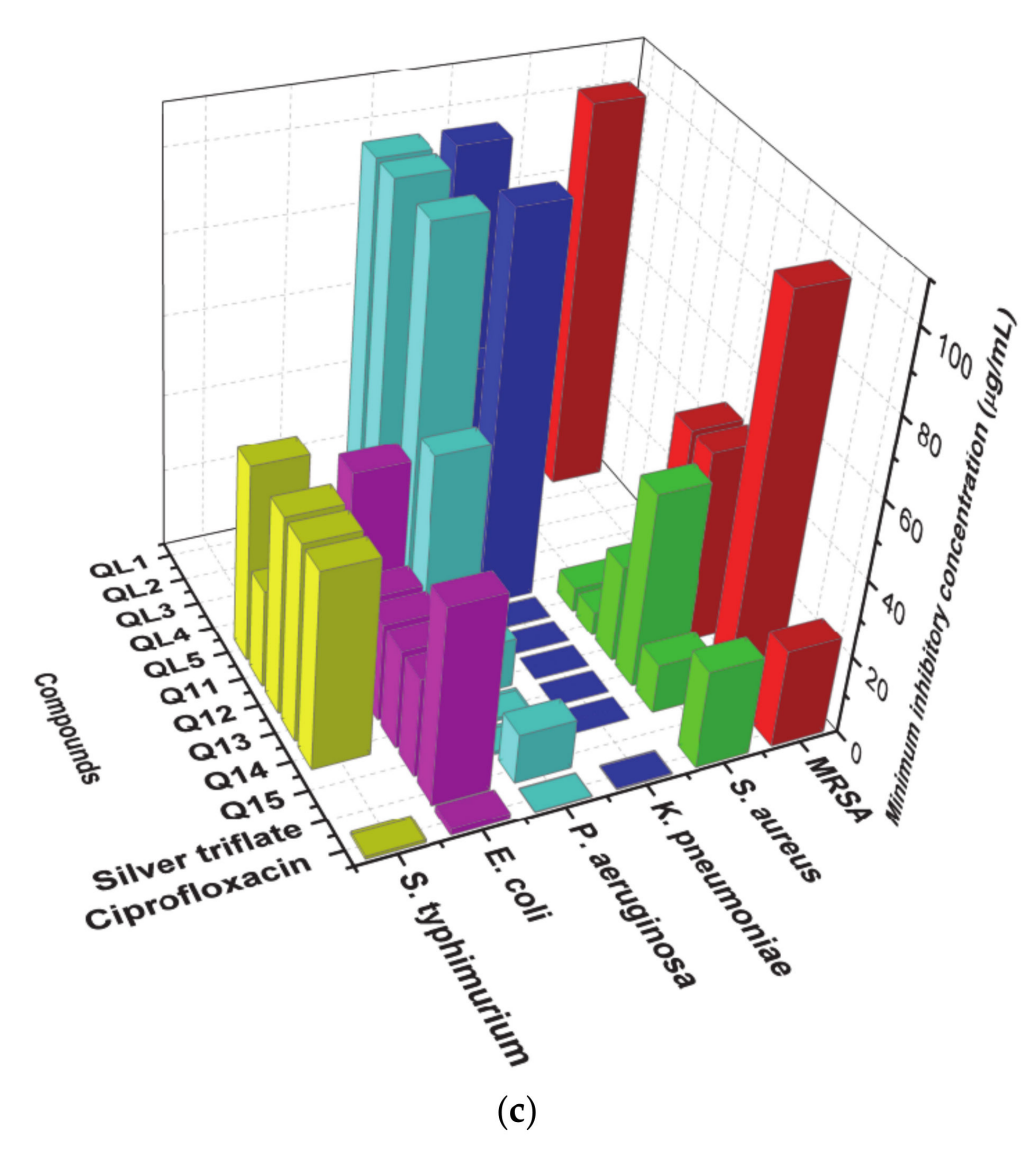
(**a**) Minimum inhibitory concentration (µg/mL) of **L1**–**L5** silver(I) complexes with nitrate as counter anion, (**b**) Minimum inhibitory concentration (µg/mL) of **L1**–**L5** silver(I) complexes with perchlorate as counter anion, and (**c**) Minimum inhibitory concentration (µg/mL) of **L1**–**L5** silver(I) complexes with triflate as counter anion.

A comparison bar chart representing each salt complex is shown in Figure 3a–c. Compounds with 1000 µg/mL MIC value and those without antimicrobial activity are omitted from the bar plot for clarity.

### 2.5. In Vitro Antioxidant Studies

Antioxidants are important in protecting the human body against damage by reactive oxygen species (ROS). Accumulation of ROS in the cells may lead to damage of the DNA, RNA, and proteins which subsequently leads to several physiological disorders such as cardiovascular disease, diabetes, rheumatism, atherosclerosis, cancer, aging and cell death [63,64]. The antioxidant activity of some compounds such as dithiocarbamate complexes [65] and pyridine derivatives complexes [66,67,68] have been reported to possess the ability to slow down and stop damage to DNA, RNA, protein substrates and thereby prevent the occurrence of diseases. The free radical scavenging ability of all ligands and complexes in this study were investigated using ferric reducing antioxidant power (FRAP). In antioxidant studies, FRAP assay is often preferred because it is simple, fast, economical, and direct in antioxidant measurement. FRAP assay is based on the electron-donating ability of an antioxidant compound. This involves the reduction of a colorless Fe^3+^ tripyridyltriazine complex (Fe^3+^ (TPTZ)) to an intense blue-colored Fe^2+^ tripyridyltriazine complex (Fe^2+^ (TPTZ)) by the electron-donating antioxidants at low pH [69]. The reducing power is to determine the reductive ability of the test compounds. Table 3 summarizes the mean values from three independent experiments of in vitro antioxidant activities of the ligands **L1**–**L5** and complexes **Q1**–**Q15** at different concentrations. The antioxidant activities of the compounds are expressed as 50% inhibitory concentration (IC_50_ in mg/mL). Ascorbic acid with an IC_50_ value of 2.68 mg/mL was used as a standard for the study.

All the compounds tested showed some antioxidant activity. Of the ligands, **L2** had the highest activity with an IC_50_ value of 2.29 mg/mL even better than that of the standard reference. The complexes and the metal salts had better activities than the ligands on the general. The antioxidant activity of the silver salts was evaluated to study the influence of the metal salts on the ligand′s antioxidant activity. Interestingly, the silver salts exhibit significant antioxidant activity comparable to the standard, but the introduction of an organic ligand to a metal ion is known to help with the regulation of metal ion absorption, distribution and metabolism in the biological systems [70,71]. Hence, we focus on the antioxidant activity of complexes **Q1**–**Q15** as an alternative synthetic antioxidant in this study.

**Figure 4:**
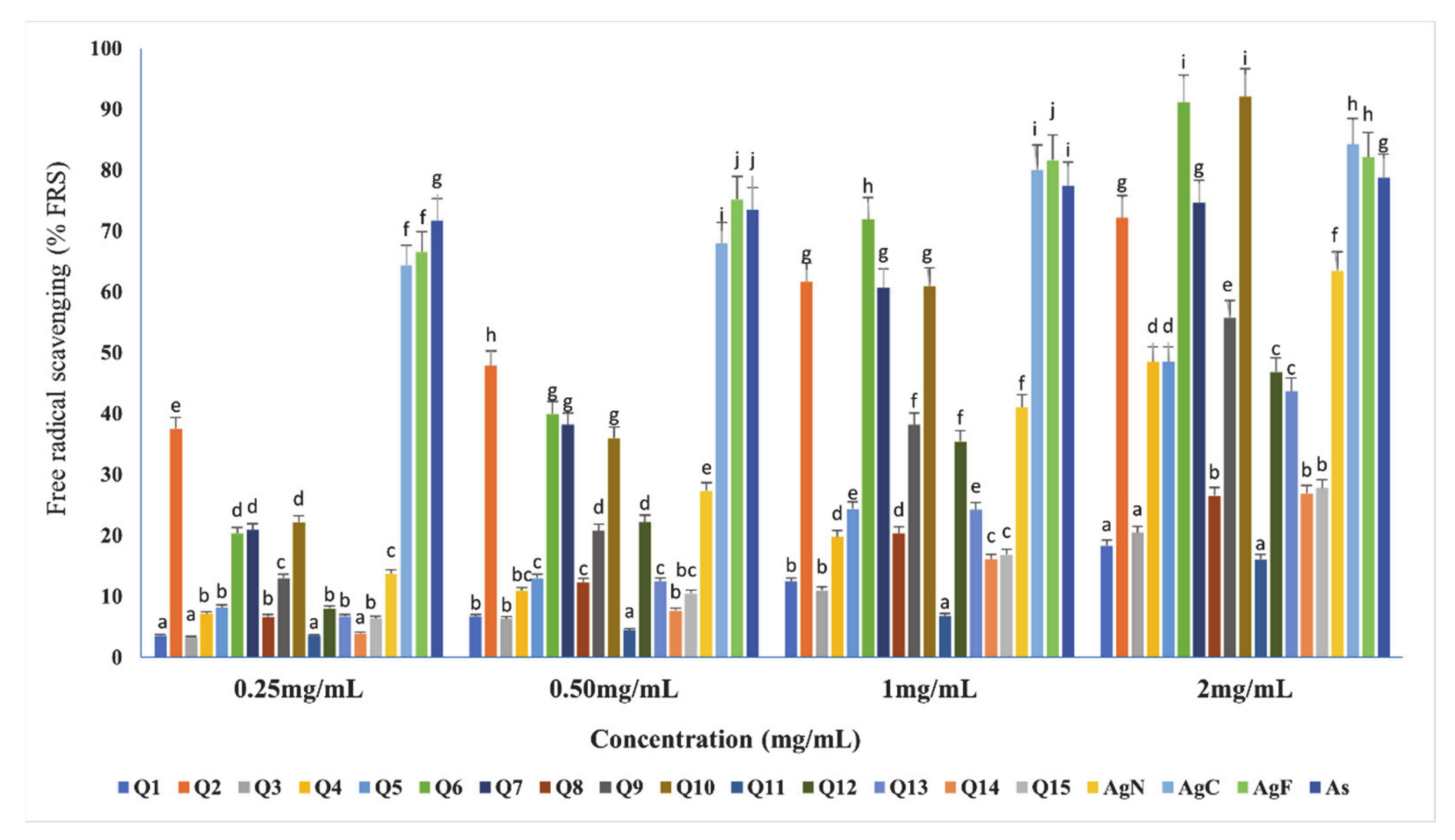
FRAP % free radical scavenging vs. Concentration (mg/mL) of complexes **Q1**–**Q15**, silver nitrate, silver perchlorate, silver triflate, and Ascorbic Acid. AgN = silver nitrate, AgC = silver perchlorate, AgF = silver triflate and As = Ascorbic acid values represent mean ± standard deviation (*n* = 3). ^a–f^ Different alphabets over the bars for a given concentration for each complex represent significant difference (Tukey′s-HSD multiple range post hoc test, *p* < 0.05).

**Q2**, **Q4**, **Q5**, **Q6**, **Q7**, **Q9**, **Q10**, and **Q13** had IC_50_ values between 0.95 and 2.22 mg/mL (Table 3) better than that of ascorbic acid (2.68 mg/mL) but not those of the free metal salts. The implication is that the complexes had a good ability to reduce Fe^3+^ to Fe^2+^ (in FRAP) was good. The common occurrence in these complexes is either a fluorine substituent, thiophene or a benzothiazole moiety. There have been reports of compounds with fluorine substituent (C_0.5 FRAP_ = 424.5–502.6 μM) with potent antioxidant activities in FRAP [72]. Similarly, other reports have shown significant antioxidant activities of benzothiazole (553.5 mmol Fe^2+^/mmol Compound) [73] and thiophene (4.55 mmol TE/g compound) [74] derivatives. In this study, **Q6** with fluorine substituent and perchlorate counter anion had the lowest ferric reducing power. It is assumed that the Ag(I) center in **Q6** which is tricoordinate, could be responsible for the enhanced activity. **Q10** has the thiophene moiety with a lone pair of electrons on its thiophene. However, despite a fluoro substituent in **Q1** and a chloro substituent in **Q3**, the two complexes had the lowest activities, with IC_50_ values of 4.97 and 4.75 mg/mL. The counter anions in both complexes is a nitrate and that could be responsible for their low activities. The bar chart in Figure 4 represent **Q1**–**Q15** free radical scavenging activity by FRAP assay.

Summarily, most of the complexes tested had significant antioxidant activity. The antioxidant data obtained for complexes **Q1**–**Q15** clearly showed that complexes with either a fluorine substituent, thiophene or a benzothiazole moiety had good reducing power. This is an evidence of their effective antioxidant activities. Thus, these complexes are potential synthetic antioxidant supplements required to protect the body against free radical damages.

### 2.6. DNA Binding Studies

Deoxyribonucleic acid (DNA) is a molecule possessing genetic information in living cells. It is an important molecular target for anticancer agents because of its involvement in gene expression and protein synthesis, which are fundamental steps in cell division and growth [75]. DNA binding studies are significant when developing novel therapeutic drugs. Their interactions with potential drugs have been associated with the anticarcinogenic activity of such drugs. This makes the determination of the type of interaction between metal complexes and DNA important. In studying DNA interaction with metal complexes, electronic absorption spectroscopy is one of the most convenient techniques usually used to investigate the possible modes of binding. DNA can interact with metal complexes either through the covalent or non-covalent bond. Non-covalent interaction of the DNA with metal complexes is usually via three major modes namely: intercalations between base pairs, binding into the groove of DNA, and electrostatic binding [76], while the covalent interaction involves the replacement of the DNA N atom by the metal complex′s labile ligand [77].

In this study, we set to examine the extent to which the ligands and the complexes interact with calf thymus DNA (CT-DNA) by monitoring the hyperchromic and hypochromic effects of the electronic absorption titration. Incremental additions of DNA to the test compounds showed prominent hypochromic shifts with intraligand π–π * bands absorption bands between 249 and 372 nm in the absorption spectra of the ligands and complexes studied. The hypochromism is probably due to the formation of strong stacking interaction between the quinoline planar aromatic chromophore and the base pairs of CT-DNA [27] which is an indication of intercalation binding behavior of the compounds to CT-DNA. Intercalators are molecules that stack perpendicular to the DNA backbone without forming covalent bonds or breaking up the hydrogen bonds between DNA bases. The *p*-orbital of the compounds could be coupled with the *p*-orbital of the base pairs, which decreases the transition energy and induces hypochromism.

In complexes **Q1**–**Q15**, a hypochromic shift between 6.27 and 80.49% in the absorption bands with slight bathochromic between 1 and 3 nm was observed in all the compounds (Supplementary Materials Figures S6–S19) except for **Q1**, **Q6**, and **Q11**, whose absorption bands are accompanied by a slight hypsochromic shift between 2 and 4 nm as the concentration of the CT-DNA was increased. **L1**–**L5**, **Q1**–**Q15** and AgNO_3_, AgClO_4_, and AgCF_3_SO_3_ in the absence and presence of CT-DNA are shown in Figures S1–S22 (see Supplementary Materials).

AgNO_3_, AgClO_4_, and AgCF_3_SO_3_ were used as the standard in this study and the titration of these metal salts against CT-DNA also showed notable hypochromic shifts of 23.64–50.86% with absorption bands between 263 and 274 nm. The silver salts: A hypochromic shift in the absorption bands with a slight bathochromic shift of ~2 nm was observed in the spectra of the metal salts (Supplementary Materials Figures S20–S22).

**Figure 5:**
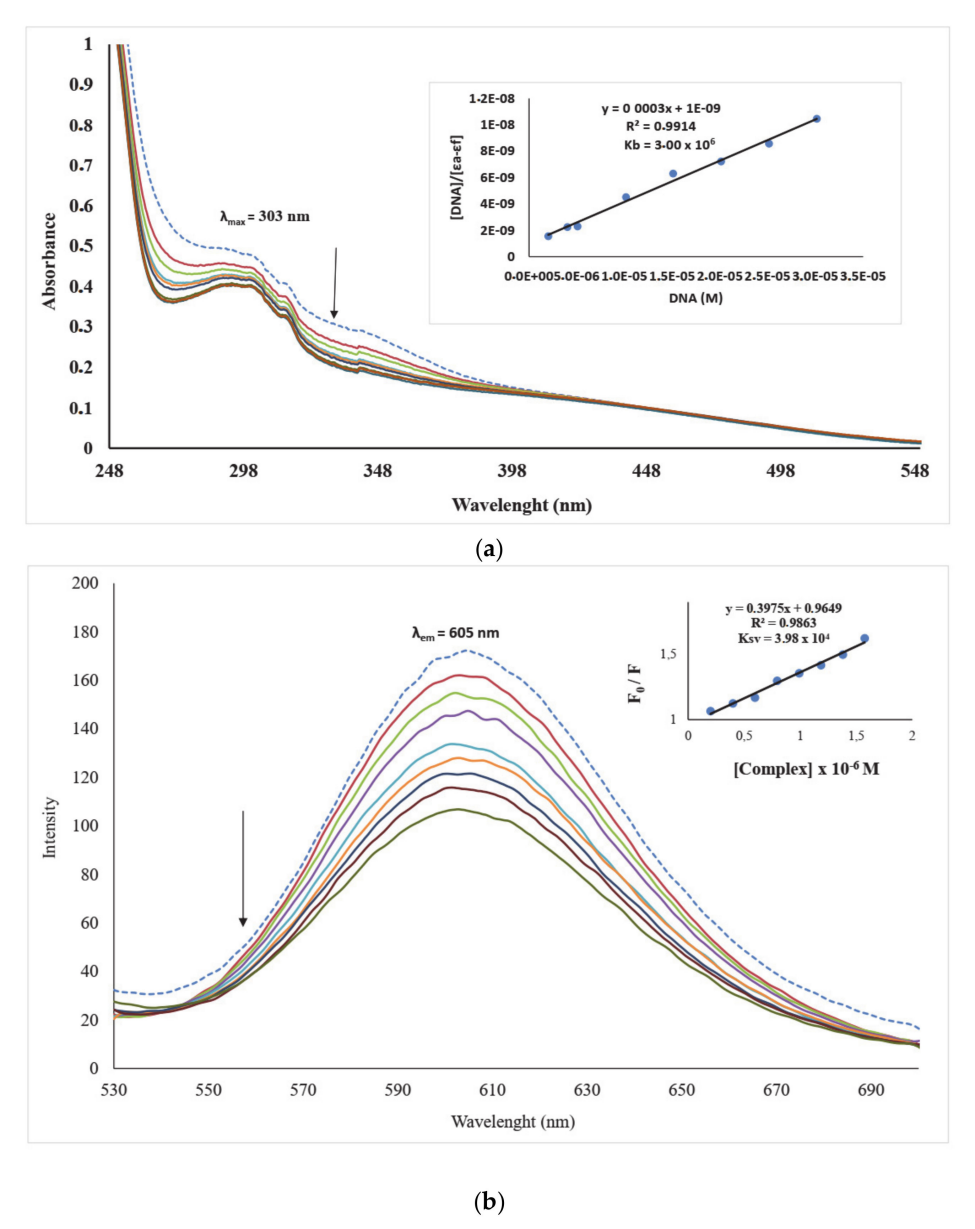

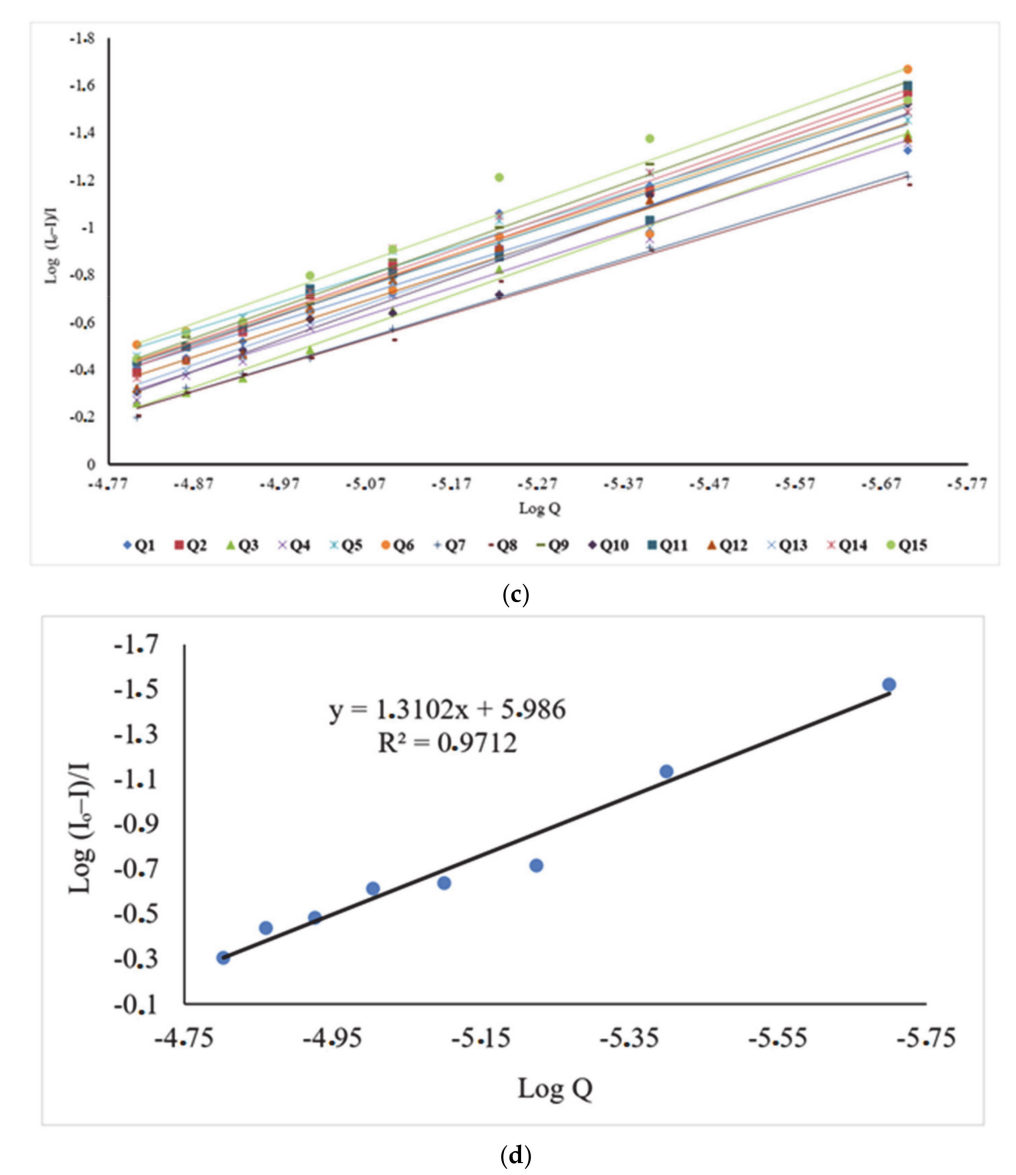
(**a**) Electronic Absorption Spectra of complex **Q8** at 3.0 × 10^6^ M in the absence (dashed line) and the presence of different concentrations of CT-DNA (0–3.0 × 10^5^ M) at 303 nm λ_max_. (inset) A Stern–Volmer plot of **Q8** interaction with CT- DNA. (**b**) Fluorescence spectra of EB-CT-DNA in the absence (dashed line) and the presence of a different concentration of complex **Q8**. (inset) The stern–Volmer plot of **Q8** interaction with EB-CT- DNA. (**c**) Stern–Volmer plot of Complexes **Q1**–**Q15** interaction with EB-CT- DNA. (**d**) The double-logarithmic plot of Complex **Q10**.

The intrinsic binding constant of **L1**–**L5**, **Q1**–**Q15** and AgNO_3_, AgClO_4_, and AgCF_3_SO_3_ were evaluated using the Wolfe–Shimer equation (Equation (5)) by determining the ratio of the slope to the intercept from the plot of [DNA]/(ε_a_ − ε_f_) versus [DNA]. The binding affinity of the free ligands and free metal salts are lower than those of complexes **Q1**–**Q15**, an indication that the ligands binding affinities were enhanced on complexation of ligands to Ag(I). The binding constant obtained lie between 3.33 × 10^5^ and 3.0 × 10^6^ M^−1^ for **Q1**–**Q15**, between 7.50 × 10^4^ and 1.38 × 10^5^ in **L1**–**L5**, and between 2.00 × 10^5^ and 3.30 × 10^5^ for AgNO_3_, AgClO_4_, and AgCF_3_SO_3_ (Table 4). **Q8** had the highest binding affinity (Figure 5a) with a calculated intrinsic binding constant (*K_b_*) of 3.00 × 10^6^, followed by **Q3** and **Q7** with 1.57 × 10^6^ and 1.5 × 10^6^, respectively. The high binding affinities of the three complexes (**Q3**, **Q7**, and **Q8**) are probably due to the π-electron interactions of the quinolinyl with the base pairs of the DNA [78]. In addition, the presence of electron-withdrawing chlorine atoms in para-position in **Q3** and **Q8** and the presence of benzothiazole moiety in **Q7** probably also contributes to the binding affinity to CT-DNA. High DNA interaction with similar compounds has been reported for complexes with electron withdrawing group and benzothiazole moiety [78,79,80]. This interaction favors the aromatic environment of the CT-DNA base pairs resulting in strong structural perturbations in the DNA molecule. These perturbations increase the distance between the adjacent base pairs [27]. The order of the interaction of complexes **Q1**–**Q15** and their metal salts with DNA are **Q8** > **Q3** > **Q7** > **Q11** > **Q9** = **Q10** > **Q12** > **Q13** > **Q1** = **Q4** = **Q5** = **Q15** > **Q6** > **Q14** > **Q2** > AgClO_4_ > AgNO_3_ > AgCF_3_SO_3_. The binding mode needs to be proved by further studies, whereas ligands **L1**–**L5** were exempted since their binding affinity to CT-DNA are lower than those of the complexes.

#### Fluorescence Competitive Displacement Studies

Further studies on binding interactions of **Q1**–**Q15** and the silver salts with CT-DNA were done using fluorescence competitive displacement assay. Although the complexes absorb UV radiation, they do not fluoresce. The fluorescence competitive displacement study is based on the displacement of ethidium bromide from the CT-DNA-EB adduct by the test compounds. Ethidium bromide, a well-known intercalating agent, is a planar cationic dye that fluoresces weakly in phosphate buffer saline solution but emits intensely in the presence of CT-DNA due to the strong intercalation between the DNA double helix base pairs [81]. The intense fluorescence of the ethidium bromide in the presence of CT-DNA can be quenched by introducing another molecule that can displace the bound ethidium bromide. A phenomenon known as fluorescence quenching [80]. The incremental addition of the complexes concentration to ethidium bromide pre-treated with CT-DNA resulted in an appreciable decrease in the ethidium bromide fluorescent intensity at 605 nm (Figure 5b and Figures S23–S39). The gradual decrease in the intensity of the EB pre-treated with CT-DNA upon addition of **Q1**–**Q15** complexes could be as a result of the complexes interacting with DNA via intercalation.

The relative binding interaction of the complexes with CT-DNA was determined by the quenching strength, *K_sv_* calculated from the slope of the linear quenching plot of F_o_/F *versus* [complex] (Equation (6)). The *K_sv_* values for **Q1**–**Q15** were between 2.03 and 3.98 × 10^4^ M^−1^ and those of the silver salts are between 3.01 and 3.31 × 10^4^ M^−1^ (Table 4). These *K_sv_* values indicated the displacement of ethidium bromide by **Q1**–**Q15** and confirmed binding via intercalation mode to CT-DNA. The complexes have high quenching efficiency and a binding affinity to CT-DNA. Complex **Q8** (Figure 5b) had the highest binding affinity to CT-DNA which is consistent with the compound with the highest intrinsic *K_b_* constant (Figure 5a). The quenching of the fluorescence intensity of EB-CT-DNA adduct by the complexes was further used to determine the binding constant (K_bin_) and the number of binding sites (*n*).

Fluorescence quenching can take place via two mechanisms, namely, static quenching and dynamic quenching. The static quenching involves the collision of the fluorophore and the quencher (complex) in the ground state while the dynamic quenching involves the collision of the fluorophore and the quencher in the excited state. In order to determine the type of quenching taking place, two approaches are commonly used, namely: temperature-dependent approach and the Stern–Volmer plot approach.

The Stern–Volmer plot approach was used based on the fact that the Stern–Volmer plots for **Q1**–**Q15** (Figure 5c), were linear an indication of only one mechanism for the quenching fluorescence, i.e., either dynamic or static. Evaluation of *K_q_* using Equation (1) would, therefore, suggest the quenching mechanism through which the complexes quench ethidium bromide emission.

(1) $$K_{sv}=K_{q}\tau_{o}$$

where *K_sv_*, *K_q_*, and *τ*_o_ represent Stern–Volmer quenching constant, bimolecular quenching constant, and an average lifetime of the fluorophore in the absence of the quencher, respectively. τₒ normally takes about 10^−8^ s in biomacromolecules [82]. The values of *K_q_* were found to be between 2.03 and 3.98 × 10^12^ M^−1^S^−1^ (Table 4), which is greater than the maximum diffusion collision quenching rate constant of various quenchers of biological macromolecules (2 × 10^10^ M^−1^S^−1^) [82]. These values showed that the quenching by **Q1**–**Q15** was not initiated by a dynamic mechanism but happened via a static quenching mechanism; thus, at the ground state, complexes formed between quenching molecules and fluorescence molecules are stabilized [83].

The double-logarithmic Equation (2) was used in determining the binding constant of complexes **Q1**–**Q15** and CT-DNA.

(2) $$\log\frac{F_{o}-F}{F}=\log K_{b}+n\;\log\left[Q\right]\;$$

A plot of log Fₒ − F/F vs. log [Q] of complexes **Q1**–**Q15** gave a straight line with a slope of *n* and y-axis intercept of log *K_b_* (Figures S40–S56) (where Fₒ and F denote the relative fluorescence intensities of CT-DNA in the absence and presence of the quencher, respectively, while [Q] is the concentration of the quencher, *K_b_* is the binding constant and *n* is the number of binding sites. The values of *n* for complexes **Q1*–*Q15** are approximately equal to 1 (Table 4), suggesting that all the complexes bind to one reactive site on the CT-DNA. The corresponding results are shown in Table 4. Complex **Q10** (Figure 5d) showed the most binding affinity to CT-DNA with a binding constant (K_bin_) equal to 9.61 × 10^5^ M^−1^ and the number of binding sites (*n*) approximately equal to 1.

Based on the spectroscopic studies of the interaction of **L1**–**L5** and complexes **Q1**–**Q15** with CT-DNA, it can be summarized that the test compounds in this study had a strong binding affinity to calf thymus DNA, especially those with either electron-withdrawing substituent or benzothiazole moiety. The binding interaction of the compounds to CT-DNA was via intercalation mode mainly due to the planarity of the chelating quinolinyl ligand and the competitive study with EB shows that the complexes can displace EB from the CT-DNA-EB adduct and compete for the DNA-binding sites with EB, which is usually characteristic of an intercalative interaction of compounds with DNA.

### 2.7. Albumin Binding Studies Using Absorption Spectroscopy Titration

Interaction of potential therapeutic drugs with carrier proteins is very important in bio-inorganic studies because the binding properties of the drug–protein reveal the absorption, transportation, distribution, and metabolism of the drugs. For this reason, the protein-binding study of **Q1** to **Q15** is an important way of studying the compounds metabolism in vivo.

**Figure 6:**
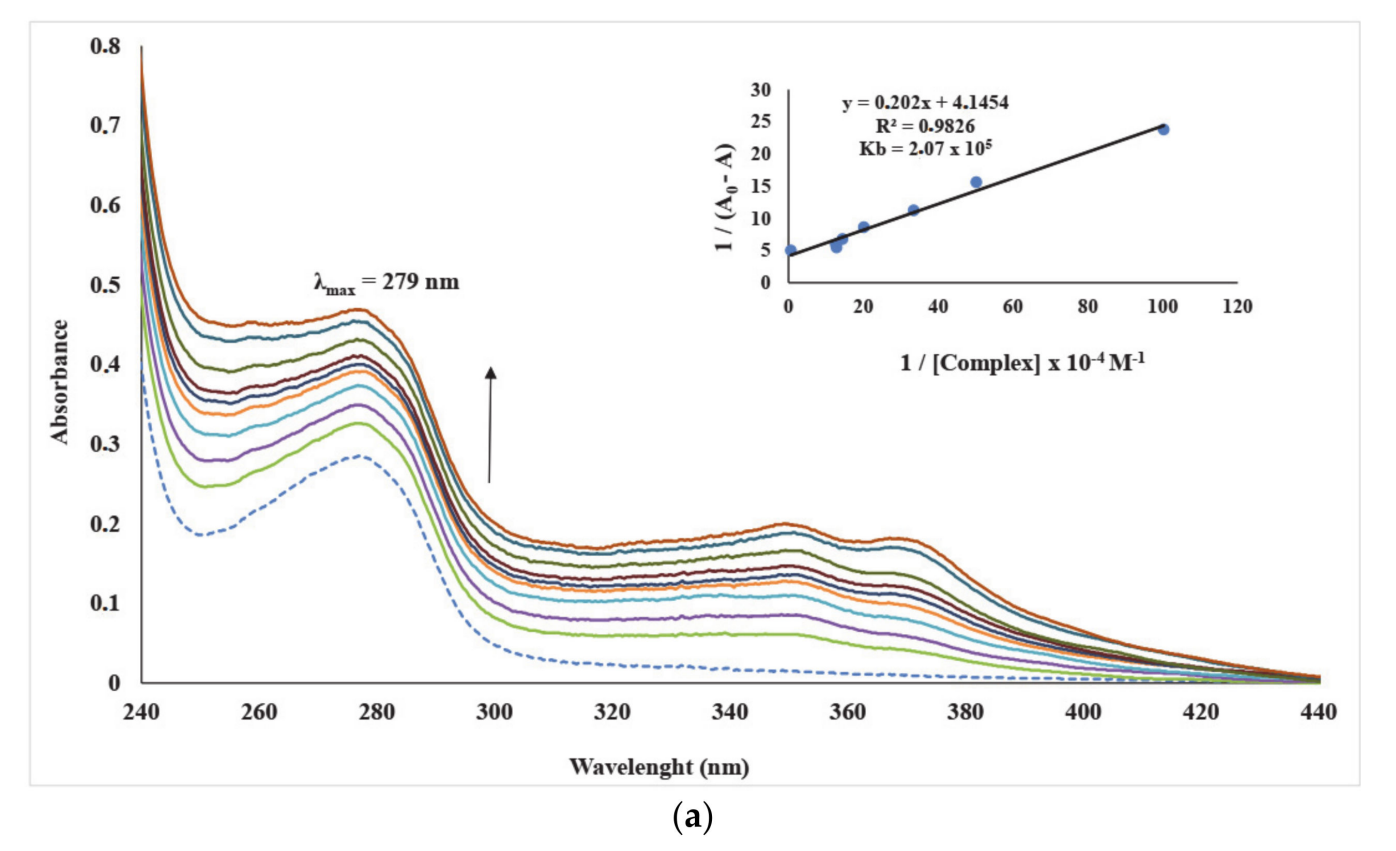

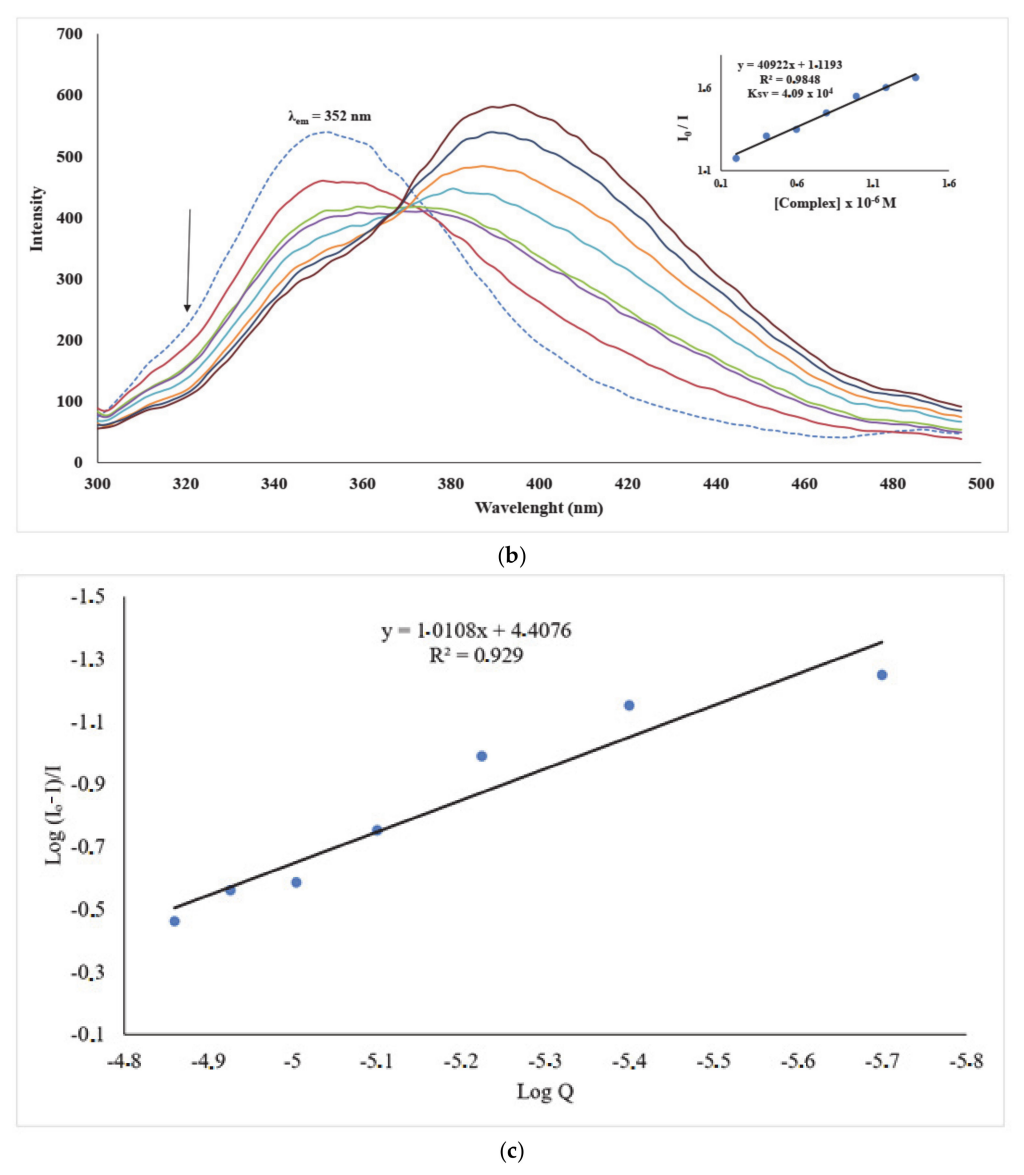
(**a**) Electronic Absorption Spectra of BSA in the absence (dashed line) and the presence of different concentrations of complexes **Q12**. (inset) Plot of 1/(Aₒ − A) vs. 1/[Complex] × 10^−4^ M^−1^. (**b**) Fluorescence emission spectra of BSA in the absence(dashed line) and the presence of a different concentration of complex **Q11**. Inset: A Stern–Volmer plot of the interaction of **Q11** with BSA. (**c**) The double-logarithmic plot of BSA–Complex Q6 interactions.

Bovine serum albumin (BSA) was used as the protein standard in this study because of its close resemblance to human serum albumin (HSA), its stability, high solubility, neutrality in many biochemical reactions and low cost [84]. In protein binding study, absorption spectroscopy titration is a known method used in studying the conformational changes in proteins, and this protein is characterized by an intense absorbance peak at 280 nm depicting the microenvironment around tyrosine and tryptophan residue polarity [85]. On each addition of various concentrations of complexes **Q1**–**Q15** and the silver salts to a constant BSA concentration, the intensity of the BSA absorbance intensified between 260 and 300 nm with slight shifts (Figures S57–S73). This shows some binding interactions between the complexes and BSA exist. *K_b_* in this instance was determined from the intercept to the slope ratio of the linear curve of a plot of 1/[A-A°] vs. 1/[complex]. The *K_b_* of all the complexes were in the range of 2.16 × 10^4^–2.07 × 10^5^ M^−1^ (Table 4) which are in conformation with reported related BSA binding constants, normally between 10^4^ and 10^6^ M^−1^ [86]. This range is a recognized range for drug-carrier complexes. The silver salts with *K_b_* value between 4.46 and 4.82 × 10^4^ is lower than those of the complexes except **Q1** (Figure S57) and **Q13** (Figure S68). This shows that the binding affinity of the ligands to BSA was enhanced on coordination. Complex **Q12** had the highest binding affinity (Figure 6a), followed by **Q4** (Figure S60) and **Q15** (Figure S70). The influence of **Q4**, **Q12**, and **Q15** ligand substituents on their binding affinity to BSA can be related to similar literature reports [87,88,89]. The order of binding constant for the complexes is: **Q12** > **Q15** > **Q4** > **Q8** > **Q7** > **Q11** > **Q10** > **Q2** > **Q14** > **Q5** > **Q6** > **Q9** > **Q3** > **Q1** > **Q13**.

#### Fluorescence Quenching of Bovine Serum Albumin in the Presence of Complexes **Q1**–**Q15**

The fluorescence quenching of protein can provide information on protein binding mechanisms, binding mode, binding sites and the rates of interaction with drugs or complexes of study [86]. When BSA is excited at 280 nm, it fluoresces strongly between 300 and 400 nm leading to excitation in tyrosine (Tyr) and tryptophan (Trp) residues in proteins. In the presence of a small molecule, BSA has the possibility of undergoing different types of interactions such as hydrophobic, hydrogen bonding, van der Waals, and electrostatic interactions. The ability of a complex to quench BSA fluorescence intensity depends on the hydrophobic interaction between the Trp and the hydrophobic ligand of a complex. The interaction is either enhanced or stabilized by the surrounding amino acid residues [90]. The binding of BSA to compounds was studied by fluorescence measurements at room temperature.

The free BSA has strong fluorescence emission around 350–360 nm when excited at 280 nm and on adding complexes **Q1**–**Q15**, the BSA fluorescence intensity of only **Q1**, **Q6**, and **Q11** decreased gradually as the concentration of these three complexes was increased (Figure 6b and Figures S74–S75). The reduction in the fluorescence intensity of **Q1**, **Q6**, and **Q11** is accompanied by a redshift of the emission wavelength between 2 and 15 nm, an indication that the interaction of **Q1**, **Q6**, and **Q11** with BSA causes a conformational change in the protein structure [91]. Furthermore, an isosbestic point between 366 and 388 nm in the spectra of the complex–BSA interaction was observed, implying the formation of a stable complex–protein complex. **Q11** (Figure 6b) quenched the intrinsic fluorescence of BSA more than **Q1** and **Q6**.

The inability of all the complexes (with the exception of **Q1**, **Q6**, and **Q11)** to quench the intrinsic fluorescence of BSA after the addition of between 1 and 2 μM concentration could be structurally related because from our previous report where pyridine moiety was involved as against the quinoline moiety in this study, a contrary behavior regarding the interaction of the protein with complexes of similar substituents and moieties were reported. Complexes **Q1**, **Q6**, and **Q11** mode of coordination are different from other complexes mode of coordination which means their interaction with BSA was probably via the aniline substituent while the quinoline ring of **L2**, **L3**, **L4**, and **L5** complexes were not planar enough and are not readily available for quenching the active site of the protein buried in the hydrophobic environment. This suggests no interaction of the complexes with the BSA protein.

The quenching mechanism of the **Q1**, **Q6**, and **Q11** was evaluated using the Stern–Volmer Equation (Equation (7)). *K_sv_* values for the complexes were obtained from the slope of the plot of I_0_/I vs. [*Q*] and found to be between 2.50 × 10^4^ and 4.09 × 10^4^ M^−1^. The calculated values of K_sv_ and *K*_q_ for the interaction of the complexes with BSA are given in Table 4. The linearity of the Stern–Volmer plots (inset Figures S64–S71) is an indicator of either a static or a dynamic quenching mechanism. The *K*_q_ values obtained for the complexes are between 2.50 × 10^12^ and 4.09 × 10^12^ M^−1^S^−1,^ which are greater than the maximum scatter collision-quenching constant of biomacromolecules (2 × 10^10^ M^−1^ S^−1^). This indicates that the interactions between the complexes and BSA are static. Therefore, the binding constant (K_bin_) and the number of binding sites (*n*) can be determined using Scatchard Equation (3):

(3) $$\log\frac{I_{o}-I}{I}=\log K_{\mathrm{bin}}+n\log\left[Q\right]\;$$

The *n* and K_bin_ can be calculated from the slope and the intercept of the double logarithm regression curve of log(Iₒ − I)/I versus log[Q] (Figures S76–S77). The values of the binding constant (K_bin_) suggest BSA has a moderate binding affinity to the complexes. Complex **Q6** (Figure 6c) has the highest binding affinity to BSA. The number of the binding site (*n*) for **Q1**, **Q6**, and **Q11** are approximately one; this suggests that one molecule of the complexes bound per bovine serum albumin. The binding constant values of **Q1**, **Q6**, and **Q11** fall within 10^4^–10^6^ M^−1,^ which is the recognized values of non-covalent interaction of BSA with drugs [92].

In summary, the interaction of the complexes with BSA causes conformation changes of both tyrosine and tryptophan environments due to the hydrophobic interactions of the complexes′ ligand with BSA. In essence, the complexes possess a moderate binding affinity for protein. Thus, most of the complexes can be stored in protein and can be easily released in desired target areas.

### 2.8. Anticancer Studies

Cancer is one of the major life-threatening diseases worldwide. The clinical application of chemotherapy is still considered a major method in treating cancer, therefore, the development of novel effective anticancer drugs is of importance. For this study, we picked complexes **Q6**, **Q7**, and **Q9** based on the significant effect the fluorine and methyl substituent and benzothiazole moiety has on their complexes′ biological activities. All these complexes have perchlorate as their anion since perchlorate has been identified to likely enhance biological activities as seen in the antimicrobial, antioxidant, activities, and DNA and protein binding assays.

The in vitro cytotoxicity of metal complexes, **Q6**, **Q7**, **Q9**, and cisplatin toward human embryonic kidney 293 (HEK293), cervical cancer cell line (HELA), breast cancer cell line (MDA-MB231) and neuronal cancer cell line (SHSY5Y) were examined via standard method. The *EC*_50_ and the selectivity index (SI) values obtained are summarized in Table 5. The result obtained indicate that there is a structure activity relationship of the complexes in terms of cytotoxicity of the complexes. **Q7** and **Q9** showed potent cytotoxicity activity towards HELA cells with *EC*_50_ values of 22.80 ± 3.11 and 22.34 ± 4.86 μM, respectively which is lower than cisplatin (EC_50_ > 50). **Q7** and **Q9** also showed slight cytotoxicity against MDA-MB231 and SHSY5Y.

The remarkable cytotoxicity recorded for **Q7** and **Q9** towards HELA cells might be due to the presence of benzothiazole moiety and methyl substituent, respectively. In similar reports, Saeed et al. 2010 [93] in their anticancer study of various benzothiazole derivatives obtained IC_50_ values in the range 38–46 μM, and Sreelatha et al. 2014 [94], identified the influence of methyl group in the anticancer activity of naphthoquinone amide derivatives against HELA with IC_50_ value of 20 μM.

The positive charge of the metal is also known to influence the cytotoxic activity of the complexes. The charge increases the acidity of the coordinated ligand that accept protons [95]. **Q6** had low cytotoxicity activity (EC_50_ > 100 μM) towards all the cancer cell lines which could be attributed to their unusual mode of coordination. In comparison, the cytotoxic activity of the complexes toward HeLa cell follows the order **Q9** > **Q7** > **Q6**.

In MDA-MB231, **Q7** and **Q9** with EC_50_ of 41.62 ± 4.25 μM and 38.84 ± 4.33 μM, respectively, showed fair cytotoxicity when compared to cisplatin (EC_50_ = 22.68 ± 4.89 μM), similarly, **Q7** and **Q9** had moderate anticancer activity when compared with cisplatin (EC_50_ = 19.74 ± 3.77 μM) against SHSY5Y with EC_50_ value of 30.33 ± 4.67 μM and 47.23 ± 3.34 μM respectively.

All the complexes are less toxic toward the normal cell (HEK293), with EC_50_ ≥ 100 μM and are significantly higher than that of cisplatin with EC_50_ values of 14.2 ± 3.8 μM. The SI data shown in Table 5 demonstrates the differential activity of the complexes and cisplatin, the greater the SI value is, the more selective it is. An SI value of less than two indicates the general toxicity of the compound. Based on this, the SI values indicate that complexes **Q7** and **Q9** displayed excellent selectivity towards all the cancer cell lines, while complexes **Q6** had better selectivity towards all the cancer cell lines better than the Cisplatin standard.

In conclusion, the cytotoxicity activity of the studied complexes is coordination geometry, structural substituent, and moiety dependent. The introduction of benzothiazole moiety and methyl substituent on the phenyl ring enhance the anticancer activity especially towards HELA, as a consequence, complexes **Q7** and **Q9** are promising anti-cervical cancer agents. Notably, all the tested complexes are benign to the normal cell and they all demonstrated a remarkable selectivity for cancer cells over normal cells.

## 3. Experimental

### 3.1. Materials and Instrumentation

Ethanol 99.5% (Aldrich, St. Louis, MO, USA), diethyl ether 99.8% (Aldrich, St. Louis, MO, USA), DMSO–*d6* 99.8% (Merck, Darmstadt, Germany), 2-quinolinecarboxaldehyde 99% (Aldrich, St. Louis, MO, USA), 2-aminobenzenethiol 99.5% (Aldrich, St. Louis, MO, USA), 2-thiophenemethylamine > 92% (MerckDarmstadt, Germany), *p*-toluidine 99.5% (Aldrich, St. Louis, MO, USA), 2-fluoroaniline 99.5% (Aldrich, St. Louis, MO, USA), 4-chloroaniline 99.5% (Aldrich, St. Louis, MO, USA), dichloromethane 99% (Aldrich, St. Louis, MO, USA), calf-thymus DNA (CT-DNA), ethidium bromide 95% (Aldrich, St. Louis, MO, USA), bovine serum albumin 99% (Aldrich, St. Louis, MO, USA)*,* and nitrogen gas, 5.0 technical grade (Air flex Industrial Gases, Pietermaritzburg, Africa) were purchased from local suppliers. All chemicals were in analytical grade and used as received, while most of the solvents were dried using conventional techniques.

^1^H NMR and ^13^C NMR spectra were recorded on a BRUKER 400 MHz spectrometer in DMSO–*d6* and acetone-*d6*. Chemical shift values are reported in parts per million (ppm) relative to the solvent residual peaks in DMSO-*d6* and acetone-*d6*; 2.5 and 2.05 ppm respectively for ^1^H NMR and 39.5 and 29.4 ppm respectively for ^13^C NMR. The splitting patterns in ^1^H NMR spectra are reported as s for singlet, d for doublet, m for multiplet while J (the coupling constant is given in Hertz). The infrared spectra were recorded using a PerkinElmer Spectrum 100 FT-IR spectrometer, and the data are reported as percentage transmittances at the respective wavenumbers (cm^−1^), between 4000 and 650 cm^−1^. The mass spectra were recorded using Shimadzu LCMS-2020 instrument with only molecular ions (M^+^) and major fragmentation peaks being reported with intensities quoted as percentages of the base peak. Elemental analyses were performed on Thermal-Scientific Flash 2000 CHNS/O analyzer. All melting points were determined using the Stuart Scientific melting point apparatus. Absorption studies of CT-DNA and protein were recorded using Shimadzu UV-1800 UV-Vis Spectrophotometer. The photoluminescence studies of CT-DNA and protein were recorded using PerkinElmer LS 45 Fluorescence spectrometer.

### 3.2. Synthesis of Quinolinyl Schiff Bases **L1**–**L5**

**L1**–**L5** were synthesized using a modified method from the literature [96,97] for similar ligands. The ligands were synthesized by the addition of a solution of 2-quinolinecarboxaldehyde (1 mmol, 0.16 g) in anhydrous ethanol (10 mL) to a solution of 2-fluoroaniline (1 mmol, 0.10 mL), 2-aminothiophenol (1 mmol, 0.10 mL), 4-chloroaniline (1 mmol, 0.13 g), *p*-toluidine (1 mmol, 0.11 g), and 2-thiophenemethylamine (1 mmol, 0.10 mL), respectively, in anhydrous ethanol (10 mL) in the presence of glacial acetic acid. The resulting reactions for **L1**, **L3**, and **L4** were refluxed at ca. 80 °C for 4 h while **L2** and **L5** were subjected to constant stirring at ambient temperature for 6 h. **L2**, **L3**, and **L4** solutions were evaporated to one-third of their initial volume under reduced pressure after which 10 mL of ether was added to each solutions to afford precipitates. The precipitates were isolated via filtration, washed twice with 10 mL cold ether, recrystallized from ethanol and dried over anhydrous magnesium sulfate. **L1** and **L5** on the other hand were evaporated completely under pressure. The oil obtained was recrystallized from ethanol and dried over anhydrous magnesium sulfate.

### 3.3. Single-Crystal X-ray Analysis

Crystal evaluation and data collection of **Q1**, **Q6**, **Q7**, **Q12**, and **Q14** were recorded on a Bruker Apex Duo diffractometer equipped with an Oxford Instruments Cryojet operating at 100 (2) K and an Incoatec microsource operating at 30 W power. The data were collected with Mo Kα (λ = 0.71073 Å) radiation at a crystal-to-detector distance of 50 mm using omega and phi scans. The data were reduced with the programme SAINT [98] using outlier rejection, scan speed scaling, as well as standard Lorentz and polarization correction factors. A SADABS [99] semi-empirical multi-scan absorption correction was applied to the data.

The structures of **Q1**, **Q6**, **Q7**, **Q12**, and **Q14** were solved by the direct method using the SHELXS [100] program and refined. The visual crystal structure information was performed using ORTEP-3 [101], system software. Non-hydrogen atoms were first refined isotropically and then by anisotropic refinement with a full-matrix least-squares method based on *F^2^* using SHELXL [102]. All hydrogen atoms were positioned geometrically, allowed to ride on their parent atoms, and refined isotropically. The crystallographic data and structure refinement parameters for **Q1**, **Q6**, **Q7**, **Q12**, and **Q14** are given in Table S3 (See Supplementary Materials).

In complex **Q7**, the perchlorate ion including the bidentate ligand was disordered over two positions with each component having 50% site occupancy. The bridging ligand was disordered over a special position with an equal site occupancy factor. PART-1 instruction was used to model the disorder.

### 3.4. Synthesis of **Q1**–**Q15**

**Q1**–**Q15** were synthesized by dropwise addition of ethanolic solution (*ca.* 15 mL) of each of **L1**–**L5** (1 mmol) to an ethanolic (*ca.* 10 mL) solution of silver nitrate (0.5 mmol, ca. 0.09 g), silver perchlorate (0.5 mmol, ca. 0.10 g), and silver trifluoromethanesulfonate (0.5 mmol, ca. 0.13 g), respectively, under constant stirring in the dark. The reactions were carried out under nitrogen at ambient temperature for 6 h. The resulting precipitates were isolated using a vacuum filter. Afterward, the precipitates were washed with cold ethanol (10 mL × 2) followed by cold ether (10 mL × 2) and dried in vacuo. The obtained complexes were recrystallized by dissolving the complexes in dichloromethane and layering with toluene.

#### 3.4.1. [Ag(**L1**)_2_]NO_3_ **Q1**

Yellow solid, 0.50 g, 94%, Melting point; 152–153 °C. ^1^H-NMR (400 MHz, DMSO-*d6*, δ ppm): 8.93 (1H, s, Hg-CH=N-), 8.62 (1H, d, *J* = 8.69 Hz, He-C_9_H_6_N), 8.31 (1H, d, *J* = 8.55 Hz, Hf-C_9_H_6_N), 8.18 (1H, d, *J* = 8.46 Hz, Hd-C_9_H_6_N), 8.12 (1H, d, *J* = 8.46 Hz, Ha-C_9_H_6_N), 7.89 (1H, m, Hb-C_9_H_6_N), 7.75 (1H, m, Hc-C_9_H_6_N), 7.55 (1H, m, Hj-C_6_H_4_), 7.34 (3H, m, Hk, i, h-C_6_H_4_), 6.95 (1H, m, Ho-C_6_H_4_), 6.86 (1H, m, Hm-C_6_H_4_), 6.75 (1H, m, Hn-C_6_H_4_), 6.50 (1H, m, Hl-C_6_H_4_), 5.04 (2H, s, Hp-NH_2_). ^13^C-NMR (400 MHz, DMSO-*d6*, δ ppm 25 °C): δ = 162.97 (C10-C=N-), 156.35 (C22-C_6_H_4_), 153.11 (C16-C_6_H_4_), 149.43 (C1-C_9_H_6_N), 146.99 (C9-C_9_H_6_N), 137.74 (C7-C_9_H_6_N), 136.36 (C11-C_6_H_4_), 136.23 (C17-C_6_H_4_), 130.70 (C6-C_6_H_4_), 129.36 (C3-C_6_H_4_), 128.89 (C2-C_6_H_4_), 128.58 (C14-C_6_H_4_N), 128.37 (C5-C_9_H_6_N), 128.18 (C4-C_6_H_4_N), 125.18 (C13-C_9_H_6_N), 125.14 (C19-C_6_H_4_), 124.41 (C12-C_6_H_4_), 124.38 (C20-C_6_H_4_), 121.73 (C8-C_9_H_6_N), 119.60 (C15-C_6_H_4_), 116.45 (C18-C_6_H_4_), 116.17 (C21-C_6_H_4_). FT-IR (cm^−1^): (-C=N-) 1612, (C=N quinolinyl) 1588, (C-F) 1500. UV/Vis (CH_3_CN): *λ*_max_ 234, 276, 326 nm. MS: *m/z* Calcd. For [C_22_H_16_AgF_2_N_3_]: 468; found [Ag(**L1**)–2F + Na + CH_3_CN]^+^: 495 (79%), 496 (26%), [Ag(**L1**) + 2EtOH]^+^: 583 (53%), 584 (18%). Anal. Calcd. (%) for [C_22_H_17_AgF_2_N_4_O_3_]: C, 49.74; H, 3.23; N, 10.55; found (%): C, 49.67; H, 3.14; N, 10.38.

#### 3.4.2. [Ag(**L2**)_2_]NO_3_ **Q2**

Brown solid, 0.39 g, 56%, Melting point; 266–267 °C. ^1^H-NMR (400 MHz, (DMSO, δ ppm): 8.62 (2H, d, *J* = 8.58 Hz, Hg-C_6_H_4_-), 8.46 (2H, d, *J* = 8.53 Hz, He-C_9_H_6_N-), 8.17 (8H, Hj-C_6_H_4_, Hd-C_9_H_6_N, Ha-C_9_H_6_N, Hf-C_9_H_6_N), 7.88 (2H, t, *J* = 7.68 Hz, Hb-C_9_H_6_N), 7.72 (2H, d, *J* = 7.50 Hz, Hc-C_9_H_6_N), 7.58 (4H, m, Hh-C_6_H_4_, Hi-C_6_H_4_). ^13^C-NMR (400 MHz, (DMSO, 25 °C): δ = 168.94 (C9-C_9_H_6_N), 153.60 (C16-C_6_H_4_), 150.38 (C10-C=N-), 147.04 (C1-C_9_H_6_N), 138.04 (C7-C_9_H_6_N), 135.61 (C11-C_6_H_4_), 130.83 (C3-C_9_H_6_N), 128.94 (C2-C_9_H_6_N), 128.77 (C5-C_9_H_6_N), 128.23 (C4-C_9_H_6_N), 128.03 (C6-C_9_H_6_N), 126.76 (C14-C_6_H_4_), 126.35 (C13-C_9_H_6_N), 123.53 (C12-C_6_H_4_), 122.65 (C15-C_6_H_4_), 118.12 (C8-C_9_H_6_N). FT-IR (cm^−1^): (Ar-CH) 3051, (C-S-C) 750, (quinolinyl) 1588. UV/Vis (CH_3_CN): *λ*_max_ 217, 284, 318, 335, 350 nm. MS: *m/z* Calcd. For [C_32_H_20_AgN_4_S_2_]: 632,53; found [Ag(**L2**)_2_ + H)]^+^: 633 (100%), 631 (90%), 634 (38 %). Anal. Calcd. (%) for [C_32_H_20_AgN_5_O_3_S_2_]: C, 55.34; H, 2.90; N, 10.08; found (%): C, 55.23; H, 2.85; N, 9.92.

#### 3.4.3. [Ag(**L3**)_2_]NO_3_ **Q3**

Brown solid, 0.67 g, 96%, Melting point; 182–183 °C. ^1^H-NMR (400 MHz, (CD_3_)_2_CO, δ ppm): 9.08 (2H, s, Hg-CH=N-), 8.72 (2H, d, *J* = 8.47 Hz, He-C_9_H_6_N), 8.27 (2H, d, *J* = 8.46 Hz, Hf-C_9_H_6_N), 8.17 (4H, m, Hd, a-C_9_H_6_N), 7.90 (2H, m, Hb-C_9_H_6_N), 7.77 (2H, m, Hc-C_9_H_6_N), 7.56 (8H, m, Hi, h-C_6_H_4_). ^13^C-NMR (400 MHz, (CD_3_)_2_CO, 25 °C): δ = 152.20 (C10-C=N-), 148.00 (C1-C_6_H_4_), 146.81 (C11-C_9_H_6_N), 138.90 (C9-C_9_H_6_N), 132.52 (C7-C_9_H_6_N), 131.52 (C14-C_9_H_6_N), 131.56 (C6-C_6_H_4_), 129.79 (C13, C15-C_9_H_6_N), 129.75 (C3-C_6_H_4_), 129.68 (C2-C_6_H_4_), 128.94 (C5-C_6_H_4_), 128.61 (C4-C_6_H_4_), 124.16 (C12 & C16-C_9_H_6_N), 122.27 (C8-C_9_H_6_N). FT-IR (cm^−1^): (Ar-CH) 3063, (-C=N-) 1613, (C=N quinolinyl) 1584. UV/Vis (CH_3_CN): *λ*_max_ 212, 257, 313, 331 nm. MS: *m/z* Calcd. for [C_32_H_22_AgCl_2_N_4_]: 641.32; found [Ag(**L3**)_2_]^+^: 641 (100%), 639 (54%), 643 (48%), 642 (31%), [Ag(**L3**) + H + CH_3_CN + Na]^+^: 597 (56%), 595 (23%). Anal. Calcd. (%) for [C_32_H_22_AgCl_2_N_5_O_3_]: C, 54.65; H, 3.15; N, 9.96; found (%): C, 54.32; H, 3.15; N, 9.93.

#### 3.4.4. [Ag(**L4**)_2_]NO_3_ **Q4**

Yellow solid, 0.47 g, 72%, Melting point; 141–142 °C. ^1^H-NMR (400 MHz, DMSO-*d6*, δ ppm): 9.21 (2H, s, Hg-CH=N-), 8.76 (2H, d, *J* = 8.26 Hz, He-C_9_H_6_N), 8.28 (2H, d, *J* = 8.46 Hz, Hf-C_9_H_6_N), 8.14 (4H, m, Hd, a-C_9_H_6_N), 7.86 (2H, m, Hb-C_9_H_6_N), 7.75 (2H, m, Hc-C_9_H_6_N), 7.52 (4H, m, Hi-C_6_H_4_), 7.29 (4H, d, Hh-C_6_H_4_), 2.33 (6H, m, Hj-CH_3_). ^13^C-NMR (400 MHz, DMSO-*d6*, 25 °C): δ = 159.06 (C10-C_9_H_6_N), 151.67 (C1-C_6_H_4_), 146.26 (C11-C_9_H_6_N), 145.71 (C9-C_9_H_6_N), 138.88 (C15-C_9_H_6_N), 138.16 (C7-C=N-), 131.43 (C6-C_6_H_4_), 130.02 (C13 & C16-C_9_H_6_N), 129.35 (C3-C_6_H_4_), 129.19 (C2-C_6_H_4_), 128.58 (C5-C_6_H_4_), 128.33 (C4-C_6_H_4_), 122.75 (C12 & C17-C_9_H_6_N), 122.25 (C8-C_9_H_6_N), 20.64 (C14-C_9_H_6_N). FT-IR (cm^−1^): (Ar-CH) 3050, (-C=N-) 1684, (quinolinyl) 1586. UV/Vis (CH_3_CN): *λ*_max_ 243, 302, 339 nm. MS: *m/z* Calcd. For [C_34_H_28_AgN_4_]: 600.49; found [Ag(**L4**)_2_ + H)]^+^: 601 (100%), 599 (90%), 602 (33%), 600 (23%). Anal. Calcd. (%) for [C_34_H_28_AgN_5_O_3_]: C, 61.64; H, 4.26; N, 10.57; found (%): C, 61.54; H, 4.04; N, 10.38.

#### 3.4.5. [Ag(**L5**)_2_]NO_3_ **Q5**

Brown solid, 0.54 g, 78%, Melting point; 134–135 °C, ^1^H-NMR (400 MHz, DMSO-*d6*, δ ppm): 9.03 (2H, t, *J* = 1.48 Hz, 1.51 Hz, Hg-CH=N-), 8.79 (2H, d, *J* = 8.22 Hz, He-C_9_H_6_N), 8.16 (2H, d, *J* = 7.36 Hz, Hd-C_9_H_6_N), 8.09 (2H, d, *J* = 8.41 Hz, Hf-C_9_H_6_N), 8.01 (2H, d, *J* = 8.32 Hz, Ha-C_9_H_6_N), 7.87 (2H, m, Hb-C_9_H_6_N), 7.76 (2H, m, Hc-C_9_H_6_N), 7.45 (2H, m, Hk-C_4_H_3_S), 7.06 (2H, m, Hi-C_4_H_3_S), 6.97 (2H, m, Hj-C_4_H_3_S), 5.20 (4H, s, Hh-CH_2_). ^13^C-NMR (400 MHz, DMSO-*d6*, 25 °C): δ = 162.60 (C10-C=N-), 149.61 (C9-C_9_H_6_N), 145.82 (C1-C_9_H_6_N), 140.21 (C15-C_4_H_3_S), 139.53 (C7-C_9_H_6_N), 131.67 (C6-C_9_H_6_N), 129.40 (C3-C_9_H_6_N), 128.78 (C2-C_9_H_6_N), 128.32 (C5-C_9_H_6_N), 127.42 (C4-C_9_H_6_N), 127.33 (C13-C_4_H_3_S), 126.96 (C12-C_4_H_3_S), 126.24 (C14-C_4_H_3_S), 123.71 (C8-C_9_H_6_N), 57.40 (C11-CH_2_). FT-IR (cm^−1^): (Ar-CH) 3059, (-C=N-) 1645, (C=N quinolinyl) 1589. UV/Vis (CH_3_CN): *λ*_max_ 288, 320sh, 334sh nm. MS: *m/z* Calcd. for [C_30_H_24_AgN_4_S_2_]: 612.54; found [Ag(**L5**)_2_ + H]^+^: 613 (100%), 611 (85%), 614 (29%), 612 (18%), 615 (10%), [Ag(**L5**) + CH_3_CN]^+^: 400 (30%), 402 (29%). Anal. Calcd. (%) for [C_30_H_24_AgN_5_O_3_S_2_]: C, 53.42; H, 3.59; N, 10.38; found (%): C, 53.14; H, 3.29; N, 10.19.

#### 3.4.6. [Ag(**L1**)_2_]ClO_4_ **Q6**

Brown solid, 0.56 g, 99%, Melting point; 198–199 °C. ^1^H-NMR (400 MHz, DMSO-*d6*, δ ppm): 8.98 (1H, s, Hg-CH=N-), 8.66 (1H, d, *J* = 8.46 Hz, He-C_9_H_6_N), 8.30 (1H, d, *J* = 8.46 Hz, Hf-C_9_H_6_N), 8.18 (1H, d, *J* = 8.26 Hz, Hd-C_9_H_6_N), 8.13 (1H, d, *J* = 8.04 Hz, Ha-C_9_H_6_N), 7.89 (1H, m, Hb-C_9_H_6_N), 7.76 (1H, m, Hc-C_9_H_6_N), 7.57 (1H, m, Hj-C_6_H_4_), 7.34 (3H, m, Hk, i, h-C_6_H_4_), 6.95 (1H, m, Ho-C_6_H_4_), 6.86 (1H, m, Hm-C_6_H_4_), 6.75 (1H, m, Hn-C_6_H_4_), 6.50 (1H, m, Hl-C_6_H_4_), 5.04 (2H, s, Hp-NH_2_). ^13^C-NMR (400 MHz, DMSO-*d6*, δ ppm 25 °C): δ = 162.73 (C10-C=N-), 155.88 (C22-C_6_H_4_), 153.90 (C16-C_6_H_4_), 150.85 (C1-C_9_H_6_N), 150.28 (C9-C_9_H_6_N), 138.64 (C7-C_9_H_6_N), 137.72 (C11-C_6_H_4_), 137.46 (C17-C_6_H_4_), 128.71 (C6-C_6_H_4_), 128.64 (C3-C_6_H_4_), 128.42 (C2-C_6_H_4_), 127.50 (C14-C_6_H_4_N), 127.31 (C5-C_9_H_6_N), 126.38 (C4-C_6_H_4_N), 125.12 (C13-C_9_H_6_N), 125.09 (C19-C_6_H_4_), 124.41 (C12-C_6_H_4_), 124.38 (C20-C_6_H_4_), 122.16 (C8-C_9_H_6_N), 121.83 (C15-C_6_H_4_), 116.41 (C18-C_6_H_4_), 116.25 (C21-C_6_H_4_). FT-IR (cm^−1^): (Ar-CH) 3070, (-C=N-) 1624, (pyridyl) 1585, (C-F) 1490. UV/Vis (CH_3_CN): *λ*_max_ 234, 276, 327 nm. MS: *m/z* Calcd. [C_22_H_16_AgF_2_N_3_]: 468; found [Ag(**L1**) + K]^+^: 507 (57%), 508 (54%), [Ag(**L1**)-F]^+^: 463 (37%). Anal. Calcd. (%) for [C_22_H_17_AgClF_2_N_3_O_4_]: C, 46.46; H, 3.01; N, 7.39; found (%): C, 46.32; H, 2.89; N, 7.14.

#### 3.4.7. [Ag(**L2**)_2_]ClO_4_ **Q7**

Light brown solid, 0.46 g, 63%, Melting point; 242–243 °C, ^1^H-NMR (400 MHz, (DMSO-*d6*, δ ppm): 8.62 (2H, d, *J* = 8.54 Hz, Hg-C_6_H_4_-), 8.47 (2H, d, *J* = 8.54 Hz, He-C_9_H_6_N-), 8.17 (8H, Hj-C_6_H_4_, Hd-C_9_H_6_N, Ha-C_9_H_6_N, Hf-C_9_H_6_N), 7.89 (2H, t, *J* = 7.67 Hz, Hb-C_9_H_6_N), 7.73 (2H, d, *J* = 7.50 Hz, Hc-C_9_H_6_N), 7.57 (4H, m, Hh-C_6_H_4_, Hi-C_6_H_4_). ^13^C-NMR (400 MHz, (DMSO-*d6*, 25 °C): δ = 169.27 (C9-C_9_H_6_N), 153.93 (C11-C_6_H_4_), 150.73 (C10-C=N-), 147.37 (C1-C_9_H_6_N), 138.33 (C7-C_9_H_6_N), 135.94 (C16-C_6_H_4_), 131.14 (C3-C_9_H_6_N), 129.25 (C2-C_9_H_6_N), 129.08 (C5-C_9_H_6_N), 128.54 (C4-C_9_H_6_N), 128.34 (C6-C_9_H_6_N), 127.06 (C13-C_6_H_4_), 126.66 (C14-C_6_H_4_), 123.85 (C15-C_6_H_4_), 122.95 (C12-C_6_H_4_), 118.41 (C8-C_9_H_6_N). FT-IR (cm^−1^): (Ar-CH) 3062, (C-S-C) 751, (quinolinyl) 1586. UV/Vis (CH_3_CN): *λ*_max_ 217, 289, 318, 335, 350 nm. MS: *m/z* Calcd. For [C_32_H_20_AgN_4_S_2_]: 632,53; found [Ag (**L2**)_2_ + H)]: 633 (100%), 631 (81%), 634 (30%), [Ag(**L2**) + H + CH_3_CN)]^+^: 412 (38%), 410 (35%). Anal. Calcd. (%) for [C_48_H_30_Ag_2_ClN_6_O_4_S_3_]: C, 52.31; H, 2.74; N, 7.63; found (%): C, 52.16; H, 2.64; N, 7.60.

#### 3.4.8. [Ag(**L3**)_2_]ClO_4_ **Q8**

Yellow solid, 0.66 g, 89%, Melting point; 190–191 °C. ^1^H-NMR (400 MHz, (CD_3_)_2_CO, δ ppm): 9.19 (2H, s, Hg-CH=N-), 8.80 (2H, d, *J* = 8.30 Hz, He-C_9_H_6_N), 8.25 (4H, q, *J* = 8.48 Hz, *J* = 8.41 Hz, Hd, f-C_9_H_6_N), 8.18 (2H, d, *J* = 7.27 Hz, Ha-C_9_H_6_N), 7.94 (2H, m, Hb-C_9_H_6_N), 7.80 (2H, m, Hc-C_9_H_6_N), 7.67 (4H, m, Hi-C_6_H_4_), 7.60 (4H, m, Hh-C_6_H_4_). ^13^C-NMR (400 MHz, (CD_3_)_2_CO, 25 °C): δ = 151.31 (C10-C=N-), 147.64 (C1-C_6_H_4_), 146.62 (C11-C_9_H_6_N), 139.73 (C9-C_9_H_6_N), 132.16 (C7-C_9_H_6_N), 131.40 (C14-C_9_H_6_N), 130.25 (C6-C_6_H_4_), 130.18 (C13 & C15-C_9_H_6_N), 130.01 (C3-C_6_H_4_), 128.96 (C2-C_6_H_4_), 128.88 (C5 & C8-C_6_H_4_), 124.69 (C4-C_6_H_4_), 124.13 (C12 & C16-C_9_H_6_N). FT-IR (cm^−1^): (Ar-CH) 3065, (quinolinyl) 1592. UV/Vis (CH_3_CN): *λ*_max_ 242, 320 nm. MS: *m/z* Calcd. for [C_32_H_22_AgCl_2_N_4_]: 641.32; found [Ag(**L3**)_2_]^+^: 641 (81%), 639 (41%), 643 (39%), [Ag(**L3**) + H + CH_3_CN]^+^: 416 (56%), 414 (44%), 418 (15%). Anal. Calcd. (%) for [C_32_H_22_AgCl_3_N_4_O_4_]: C, 51.89; H, 2.99; N, 7.56; found (%): C, 51.69; H, 2.85; N, 7.31.

#### 3.4.9. [Ag(**L4**)_2_]ClO_4_ **Q9**

Brown solid, 0.55 g, 80%, Melting point: 149–150 °C. ^1^H-NMR (400 MHz, DMSO-*d6*, δ ppm): 9.20 (2H, s, Hg-CH=N-), 8.76 (2H, d, *J* = 8.37 Hz, He-C_9_H_6_N), 8.27 (2H, d, *J* = 8.50 Hz, Hf-C_9_H_6_N), 8.15 (4H, m, Hd, a-C_9_H_6_N), 7.87 (2H, m, Hb-C_9_H_6_N), 7.76 (2H, m, Hc-C_9_H_6_N), 7.52 (4H, m, Hi-C_6_H_4_), 7.29 (4H, d, *J* = 7.95 Hz, Hh-C_6_H_4_), 2.34 (6H, m, Hj-CH_3_). ^13^C-NMR (400 MHz, DMSO-*d6*, 25 °C): δ = 159.03 (C10-C_9_H_6_N), 151.62 (C1-C_6_H_4_), 146.25 (C11-C_9_H_6_N), 145.71 (C9-C_9_H_6_N), 138.89 (C15-C_9_H_6_N), 138.16 (C7-C=N-), 131.43 (C6-C_6_H_4_), 130.02 (C13 & C16-C_9_H_6_N), 129.37 (C3-C_6_H_4_), 129.18 (C2-C_6_H_4_), 128.58 (C5-C_6_H_4_), 128.33 (C4-C_6_H_4_), 122.79 (C12 & C17-C_9_H_6_N), 122.26 (C8-C_9_H_6_N), 20.64 (C14-C_9_H_6_N). FT-IR (cm^−1^): (Ar-CH) 3043, (-C=N-) 1682, (quinolinyl) 1587. UV/Vis (CH_3_CN): *λ*_max_ 242, 301, 339 nm. MS: *m/z* Calcd. For [C_34_H_28_AgN_4_]: 600.49; found [Ag (**L4**)_2_ + H)]: 601 (100%), 599 (90%), 602 (29%), 600 (19%) [Ag(**L4**) + H + CH_3_CN)]^+^: 396 (26%), 394 (21%). Anal. Calcd. (%) for [C_34_H_28_AgClN_4_O_4_]: C, 58.34; H, 4.03; N, 8.00; found (%): C, 58.11; H, 3.98; N, 7.92.

#### 3.4.10. [Ag(**L5**)_2_]ClO_4_ **Q10**

Orange solid, 0.71 g, 100%, Melting point; 132–133 °C. ^1^H-NMR (400 MHz, (CD_3_)_2_CO, δ ppm): 9.02 (2H, s, Hg-CH=N-), 8.76 (2H, d, *J* = 8.41 Hz, He-C_9_H_6_N), 8.12 (4H, d, *J* = 8.48 Hz, Hf, d-C_9_H_6_N), 7.71 (6H, m, Ha, b, c-C_9_H_6_N), 7.23 (2H, m, Hk-C_4_H_3_S), 6.98 (2H, d, *J* = 3.43 Hz, Hi-C_4_H_3_S), 6.77 (2H, m, Hj-C_4_H_3_S), 5.13 (4H, s, Hh-CH_2_). ^13^C-NMR (400 MHz, DMSO-*d6*, 25 °C): δ = 162.47 (C10-C=N-), 150.07 (C9-C_9_H_6_N), 146.13 (C1-C_9_H_6_N), 140.31 (C15-C_4_H_3_S), 136.65 (C7-C_9_H_6_N-), 132.09 (C6-C_9_H_6_N), 130.19 (C3-C_9_H_6_N), 129.24 (C2-C_9_H_6_N), 128.70 (C5-C_9_H_6_N), 127.92 (C4-C_9_H_6_N), 127.47 (C13-C_4_H_3_S), 127.35 (C12-C_4_H_3_S), 126.26 (C14-C_4_H_3_S), 124.00 (C8-C_9_H_6_N), 57.70 (C11-CH_2_). FT-IR (cm^−1^): (Ar-CH) 3063, (-C=N-) 1644, (quinolinyl) 1589. UV/Vis (CH_3_CN): *λ*_max_ 287, 320sh, 334sh nm. MS: *m/z* Calcd. for [C_30_H_24_AgN_4_S_2_]: 612.54; found [Ag(**L5**)_2_ + H]^+^: 613 (100%), 611 (79%), 614 (30%), 612 (15%), 615 (11%). Anal. Calcd. (%) for [C_30_H_24_AgClN_4_O_4_S_2_]: C, 50.61; H, 3.40; N, 7.87; found (%): C, 50.32; H, 3.40; N, 7.83.

#### 3.4.11. [Ag(**L1**)_2_]CF_3_SO_3_ **Q11**

Brown solid, 0.60 g, 97%, Melting point; 154–155 °C, ^1^H-NMR (400 MHz, DMSO-*d6*, δ ppm): 9.17 (1H, s, Hg-CH=N-), 8.78 (1H, d, *J* = 8.36 Hz, He-C_9_H_6_N), 8.30 (1H, d, *J* = 8.45 Hz, Hf-C_9_H_6_N), 8.17 (2H, m, Ha, d-C_9_H_6_N), 7.88 (1H, m, Hb-C_9_H_6_N), 7.77 (1H, m, Hc-C_6_H_4_), 7.62 (1H, m, Hj-C_9_H_6_N), 7.34 (3H, m, Hk, i, h-C_6_H_4_), 6.95 (1H, m, Ho-C_6_H_4_), 6.86 (1H, m, Hm-C_6_H_4_), 6.76 (1H, m, Hn-C_6_H_4_), 6.50 (1H, m, Hl-C_6_H_4_). ^13^C-NMR (400 MHz, DMSO-*d6*, δ ppm 25 °C): δ = 162.83 (C10-C=N-), 156.48 (C22-C_6_H_4_), 154.00 (C16-C_6_H_4_), 151.17 (C1-C_9_H_6_N), 146.26 (C9-C_9_H_6_N), 139.02 (C7-C_9_H_6_N), 137.11 (C11-C_6_H_4_), 137.02 (C17-C_6_H_4_), 131.51 (C6-C_6_H_4_), 129.48 (C3-C_6_H_4_), 129.33 (C2-C_6_H_4_), 129.25 (C14-C_6_H_4_N), 128.84 (C5-C_9_H_6_N), 128.34 (C4-C_6_H_4_N), 125.24 (C13-C_9_H_6_N), 125.21 (C19-C_6_H_4_), 124.42 (C12-C_6_H_4_), 124.39 (C20-C_6_H_4_), 122.81 (C8-C_9_H_6_N), 122.11 (C15-C_6_H_4_), 116.57 (C18-C_6_H_4_), 116.37 (C21-C_6_H_4_). FT-IR (cm^−1^): (Ar-CH) 3061, (-C=N-) 1624, (C=N quinolinyl) 1585. UV/Vis (CH_3_CN): *λ*_max_ 234, 276, 327 nm. MS: *m/z* Calcd. for [C_22_H_16_AgF_2_N_3_]: 468; found [Ag(**L1**) + CH_3_CN]^+^: 509 (98%), 507 (87%). Anal. Calcd. (%) for [C_23_H_17_AgF_5_N_3_O_3_S]: C, 44.68; H, 2.77; N, 6.80; found (%): C, 44.62; H, 2.51; N, 6.73.

#### 3.4.12. [Ag(**L2**)_2_]CF_3_SO_3_ **Q12**

Yellow solid, 0.68 g, 87%, Melting point; 245–246 °C. ^1^H-NMR (400 MHz, (CD_3_)_2_CO, δ ppm): 8.62 (2H, d, *J* = 8.11 Hz, Hg-C_6_H_4_-), 8.48 (2H, d, *J* = 8.54 Hz, He-C_9_H_6_N-), 8.16 (8H, Hj-C_6_H_4_, Hd-C_9_H_6_N, Ha-C_9_H_6_N, Hf-C_9_H_6_N), 7.89 (2H, m, Hb-C_9_H_6_N), 7.72 (2H, m, Hc-C_9_H_6_N), 7.58 (4H, m, Hh-C_6_H_4_, Hi-C_6_H_4_). ^13^C-NMR (400 MHz, (CD_3_)_2_CO, 25 °C): δ = 150.84 (C9-C_9_H_6_N), 138.27 (C11-C_6_H_4_), 135.97 (C10-C=N-), 131.10 (C1-C_9_H_6_N), 129.22 (C7-C_9_H_6_N), 129.06 (C16-C_6_H_4_), 128.54 (C3-C_9_H_6_N), 128.32 (C2 & C5-C_9_H_6_N), 127.03 (C4-C_9_H_6_N), 126.63 (C6 & C13-C_9_H_6_N), 123.85 (C14-C_6_H_4_), 122.94 (C12 & C15-C_6_H_4_), 118.30 (C8-C_9_H_6_N). FT-IR (cm^−1^): (Ar-CH) 3059, (C-S-C) 754, (quinolinyl) 1588. UV/Vis (CH_3_CN): *λ*_max_ 217, 285, 320, 335, 350 nm. MS: *m/z* Calcd. For [C_32_H_20_AgN_4_S_2_]: 632,53; found [Ag (**L2**)_2_ + H)]^+^: 633 (100%), 631 (81%), 634 (30%), [Ag(**L2**) + H + CH_3_CN)]^+^: 412 (38%), 410 (35%). Anal. Calcd. (%) for [C_34_H_20_Ag_2_F_6_N_4_O_6_S_4_]: C, 39.32; H, 1.94; N, 5.39; found (%): C, 39.15; H, 1.78; N, 5.27.

#### 3.4.13. [Ag(**L3**)_2_]CF_3_SO_3_ **Q13**

Brown solid, 0.40 g, 50%, Melting point; 193–194 °C. ^1^H-NMR (400 MHz, (CD_3_)_2_CO, δ ppm): 9.16 (2H, s, Hg-CH=N-), 8.77 (2H, d, *J* = 8.30 Hz, He-C_9_H_6_N), 8.24 (4H, q, *J* = 8.48 Hz, *J* = 8.41 Hz, Hd, f-C_9_H_6_N), 8.17 (2H, d, *J* = 7.27 Hz, Ha-C_9_H_6_N), 7.92 (2H, m, Hb-C_9_H_6_N), 7.78 (2H, m, Hc-C_9_H_6_N), 7.63 (4H, m, Hi-C_6_H_4_), 7.58 (4H, m, Hh-C_6_H_4_). ^13^C-NMR (400 MHz, (CD_3_)_2_CO, 25 °C): δ = 151.20 (C10-C=N-), 147.32 (C1-C_6_H_4_), 146.24 (C11-C_9_H_6_N), 138.99 (C9-C_9_H_6_N), 132.44 (C7-C_9_H_6_N), 131.50 (C14-C_9_H_6_N), 129.59 (C6-C_6_H_4_), 129.58 (C13 & C15-C_9_H_6_N), 129.47 (C3-C_6_H_4_), 128.79 (C2-C_6_H_4_), 128.34 (C5-C_6_H_4_), 124.05 (C4-C_6_H_4_), 123.00 (C12 & C16-C_9_H_6_N), 121.94 (C8-C_9_H_6_N). UV/Vis (CH_3_CN): *λ*_max_ 244, 307, 335 nm. MS: *m/z* Calcd. for [C_32_H_22_AgCl_2_N_4_]: 641.32; found [Ag(**L3**)_2_]^+^: 641 (100%), 639 (65%), 643 (52%), 642 (25%), [Ag(**L3**) + H + CH_3_CN ]^+^: 416 (37%), 414 (29%). Anal. Calcd. (%) for [C_33_H_22_AgCl_2_F_3_N_4_O_3_S]: C, 50.15; H, 2.81; N, 7.09; found (%): C, 50.15; H, 2.70; N, 6.98. FT-IR (cm^−1^): (Ar-CH) 3063, (quinolinyl) 1591.

#### 3.4.14. [Ag(**L4**)_2_]CF_3_SO_3_ **Q14**

Brown solid, 0.60 g, 80%, Melting point: 152–153 °C. ^1^H-NMR (400 MHz, DMSO-*d6*, δ ppm): 9.04 (2H, s, Hg-CH=N-), 8.66 (2H, d, *J* = 8.58 Hz, He-C_9_H_6_N), 8.28 (2H, d, *J* = 8.50 Hz, Hf-C_9_H_6_N), 8.14 (4H, m, Hd, a-C_9_H_6_N), 7.89 (2H, m, Hb-C_9_H_6_N), 7.75 (2H, m, Hc-C_9_H_6_N), 7.47 (4H, m, Hi-C_6_H_4_), 7.31 (4H, d, *J* = 7.92 Hz, Hh-C_6_H_4_), 2.36 (6H, m, Hi-CH_3_). ^13^C-NMR (400 MHz, DMSO-*d6*, 25 °C): δ = 159.01 (C10-C_9_H_6_N), 151.62 (C1-C_6_H_4_), 146.21 (C11-C_9_H_6_N), 145.66 (C9-C_9_H_6_N), 138.83 (C15-C_9_H_6_N), 138.11 (C7-C=N-), 129.97 (C6-C_6_H_4_), 129.43 (C13 & C16-C_9_H_6_N), 129.30 (C3-C_6_H_4_), 129.14 (C2-C_6_H_4_), 128.53 (C5-C_6_H_4_), 128.28 (C4-C_6_H_4_), 122.70 (C12 & C17-C_9_H_6_N), 122.20 (C8-C_9_H_6_N), 20.59 (C14-C_9_H_6_N). UV/Vis (CH_3_CN): *λ*_max_ 245, 299, 340 nm. FT-IR (cm^−1^): (Ar-CH) 3067, (-C=N-) 1626, (quinolinyl) 1584. MS: *m/z* Calcd. For [C_34_H_28_AgN_4_]: 600.49; found [Ag (**L4**)_2_ + H)]^+^: 601 (100%), 599 (99%), 602 (34%), 600 (22%), 603 (6%), [Ag(**L4**) + H + CH_3_CN)]^+^: 396 (38%), 394 (34%). Anal. Calcd. (%) for [C_35_H_28_AgF_3_N_4_O_3_S]: C, 56.08; H, 3.77; N, 7.47; found (%): C, 55.96; H, 3.67; N, 7.23.

#### 3.4.15. [Ag(**L5**)_2_]CF_3_SO_3_ **Q15**

Brown solid, 0.66 g, 87%, Melting point; 148–149 °C. ^1^H-NMR (400 MHz, DMSO-*d6*, δ ppm): 8.84 (2H, s, Hg-CH=N-), 8.62 (2H, d, *J* = 8.62 Hz, He-C_9_H_6_N), 8.10 (4H, t, *J* = 7.22 Hz, Hf, d-C_9_H_6_N), 7.99 (2H, d, *J* = 8.29 Hz, Ha-C_9_H_6_N), 7.82 (2H, m, Hb-C_9_H_6_N), 7.71 (2H, t, *J* = 7.49 Hz, Hc-C_9_H_6_N), 7.42 (2H, m, Hk-C_4_H_3_S), 7.09 (2H, m, Hi-C_4_H_3_S), 6.97 (2H, m, Hj-C_4_H_3_S), 5.15 (2H, s, Hl-CH_2_). ^13^C-NMR (400 MHz, DMSO-*d6*, 25 °C): δ = 162.40 (C10-C=N-), 150.00 (C9-C_9_H_6_N), 146.06 (C1-C_9_H_6_N), 140.24 (C15-C_4_H_3_S), 136.58 (C7-C_9_H_6_N-), 132.03 (C6-C_9_H_6_N), 130.12 (C3-C_9_H_6_N), 129.47 (C2-C_9_H_6_N), 128.63 (C5-C_9_H_6_N), 127.85 (C4-C_9_H_6_N), 127.40 (C13-C_4_H_3_S), 127.28 (C12-C_4_H_3_S), 126.19 (C14-C_4_H_3_S), 123.93 (C8-C_9_H_6_N), 57.63 (C11-CH_2_). FT-IR (cm^−1^): (Ar-CH) 3068, (-C=N-) 1646, (C=N quinolinyl) 1591. UV/Vis (CH_3_CN): *λ*_max_ 291, 316 sh, 330 sh nm. MS: *m/z* Calcd. for [C_30_H_24_AgN_4_S_2_]^+^: 612.54; found [Ag(**L5**)_2_ + H]^+^: 613 (100%), 611 (85%), 614 (29%), 612 (18%), 615 (10%), [Ag(**L5**) + CH_3_CN]^+^: 400 (30%), 402 (29%). Anal. Calcd. (%) for [C_31_H_24_AgF_3_N_4_O_3_S_3_]: C, 48.89; H, 3.18; N, 7.36; found (%): C, 48.65; H, 3.11; N, 7.24.

### 3.5. In Vitro Antimicrobial Studies

#### 3.5.1. Müeller–Hinton Agar Test Plates Preparation

Sterilized Nutrient agar medium was first prepared by dissolving 38 g of Müeller–Hinton agar (MHA) (Biolab, Midrand, Africa) in distilled water (1 L). The resulting Nutrient agar medium was subjected to sterilization by autoclaving for 15 min at 121 °C and then cooled down to 45 °C in a water bath. The cooled Agar medium was poured in Petri dishes while ensuring a uniform 4 mm depth of the medium and cooling further to ambient temperature [25].

#### 3.5.2. Inoculation Procedure

Complexes **Q1**–**Q15** and their ligands were tested against four Gram-negative bacteria: *Salmonella typhimurium* ATCC 14026, *Pseudomonas aeruginosa* ATCC 27853, *Escherichia coli* ATCC 25922, and *Klebsiella pneumoniae* ATCC 31488; and two Gram-positive bacteria, viz., *Staphylococcus aureus* ATCC 700699 (methicillin-resistant) and *Staphylococcus aureus* ATCC 25923. These bacteria were inoculated in a sterilized Nutrient Broth (NB) (Biolab, Midrand, Africa) through streak plate technique and incubated at 37 °C for 18 h. The nutrient broth was sterilized by dissolving 1.3 g in distilled water (100 mL). Ca. 10 mL of the nutrient broth was transferred into a cotton wool-plugged test tubes and wrapped with an aluminium foil. The nutrient broth in the test tubes was then autoclaved for 15 min at 121 °C and cooled to 37 °C. Following this was the isolation of a single colony of the bacteria and inoculated into a 10 mL sterile NB which was then incubated in a shaking incubator at 37 °C for 18 h. Each of the bacteria strain concentrations was adjusted with sterile distilled water to get a final concentration of 1.5 × 10^8^ cfu/mL, i.e., 0.5 Mc Farland’s Standard using a densitometer (Mc Farland Latvia) [62]. The bacteria were lawn inoculated on the set MHA Petri dishes using a sterile cotton swab. Screening of **L1**–**L5** and **Q1**–**Q15** for antibacterial activity was first carried out by spotting 5 µL of their solution (prepared from the dissolution of 1000 µg of the **L1**–**L5** and **Q1**–**Q15** in 1 mL dimethyl sulfoxide (DMSO)) on an MHA petri dish and incubated at 37 °C for 18 h. The antibacterial activity was determined by looking out for a clear zone at the spotting point. The compounds with potential antimicrobial activity were then tested for their minimum inhibitory concentration (MIC) against six bacterial. Ten serial dilution of **L1**–**L5** and **Q1**–**Q15** was done to obtain 1000 µg/mL to 0.2 µg/mL concentrations, where the compounds showed lower MICs than 0.2 µg/mL, the solution was further diluted 5 times to obtain 0.100 µg/mL to 0.00625 µg/mL concentrations. Evaluation of the compounds MIC was determined by spotting 5 µL of each concentration of **L1**–**L5** and **Q1**–**Q15** on the MHA plates, and the plates were incubated at 37 °C for 18 h. These procedures were done in triplicate to give the accurate lowest concentration of the compounds where no visible bacterial growth was seen after incubation. Ciprofloxacin was used as a standard and DMSO used as a negative control showed no bactericidal effect against all the bacteria strains [50,62,103].

### 3.6. Antioxidant Assay

Antioxidant activity studies of **L1**–**L5** and **Q1**–**Q15** were done using ferric reducing antioxidant power (FRAP) assay. The ferric reducing antioxidant power assay is often used to evaluate the ability of an antioxidant to donate an electron. The reducing ability of the compounds was measured using a previously published method [104] after slight modifications where different concentrations (0.25 mg/mL, 0.5 mg/mL, 1 mg/mL, and 2 mg/mL) of **L1**–**L5** and **Q1**–**Q15** and standard (Ascorbic acid) were mixed with 2.5 mL of phosphate buffer (0.2 mol/L, pH 6.6) and 2.5 mL of 1% *w*/*v* potassium ferricyanide respectively. The resulting mixture was incubated at 50 °C for 20 min, and 2.5 mL of 10% trichloroacetic acid was added to acidify the mixture. After that, 1 mL of the acidified mixture was added to 1 mL of distilled water and 0.5 mL of 0.1% FeCl_3_. The absorbance of the resulting solution was then measured at 700 nm. The antioxidant power of the compounds was expressed as a percentage of ferric reducing antioxidant power Ascorbic acid equivalent, as shown in Equation (4).

(4) $$\%\;FRAP\;=Absorbance\;of\;\frac{Absorbance\;of\;Sample}{Absorbance\;of\;Ascorbic\;Acid}\;\times 100\;$$

### 3.7. DNA Binding Experiments

The interaction of the **L1**–**L5** and complexes **Q1**–**Q15** with calf thymus-DNA was carried out in a Phosphate buffer saline solution pH 7.2 stored at 4 °C. The DNA stock solution was prepared by dissolving CT-DNA sodium salt in Phosphate buffer saline solution with continuous stirring overnight. It was filtered and stored at 4 °C and used within four days. The final concentration of CT-DNA sodium salt was determined by UV-visible absorption using the extinction coefficient ε260 = 6600 M^−1^ cm^−1^ [105]. The ratio of CT-DNA at 260 and 280 nm UV absorbance was 1.8–1.93:1, indicating that it is pure and sufficiently free of protein contamination. A fixed amount of the **L1**–**L5** and complexes **Q1**–**Q15** (50 µM) were prepared by dissolving an appropriate amount in DMSO. At a low DMSO concentration, the stability of biomolecules is known to be retained [106,107]. Therefore, in all the experimental of the interaction of **L1**–**L5** and complexes **Q1**–**Q15** with biomolecules, the final concentration of DMSO in the incubation mixture was ~2% (*v*/*v*). Thus, the effect of DMSO on the stability of the biomolecules are negligible.

#### 3.7.1. DNA Absorption Spectral Study

The binding modes of **L1**–**L5** and complexes **Q1**–**Q15** to CT-DNA were characterized through electronic absorption titration by varying concentrations of CT-DNA (0–30 µM) in phosphate buffer saline solution against fixed concentrations (20 µM) of **L1**–**L5** and complexes **Q1**–**Q15**. The compounds-CT-DNA mixture was incubated for 10 min before measuring the absorbance using UV-vis absorption spectroscopy [108]. The absorbance of CT-DNA is cancelled by adding equivalent amounts of CT-DNA to both the tested compounds and the reference solutions.

The compounds intrinsic binding constant, *K_b_* were determined using Wolfe–Shimer equation as follows:

(5) $$\frac{\left[\mathrm{DNA}\right]}{\varepsilon_{a}-\varepsilon_{f}}=\frac{\left[\mathrm{DNA}\right]}{\varepsilon_{b}-\varepsilon_{f}}=\frac{1}{\varepsilon_{b}-\varepsilon_{f}}$$

where [DNA], *ε*_a_, *ε*_b_, and *ε*_f_ are DNA concentration, apparent, fully bound complex, and free complex extinction coefficients, respectively. The ratio of the slope to the intercept from the plot of [DNA]/(ε_a_ − ε_f_) against [DNA] gave the binding constant *K_b_* of the compounds.

#### 3.7.2. Luminescence Competitive Displacement Study

Ethidium bromide (EB) is one of the most sensitive fluorescent probes which can bind to DNA through intercalation. Competitive binding of complexes **Q1**–**Q15** to DNA with EB could provide information with regards to the DNA-binding affinity.

A competitive displacement study was carried out using fluorescence spectroscopy between CT-DNA pre-treated with ethidium bromide (EB) and complexes **Q1**–**Q15** to establish the complexes′ actual mode of binding to CT-DNA. Ethidium bromide in buffer solution has low fluorescence intensity due to fluorescence quenching of the free ethidium bromide by the solvent molecule, but on binding through intercalation to DNA, its emission intensity is drastically enhanced [109]. The competitive luminescence displacement assay was done using a modified method [91]. Pre-treated 15 µM solution of EB with 15 µM of CT-DNA was prepared in a phosphate buffer of pH 7.2 and left to equilibrate for 30 min. Keeping CT-DNA-EB concentrations constant, different concentrations (0–20 µM) of the complexes were added at 8 min incubation interval at room temperature. The fluorescence quenching ability of CT-DNA bound EB by the complexes were recorded in the wavelength range of 530–700 nm with an excitation wavelength at 525 nm at 25 °C.

(6) $$\frac{F_{o}}{F}=1+K_{SV}\left[Q\right]=1+K_{q}\tau_{o}\left[Q\right]$$

A Stern–Volmer (Equation (6)) was used to evaluate the CT-DNA-EB fluorescence quenching of the complexes. Where *F*_o_ and *F* denote the relative fluorescence intensities of CT-DNA in the absence and presence of the quencher, *K_sv_*, Stern–Volmer quenching constant, [*Q*], quencher concentration, K*q*, bimolecular quenching constant and *τ*_o_, average lifetime of the fluorophore in the absence of the quencher which is typically equal to 10^−8^ s in biomacromolecules.

#### 3.7.3. Albumin Binding Assay Using Absorption Spectroscopy Titration

The absorbance assay of albumin binding is a very simple method used in identifying the conformational changes in protein. The studies aimed at the binding of bioactive compounds with protein to provide useful information on their biodistribution, toxicity, and mechanism of action [18]. A stock solution of bovine serum albumin (BSA) was prepared by dissolving an appropriate amount of BSA in phosphate buffer saline solution (pH 7.2) under constant stirring for 1 h at 25 °C. It was kept at 4 °C and used within four days. The BSA concentration was determined spectrophotometrically by using ε280 = 44300 M^−1^ cm^−1^ absorption coefficient. The stock solution of the complex was prepared by dissolving 1 mmol of the complexes in DMSO. The absorption titration assay was done by adding different concentrations (0–10 µM) of the complexes to a constant BSA concentration (6 µM). The sample solution was incubated after each addition of the concentrations for 10 min at 25 °C before recording the absorbance at λmax 280 nm. Binding constant *K_b_* was calculated from the intercept to the slope ratio of 1/[*A*-*A*°] vs. 1/[complex] linear curve where A and A° represent BSA absorbance in the presence and absence of complexes, respectively [86].

#### 3.7.4. Albumin Binding Studies Using Fluorescence Quenching Method

A solution of 1 µM of BSA was titrated against various concentrations of the complexes (0–35 µM) and incubated for 8 min at 25 °C. BSA was excited at 280 nm and emitted at 346 nm wavelength [92]. *K_sv_* constant was determined from the Stern–Volmer equation:

(7) $$I_{o}/I=1+Kq\;\tau_{o}\;\left[Q\right]=1+Ksv\left[Q\right]$$

where *I*_o_ and *I* represent the intensities of the fluorescence in the presence and absence of the quencher respectively while *K_q_*, *K_sv_*, *τ*_o_, [*Q*] represent the BSA quenching rate constant, dynamic quenching constant, the average lifetime of BSA without the quencher (*τ*_o_ = 10^−8^ s) and concentration of quencher, respectively.

### 3.8. Cytotoxicity Evaluation

In vitro cell viability with cultured cells is a method commonly used for cytotoxicity activities of potential anticancer compounds due to reduced cost speed, and potential for automation, and tests using human cells other than some in vivo animal tests [110]. The significant effect the perchlorate anion has on the complexes’ biological activities, as seen in the antimicrobial, antioxidant, DNA and protein binding assays prompted the choice of complexes to be assayed for cytotoxicity activity. Therefore, the cytotoxicity of some selected complexes; **Q6**, **Q7**, **Q9**, and cisplatin as reference standard was evaluated on four human cell lines, human embryonic kidney 293 (HEK293), cervical cancer cell line (HELA), breast cancer cell line (MDA-MB231), and neuronal cancer cell line (SHSY5Y).

The cytotoxicity assay was conducted as per the standard method and as described by Abrahams et al. 2018 [111]. Briefly, 96-well microtiter plates were seeded with HEK293 (non -cancerous cell line), HELA, MDA-MB231, and SHSY5Y at a concentration of 1 × 10^5^ cells/mL and allowed to stabilize for 4 h at 37 °C and 5% CO_2_. Thereafter, **Q6**, **Q7**, **Q9**, and cisplatin were added to the plate through twofold serial dilution to allow for eight final compound concentrations ranging from 100 to 0.781 µM in a total volume of 200 µL / well. The plate was then incubated for 72 h at 37 °C and 5% CO_2_. To each well, 20 µL CellTiter 96 AQ_ueous_ One Solution (Promega, Madison, WI, USA) was added and the plates were incubated for 4 h as previously described. Thereafter, absorbance was read at 490 nm on a multiplate reader (Molecular Devices, San Jose, CA, USA). EC_50_ values were determined as the concentration of each compound required to reduce cell viability by 50% and were calculated using GraphPad Prism 8 (2019) (GraphPad Software, San Diego, CA, USA). The values recorded are averages of at least three separate experiments (*n* = 3).

## 4. Conclusions

Herein, we have described the synthesis and characterization of silver(I) complexes of five quinolinyl imine. The influence of the quinolinyl imine variable substituents and silver(I) anion on the interaction of the compounds with Ct-DNA, protein, and their antimicrobial, antioxidant, and cytotoxicity activities were evaluated. The antimicrobial evaluation data revealed that all the complexes had moderate to excellent antibacterial activity and complexes [Ag(**L2**)_2_]ClO_4_ **Q7** and [Ag(**L3**)_2_]ClO_4_ **Q8** showed remarkable antimicrobial activity compare to the standard ciprofloxacin against all the bacteria tested. The ligands of each of [Ag(**L2**)_2_]ClO_4_ **Q7** and [Ag(**L3**)_2_]ClO_4_ **Q8** possess either the benzothiazole moiety or the *p*-chloro substituent, and both complexes have perchlorate as the anion, which could have enhanced their antimicrobial activity. Similarly, complexes with notable antimicrobial activity also displayed good antioxidant activity than the ascorbic acid (2.68 mg/mL) as seen in **Q2**, **Q4**, **Q5**, **Q6**, **Q7**, **Q9**, **Q10**, and **Q13** with IC_50_ between 0.95 and 2.22 mg/mL. In the absorption and emission spectral of the interaction of the compounds with CT-DNA and bovine serum albumin, the results indicate that the compounds bind to CT-DNA via intercalation mode due to the planarity of the chelating ligand and the competitive study with ethidium bromide shows that the complexes can displace EB from the CT-DNA-EB adduct and compete for the DNA-binding sites with EB, which is usually characteristic of the intercalative interaction of compounds with DNA.

Further study demonstrated that the complexes exhibit a moderate affinity for protein and the possible quenching mechanism between the complexes and bovine serum albumin is static. Thus, [Ag(**L1**)_2_]NO_3_ **Q1**, [Ag(**L1**)_2_]ClO_4_ **Q6** and [Ag(**L1**)_2_]CF_3_SO_3_ **Q11** can be stored in protein and can be easily released in desired target areas.

Complexes [Ag(**L2**)_2_]ClO_4_ **Q7** and [Ag(**L4**)_2_]ClO_4_ **Q9** with benzothiazole moiety and Methyl substituent respectively displayed a remarkable cytotoxicity activity especially towards Hela (cervical cancer cell line) ca. two times better than the cisplatin standard and five times than [Ag(**L1**)_2_]ClO_4_ **Q6** with fluorine substituent and a trigonal geometry.

Based on the remarkable biological activities recorded for silver(I) quinolinyl Schiff base derivatives in this study, Ag(I) quinolinyl Schiff bases tend to be promising candidates for the development of novel anticancer, antioxidant, and antibiotics drugs.

## Figures and Tables

**Table 1:** Selected geometric parameters for complexes **Q1**, **Q6**, **Q7**, **Q12**, and **Q14**.

	**Q1**	**Q6**	**Q7**	**Q12**	**Q14**
Ag–N_im_	2.398 (16)	2.400 (17)			2.317 (18)
Ag–N_qy_	2.240 (16)	2.246 (18)	2.37 (6)	2.346 (3)	2.321 (17)
Ag–N_am_	2.192 (16)	2.208 (17)			
Ag–N_th_			2.37 (2)	2.465 (3)	
Ag–O			2.66 (6)	2.587 (3) 2.397 (3)	2.609 (2) 2.233 (16)
Ag–Ag			3.06 (7)		
N–Ag–N	146.71 (6) 140.28 (6) 72.97 (6)	145.25 (6) 140.93 (6) 73.12 (6)	72.8 (4)	70.42 (10)	73.08 (6)
N–Ag–O			105.49 (2) 83.23 (2)	105.57 (10) 162.01 (10) 175.69 (9) 99.86 (10)	145.17 (6) 134.92 (6) 95.22 (6) 93.18 (6)
O–Ag–O				83.56 (9)	101.85 (7)

**Table 2:** Minimum inhibitory concentration of Silver complexes of **L1**–**L5** (µg/mL).

Compounds	Gram + Ve Bacteria			Gram − Ve Bacteria		
	SA	MRSA	PA	ST	EC	KP
**L1**	100	NA	NA	NA	1000	NA
**L2**	NA	NA	100	NA	NA	100
**L3**	NA	1000	100	NA	NA	50
**L4**	NA	NA	NA	1000	1000	NA
**L5**	NA	NA	100	50	NA	100
**Q1**	25	1000	25	100	50	0.2
**Q2**	25	50	12.5	50	25	0.05
**Q3**	50	50	12.5	50	25	0.05
**Q4**	12.5	50	12.5	50	12.5	0.8
**Q5**	50	100	6.25	12.5	1.6	0.025
**Q6**	3.125	25	1.6	25	12.5	1.6
**Q7**	1.6	50	0.2	50	25	0.4
**Q8**	50	25	0.1	25	1.6	0.0125
**Q9**	50	1000	6.25	50	12.5	0.05
**Q10**	25	1000	1.6	100	6.25	25
**Q11**	6.25	1000	50	25	50	0.2
**Q12**	6.25	50	12.5	50	25	0.1
**Q13**	25	50	12.5	50	25	0.1
**Q14**	50	1000	0.8	50	25	0.1
**Q15**	12.5	100	0.8	1000	25	0.05
AgNO_3_	3.125	0.2	12.5	1000	0.8	1000
AgClO_4_	3.125	6.25	0.8	0.2	25	1.6
AgCF_3_SO_3_	NA	1000	1000	12.5	50	1000
Ciprofloxacin ^s^ [54]	25	25	0.2	0.4	1.6	0.8

**Table 3:** In vitro antioxidant activities of **L1**–**L5** and Complexes **Q1**–**Q15** done in triplicate.

	Antioxidant (IC_50_ mg/mL)
COMPOUND	FRAP
**L1**	22.42 ± 0.0048
**L2**	2.29 ± 0.0041
**L3**	20.35 ± 0.0047
**L4**	4.50 ± 0.0053
**L5**	3.79 ± 0.0052
**Q1**	4.97 ± 0.002
**Q2**	1.10 ± 0.0044
**Q3**	4.75 ± 0.0042
**Q4**	2.14 ± 0.0035
**Q5**	2.04 ± 0.0032
**Q6**	0.95 ± 0.003
**Q7**	1.13 ± 0.044
**Q8**	3.27 ± 0.093
**Q9**	1.62 ± 0.078
**Q10**	0.98 ± 0.055
**Q11**	6.27 ± 0.038
**Q12**	1.86 ± 0.024
**Q13**	2.22 ± 0.0055
**Q14**	3.55 ± 0.022
**Q15**	3.35 ± 0.012
**AgNO_3_**	1.43 ± 0.03
**AgClO_4_**	0.88 ± 0.051
**AgCF_3_SO_3_**	0.88 ± 0.054
**Ascorbic Acid**	2.68 ± 0.11

**Table 4:** The binding parameters of complexes **Q1**–**Q15** interactions with CT-DNA, ethidium bromide, and bovine serum albumin.

Cmp.	CT-DNA-Cmp.		CT-DNA-EB-Complex				Protein-Complex				
	*K_b_* (M^−1^) × 10^5^	*R* ^2^	*K_sv_* (M^−1^) × 10^4^	*K*_q_ (M^−1^S^−1^) × 10^12^	*K*_bin_ (M^−1^) × 10^5^	*n*	*K_b_* (M^−1^) × 10^4^	*K_sv_* (M^−1^) × 10^4^	*K*_q_ (M^−1^S^−1^) × 10^12^	*K*_bin_ (M^−1^) × 10^4^	*n*
**Q1**	4.00	0.99	2.76	2.75	1.07	1.13	2.16	2.50	2.50	0.117	0.69
**Q2**	3.00	0.99	2.68	2.68	5.03	1.27	7.97				
**Q3**	15.7	0.99	3.93	3.93	9.17	1.29	5.11				
**Q4**	4.00	0.99	3.50	3.50	2.11	1.17	10.3				
**Q5**	4.00	0.99	2.31	2.31	1.03	1.15	5.95				
**Q6**	3.33	0.99	2.03	2.03	2.41	1.21	5.73	2.59	2.59	2.56	1.01
**Q7**	15.0	0.99	3.96	3.96	1.29	1.11	9.38				
**Q8**	30.0	0.99	3.98	3.98	1.02	1.09	9.48				
**Q9**	5.00	0.99	2.49	2.49	6.53	1.30	5.43				
**Q10**	5.00	0.99	2.98	2.98	9.61	1.31	8.01				
**Q11**	6.67	0.99	2.38	2.38	3.45	1.21	8.62	4.09	4.09	0.138	0.68
**Q12**	4.50	0.99	3.14	3.14	2.12	1.19	20.7				
**Q13**	4.29	0.99	3.33	3.33	5.74	1.27	2.89				
**Q14**	3.33	0.99	2.98	2.98	5.31	1.28	7.50				
**Q15**	4.00	0.99	2.48	2.48	5.29	1.30	13.1				
AgNO_3_	3.00	0.99	3.12	3.12	4.50	1.24	4.49				
AgClO_4_	3.30	0.99	3.31	3.31	6.00	1.26	4.82				
AgCF_3_SO_3_	2.00	0.99	3.01	3.01	1.60	1.15	4.46				
**L1**	1.38	0.96									
**L2**	1.50	0.98									
**L3**	7.50	0.97									
**L4**	1.60	0.99									
**L5**	1.17	0.98									

*R* is the correlation coefficient.

**Table 5:** EC_50_ and SI values of the cytotoxic activity of complexes **Q6**, **Q7**, **Q9**, and cisplatin against various human cell lines.

Compound	EC_50_ ± Std Dev (µM)				Selectivity Index (SI)		
	HEK293	HELA	MDA-MB231	SHSY5Y	HELA	MDA-MB231	SHSY5Y
**Cisplatin**	14.2 ± 3.8	>50	22.68 ± 4.89	19.74 ± 3.77	0.28	0.63	0.72
**Q6**	≥100	≥100	≥ 100	≥100	1.00	1.00	1.00
**Q7**	≥100	22.80 ± 3.11	41.62 ± 4.25	30.33 ± 4.67	4.39	2.40	3.30
**Q9**	≥100	22.34 ± 4.86	38.84 ± 4.33	47.23 ± 3.34	4.48	2.57	2.12

EC_50_ value calculated as the compound concentration required to reduce cell viability by 50%. Std Dev = Standard deviation. *n* = 3 for all samples. SI values: selectivity index; calculated as the quotient of the EC_50_ value obtained in HEK293 over the EC_50_ value obtained in the respective cancer cell lines.

## Supplementary Materials

The following are available online at https://www.mdpi.com/1420-3049/26/5/1205/s1, Figures S1–S22: Electronic absorption spectra of **L1**–**L5**, silver salts and **Q1**–**Q15** at 5.0 × 10^5^ M in the absence (dashed line) and the presence of difference concentrations of CT-DNA, Figures S23–S39: The fluorescence spectra of EB-CT-DNA in the absence (dashed line) and the presence of different concentration of silver salts and **Q1**–**Q15**, Figures S40–S56: The double-logarithmic plot of EB-CT-DNA–Complex **Q1**–**Q15** and silver salts interaction, Figures S57–S73: Electronic Absorption Spectra of BSA in the absence (dashed line) and the presence of different concentrations of **Q1**–**Q15** and silver salts, Figures S74 and S75: Fluorescence emission spectra of BSA in the absence(dashed line) and the presence of different concentration of complex **Q1** and **Q6**, Figures S76 and S77: The double-logarithmic plot of BSA–Complex **Q1** and **Q11** interactions, Figures S78–S97: ^1^H NMR spectra of **L1**–**L5** and **Q1**–**Q15**, Figures S98–S117: ^13^C NMR spectra of **L1**–**L5** and **Q1**–**Q15**, Figures S118–S137: IR spectra of **L1**–**L5** and **Q1**–**Q5**, Figure S138-S157: Mass Spec. spectra of **L1**–**L5** and **Q1**–**Q15**, Table S1: ^1^H-NMR chemical shifts of some protons in **L1**–**L5** complexes (**Q1**–**Q15**) and the IR band of (C=N) and quinolinyl N for **L1**–**L5** and complexes **Q1**–**Q15**, Table S2: Physical and chemical data of silver(I) complexes **Q1**–**Q15**, Table S3: Crystal data and structure refinement for complexes **Q1**, **Q6**, **Q7**, **Q12** and **Q14**.
