# Supplementary material for: Quinoline Functionalized Schiff Base Silver (I) Complexes: Interactions with Biomolecules and In Vitro Cytotoxicity, Antioxidant and Antimicrobial Activities
Source: Molecules. 2021 Feb 24;26(5):1205. doi: 10.3390/molecules26051205 (PMC7956476; doi:10.3390/molecules26051205)
Supplement: Supplementary file 1 [file molecules-26-01205-s001.zip › molecules-1074810-supplementary.docx]

**Quinoline functionalized Schiff base silver(I) complexes: Interactions with biomolecules and *in* *vitro* cytotoxicity, antioxidant and antimicrobial activities**

Adesola A. Adeleke^1,5^, Sizwe J. Zamisa^2^, Md. Shahidul Islam^3^, Kolawole Olofinsan^3^, Veronica F. Salau^3^, Chunderika Mocktar^4^ and Bernard Omondi*^1^

^1^ School of Chemistry and Physics, University of Kwazulu-Natal, Pietermaritzburg Campus, Private Bag X01, Scottsville 3209, South Africa; [217080311@stu.ukzn.ac.za](mailto:217080311@stu.ukzn.ac.za) (A.A.A.); [owaga@ukzn.ac.za](mailto:owaga@ukzn.ac.za) (B.O.)

^2^ School of Chemistry and Physics, University of Kwazulu-Natal, Westville Campus, Private Bag X54001, Westville 4001, South Africa; [zamisas@ukzn.ac.za](mailto:zamisas@ukzn.ac.za) (S.J.Z.)

^3^ Discipline of Biochemistry, School of Life Sciences, University of Kwazulu-Natal, Westville Campus, Private Bag X54001, Durban 4000, South Africa; [islamd@ukzn.ac.za](mailto:islamd@ukzn.ac.za) (M.S.I); [219017967@stu.ukzn.ac.za](mailto:219017967@stu.ukzn.ac.za) (K.O.); [218087036@stu.ukzn.ac.za](mailto:218087036@stu.ukzn.ac.za) (V.F.S)
^4^ Discipline of Pharmaceutical Sciences, School of Health Sciences, University of Kwazulu-Natal, Westville Campus, Private Bag X54001, Durban 4000, South Africa; [mocktarc@ukzn.ac.za](mailto:mocktarc@ukzn.ac.za) (C.M.)

^5^ Department of Chemical Sciences, Olabisi Onabanjo University, Ago-Iwoye, P. M. B. 2002, Nigeria

***Corresponding author.**

E-mail address: owaga@ukzn.ac.za http://orcid.org/0000-0002-3003-6712.

**Table of Contents**

[**Table S1:** ^1^H-NMR chemical shifts of some protons in **L1**–**L5** complexes (**Q1**–**Q15**) and the IR band of (C=N) and quinolinyl N for **L1**–**L5** and complexes **Q1**–**Q15** 9](#_Toc61431541)

[**Table S2**: Physical and chemical data of silver(I) complexes **Q1–Q15** 9](#_Toc61431542)

[Figure S1: Electronic Absorption Spectra of Ligand 1 at 5.0 X 10^5^ M in the absence (dashed line) and the presence of different concentrations of CT-DNA (0–3.0 X 10^5^ M) at 320 nm λ_max_. (inset) A stern-Volmer plot of **L1** interaction with CT- DNA 11](#_Toc61431543)

[Figure S2: Electronic Absorption Spectra of Ligand 2 at 5.0 x 10^5^ M in the absence (dashed line) and the presence of different concentrations of CT-DNA (0–3.0 x 10^5^ M) at 366 nm λ_max_. (inset) A stern-Volmer plot of **L2** interaction with CT- DNA 12](#_Toc61431544)

[Figure S3: Electronic Absorption Spectra of Ligand 3 at 5.0 x 10^5^ M in the absence (dashed line) and the presence of different concentrations of CT-DNA (0–3.0 x 10^5^ M) at 332 nm λ_max_. (inset) A stern-Volmer plot of **L3** interaction with CT- DNA 12](#_Toc61431545)

[Figure S4: Electronic Absorption Spectra of Ligand 4 at 5.0 x 10^5^ M in the absence (dashed line) and the presence of different concentrations of CT-DNA (0–3.0 x 10^5^ M) at 341 nm λ_max_. (inset) A stern-Volmer plot of **L4** interaction with CT- DNA 13](#_Toc61431546)

[Figure S5: Electronic Absorption Spectra of Ligand 5 at 5.0 x 10^5^ M in the absence (dashed line) and the presence of different concentrations of CT-DNA (0–3.0 x 10^5^ M) at 356 nm λ_max_. (inset) A stern-Volmer plot of **L5** interaction with CT- DNA 13](#_Toc61431547)

[Figure S6: Electronic Absorption Spectra of **Q1** at 5.0 x 10^5^ M in the absence (dashed line) and the presence of different concentrations of CT-DNA (0–3.0 x 10^5^ M) at 252 nm λ_max_. (inset) A stern-Volmer plot of **Q1** interaction with CT- DNA 14](#_Toc61431548)

[Figure S7: Electronic Absorption Spectra of **Q2** at 5.0 x 10^5^ M in the absence (dashed line) and the presence of different concentrations of CT-DNA (0 – 3.0 x 10^5^ M) at 370 nm λ_max_. (inset) A stern-Volmer plot of **Q2** interaction with CT- DNA 14](#_Toc61431549)

[Figure S8: Electronic Absorption Spectra of **Q3** at 5.0 x 10^5^ M in the absence (dashed line) and the presence of different concentrations of CT-DNA (0 – 3.0 x 10^5^ M) at 302 nm λ_max_. (inset) A stern-Volmer plot of **Q3** interaction with CT- DNA 15](#_Toc61431550)

[Figure S9: Electronic Absorption Spectra of **Q4** at 5.0 x 10^5^ M in the absence (dashed line) and the presence of different concentrations of CT-DNA (0 – 3.0 x 10^5^ M) at 292 nm λ_max_. (inset) A stern-Volmer plot of **Q4** interaction with CT- DNA 15](#_Toc61431551)

[Figure S10: Electronic Absorption Spectra of **Q5** at 5.0 x 10^5^ M in the absence (dashed line) and the presence of different concentrations of CT-DNA (0 – 3.0 x 10^5^ M) at 302 nm λ_max_. (inset) A stern-Volmer plot of **Q5** interaction with CT- DNA 16](#_Toc61431552)

[Figure S11: Electronic Absorption Spectra of **Q6** at 5.0 x 10^5^ M in the absence (dashed line) and the presence of different concentrations of CT-DNA (0 – 3.0 x 10^5^ M) at 249 nm λ_max_. (inset) A stern-Volmer plot of **Q6** interaction with CT- DNA 16](#_Toc61431553)

[Figure S12: Electronic Absorption Spectra of **Q7** at 5.0 x 10^5^ M in the absence (dashed line) and the presence of different concentrations of CT-DNA (0 – 3.0 x 10^5^ M) at 370 nm λ_max_. (inset) A stern-Volmer plot of **Q7** interaction with CT- DNA 17](#_Toc61431554)

[Figure S13: Electronic Absorption Spectra of **Q9** at 5.0 x 10^5^ M in the absence (dashed line) and the presence of different concentrations of CT-DNA (0 – 3.0 x 10^5^ M) at 295 nm λ_max_. (inset) A stern-Volmer plot of **Q9** interaction with CT- DNA 18](#_Toc61431555)

[Figure S14: Electronic Absorption Spectra of **Q10** at 5.0 x 10^5^ M in the absence (dashed line) and the presence of different concentrations of CT-DNA (0 – 3.0 x 10^5^ M) at 301 nm λ_max_. (inset) A stern-Volmer plot of **Q10** interaction with CT- DNA 18](#_Toc61431556)

[Figure S15: Electronic Absorption Spectra of **Q11** at 5.0 x 10^5^ M in the absence (dashed line) and the presence of different concentrations of CT-DNA (0 – 3.0 x 10^5^ M) at 251 nm λ_max_. (inset) A stern-Volmer plot of **Q11** interaction with CT- DNA 18](#_Toc61431557)

[Figure S16: Electronic Absorption Spectra of **Q12** at 5.0 x 10^5^ M in the absence (dashed line) and the presence of different concentrations of CT-DNA (0 – 3.0 x 10^5^ M) at 369 nm λ_max_. (inset) A stern-Volmer plot of **Q12** interaction with CT- DNA 19](#_Toc61431558)

[Figure S17: Electronic Absorption Spectra of **Q13** at 5.0 x 10^5^ M in the absence (dashed line) and the presence of different concentrations of CT-DNA (0 – 3.0 x 10^5^ M) at 300 nm λ_max_. (inset) A stern-Volmer plot of **Q13** interaction with CT- DNA 19](#_Toc61431559)

[Figure S18: Electronic Absorption Spectra of **Q14** at 5.0 x 10^5^ M in the absence (dashed line) and the presence of different concentrations of CT-DNA (0 – 3.0 x 10^5^ M) at 292 nm λ_max_. (inset) A stern-Volmer plot of **Q14** interaction with CT- DNA 20](#_Toc61431560)

[**Figure S19**: Electronic Absorption Spectra of **Q15** at 5.0 X 10^5^ M in the absence (dashed line) and the presence of different concentrations of CT-DNA (0–3.0 X 10^5^ M) at 302 nm λ_max_. (inset) A stern-Volmer plot of **Q15** interaction with CT- DNA 20](#_Toc61431561)

[**Figure S20:** Electronic Absorption Spectra of **silver nitrate** at 5.0 x 10^5^ M in the absence (dashed line) and the presence of different concentrations of CT-DNA (0 – 3.0 x 10^5^ M) at 302 nm λ_max_. (inset) A stern-Volmer plot of **silver nitrate** interaction with CT- DNA 21](#_Toc61431562)

[**Figure S21:** Electronic Absorption Spectra of **silver perchlorate** at 5.0 x 10^5^ M in the absence (dashed line) and the presence of different concentrations of CT-DNA (0 – 3.0 x 10^5^ M) at 302 nm λ_max_. (inset) A stern-Volmer plot of **silver perchlorate** interaction with CT- DNA 21](#_Toc61431563)

[Figure S22: Electronic Absorption Spectra of **silver triflate** at 5.0 x 10^5^ M in the absence (dashed line) and the presence of different concentrations of CT-DNA (0 – 3.0 x 10^5^ M) at 302 nm λ_max_. (inset) A stern-Volmer plot of **silver triflate** interaction with CT- DNA 22](#_Toc61431564)

[Figure S23: The Fluorescence spectra of EB-CT-DNA in the absence (dashed line) and the presence of different concentration of complex **Q1**. (inset) Stern-Volmer plot of **Q1** interaction with EB-CT- DNA 22](#_Toc61431565)

[Figure S24: The Fluorescence spectra of EB-CT-DNA in the absence (dashed line) and the presence of different concentration of complex **Q2**. (inset) Stern-Volmer plot of **Q2** interaction with EB-CT- DNA 23](#_Toc61431566)

[Figure S25: The Fluorescence spectra of EB-CT-DNA in the absence (dashed line) and the presence of different concentration of complex **Q3**. (inset) Stern-Volmer plot of **Q3** interaction with EB-CT- DNA 23](#_Toc61431567)

[Figure S26: The Fluorescence spectra of EB-CT-DNA in the absence (dashed line) and the presence of different concentration of complex **Q4**. (inset) Stern-Volmer plot of **Q4** interaction with EB-CT- DNA 24](#_Toc61431568)

[Figure S27: The Fluorescence spectra of EB-CT-DNA in the absence (dashed line) and the presence of different concentration of complex **Q5**. (inset) Stern-Volmer plot of **Q5** interaction with EB-CT- DNA 24](#_Toc61431569)

[Figure S28: The Fluorescence spectra of EB-CT-DNA in the absence (dashed line) and the presence of different concentration of complex **Q6**. (inset) Stern-Volmer plot of **Q6** interaction with EB-CT- DNA 25](#_Toc61431570)

[Figure S29: The Fluorescence spectra of EB-CT-DNA in the absence (dashed line) and the presence of different concentration of complex **Q7**. (inset) Stern-Volmer plot of **Q7** interaction with EB-CT- DNA 25](#_Toc61431571)

[Figure S30: The Fluorescence spectra of EB-CT-DNA in the absence (dashed line) and the presence of different concentration of complex **Q9**. (inset) Stern-Volmer plot of **Q9** interaction with EB-CT- DNA 26](#_Toc61431572)

[Figure S31: The Fluorescence spectra of EB-CT-DNA in the absence (dashed line) and the presence of different concentration of complex **Q10**. (inset) Stern-Volmer plot of **Q10** interaction with EB-CT- DNA 26](#_Toc61431573)

[Figure S32: The Fluorescence spectra of EB-CT-DNA in the absence (dashed line) and the presence of different concentration of complex **Q11**. (inset) Stern-Volmer plot of **Q11** interaction with EB-CT- DNA 27](#_Toc61431574)

[Figure S33: The Fluorescence spectra of EB-CT-DNA in the absence (dashed line) and the presence of different concentration of complex **Q12**. (inset) Stern-Volmer plot of **Q12** interaction with EB-CT- DNA 27](#_Toc61431575)

[Figure S34: The Fluorescence spectra of EB-CT-DNA in the absence (dashed line) and the presence of different concentration of complex **Q13**. (inset) Stern-Volmer plot of **Q13** interaction with EB-CT- DNA 28](#_Toc61431576)

[Figure S35: The Fluorescence spectra of EB-CT-DNA in the absence (dashed line) and the presence of different concentration of complex **Q14**. (inset) Stern-Volmer plot of **Q14** interaction with EB-CT- DNA 28](#_Toc61431577)

[Figure S36: The Fluorescence spectra of EB-CT-DNA in the absence (dashed line) and the presence of different concentration of complex **Q15**. (inset) Stern-Volmer plot of **Q15** interaction with EB-CT- DNA 29](#_Toc61431578)

[Figure S37: The Fluorescence spectra of EB-CT-DNA in the absence (dashed line) and the presence of different concentration of **silver nitrate**. (inset) Stern-Volmer plot of **silver nitrate** interaction with EB-CT- DNA 29](#_Toc61431579)

[Figure S38: The Fluorescence spectra of EB-CT-DNA in the absence (dashed line) and the presence of different concentration of **silver perchlorate**. (inset) Stern-Volmer plot of **silver perchlorate** interaction with EB-CT- DNA 30](#_Toc61431580)

[Figure S39: The Fluorescence spectra of EB-CT-DNA in the absence (dashed line) and the presence of different concentration of **silver triflate**. (inset) Stern-Volmer plot of **silver triflate** interaction with EB-CT- DNA 30](#_Toc61431581)

[**The double-logarithmic plot of EB-CT-DNA–Complexes interactions at room temperature.** 31](#_Toc61431582)

[Figure S40: The double-logarithmic plot of EB-CT-DNA–Complex **Q1** interaction at room temperature. 31](#_Toc61431583)

[Figure S42: The double-logarithmic plot of EB-CT-DNA–Complex **Q3** interaction at room temperature. 32](#_Toc61431584)

[Figure S44: The double-logarithmic plot of EB-CT-DNA–Complex **Q5** interaction at room temperature. 33](#_Toc61431585)

[Figure S46: The double-logarithmic plot of EB-CT-DNA–Complex **Q7** interaction at room temperature. 34](#_Toc61431586)

[Figure S48: The double-logarithmic plot of EB-CT-DNA–Complex **Q9** interaction at room temperature. 35](#_Toc61431587)

[Figure S50: The double-logarithmic plot of EB-CT-DNA–Complex **Q12** interaction at room temperature. 36](#_Toc61431588)

[Figure S54: The double-logarithmic plot of EB-CT-DNA–silver nitrate interaction at room temperature. 38](#_Toc61431589)

[Figure S56: The double-logarithmic plot of EB-CT-DNA–silver triflate interaction at room temperature. 39](#_Toc61431590)

[**BSA Binding studies using Electronic Absorption method** 39](#_Toc61431591)

[Figure S57: Electronic Absorption Spectra of BSA in the absence (dashed line) and the presence of different concentrations of complexes **Q1**. (inset) Plot of 1/(Aₒ ‒ A) vs. 1/[Complex] x 10^-4^ M^-1^ 39](#_Toc61431592)

[Figure S58: Electronic Absorption Spectra of BSA in the absence (dashed line) and the presence of different concentrations of complexes **Q2**. (inset) Plot of 1/(Aₒ ‒ A) vs. 1/[Complex] x 10^-4^ M^-1^ 40](#_Toc61431593)

[Figure S59: Electronic Absorption Spectra of BSA in the absence (dashed line) and the presence of different concentrations of complexes **Q3**. (inset) Plot of 1/(Aₒ ‒ A) vs. 1/[Complex] x 10^-4^ M^-1^ 40](#_Toc61431594)

[Figure S60: Electronic Absorption Spectra of BSA in the absence (dashed line) and the presence of different concentrations of complexes **Q4**. (inset) Plot of 1/(Aₒ ‒ A) vs. 1/[Complex] x 10^-4^ M^-1^ 41](#_Toc61431595)

[Figure S61: Electronic Absorption Spectra of BSA in the absence (dashed line) and the presence of different concentrations of complexes **Q5**. (inset) Plot of 1/(Aₒ ‒ A) vs. 1/[Complex] x 10^-4^ M^-1^ 41](#_Toc61431596)

[Figure S62: Electronic Absorption Spectra of BSA in the absence (dashed line) and the presence of different concentrations of complexes **Q6**. (inset) Plot of 1/(Aₒ ‒ A) vs. 1/[Complex] x 10^-4^ M^-1^ 42](#_Toc61431597)

[Figure S63: Electronic Absorption Spectra of BSA in the absence (dashed line) and the presence of different concentrations of complexes **Q7**. (inset) Plot of 1/(Aₒ ‒ A) vs. 1/[Complex] x 10^-4^ M^-1^ 42](#_Toc61431598)

[Figure S64: Electronic Absorption Spectra of BSA in the absence (dashed line) and the presence of different concentrations of complexes **Q8**. (inset) Plot of 1/(Aₒ ‒ A) vs. 1/[Complex] x 10^-4^ M^-1^ 43](#_Toc61431599)

[Figure S65: Electronic Absorption Spectra of BSA in the absence (dashed line) and the presence of different concentrations of complexes **Q9**. (inset) Plot of 1/(Aₒ ‒ A) vs. 1/[Complex] x 10^-4^ M^-1^ 43](#_Toc61431600)

[Figure S66: Electronic Absorption Spectra of BSA in the absence (dashed line) and the presence of different concentrations of complexes **Q10**. (inset) Plot of 1/(Aₒ ‒ A) vs. 1/[Complex] x 10^-4^ M^-1^ 44](#_Toc61431601)

[Figure S67: Electronic Absorption Spectra of BSA in the absence (dashed line) and the presence of different concentrations of complexes **Q11**. (inset) Plot of 1/(Aₒ ‒ A) vs. 1/[Complex] x 10^-4^ M^-1^ 44](#_Toc61431602)

[Figure S68: Electronic Absorption Spectra of BSA in the absence (dashed line) and the presence of different concentrations of complexes **Q13**. (inset) Plot of 1/(Aₒ ‒ A) vs. 1/[Complex] x 10^-4^ M^-1^ 45](#_Toc61431603)

[Figure S69: Electronic Absorption Spectra of BSA in the absence (dashed line) and the presence of different concentrations of complexes **Q14**. (inset) Plot of 1/(Aₒ ‒ A) vs. 1/[Complex] x 10^-4^ M^-1^ 45](#_Toc61431604)

[Figure S70: Electronic Absorption Spectra of BSA in the absence (dashed line) and the presence of different concentrations of complexes **Q15**. (inset) Plot of 1/(Aₒ ‒ A) vs. 1/[Complex] x 10^-4^ M^-1^ 46](#_Toc61431605)

[Figure S71: Electronic Absorption Spectra of BSA in the absence (dashed line) and the presence of different concentrations of complexes silver nitrate. (inset) Plot of 1/(Aₒ ‒ A) vs. 1/[Complex] x 10^-4^ M^-1^ 46](#_Toc61431606)

[Figure S72: Electronic Absorption Spectra of BSA in the absence (dashed line) and the presence of different concentrations of complexes silver perchlorate. (inset) Plot of 1/(Aₒ ‒ A) vs. 1/[Complex] x 10^-4^ M^-1^ 47](#_Toc61431607)

[Figure S73: Electronic Absorption Spectra of BSA in the absence (dashed line) and the presence of different concentrations of complexes silver trifluoromethanesulfonate. (inset) Plot of 1/(Aₒ ‒ A) vs. 1/[Complex] x 10^-4^ M^-1^ 47](#_Toc61431608)

[**BSA Binding studies using the Fluorescence method** 47](#_Toc61431609)

[**Figure S74:** Fluorescence emission spectra of BSA in the absence(dashed line) and the presence of different concentration of complex **Q1**. (inset) Stern-Volmer plot of complex **Q1** interaction with BSA 48](#_Toc61431610)

[**Figure S75:**Fluorescence emission spectra of BSA in the absence(dashed line) and the presence of different concentration of complex **Q6**. (inset) Stern-Volmer plot of complex **Q6** interaction with BSA 48](#_Toc61431611)

[**The double-logarithmic plot of BSA–Complexes interactions at room temperature.** 48](#_Toc61431612)

[**Figure S76:** The double-logarithmic plot of BSA–Complex **Q1** interactions. 49](#_Toc61431613)

[**Figure S77:** The double-logarithmic plot of BSA–Complex **E2** interactions. 49](#_Toc61431614)

[**^1^H-NMR Spectra of Ligands L1-L5** 50](#_Toc61431615)

[Figure S78: (E)-N-(2-fluorophenyl)-1-(quinolin-2-yl)methanimine L1 50](#_Toc61431616)

[Figure S79: 2-(quinolin-2-yl)benzo[d]thiazole L2 50](#_Toc61431617)

[Figure S80: (E)-N-(4-chlorophenyl)-1-(quinolin-2-yl)methanimine L3 51](#_Toc61431618)

[Figure S81: (E)-1-(quinolin-2-yl)-N-(p-tolyl)methanimine L4 51](#_Toc61431619)

[Figure S82: (E)-1-(quinolin-2-yl)-N-(thiophen-2-ylmethyl)methanimine L5 52](#_Toc61431620)

[**^1^H-NMR Spectra of complexes Q1-Q5** 52](#_Toc61431621)

[Figure S83: ***[Ag(L1)_2_]NO_3_***  ***Q1*** 52](#_Toc61431622)

[Figure S84: ***[Ag(L2)_2_]NO_3_***  ***Q2*** 53](#_Toc61431623)

[Figure S85: ***[Ag(L3)_2_]NO_3_***  ***Q3*** 53](#_Toc61431624)

[Figure S86: ***[Ag(L4)_2_]NO_3_***  ***Q4*** 54](#_Toc61431625)

[Figure S87: ***[Ag(L5)_2_]NO_3_***  ***Q5*** 54](#_Toc61431626)

[Figure S88: ***[Ag(L1)_2_]ClO_4_***  ***Q6*** 55](#_Toc61431627)

[Figure S89: ***[Ag(L2)_2_]ClO_4_***  ***Q7*** 55](#_Toc61431628)

[Figure S90: ***[Ag(L3)_2_]ClO_4_***  ***Q8*** 56](#_Toc61431629)

[Figure S91: ***[Ag(L4)_2_]ClO_4_***  ***Q9*** 56](#_Toc61431630)

[Figure S92: ***[Ag(L5)_2_]ClO_4_***  ***Q10*** 57](#_Toc61431631)

[Figure S93: ***[Ag(L1)_2_]CF_3_SO_3_***  ***Q11*** 57](#_Toc61431632)

[Figure S94: ***[Ag(L2)_2_]CF_3_SO_3_***  ***Q12*** 58](#_Toc61431633)

[Figure S95: ***[Ag(L3)_2_]CF_3_SO_3_***  ***Q13*** 58](#_Toc61431634)

[Figure S96: ***[Ag(L4)_2_]CF_3_SO_3_*** ***Q14*** 59](#_Toc61431635)

[Figure S97: ***[Ag(L5)_2_]CF_3_SO_3_***  ***Q15*** 59](#_Toc61431636)

[**^13^C-NMR SPECTRA OF L1-L5** 60](#_Toc61431637)

[Figure S98: (E)-N-(2-fluorophenyl)-1-(quinolin-2-yl)methanimine L1 60](#_Toc61431638)

[Figure S99: 2-(quinolin-2-yl)benzo[d]thiazole L2 60](#_Toc61431639)

[Figure S100: (E)-N-(4-chlorophenyl)-1-(quinolin-2-yl)methanimine L3 61](#_Toc61431640)

[Figure S101: (E)-1-(quinolin-2-yl)-N-(p-tolyl)methanimine L4 61](#_Toc61431641)

[Figure S102: (E)-1-(quinolin-2-yl)-N-(thiophen-2-ylmethyl)methanimine L5 62](#_Toc61431642)

[**^13^C-NMR SPECTRA OF COMPLEXES Q1-Q15** 62](#_Toc61431643)

[Figure S103: ***[Ag(L1)_2_]NO_3_***  ***Q1*** 62](#_Toc61431644)

[Figure S104: ***[Ag(L2)_2_]NO_3_***  ***Q2*** 63](#_Toc61431645)

[Figure S105: ***[Ag(L3)_2_]NO_3_***  ***Q3*** 63](#_Toc61431646)

[Figure S106: ***[Ag(L4)_2_]NO_3_***  ***Q4*** 64](#_Toc61431647)

[Figure S107: ***[Ag(L5)_2_]NO_3_***  ***Q5*** 64](#_Toc61431648)

[Figure S108: ***[Ag(L1)_2_]ClO_4_***  ***Q6*** 65](#_Toc61431649)

[Figure S109: ***[Ag(L2)_2_]ClO_4_***  ***Q7*** 65](#_Toc61431650)

[Figure S110: ***[Ag(L3)_2_]ClO_4_***  ***Q8*** 66](#_Toc61431651)

[Figure S111: ***[Ag(L4)_2_]ClO_4_***  ***Q9*** 66](#_Toc61431652)

[Figure S112: ***[Ag(L5)_2_]ClO_4_***  ***Q10*** 67](#_Toc61431653)

[Figure S113: ***[Ag(L1)_2_]CF_3_SO_3_***  ***Q11*** 67](#_Toc61431654)

[Figure S114: ***[Ag(L2)_2_]CF_3_SO_3_***  ***Q12*** 68](#_Toc61431655)

[Figure S115: ***[Ag(L3)_2_]CF_3_SO_3_***  ***Q13*** 68](#_Toc61431656)

[Figure S116: ***[Ag(L4)_2_]CF_3_SO_3_*** ***Q14*** 69](#_Toc61431657)

[Figure S117: ***[Ag(L5)_2_]CF_3_SO_3_***  ***Q15*** 69](#_Toc61431658)

[IR SPECTRA OF LIGANDS L1-L5 70](#_Toc61431659)

[Figure S118: (E)-N-(2-fluorophenyl)-1-(quinolin-2-yl)methanimine L1 70](#_Toc61431660)

[Figure S119: 2-(quinolin-2-yl)benzo[d]thiazole L2 70](#_Toc61431661)

[Figure S120: (E)-N-(4-chlorophenyl)-1-(quinolin-2-yl)methanimine L3 71](#_Toc61431662)

[Figure S121: (E)-1-(quinolin-2-yl)-N-(p-tolyl)methanimine L4 71](#_Toc61431663)

[Figure S122: (E)-1-(quinolin-2-yl)-N-(thiophen-2-ylmethyl)methanimine ***L5*** 72](#_Toc61431664)

[IR SPECTRA OF COMPLEXES Q1-Q15 72](#_Toc61431665)

[Figure S123: ***[Ag(L1)_2_]NO_3_***  ***Q1*** 72](#_Toc61431666)

[Figure S124: ***[Ag(L2)_2_]NO_3_***  ***Q2*** 73](#_Toc61431667)

[Figure S125: ***[Ag(L3)_2_]NO_3_***  ***Q3*** 73](#_Toc61431668)

[Figure S126: ***[Ag(L4)_2_]NO_3_***  ***Q4*** 74](#_Toc61431669)

[Figure S127: ***[Ag(L5)_2_]NO_3_***  ***Q5*** 74](#_Toc61431670)

[Figure S128: ***[Ag(L1)_2_]ClO_4_***  ***Q6*** 75](#_Toc61431671)

[Figure S129: ***[Ag(L2)_2_]ClO_4_***  ***Q7*** 75](#_Toc61431672)

[Figure S130: ***[Ag(L3)_2_]ClO_4_***  ***Q8*** 76](#_Toc61431673)

[Figure S131: ***[Ag(L4)_2_]ClO_4_***  ***Q9*** 76](#_Toc61431674)

[Figure S132: ***[Ag(L5)_2_]ClO_4_***  ***Q10*** 77](#_Toc61431675)

[Figure S133: ***[Ag(L1)_2_]CF_3_SO_3_***  ***Q11*** 77](#_Toc61431676)

[Figure S134: ***[Ag(L2)_2_]CF_3_SO_3_***  ***Q12*** 78](#_Toc61431677)

[Figure S135: ***[Ag(L3)_2_]CF_3_SO_3_***  ***Q13*** 78](#_Toc61431678)

[Figure S136: ***[Ag(L4)_2_]CF_3_SO_3_*** ***Q14*** 79](#_Toc61431679)

[Figure S137: ***[Ag(L5)_2_]CF_3_SO_3_***  ***Q15*** 79](#_Toc61431680)

[**Mass Spectra of Ligands L1-L5** 80](#_Toc61431681)

[Figure S138: (E)-N-(2-fluorophenyl)-1-(quinolin-2-yl)methanimine L1 80](#_Toc61431682)

[Figure S139: 2-(quinolin-2-yl)benzo[d]thiazole L2 80](#_Toc61431683)

[Figure S140: (E)-N-(4-chlorophenyl)-1-(quinolin-2-yl)methanimine L3 81](#_Toc61431684)

[Figure S142: (E)-1-(quinolin-2-yl)-N-(thiophen-2-ylmethyl)methanimine L5 82](#_Toc61431685)

[**MASS SPECTRA OF COMPLEXES Q1-Q15** 82](#_Toc61431686)

[Figure S143: ***[Ag(L1)_2_]NO_3_***  ***Q1*** 82](#_Toc61431687)

[Figure S144: ***[Ag(L2)_2_]NO_3_***  ***Q2*** 83](#_Toc61431688)

[Figure S145: ***[Ag(L3)_2_]NO_3_***  ***Q3*** 83](#_Toc61431689)

[Figure S146: ***[Ag(L4)_2_]NO_3_***  ***Q4*** 84](#_Toc61431690)

[Figure S147: ***[Ag(L5)_2_]NO_3_***  ***Q5*** 84](#_Toc61431691)

[Figure S148: ***[Ag(L1)_2_]ClO_4_ Q6*** 85](#_Toc61431692)

[Figure S149: ***[Ag(L2)_2_]ClO_4_***  ***Q7*** 85](#_Toc61431693)

[Figure S150: ***[Ag(L3)_2_]ClO_4_***  ***Q8*** 86](#_Toc61431694)

[Figure S151: ***[Ag(L4)_2_]ClO_4_***  ***Q9*** 86](#_Toc61431695)

[Figure S152: ***[Ag(L5)_2_]ClO_4_***  ***Q10*** 87](#_Toc61431696)

[Figure S153: ***[Ag(L1)_2_]CF_3_SO_3_***  ***Q11*** 87](#_Toc61431697)

[Figure S154: ***[Ag(L2)_2_]CF_3_SO_3_***  ***Q12*** 88](#_Toc61431698)

[Figure S155: ***[Ag(L3)_2_]CF_3_SO_3_***  ***Q13*** 88](#_Toc61431699)

[Figure S156: ***[Ag(L4)_2_]CF_3_SO_3_*** ***Q14*** 89](#_Toc61431700)

[Figure S157: ***[Ag(L5)_2_]CF_3_SO_3_***  ***Q15*** 89](#_Toc61431701)

## **Table S1:** ^1^H-NMR chemical shifts of some protons in **L1**–**L5** complexes (**Q1**–**Q15**) and the IR band of (C=N) and quinolinyl N for **L1**–**L5** and complexes **Q1**–**Q15**

| Ligands (Complex) | δC(H=N)ppm | Δδ | Ha-Qui | Δδ | ѵ(C=N) cm^-1^ | Δѵ | ѵ(Qui―N) cm^-1^ | Δѵ |
| --- | --- | --- | --- | --- | --- | --- | --- | --- |
| L1 (Q1) | 8.83 (8.93) | 0.10 | 8.37 (8.12) | 0.25 | 1618 (1612) | 6 | 1594 (1588) | 6 |
| L2 (Q2) | - | - | 8.15 (8.17) | 0.02 | - | - | 1591 (1587) | 3 |
| L3 (Q3) | 8.76 (9.08) | 0.32 | 8.46 (8.17) | 0.29 | 1621 (1613) | 8 | 1588 (1584) | 4 |
| L4 (Q4) | 8.76 (9.21) | 0.45 | 8.03 (8.14) | 0.11 | 1621 (1684) | 63 | 1593 (1586) | 7 |
| L5 (Q5) | 8.62 (9.03) | 0.41 | 7.99 (8.01) | 0.02 | 1636 (1645) | 9 | 1592 (1589) | 3 |
| L1 (Q6) | 8.83 (8.98) | 0.15 | 8.37 (8.13) | 0.24 | 1618 (1624) | 6 | 1594 (1585) | 9 |
| L2 (Q7) | - | - | 8.15 (8.17) | 0.02 |  | - | 1591 (1586) | 5 |
| L3 (Q5) | 8.76 (9.19) | 0.43 | 8.46 (8.18) | 0.28 | 1621 (-) | - | 1588 (1592) | 4 |
| L4 (Q9) | 8.76 (9.20) | 0.44 | 8.03 (8.15) | 0.12 | 1621 (1682) | 61 | 1593 (1587) | 6 |
| L5 (Q10) | 8.62 (9.02) | 0.40 | 7.99 (7.71) | 0.28 | 1636 (1644) | 8 | 1592 (1589) | 3 |
| L1 (Q11) | 8.83 (9.17) | 0.34 | 8.37 (8.17) | 0.20 | 1618 (1624) | 6 | 1594 (1585) | 9 |
| L2 (Q12) | - | - | 8.15 (8.16) | 0.01 |  | - | 1591 (1588) | 3 |
| L3 (Q13) | 8.76 (9.16) | 0.40 | 8.46 (8.17) | 0.29 | 1621 (-) | - | 1588 (1591) | 3 |
| L4 (Q14) | 8.76 (9.04) | 0.28 | 8.03 (8.14) | 0.11 | 1621 (1626) | 5 | 1593 (1584) | 9 |
| L5 (Q15) | 8.62 (8.84) | 0.22 | 7.99 (7.99) | 0.00 | 1636 (1646) | 10 | 1592 (1591) | 1 |

## **Table S2**: Physical and chemical data of silver(I) complexes **Q1–Q15**

| Comp. | Anal. Calcd.(Anal. found)  C H N | M.pt.  (°C) | MS  Calcd. Found | MC  (Λ_m_/ S m^2^ mol^-1^)  X 10^4^ |
| --- | --- | --- | --- | --- |
| **Q1** | 49.74 (49.67) 3.23 (3.14) 10.55 (10.38) | 152-153 | 468 468 | 2.81 |
| **Q2** | 55.34 (55.23) 2.90 (2.85) 10.08 (9.92) | 266-267 | 632.53 633 | 2.83 |
| **Q3** | 54.65 (54.32) 3.15 (3.15) 9.96 (9.93) | 182-183 | 641.32 641 | 2.05 |
| **Q4** | 61.64 (61.54) 4.26 (4.04) 10.57 (10.38) | 141-142 | 600.49 601 | 0.30 |
| **Q5** | 53.42 (53.14) 3.59 (3.29) 10.38 (10.19) | 134-135 | 612.54 613 | 1.57 |
| **Q6** | 46.46 (46.32) 3.01 (2.89) 7.39 (7.14) | 198-199 | 468 468 | 4.05 |
| **Q7** | 52.31 (52.16) 2.74 (2.64) 7.63 (7.60) | 242-243 | 632.53 633 | 2.16 |
| **Q8** | 51.89 (51.69) 2.99 (2.85) 7.56 (7.31) | 190-191 | 641.32 641 | 1.82 |
| **Q9** | 58.34 (58.11) 4.03 (3.98) 8.00 (7.92) | 149-150 | 600.49 601 | 0.61 |
| **Q10** | 50.61 (50.32) 3.40 (3.40) 7.87 (7.83) | 132-133 | 612.54 613 | 1.45 |
| **Q11** | 44.68 (44.62) 2.77 (2.51) 6.80 (6.73) | 154-155 | 468 468 | 3.05 |
| **Q12** | 39.32 (39.15) 1.94 (1.78) 5.39 (5.27) | 245-246 | 632.53 633 | 2.49 |
| **Q13** | 50.15 (50.15) 2.81 (2.70) 7.09 (6.98) | 193-194 | 641.32 641 | 1.13 |
| **Q14** | 56.08 (55.97) 3.77 (3.67) 7.47 (7.23) | 152-153 | 600.49 601 | 0.28 |
| **Q15** | 48.89 (48.65) 3.18 (3.11) 7.36 (7.24) | 148-149 | 612.54 613 | 1.05 |

**Table S3.** Crystal data and structure refinement for complexes **Q1**, **Q6**, **Q7**, **Q12** and **Q14.**

|  | **Q1** | **Q6** | **Q7** | **Q12** | **Q14** |
| --- | --- | --- | --- | --- | --- |
| Chemical formula | C_22_H_17_AgF_2_N_4_O_3_ | C_22_H_17_AgClF_2_N_3_O_4_ | C_48_H_30_Ag_2_Cl_2_N_6_O_8_S_3_ | C_34_H_20_Ag_2_F_6_N_4_O_6_S_4_ | C_18_AgF_3_N_2_O_3_S_2_H_0.5_ |
| Formula weight | 531.12 | 568.70 | 1201.60 | 1038.552 | 521.69 |
| Crystal system | Triclinic | Triclinic | monoclinic | Monoclinic | triclinic |
| Space group | *P*$\bar{1}$ | *P*$\bar{1}$ | C2/c | *P*2_1_/n | *P*$\bar{1}$ |
| *a* (Å) | 9.7976(6) | 9.5076(8) | 25.699(2) | 9.0225(8) | 7.2354(8) |
| *b* (Å) | 9.9760(6) | 10.7916(8) | 13.3032(9) | 19.5615(18) | 11.0217(9) |
| *c* (Å) | 10.4066(7) | 10.8342(9) | 14.6474(10) | 10.8179(10) | 13.2506(14) |
| *α* (°) | 80.685(3) | 84.951(4) | 90 | 90 | 80.002(6) |
| *β* (°) | 86.1270(10) | 87.905(4) | 117.937(5) | 114.453(4) | 74.283(4) |
| *γ* (°) | 86.991(3) | 75.382(4) | 90 | 90 | 72.846(4) |
| *V* (Å^3^) | 1000.55(11) | 1071.34(15) | 4424.0(6) | 1738.0(3) | 966.87(17) |
| *Z* | 2 | 2 | 4 | 2 | 2 |
| *ρ*_calc_ (gcm^-3^) | 1.763 | 1.763 | 1.804 | 1.984 | 1.792 |
| *µ* (mm^−1^) | 1.060 | 1.119 | 1.213 | 1.454 | 1.308 |
| *F*(000) | 532.0 | 568.0 | 2400.0 | 1022.1 | 505.0 |
| Crystal size (mm^3^) | 0.26×0.22×0.12 | 0.38 × 0.28 × 0.26 | 0.28 × 0.24 × 0.16 | 0.25 × 0.21 × 0.14 | 0.54 × 0.14 × 0.12 |
| *θ* range for data collection (°) | 3.974 to 56.52 | 3.774 to 56.41 | 3.548 to 56.612 | 4.16 to 53.56 | 3.21 to 58.2 |
| Index ranges | -13 ≤ h ≤ 12  -13 ≤ k ≤ 12  -13 ≤ l ≤ 13 | -12 ≤ h ≤ 12  -14 ≤ k ≤ 14  -13 ≤ l ≤ 14 | -34 ≤ h ≤ 34,  -17 ≤ k ≤ 17,  -19 ≤ l ≤ 19 | -11 ≤ h ≤ 11  -22 ≤ k ≤ 24  -13 ≤ l ≤ 13 | -9 ≤ h ≤ 4  -15 ≤ k ≤ 14  -18 ≤ l ≤ 17 |
| Reflections collected | 17942 | 18983 | 36696 | 26716 | 18893 |
| Independent reflections | 4846 [R_int_ = 0.0211] | 5178 [R_int_ = 0.0182] | 5382 [R_int_ = 0.0573] | 3702 [R_int_ = 0.0320] | \|  \| 5057 [R_int_ = 0.0287] \| \| --- \| --- \| |
| Completeness to theta = 28.96 | 98.0 % | 98.0 % | 99 % | 100 % | 98.0 % |
| Data / restraints / parameters | 4846 / 0 / 289 | 5178 / 0 / 298 | 5382 / 633 / 430 | 3702 / 173 / 280 | 5057 / 0 / 271 |
| Goodness-of-fit on F^2^ | 1.060 | 1.054 | 1.104 | 1.134 | 0.998 |
| R indices [I>2sigma(I)] *R_1,_  wR_2_* | 0.025,1 0.0625 | 0.0266, 0.0667 | 0.0579, 0.1159 | 0.0422, 0.0874 | 0.0273, 0.0666 |
| R indices (all data)  *R_1,_  wR_2_* | 0.0272, 0.0636 | 0.0299, 0.0685 | 0.0604, 0.1173 | 0.0442, 0.0888 | 0.0316, 0.0690 |
| Largest diff. peak and hole (e Å^-3^) | 1.20 and -0.44 | 0.98 and -0.43 | 1.36/-0.70 | 2.07 and -0.98 | 1.09 and -0.43 |


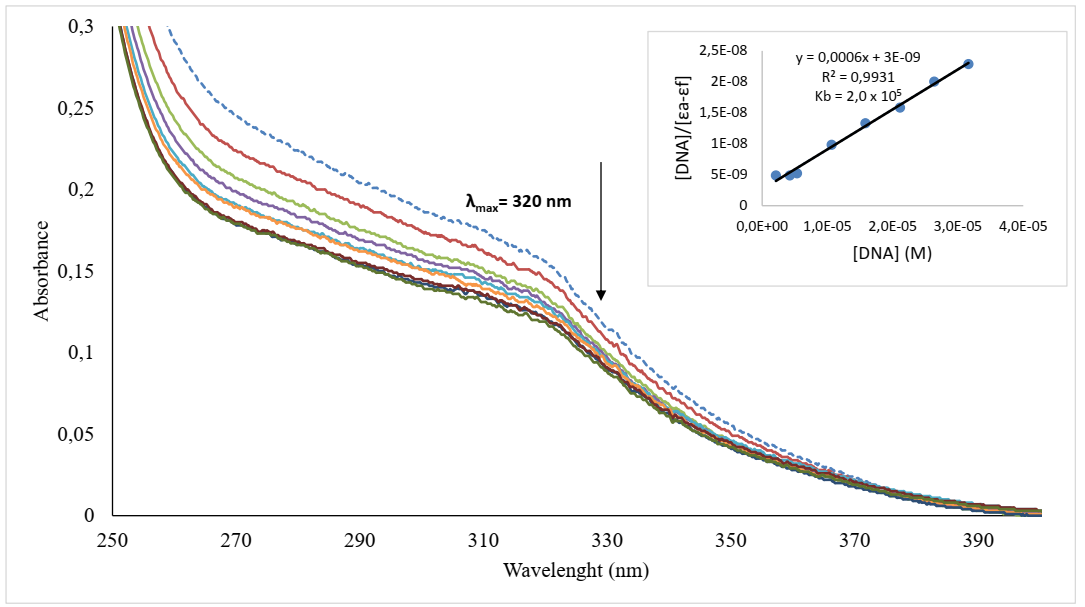


## Figure S1: Electronic Absorption Spectra of Ligand 1 at 5.0 X 10^5^ M in the absence (dashed line) and the presence of different concentrations of CT-DNA (0–3.0 X 10^5^ M) at 320 nm λ_max_. (inset) A stern-Volmer plot of **L1** interaction with CT- DNA


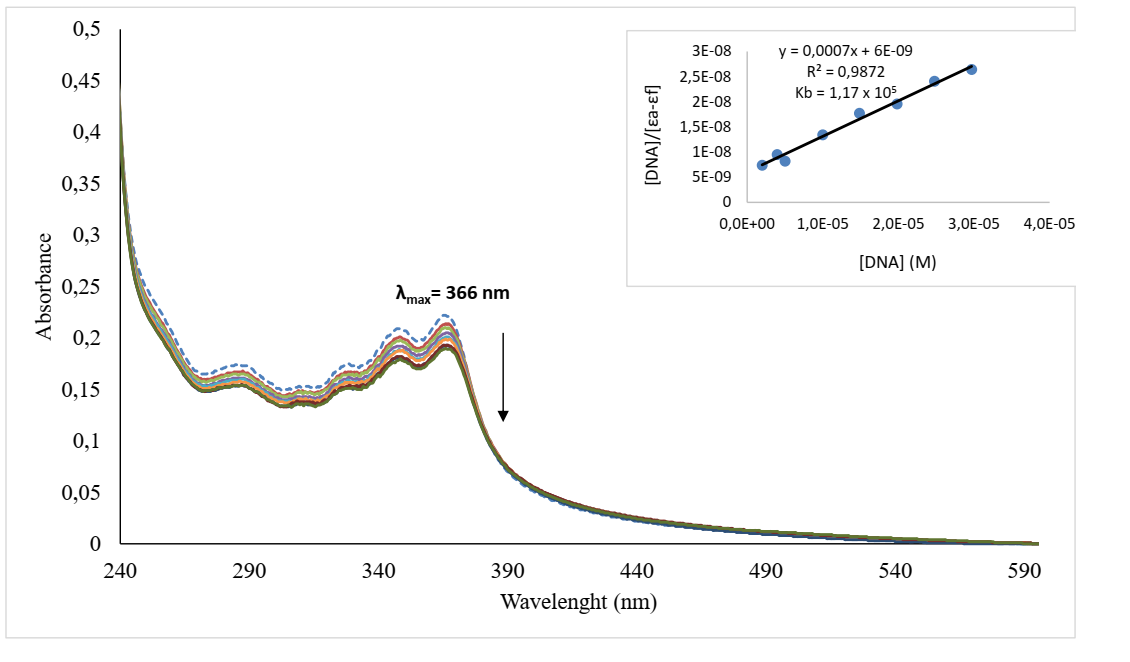


## Figure S2: Electronic Absorption Spectra of Ligand 2 at 5.0 x 10^5^ M in the absence (dashed line) and the presence of different concentrations of CT-DNA (0–3.0 x 10^5^ M) at 366 nm λ_max_. (inset) A stern-Volmer plot of **L2** interaction with CT- DNA


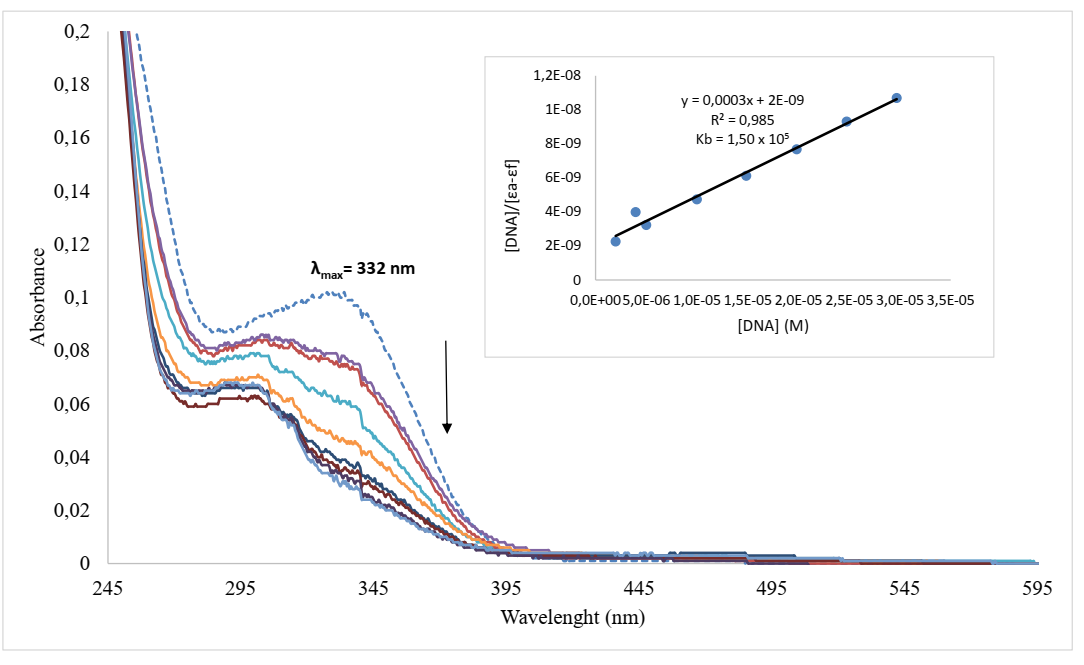


## Figure S3: Electronic Absorption Spectra of Ligand 3 at 5.0 x 10^5^ M in the absence (dashed line) and the presence of different concentrations of CT-DNA (0–3.0 x 10^5^ M) at 332 nm λ_max_. (inset) A stern-Volmer plot of **L3** interaction with CT- DNA


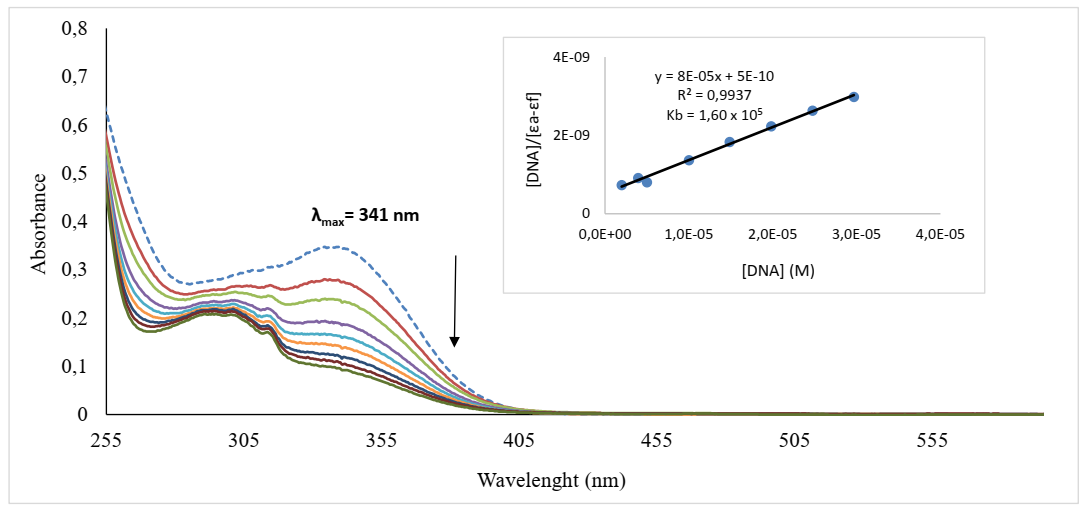


## Figure S4: Electronic Absorption Spectra of Ligand 4 at 5.0 x 10^5^ M in the absence (dashed line) and the presence of different concentrations of CT-DNA (0–3.0 x 10^5^ M) at 341 nm λ_max_. (inset) A stern-Volmer plot of **L4** interaction with CT- DNA


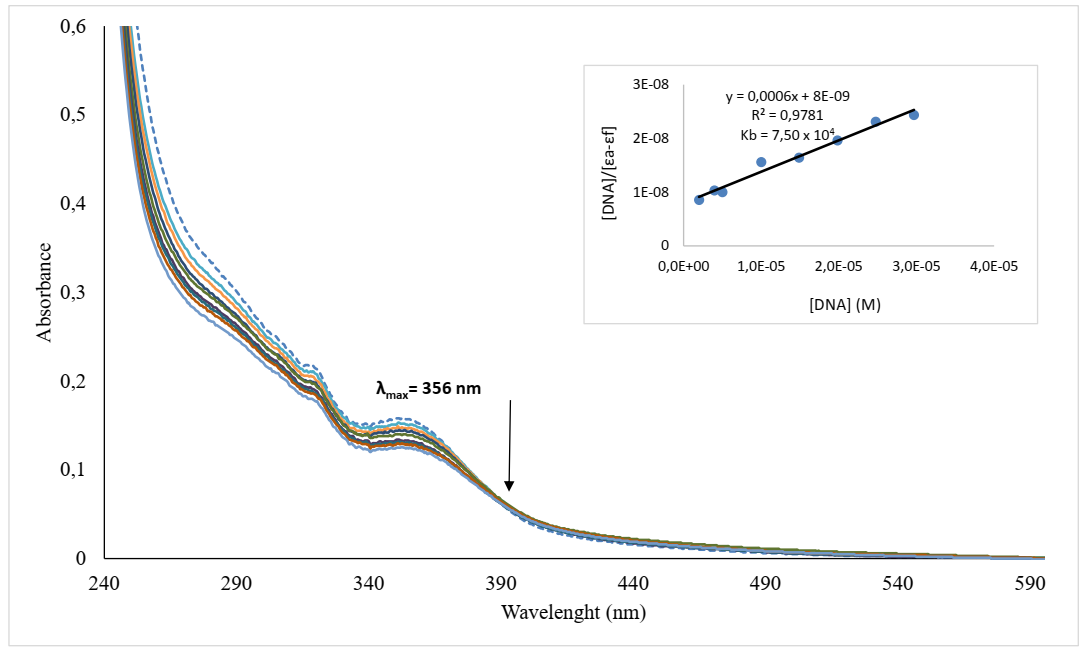


## Figure S5: Electronic Absorption Spectra of Ligand 5 at 5.0 x 10^5^ M in the absence (dashed line) and the presence of different concentrations of CT-DNA (0–3.0 x 10^5^ M) at 356 nm λ_max_. (inset) A stern-Volmer plot of **L5** interaction with CT- DNA


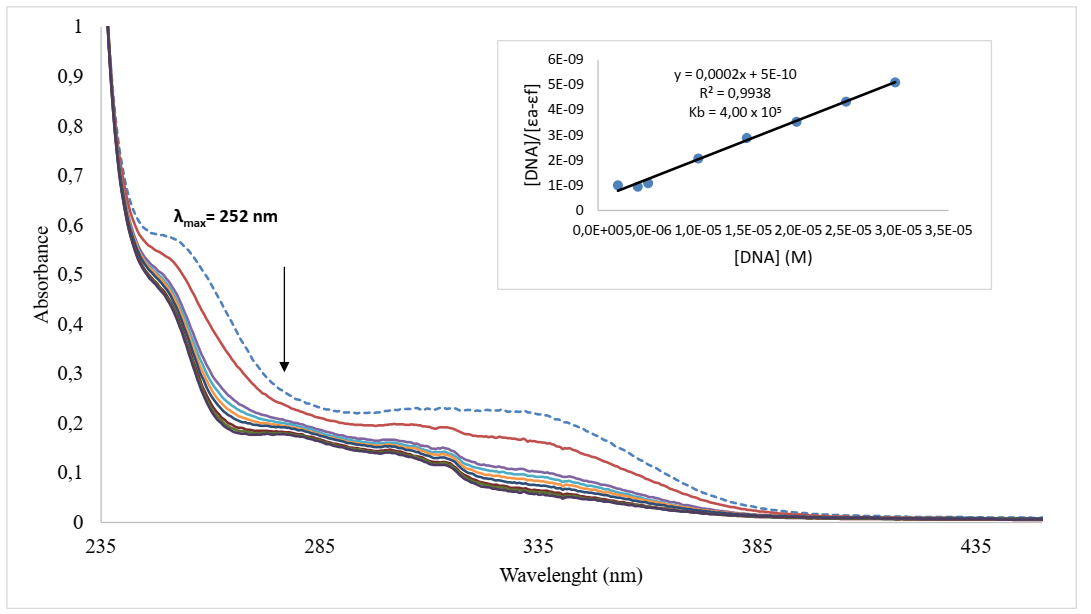


## Figure S6: Electronic Absorption Spectra of **Q1** at 5.0 x 10^5^ M in the absence (dashed line) and the presence of different concentrations of CT-DNA (0–3.0 x 10^5^ M) at 252 nm λ_max_. (inset) A stern-Volmer plot of **Q1** interaction with CT- DNA


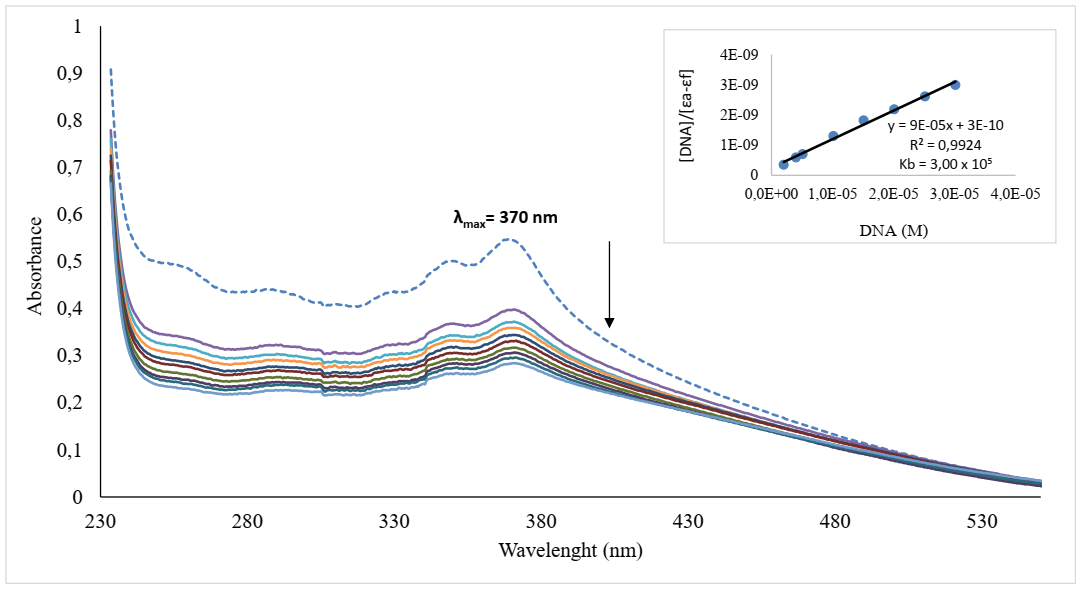


## Figure S7: Electronic Absorption Spectra of **Q2** at 5.0 x 10^5^ M in the absence (dashed line) and the presence of different concentrations of CT-DNA (0 – 3.0 x 10^5^ M) at 370 nm λ_max_. (inset) A stern-Volmer plot of **Q2** interaction with CT- DNA


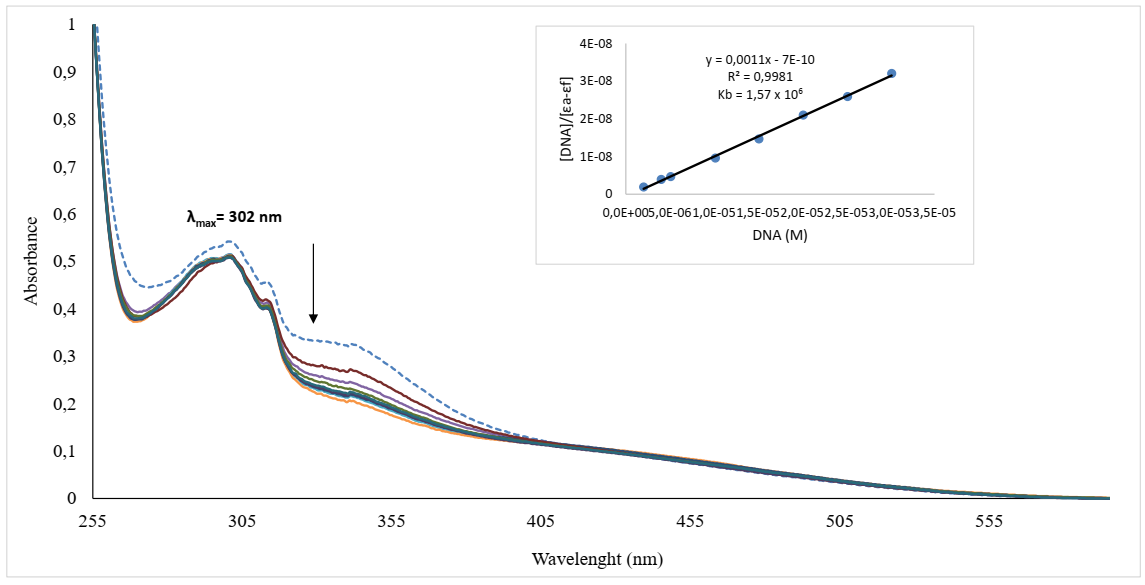


## Figure S8: Electronic Absorption Spectra of **Q3** at 5.0 x 10^5^ M in the absence (dashed line) and the presence of different concentrations of CT-DNA (0 – 3.0 x 10^5^ M) at 302 nm λ_max_. (inset) A stern-Volmer plot of **Q3** interaction with CT- DNA


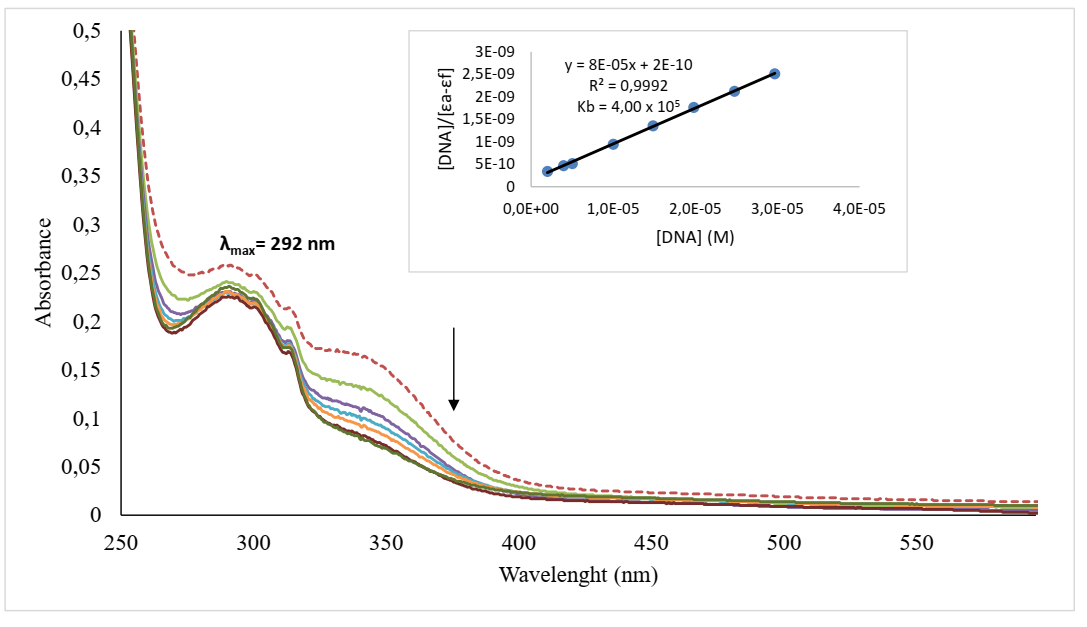


## Figure S9: Electronic Absorption Spectra of **Q4** at 5.0 x 10^5^ M in the absence (dashed line) and the presence of different concentrations of CT-DNA (0 – 3.0 x 10^5^ M) at 292 nm λ_max_. (inset) A stern-Volmer plot of **Q4** interaction with CT- DNA


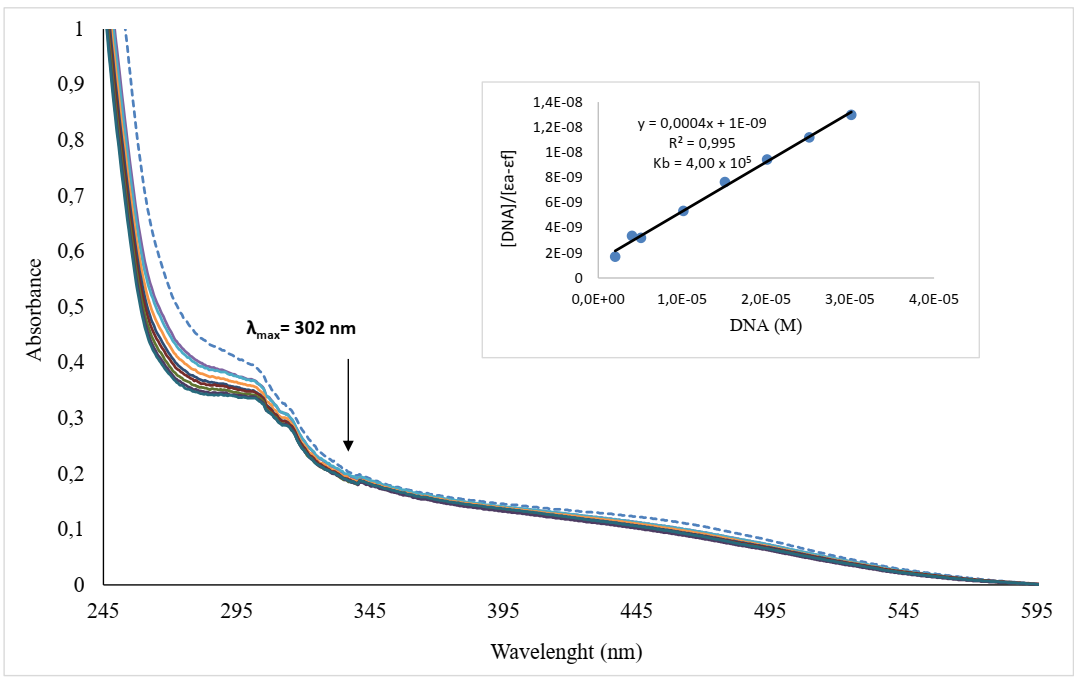


## Figure S10: Electronic Absorption Spectra of **Q5** at 5.0 x 10^5^ M in the absence (dashed line) and the presence of different concentrations of CT-DNA (0 – 3.0 x 10^5^ M) at 302 nm λ_max_. (inset) A stern-Volmer plot of **Q5** interaction with CT- DNA


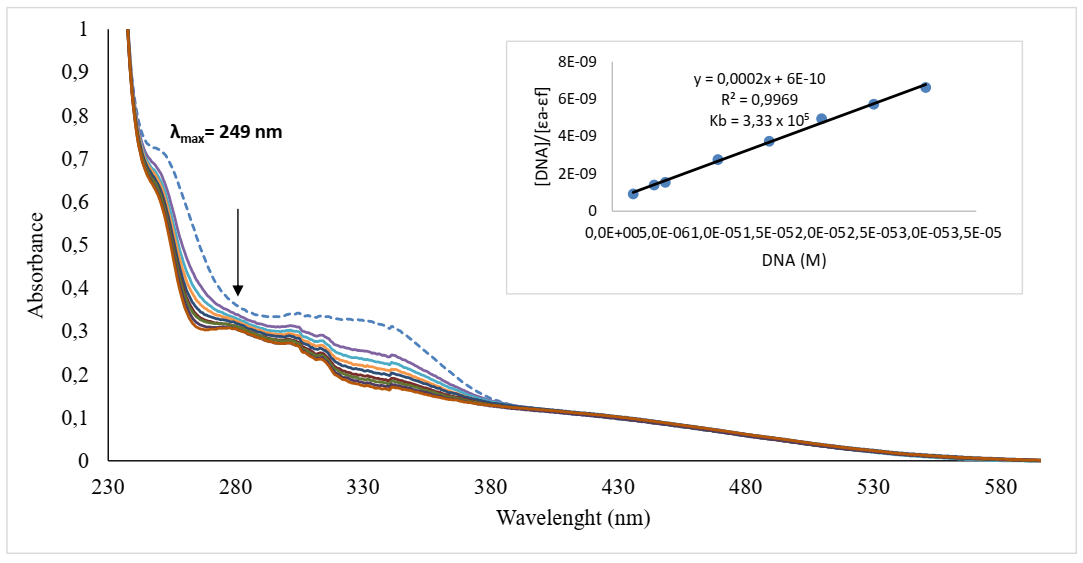


## Figure S11: Electronic Absorption Spectra of **Q6** at 5.0 x 10^5^ M in the absence (dashed line) and the presence of different concentrations of CT-DNA (0 – 3.0 x 10^5^ M) at 249 nm λ_max_. (inset) A stern-Volmer plot of **Q6** interaction with CT- DNA


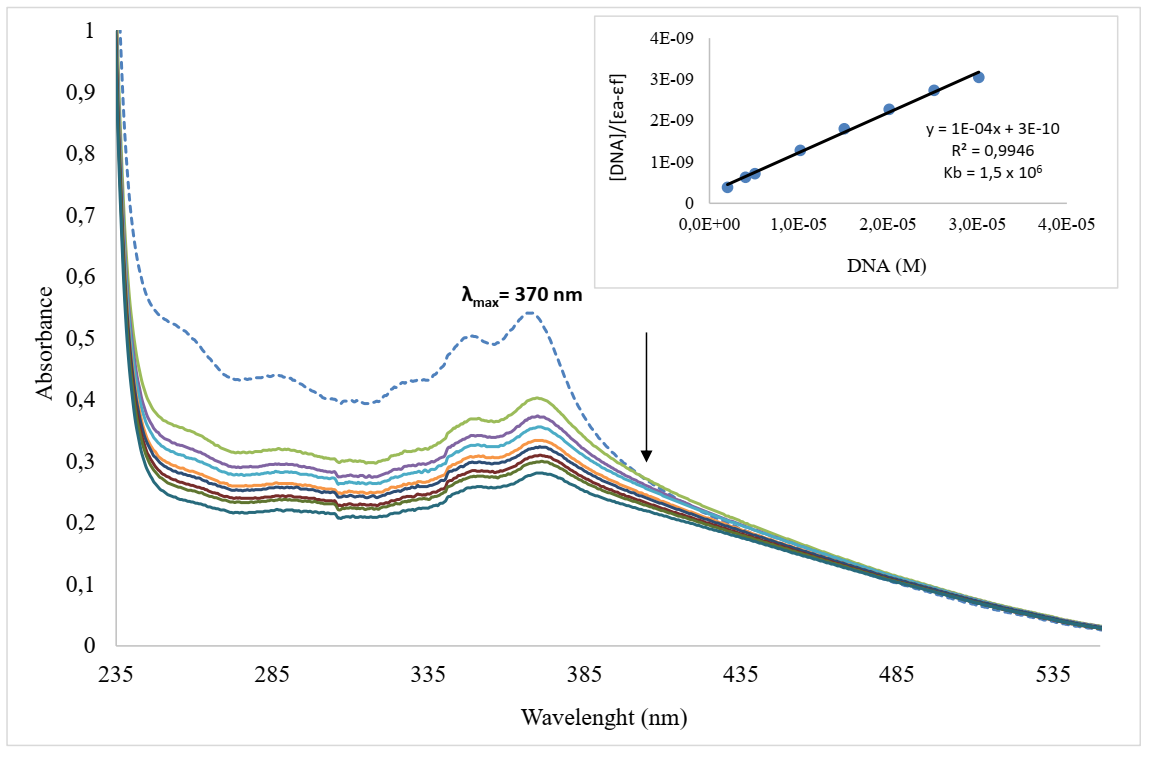


## Figure S12: Electronic Absorption Spectra of **Q7** at 5.0 x 10^5^ M in the absence (dashed line) and the presence of different concentrations of CT-DNA (0 – 3.0 x 10^5^ M) at 370 nm λ_max_. (inset) A stern-Volmer plot of **Q7** interaction with CT- DNA


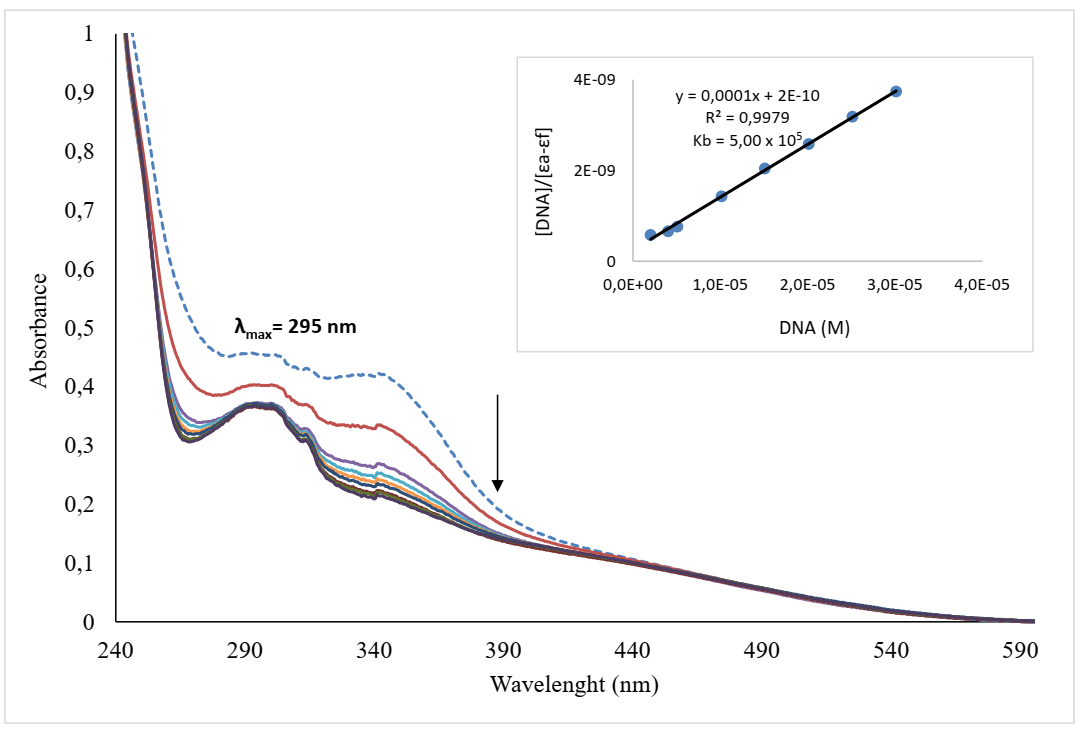


## Figure S13: Electronic Absorption Spectra of **Q9** at 5.0 x 10^5^ M in the absence (dashed line) and the presence of different concentrations of CT-DNA (0 – 3.0 x 10^5^ M) at 295 nm λ_max_. (inset) A stern-Volmer plot of **Q9** interaction with CT- DNA


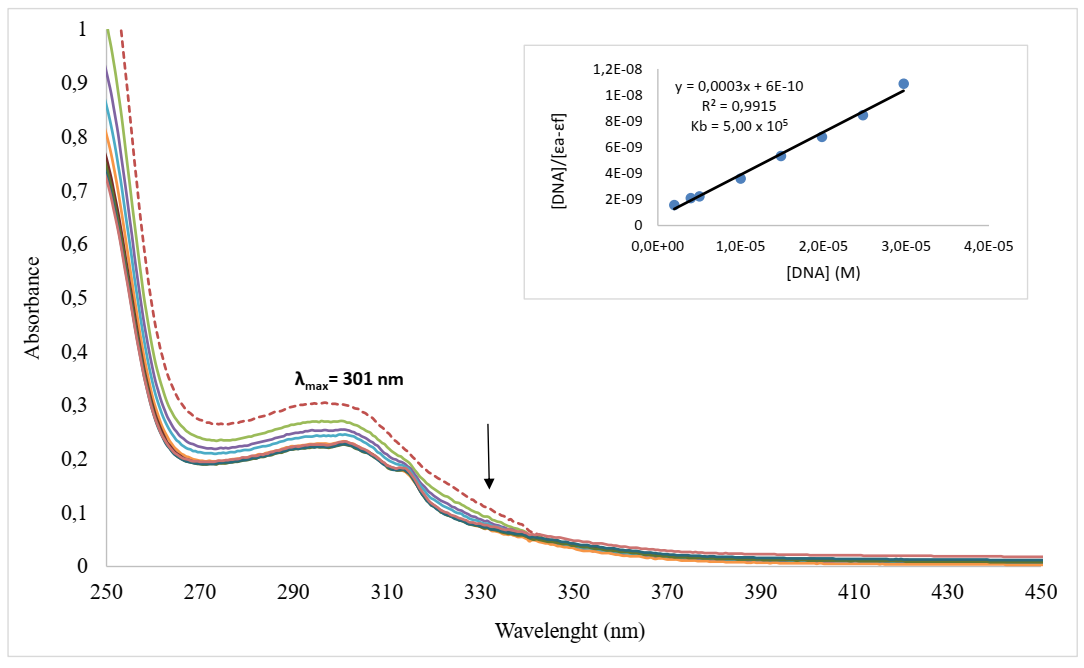


## Figure S14: Electronic Absorption Spectra of **Q10** at 5.0 x 10^5^ M in the absence (dashed line) and the presence of different concentrations of CT-DNA (0 – 3.0 x 10^5^ M) at 301 nm λ_max_. (inset) A stern-Volmer plot of **Q10** interaction with CT- DNA


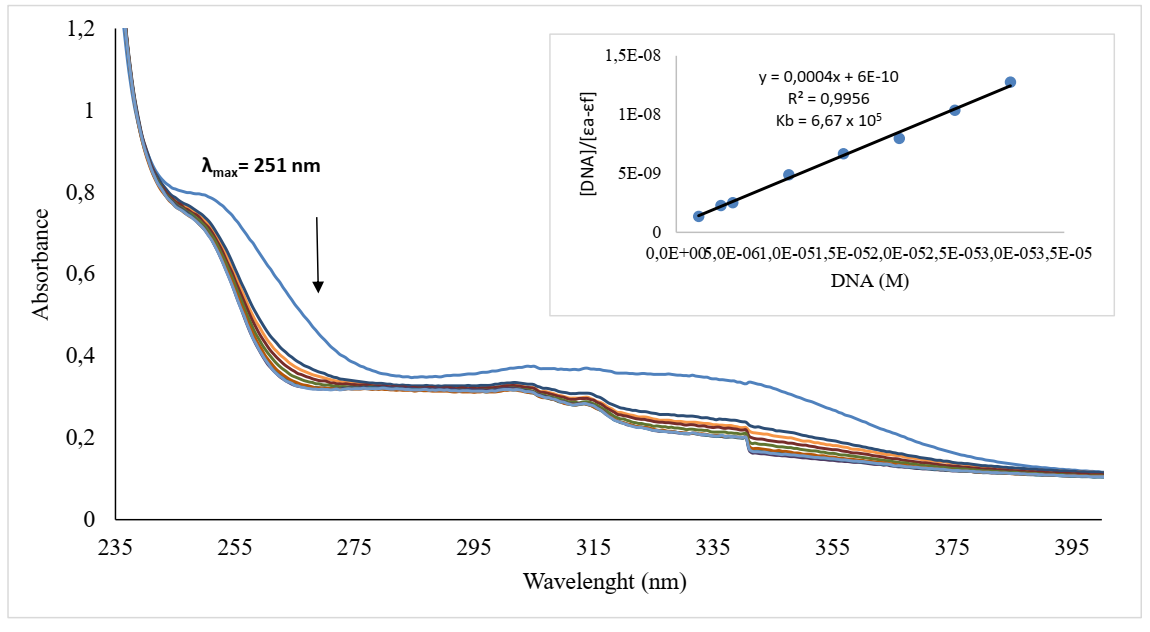


## Figure S15: Electronic Absorption Spectra of **Q11** at 5.0 x 10^5^ M in the absence (dashed line) and the presence of different concentrations of CT-DNA (0 – 3.0 x 10^5^ M) at 251 nm λ_max_. (inset) A stern-Volmer plot of **Q11** interaction with CT- DNA


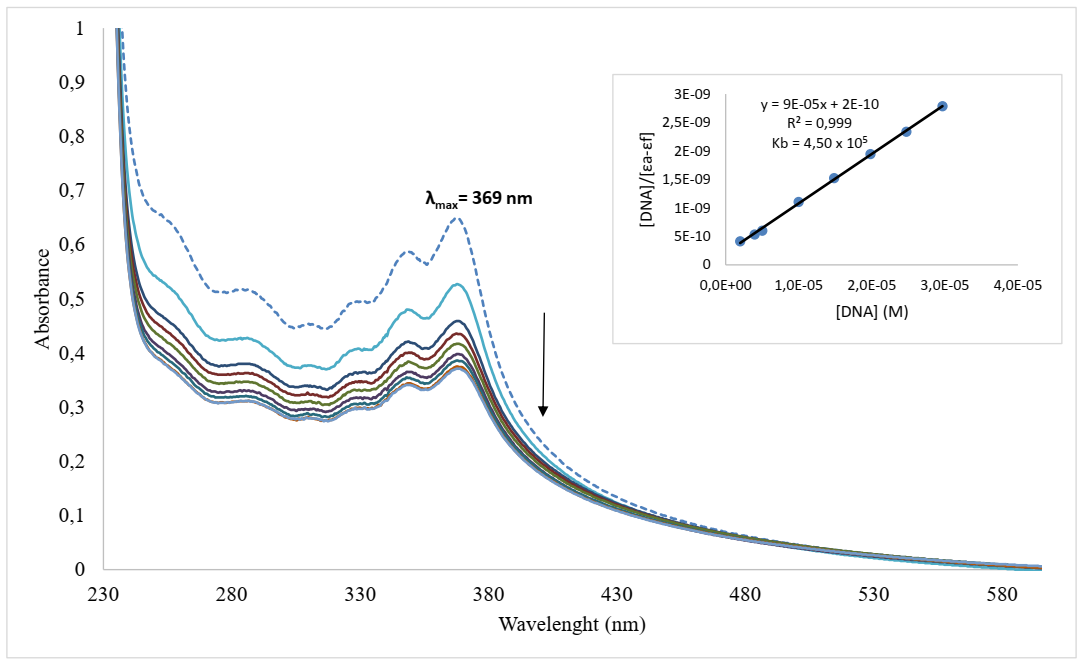


## Figure S16: Electronic Absorption Spectra of **Q12** at 5.0 x 10^5^ M in the absence (dashed line) and the presence of different concentrations of CT-DNA (0 – 3.0 x 10^5^ M) at 369 nm λ_max_. (inset) A stern-Volmer plot of **Q12** interaction with CT- DNA


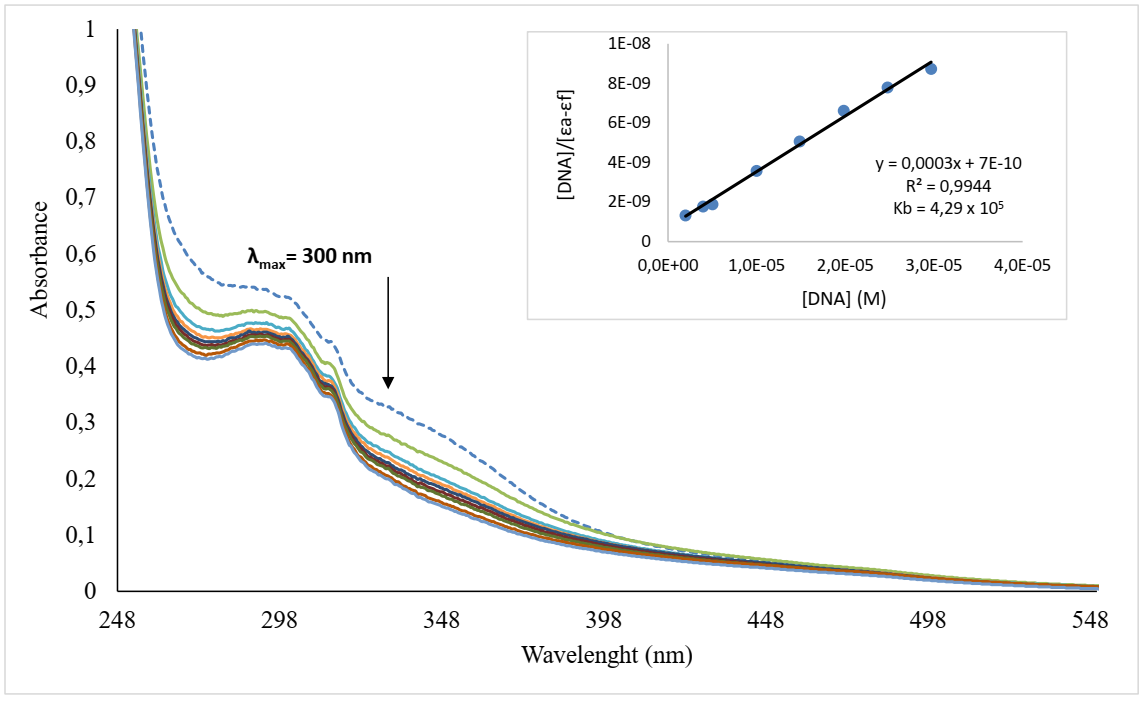


## Figure S17: Electronic Absorption Spectra of **Q13** at 5.0 x 10^5^ M in the absence (dashed line) and the presence of different concentrations of CT-DNA (0 – 3.0 x 10^5^ M) at 300 nm λ_max_. (inset) A stern-Volmer plot of **Q13** interaction with CT- DNA


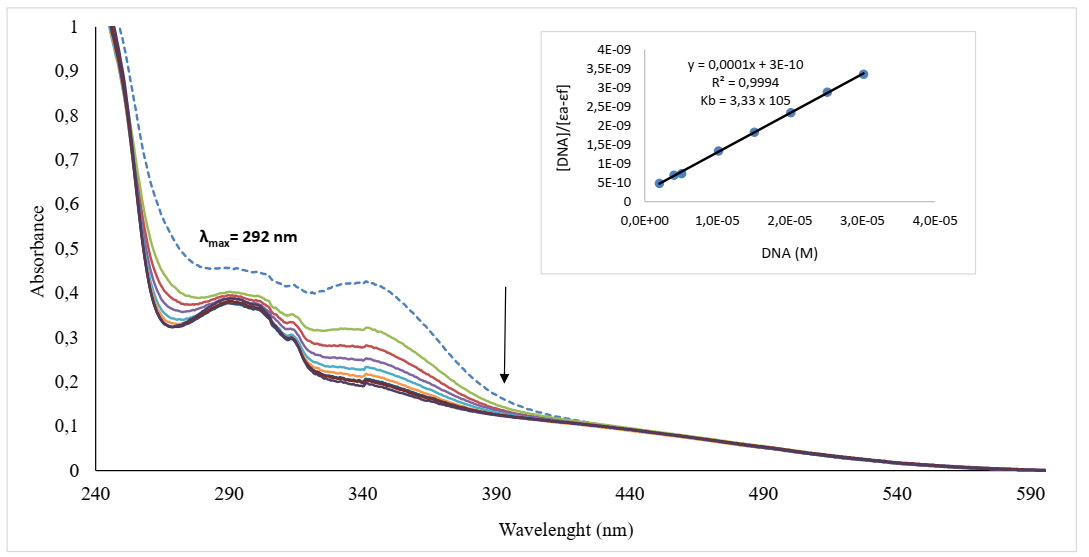


## Figure S18: Electronic Absorption Spectra of **Q14** at 5.0 x 10^5^ M in the absence (dashed line) and the presence of different concentrations of CT-DNA (0 – 3.0 x 10^5^ M) at 292 nm λ_max_. (inset) A stern-Volmer plot of **Q14** interaction with CT- DNA


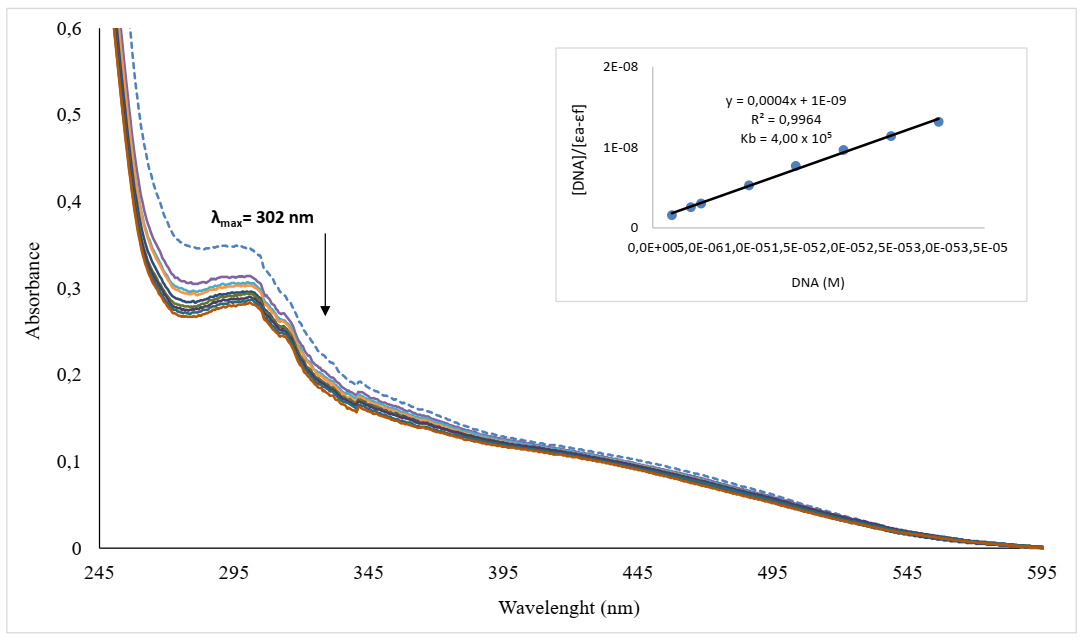


## **Figure S19**: Electronic Absorption Spectra of **Q15** at 5.0 X 10^5^ M in the absence (dashed line) and the presence of different concentrations of CT-DNA (0–3.0 X 10^5^ M) at 302 nm λ_max_. (inset) A stern-Volmer plot of **Q15** interaction with CT- DNA


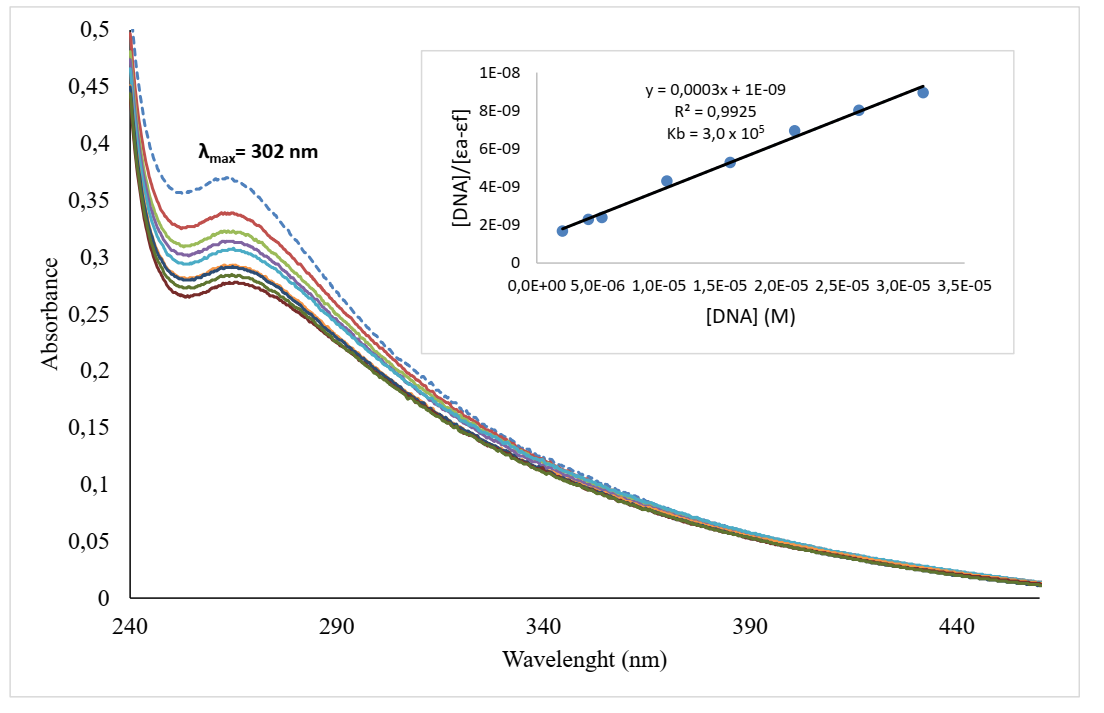


## **Figure S20:** Electronic Absorption Spectra of **silver nitrate** at 5.0 x 10^5^ M in the absence (dashed line) and the presence of different concentrations of CT-DNA (0 – 3.0 x 10^5^ M) at 302 nm λ_max_. (inset) A stern-Volmer plot of **silver nitrate** interaction with CT- DNA


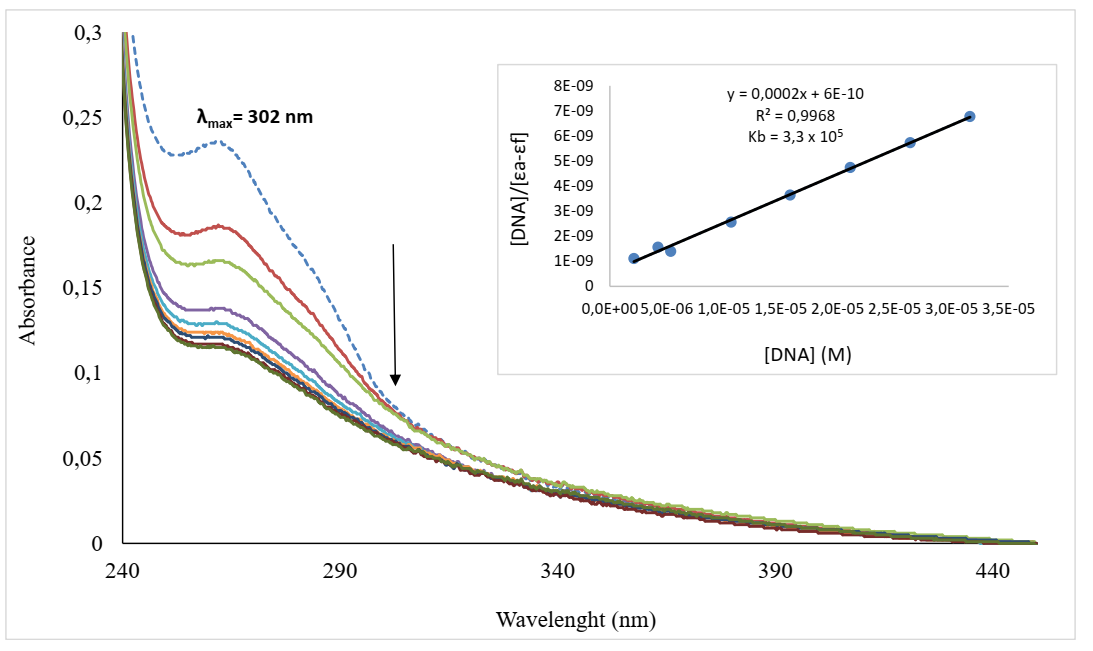


## **Figure S21:** Electronic Absorption Spectra of **silver perchlorate** at 5.0 x 10^5^ M in the absence (dashed line) and the presence of different concentrations of CT-DNA (0 – 3.0 x 10^5^ M) at 302 nm λ_max_. (inset) A stern-Volmer plot of **silver perchlorate** interaction with CT- DNA


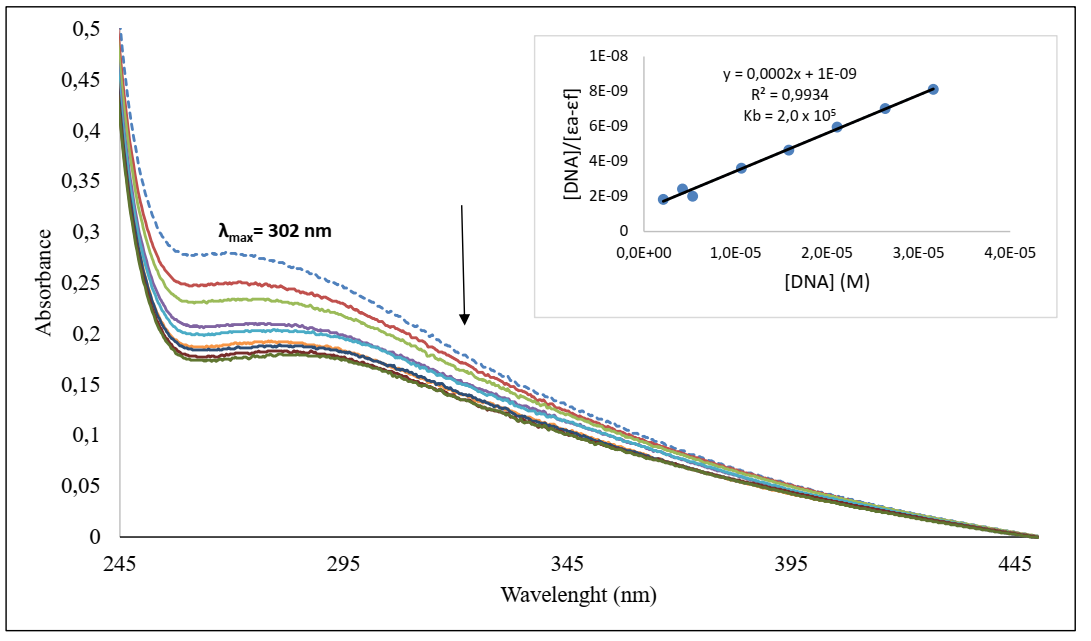


## Figure S22: Electronic Absorption Spectra of **silver triflate** at 5.0 x 10^5^ M in the absence (dashed line) and the presence of different concentrations of CT-DNA (0 – 3.0 x 10^5^ M) at 302 nm λ_max_. (inset) A stern-Volmer plot of **silver triflate** interaction with CT- DNA


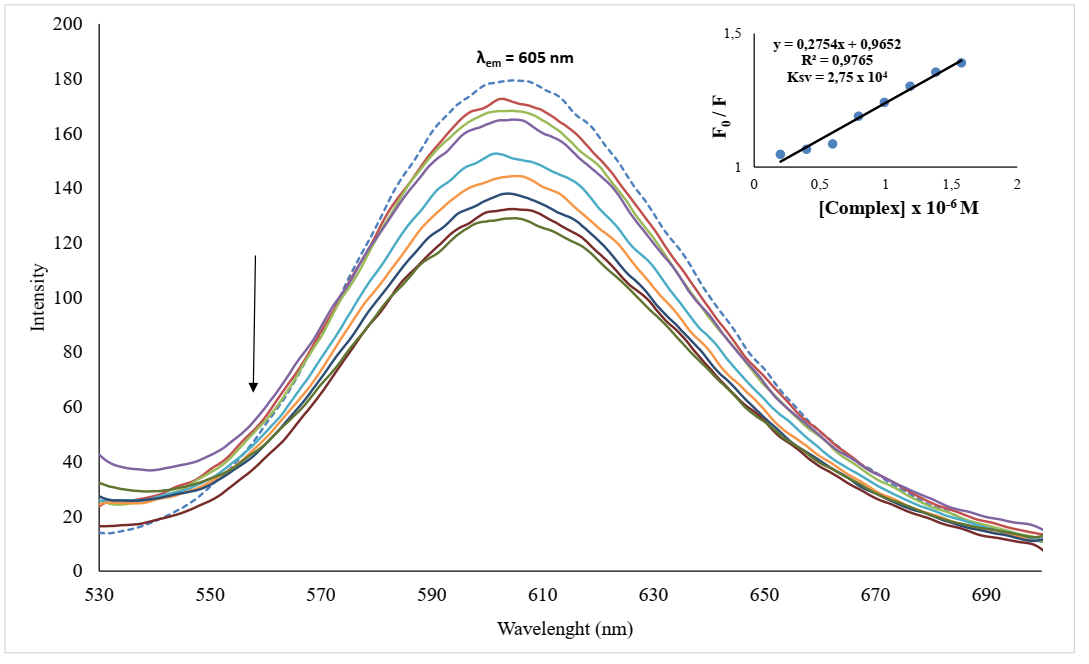


## Figure S23: The Fluorescence spectra of EB-CT-DNA in the absence (dashed line) and the presence of different concentration of complex **Q1**. (inset) Stern-Volmer plot of **Q1** interaction with EB-CT- DNA


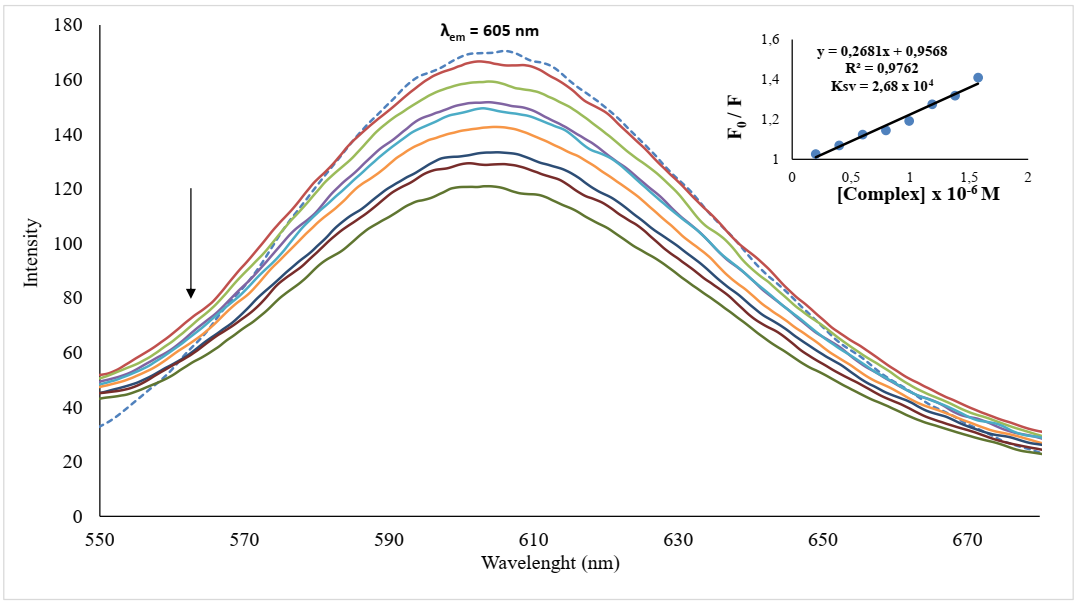


## Figure S24: The Fluorescence spectra of EB-CT-DNA in the absence (dashed line) and the presence of different concentration of complex **Q2**. (inset) Stern-Volmer plot of **Q2** interaction with EB-CT- DNA


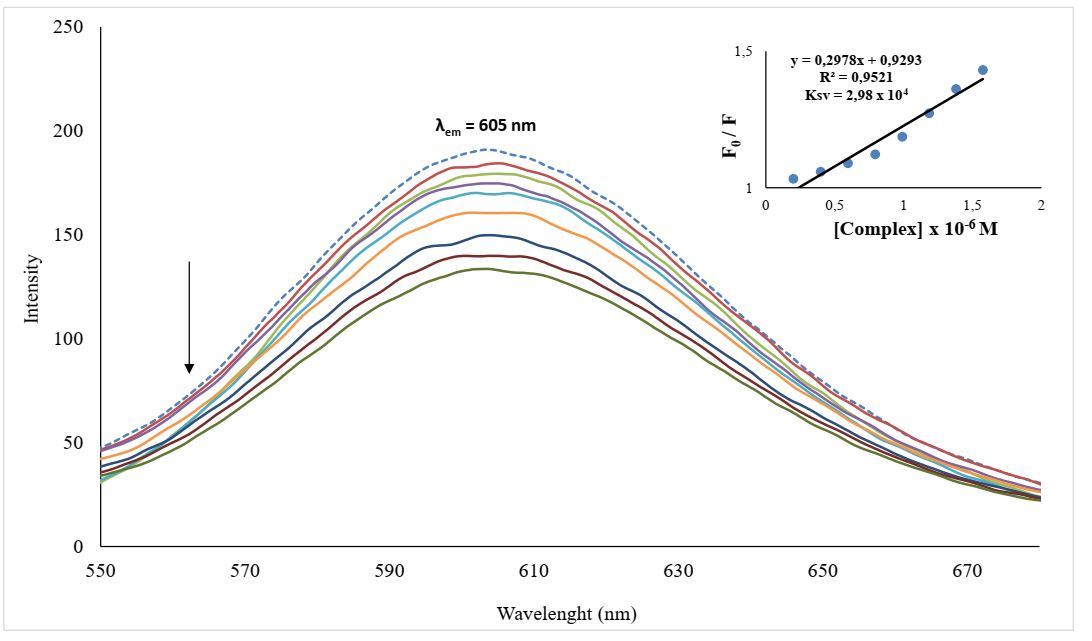


## Figure S25: The Fluorescence spectra of EB-CT-DNA in the absence (dashed line) and the presence of different concentration of complex **Q3**. (inset) Stern-Volmer plot of **Q3** interaction with EB-CT- DNA


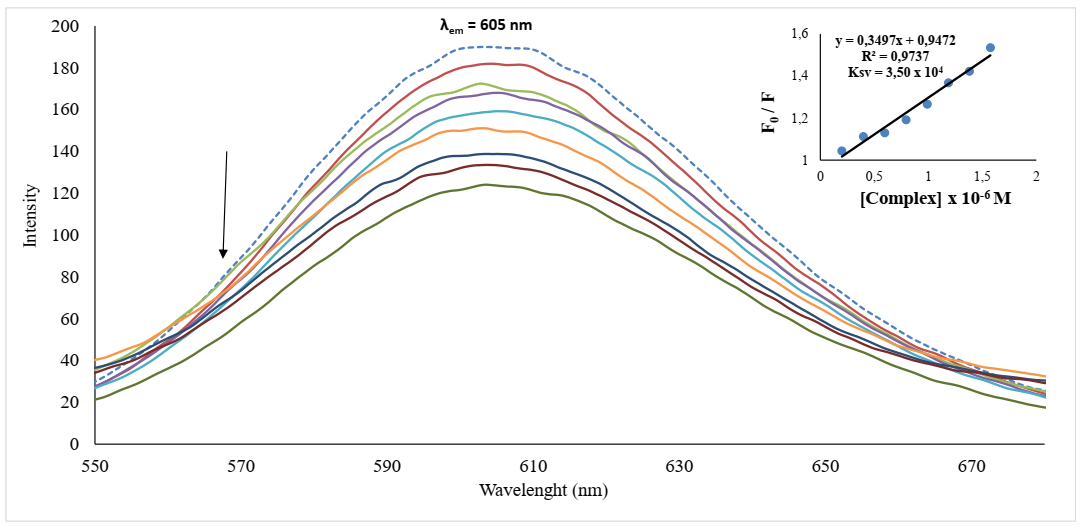


## Figure S26: The Fluorescence spectra of EB-CT-DNA in the absence (dashed line) and the presence of different concentration of complex **Q4**. (inset) Stern-Volmer plot of **Q4** interaction with EB-CT- DNA


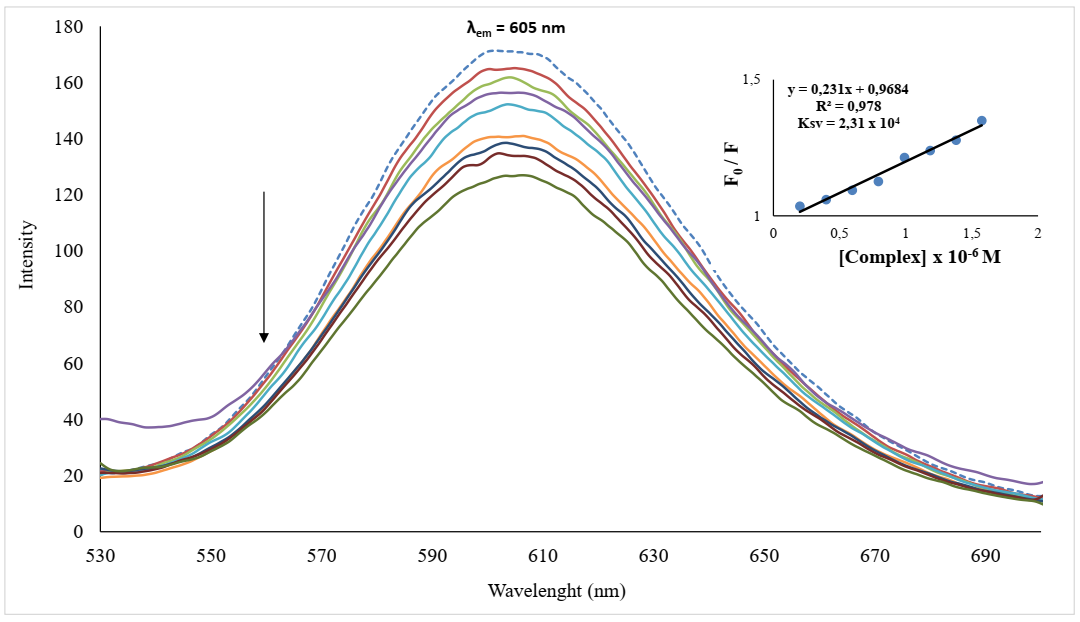


## Figure S27: The Fluorescence spectra of EB-CT-DNA in the absence (dashed line) and the presence of different concentration of complex **Q5**. (inset) Stern-Volmer plot of **Q5** interaction with EB-CT- DNA


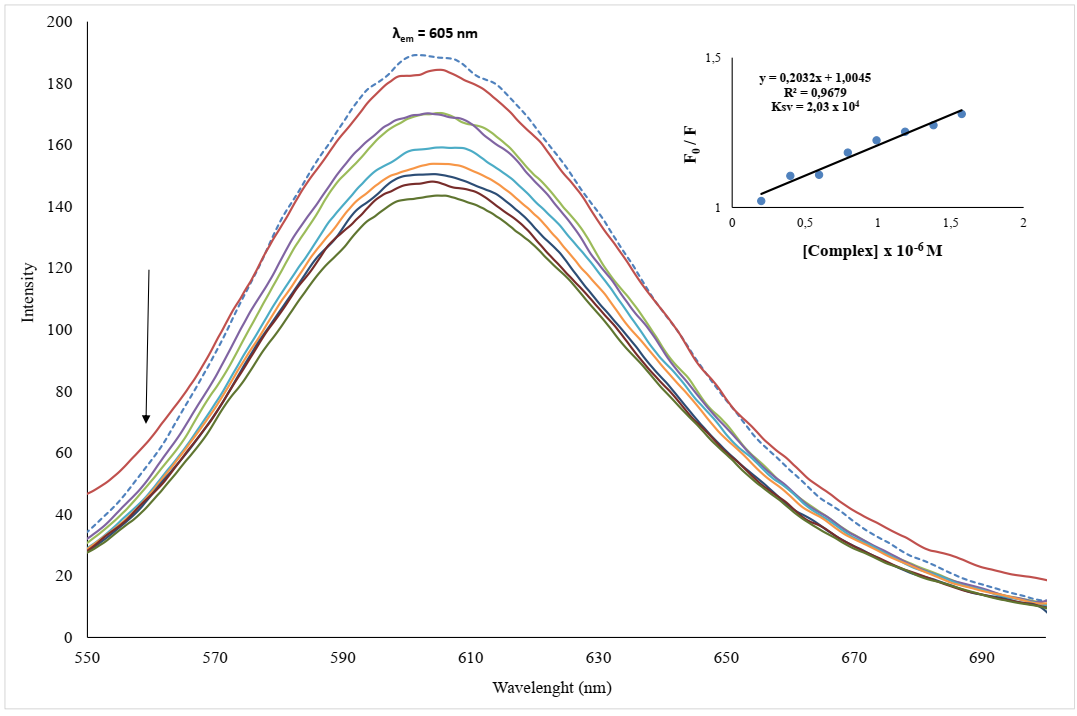


## Figure S28: The Fluorescence spectra of EB-CT-DNA in the absence (dashed line) and the presence of different concentration of complex **Q6**. (inset) Stern-Volmer plot of **Q6** interaction with EB-CT- DNA


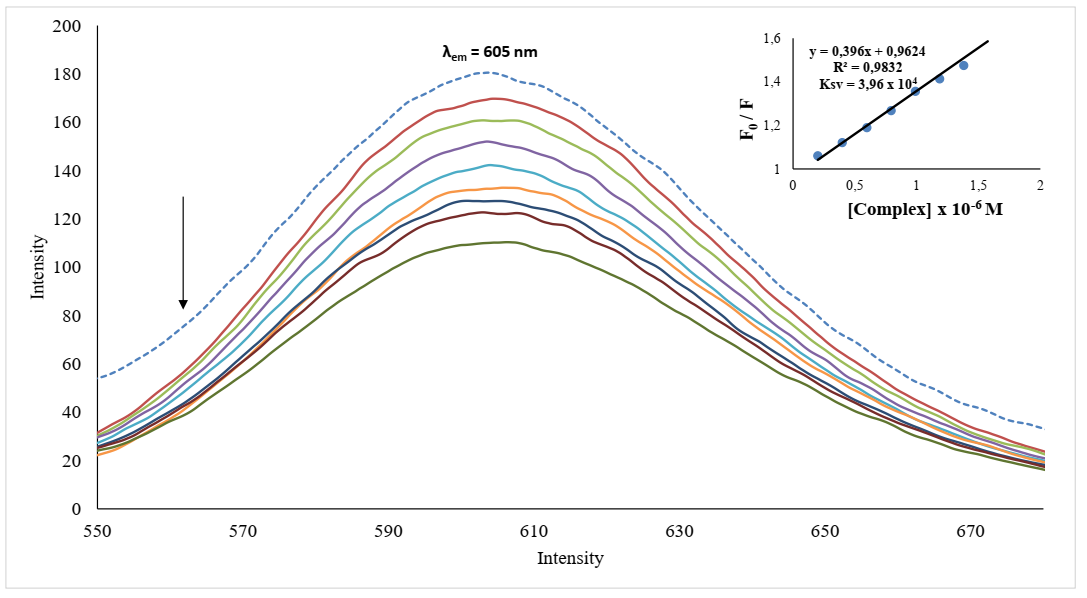


## Figure S29: The Fluorescence spectra of EB-CT-DNA in the absence (dashed line) and the presence of different concentration of complex **Q7**. (inset) Stern-Volmer plot of **Q7** interaction with EB-CT- DNA


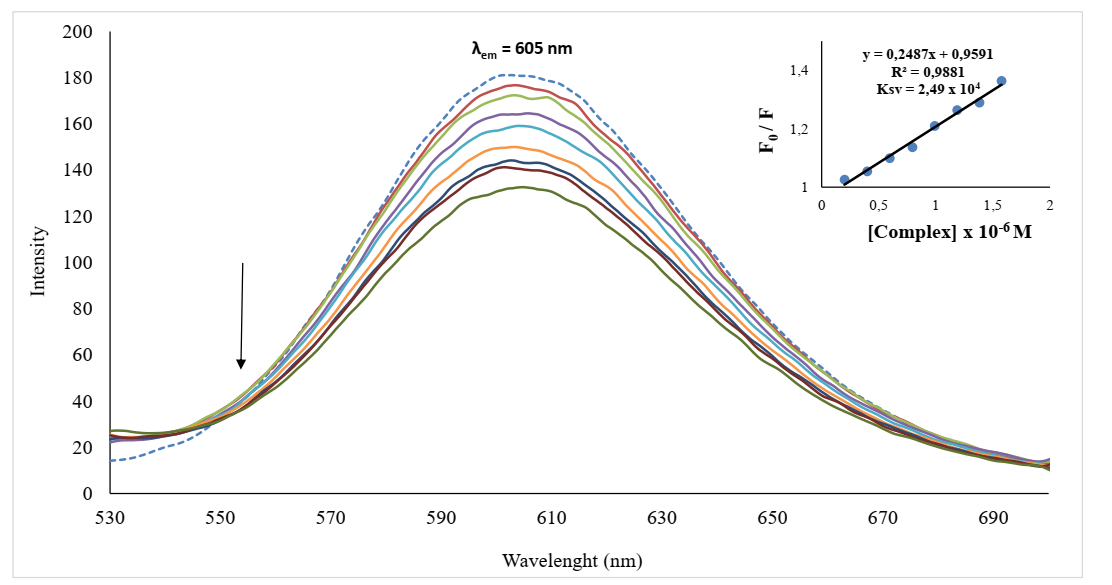


## Figure S30: The Fluorescence spectra of EB-CT-DNA in the absence (dashed line) and the presence of different concentration of complex **Q9**. (inset) Stern-Volmer plot of **Q9** interaction with EB-CT- DNA


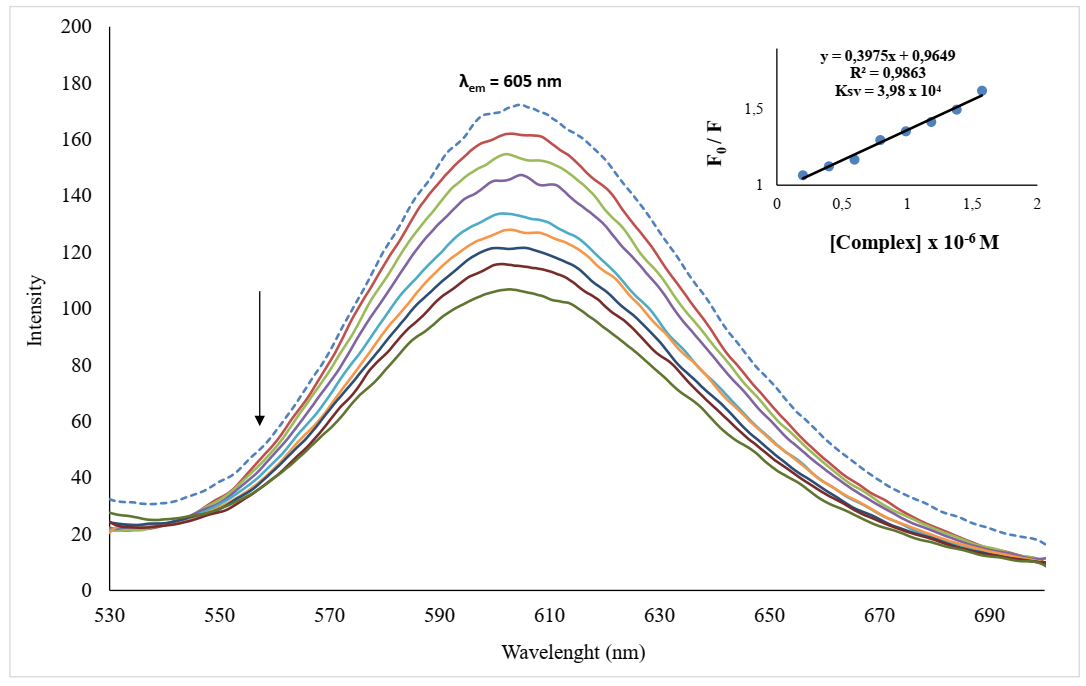


## Figure S31: The Fluorescence spectra of EB-CT-DNA in the absence (dashed line) and the presence of different concentration of complex **Q10**. (inset) Stern-Volmer plot of **Q10** interaction with EB-CT- DNA


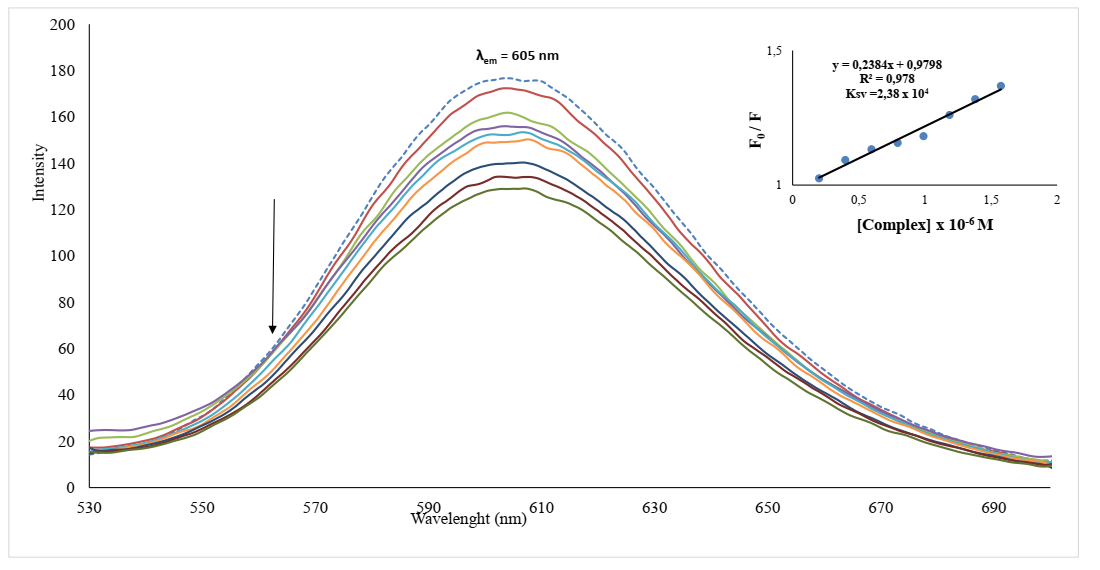


## Figure S32: The Fluorescence spectra of EB-CT-DNA in the absence (dashed line) and the presence of different concentration of complex **Q11**. (inset) Stern-Volmer plot of **Q11** interaction with EB-CT- DNA


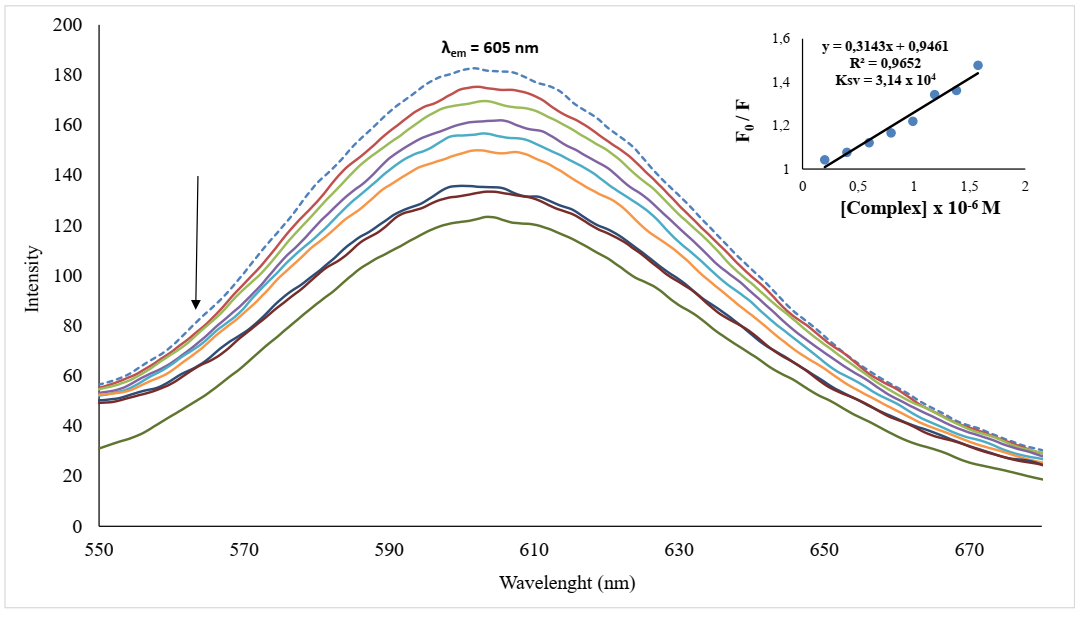


## Figure S33: The Fluorescence spectra of EB-CT-DNA in the absence (dashed line) and the presence of different concentration of complex **Q12**. (inset) Stern-Volmer plot of **Q12** interaction with EB-CT- DNA


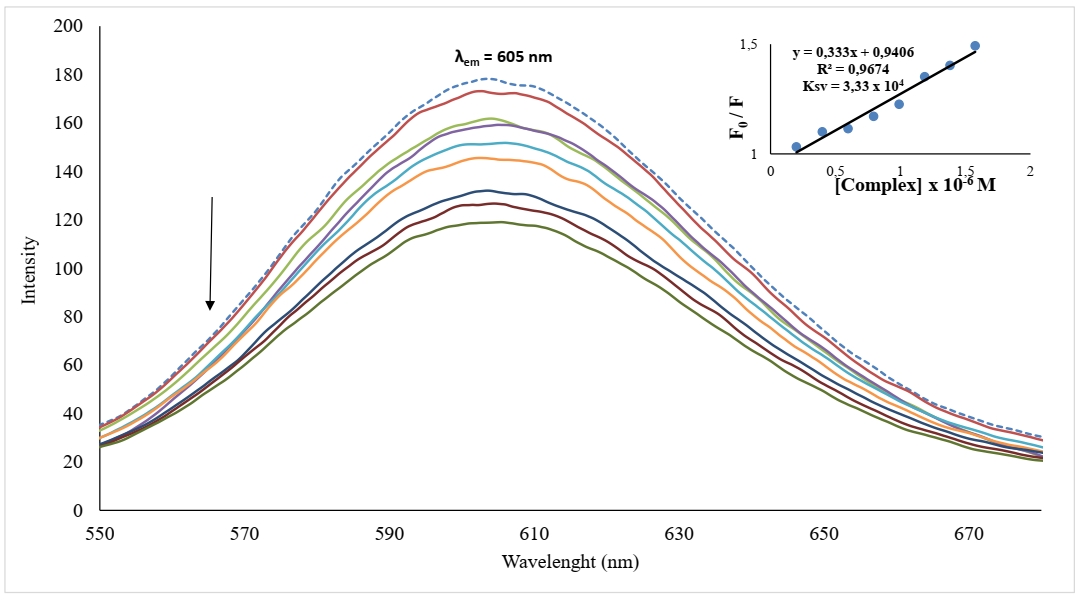


## Figure S34: The Fluorescence spectra of EB-CT-DNA in the absence (dashed line) and the presence of different concentration of complex **Q13**. (inset) Stern-Volmer plot of **Q13** interaction with EB-CT- DNA


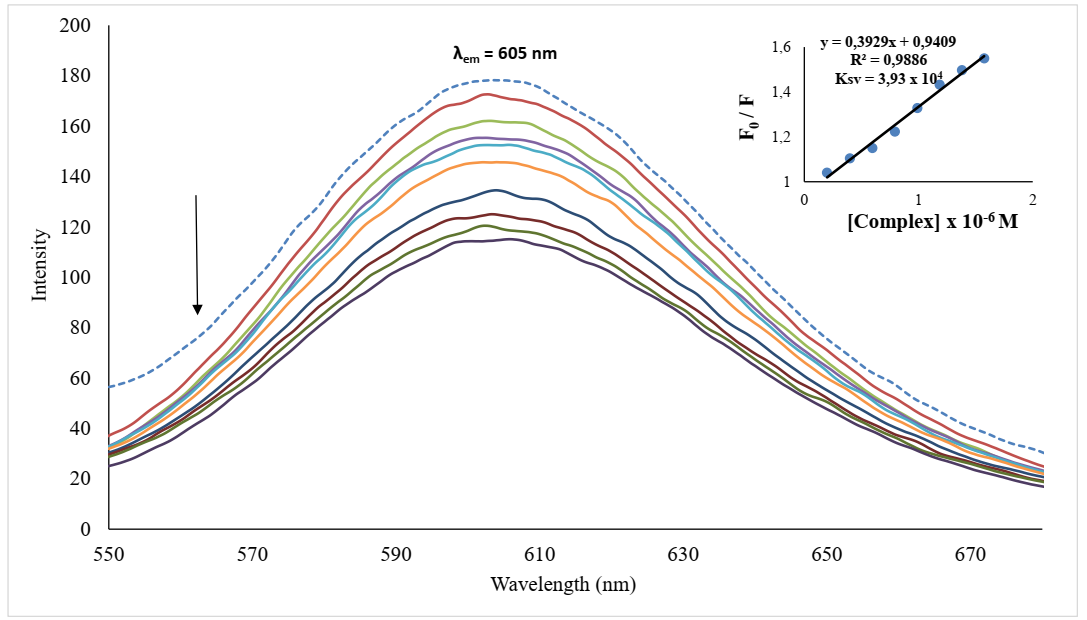


## Figure S35: The Fluorescence spectra of EB-CT-DNA in the absence (dashed line) and the presence of different concentration of complex **Q14**. (inset) Stern-Volmer plot of **Q14** interaction with EB-CT- DNA


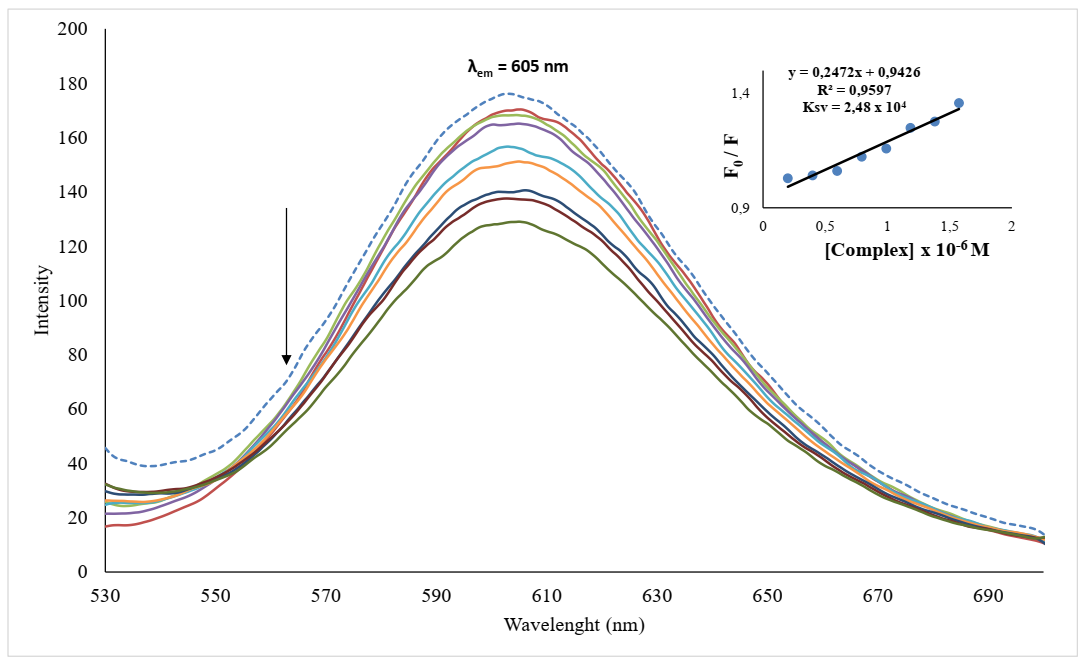


## Figure S36: The Fluorescence spectra of EB-CT-DNA in the absence (dashed line) and the presence of different concentration of complex **Q15**. (inset) Stern-Volmer plot of **Q15** interaction with EB-CT- DNA


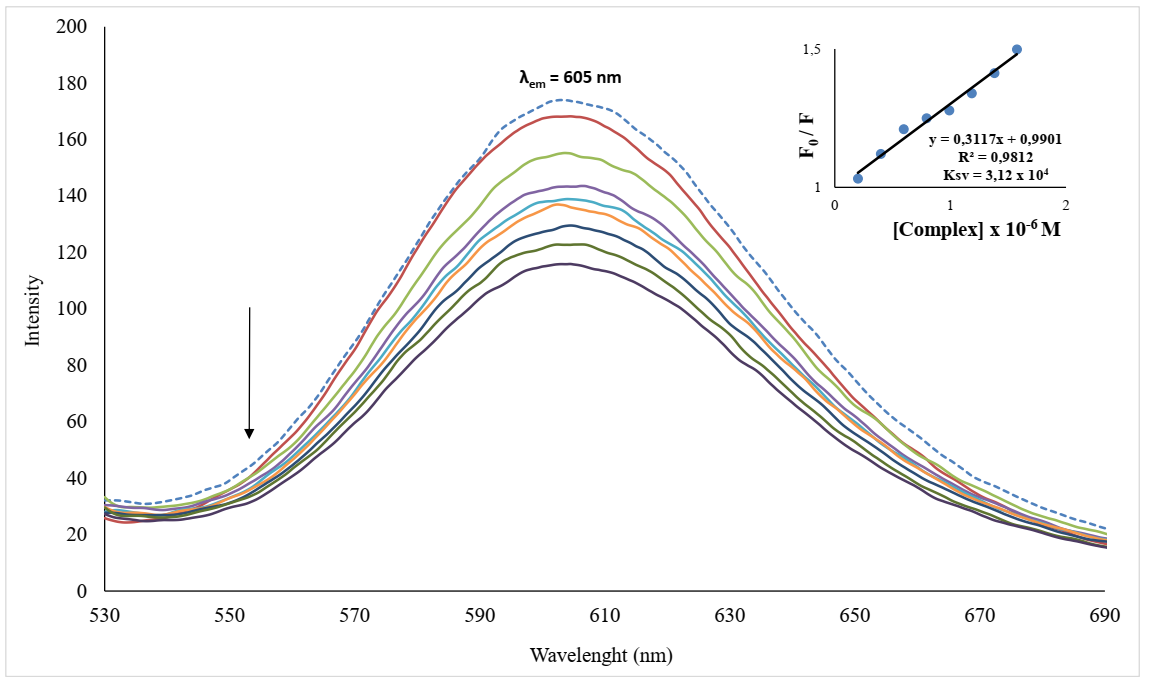


## Figure S37: The Fluorescence spectra of EB-CT-DNA in the absence (dashed line) and the presence of different concentration of **silver nitrate**. (inset) Stern-Volmer plot of **silver nitrate** interaction with EB-CT- DNA


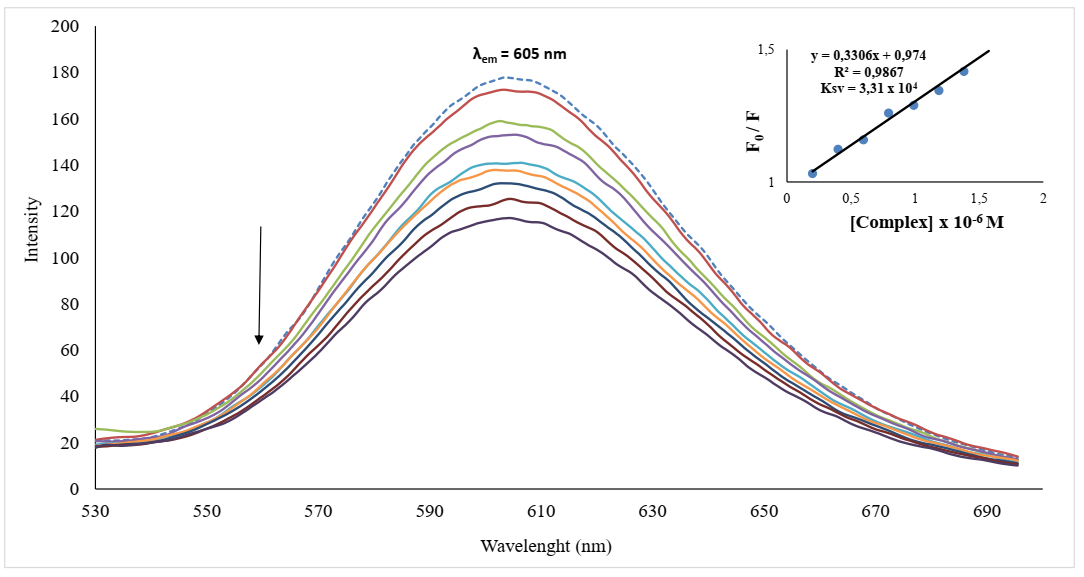


## Figure S38: The Fluorescence spectra of EB-CT-DNA in the absence (dashed line) and the presence of different concentration of **silver perchlorate**. (inset) Stern-Volmer plot of **silver perchlorate** interaction with EB-CT- DNA


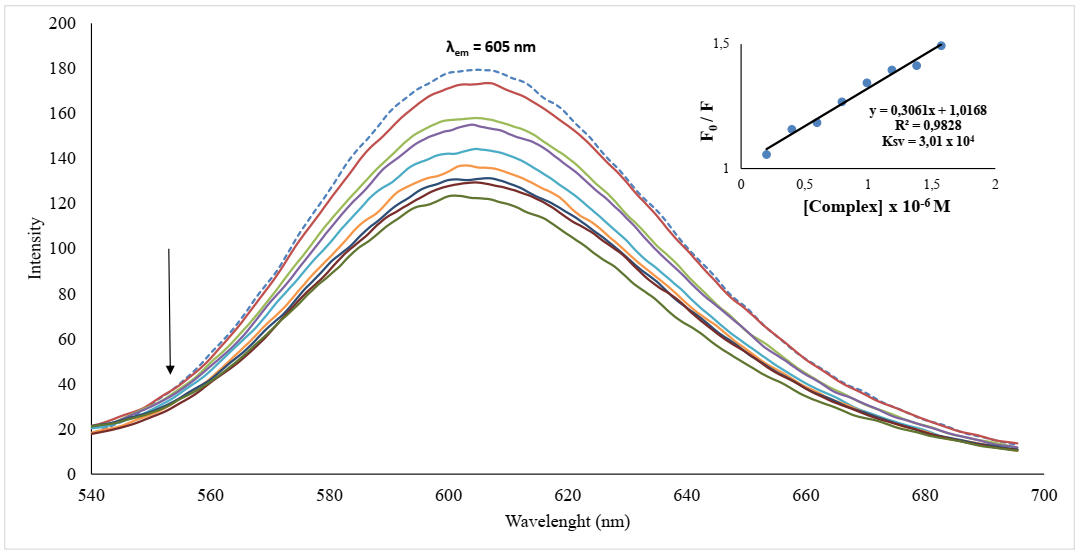


## Figure S39: The Fluorescence spectra of EB-CT-DNA in the absence (dashed line) and the presence of different concentration of **silver triflate**. (inset) Stern-Volmer plot of **silver triflate** interaction with EB-CT- DNA

# **The double-logarithmic plot of EB-CT-DNA–Complexes interactions at room temperature.**


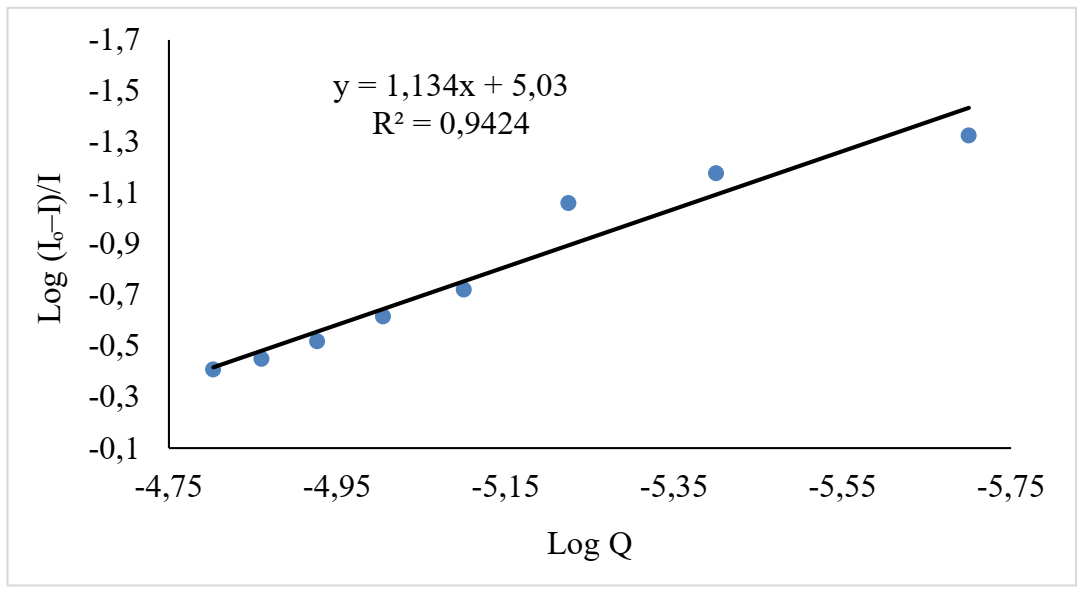


## Figure S40: The double-logarithmic plot of EB-CT-DNA–Complex **Q1** interaction at room temperature.


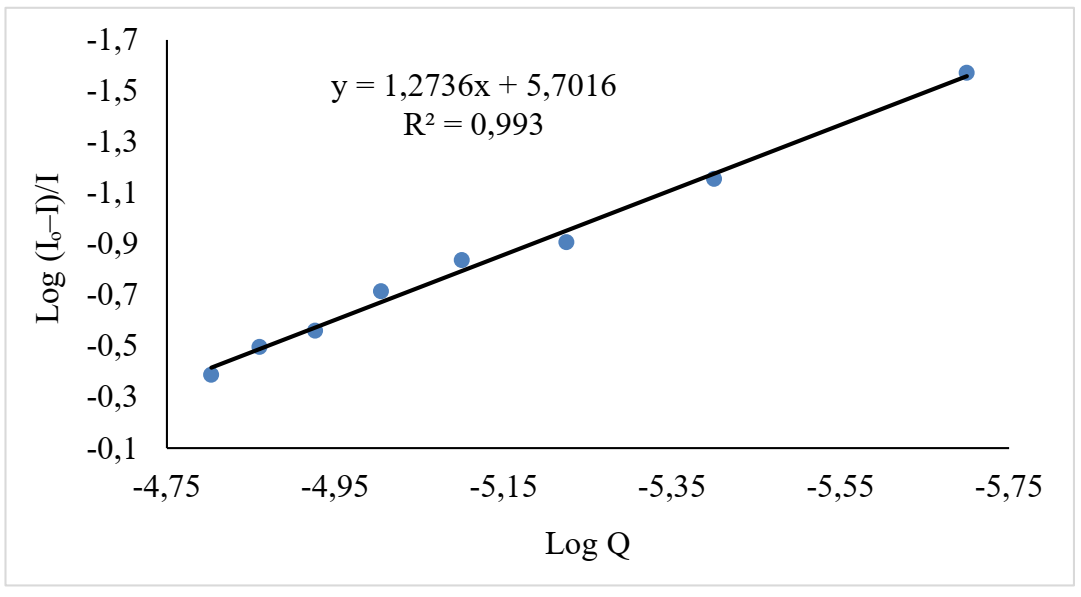


Figure S41: The double-logarithmic plot of EB-CT-DNA–Complex **Q2** interaction at room temperature.


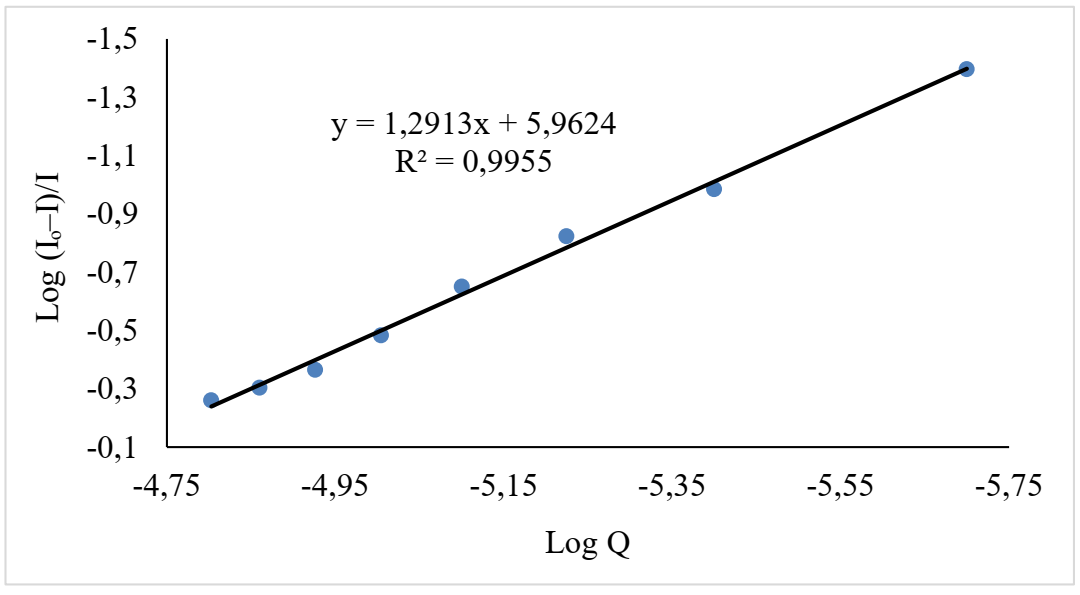


## Figure S42: The double-logarithmic plot of EB-CT-DNA–Complex **Q3** interaction at room temperature.


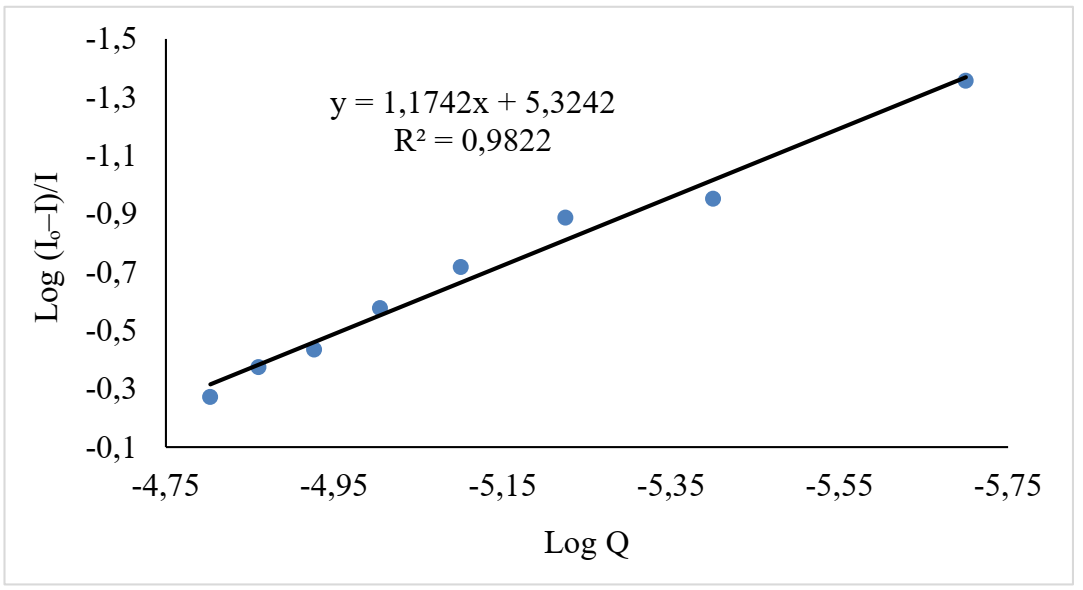
Figure S43: The double-logarithmic plot of EB-CT-DNA–Complex **Q4** interaction at room temperature.


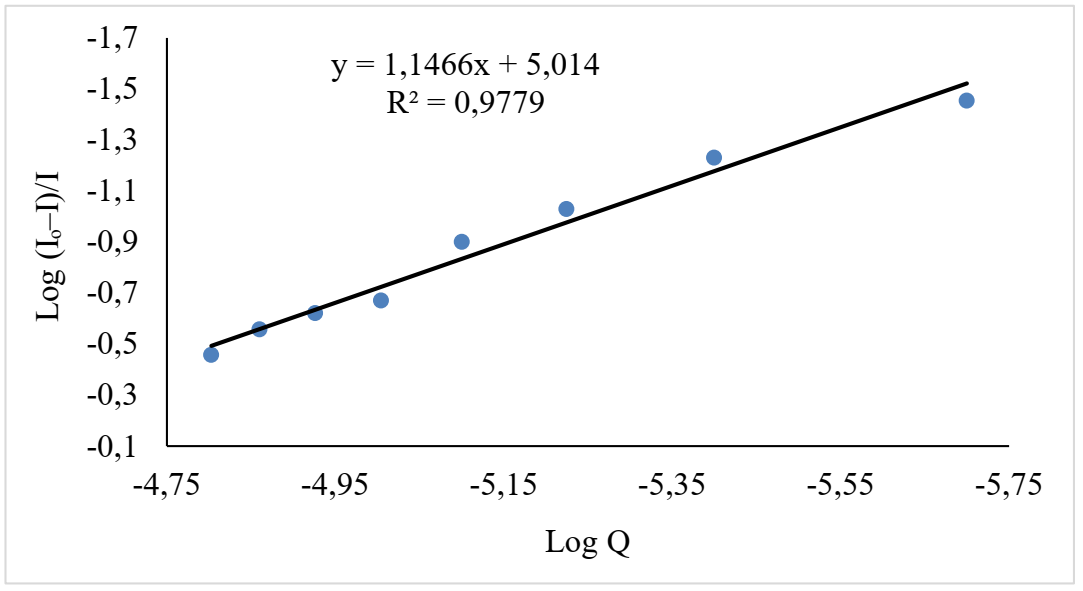


## Figure S44: The double-logarithmic plot of EB-CT-DNA–Complex **Q5** interaction at room temperature.


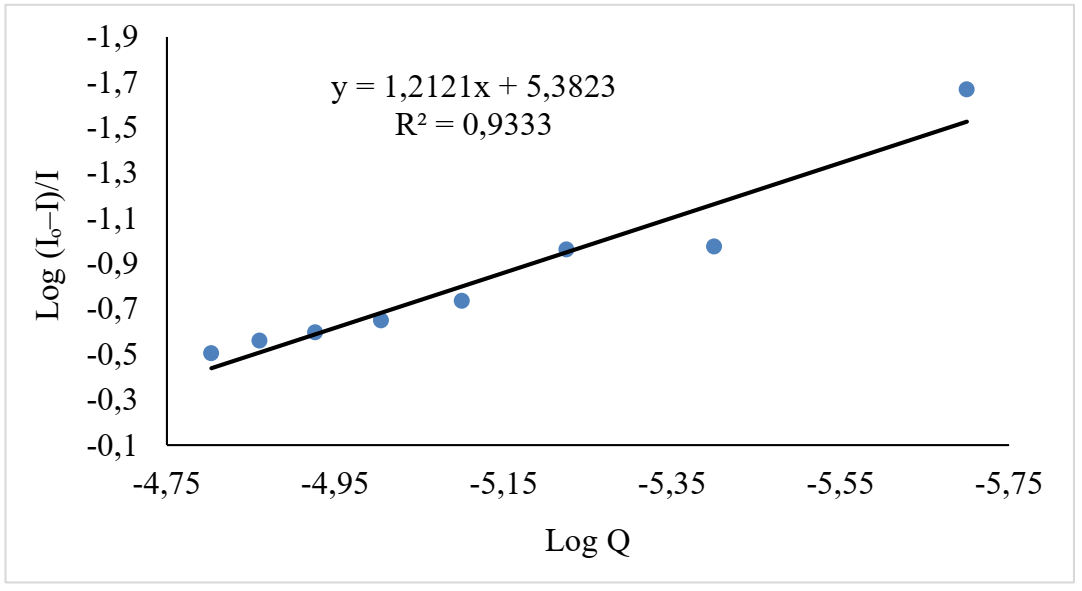
Figure S45: The double-logarithmic plot of EB-CT-DNA–Complex **Q6** interaction at room temperature.


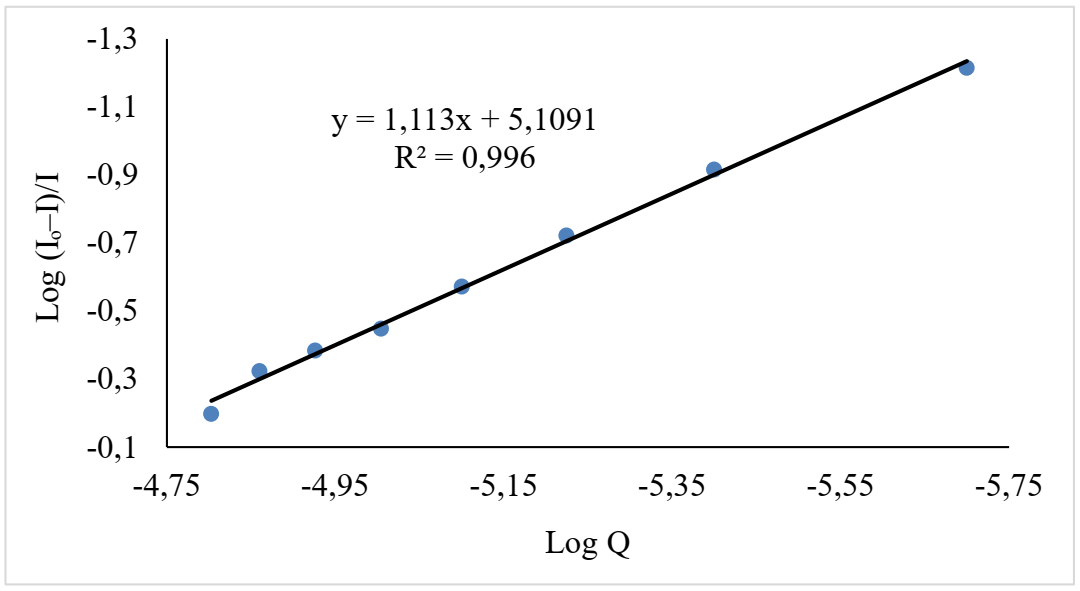


## Figure S46: The double-logarithmic plot of EB-CT-DNA–Complex **Q7** interaction at room temperature.


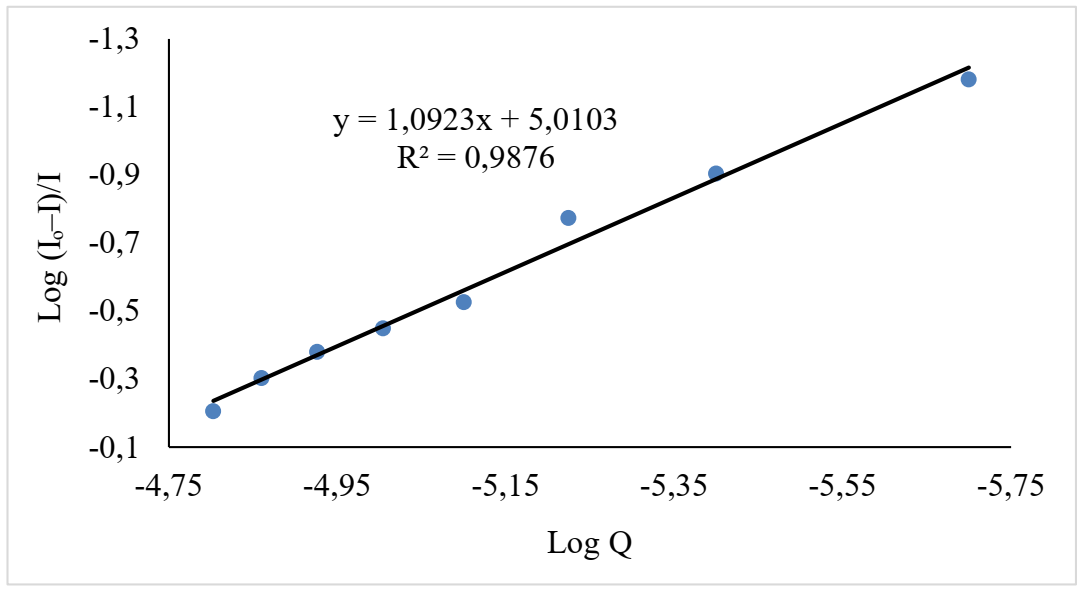
Figure S47: The double-logarithmic plot of EB-CT-DNA–Complex **Q8** interaction at room temperature.


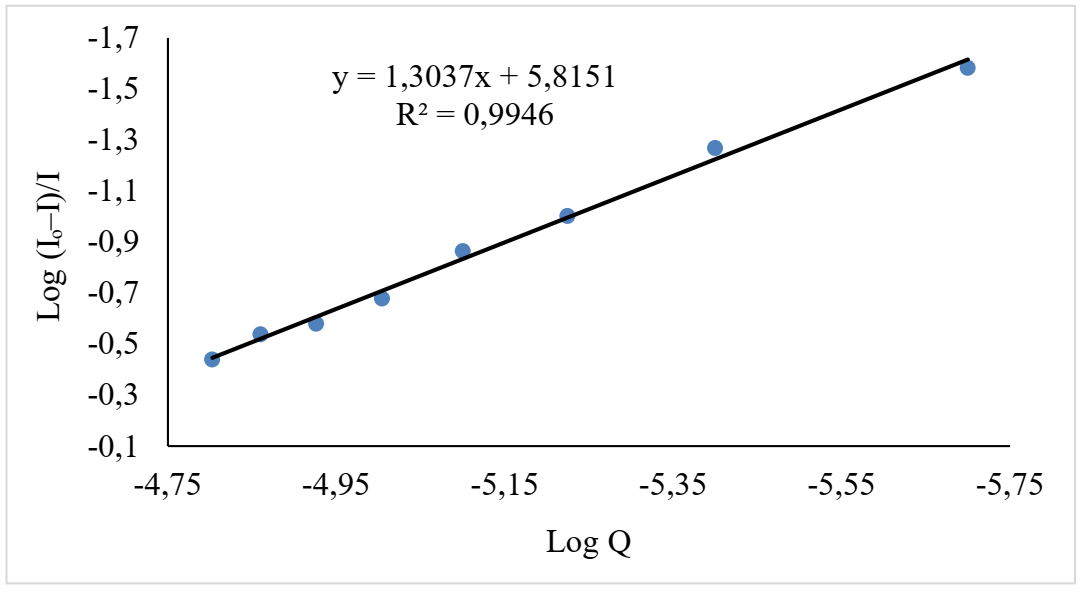


## Figure S48: The double-logarithmic plot of EB-CT-DNA–Complex **Q9** interaction at room temperature.


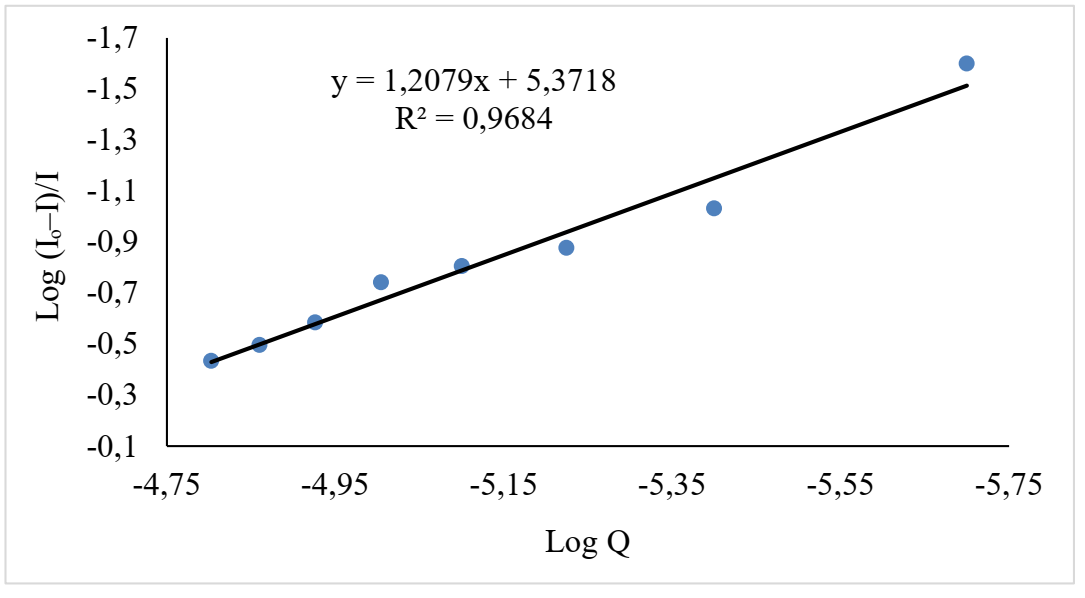
Figure S49: The double-logarithmic plot of EB-CT-DNA–Complex **Q11** interaction at room temperature.


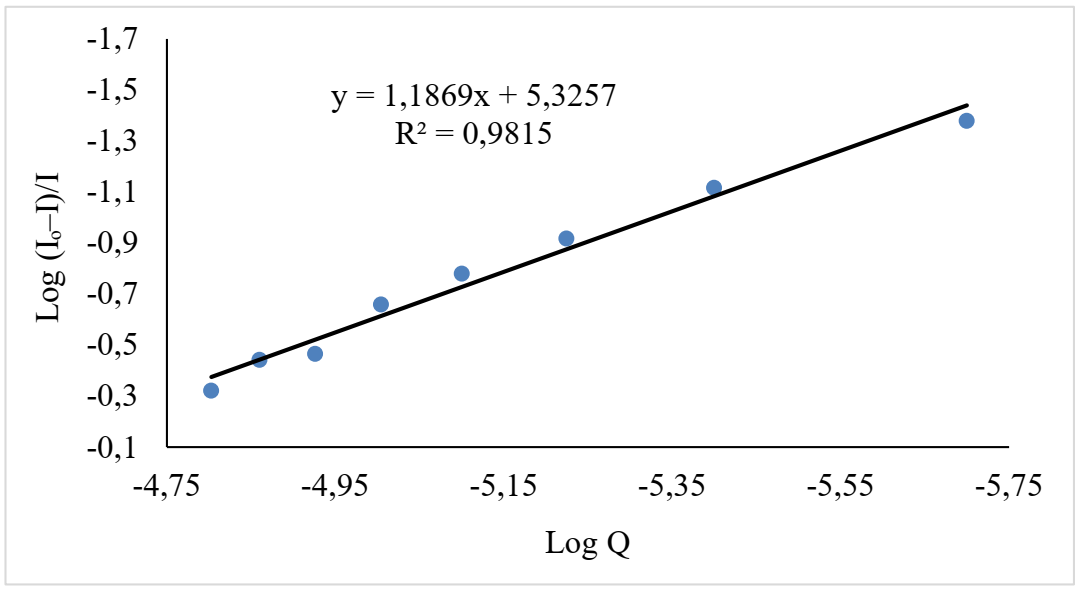


## Figure S50: The double-logarithmic plot of EB-CT-DNA–Complex **Q12** interaction at room temperature.


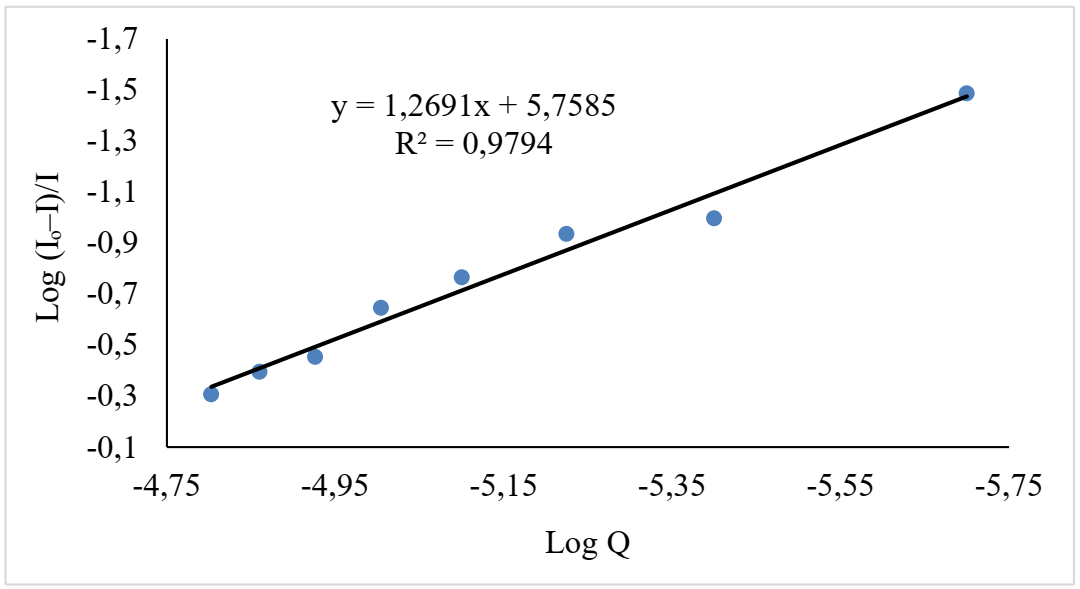
Figure S51: The double-logarithmic plot of EB-CT-DNA–Complex **Q13** interaction at room temperature.


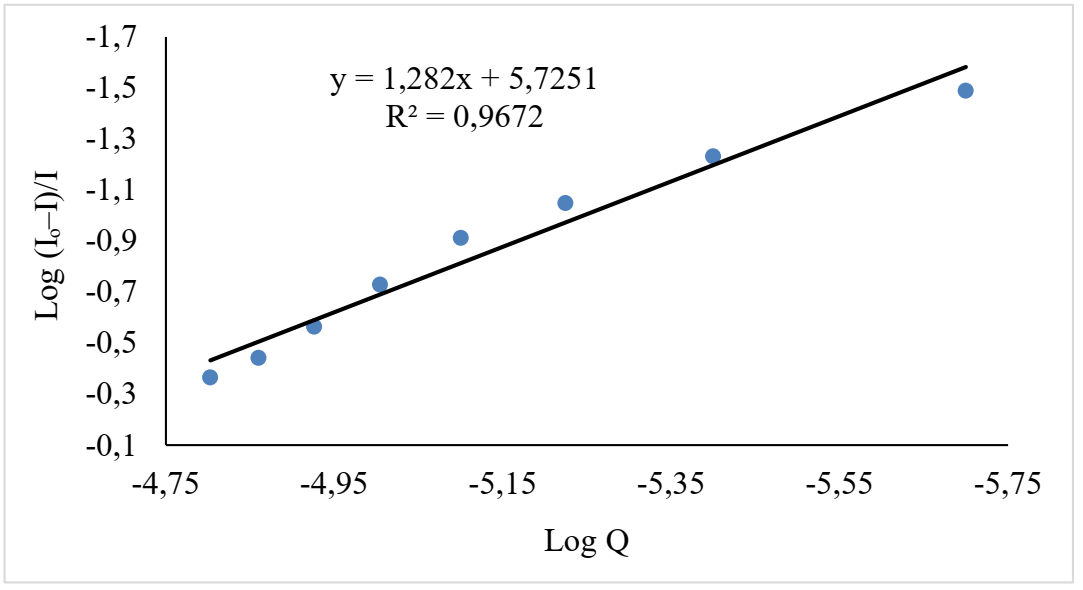
Figure S52: The double-logarithmic plot of EB-CT-DNA–Complex **Q14** interaction at room temperature.


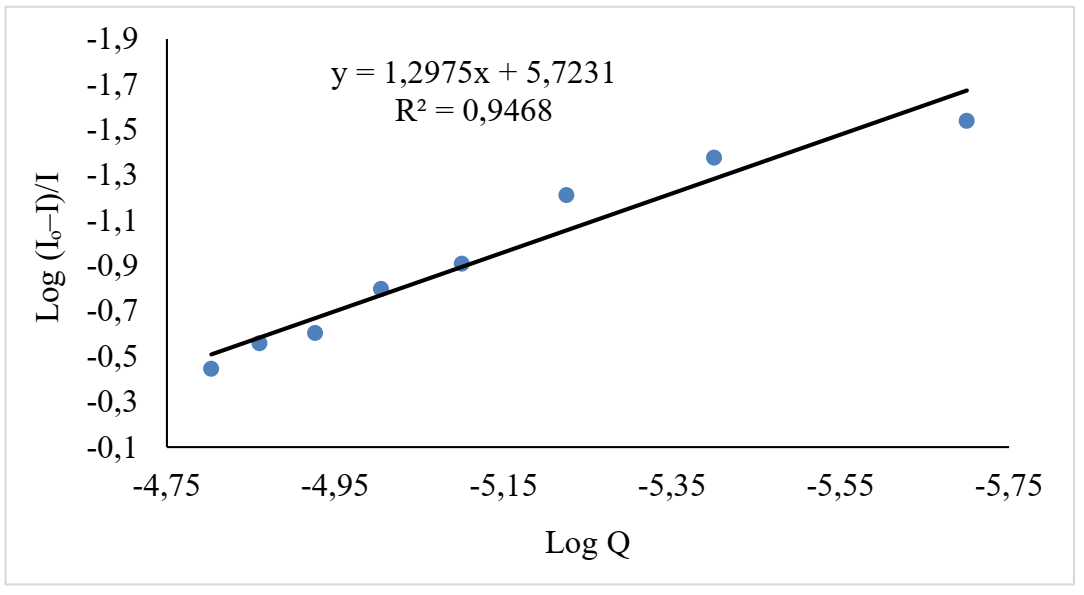
Figure S53: The double-logarithmic plot of EB-CT-DNA–Complex **Q15** interaction at room temperature.


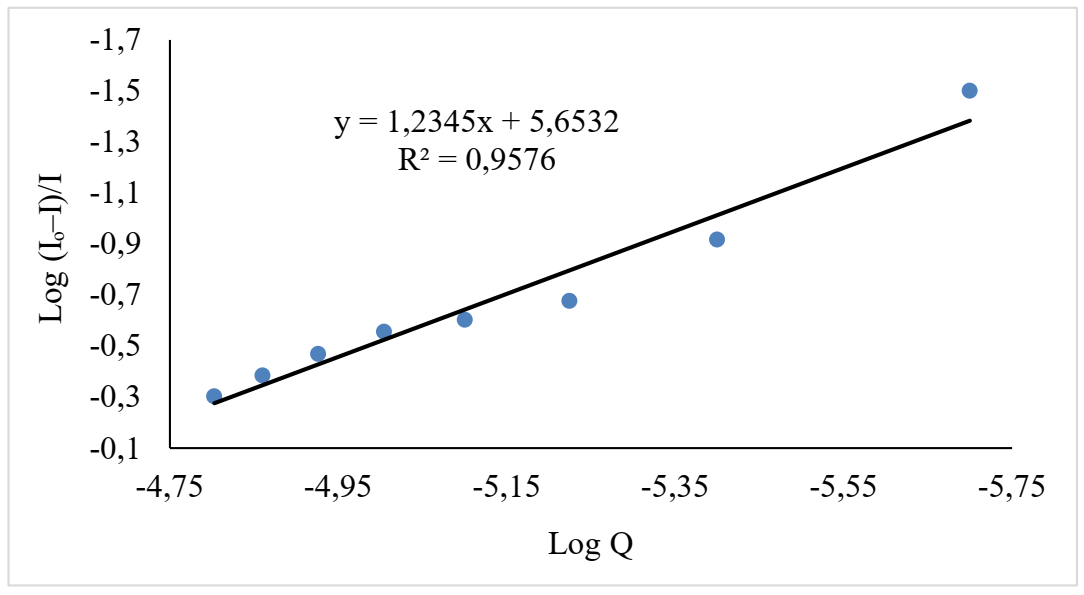


## Figure S54: The double-logarithmic plot of EB-CT-DNA–silver nitrate interaction at room temperature.


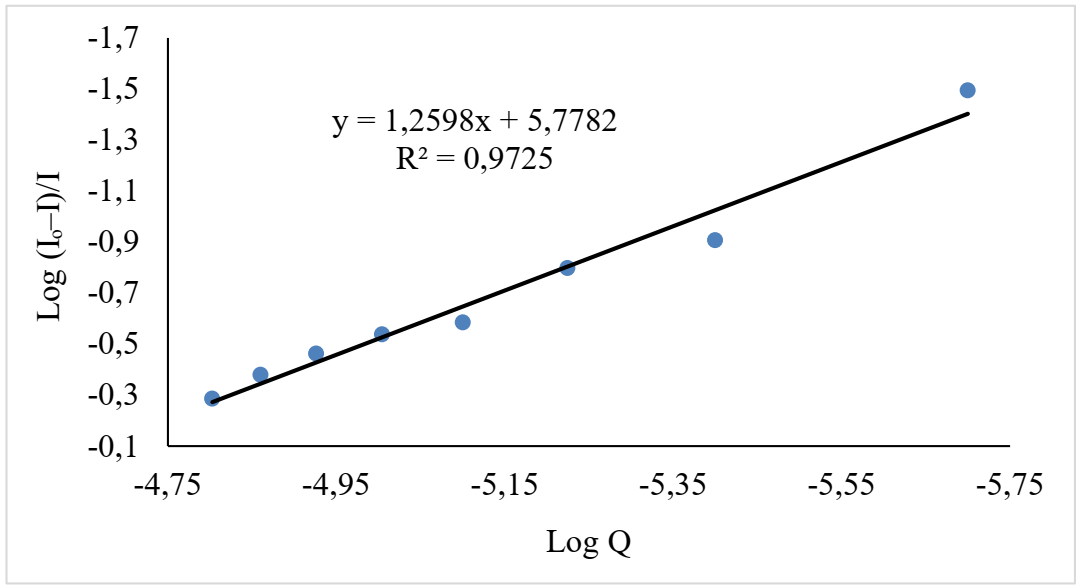
Figure S55: The double-logarithmic plot of EB-CT-DNA–silver perchlorate interaction at room temperature.


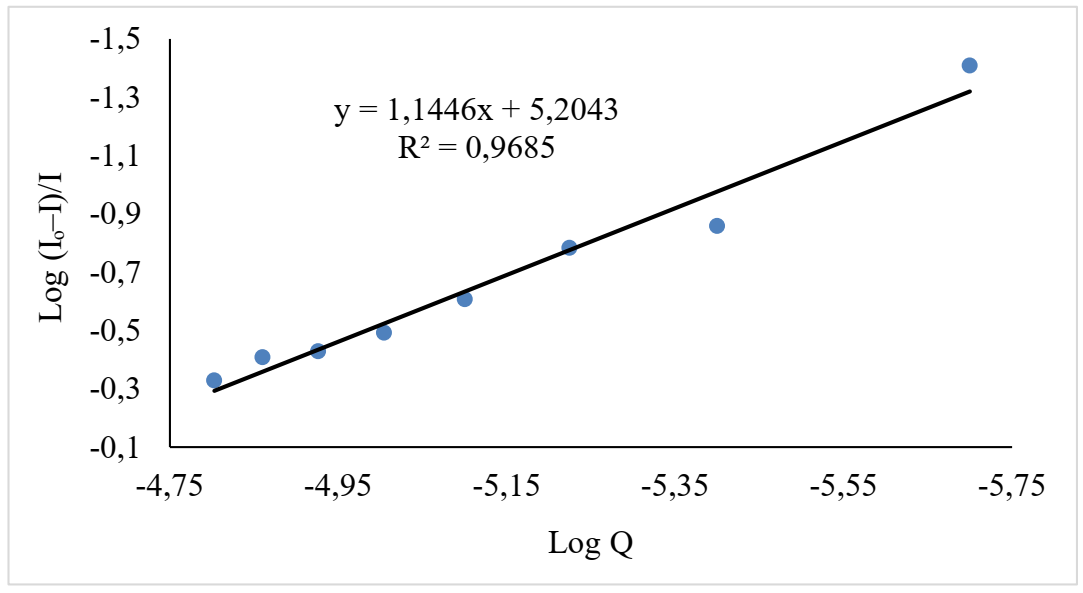


## Figure S56: The double-logarithmic plot of EB-CT-DNA–silver triflate interaction at room temperature.

# **BSA Binding studies using Electronic Absorption method**


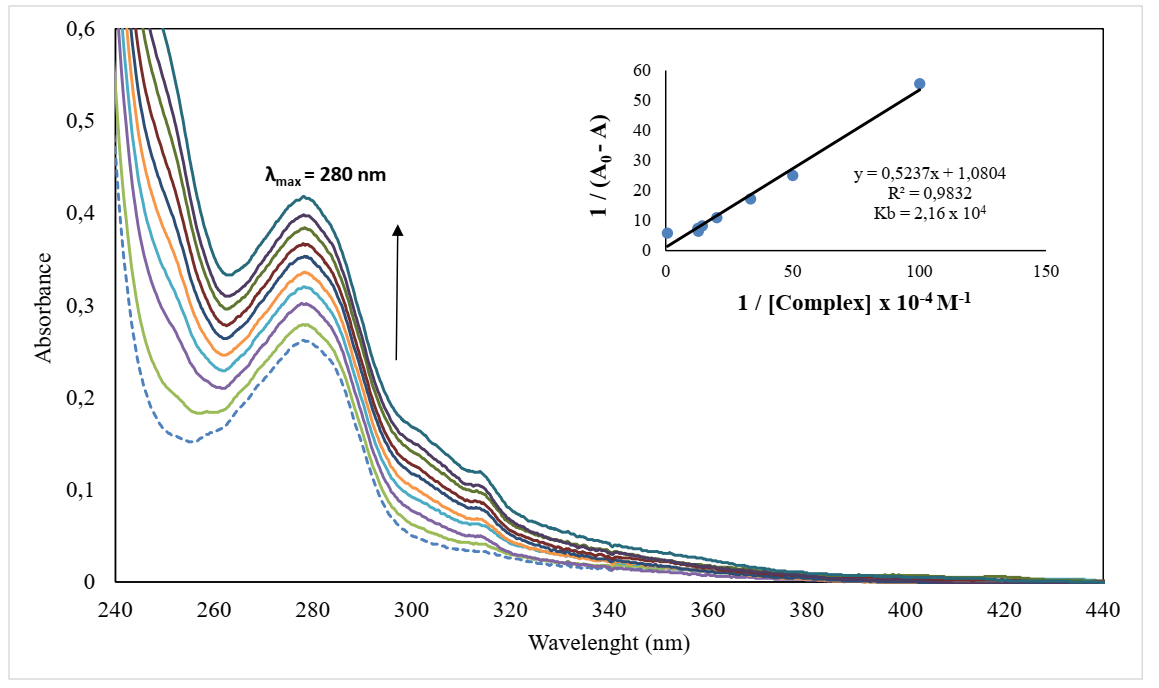


## Figure S57: Electronic Absorption Spectra of BSA in the absence (dashed line) and the presence of different concentrations of complexes **Q1**. (inset) Plot of 1/(Aₒ ‒ A) vs. 1/[Complex] x 10^-4^ M^-1^


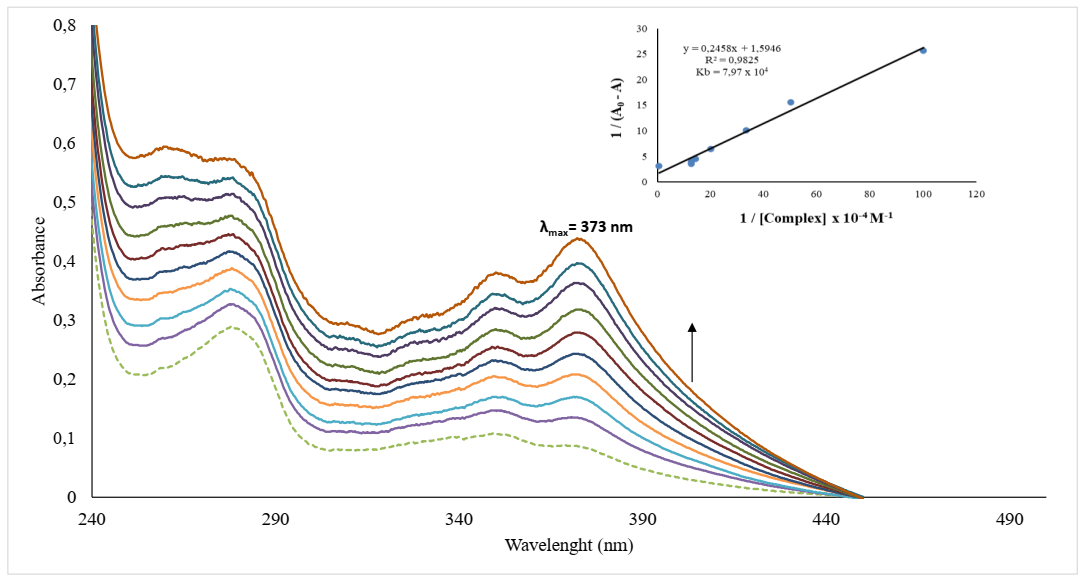


## Figure S58: Electronic Absorption Spectra of BSA in the absence (dashed line) and the presence of different concentrations of complexes **Q2**. (inset) Plot of 1/(Aₒ ‒ A) vs. 1/[Complex] x 10^-4^ M^-1^


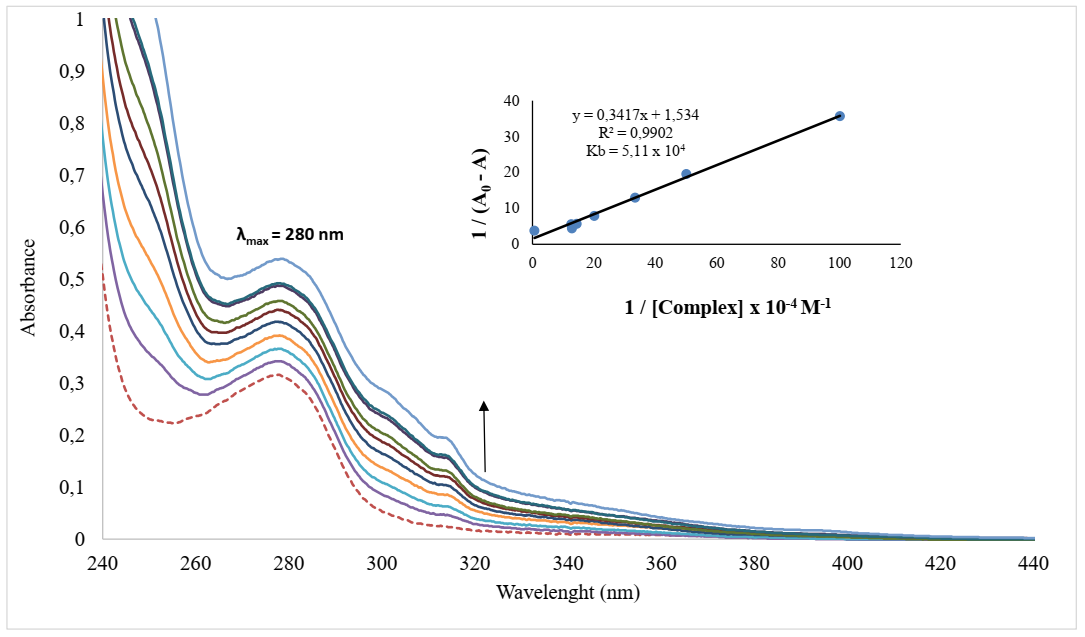


## Figure S59: Electronic Absorption Spectra of BSA in the absence (dashed line) and the presence of different concentrations of complexes **Q3**. (inset) Plot of 1/(Aₒ ‒ A) vs. 1/[Complex] x 10^-4^ M^-1^


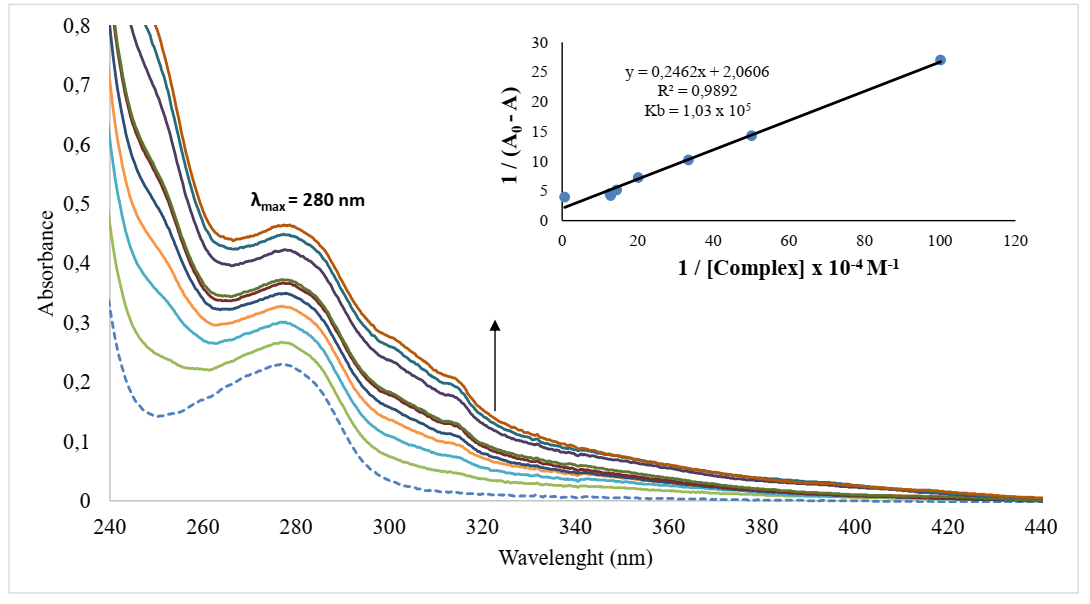


## Figure S60: Electronic Absorption Spectra of BSA in the absence (dashed line) and the presence of different concentrations of complexes **Q4**. (inset) Plot of 1/(Aₒ ‒ A) vs. 1/[Complex] x 10^-4^ M^-1^


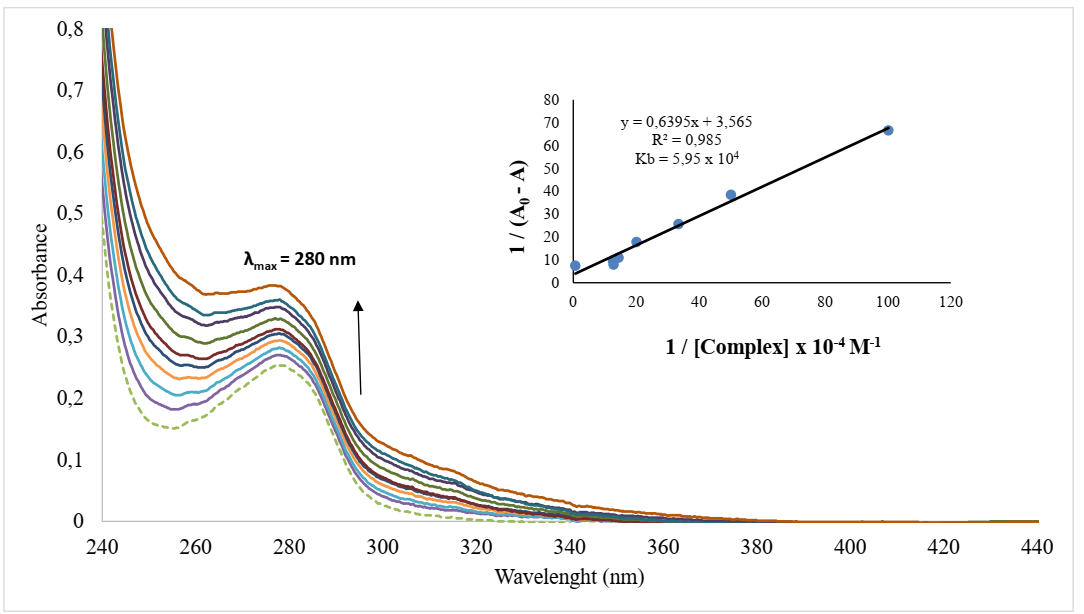


## Figure S61: Electronic Absorption Spectra of BSA in the absence (dashed line) and the presence of different concentrations of complexes **Q5**. (inset) Plot of 1/(Aₒ ‒ A) vs. 1/[Complex] x 10^-4^ M^-1^


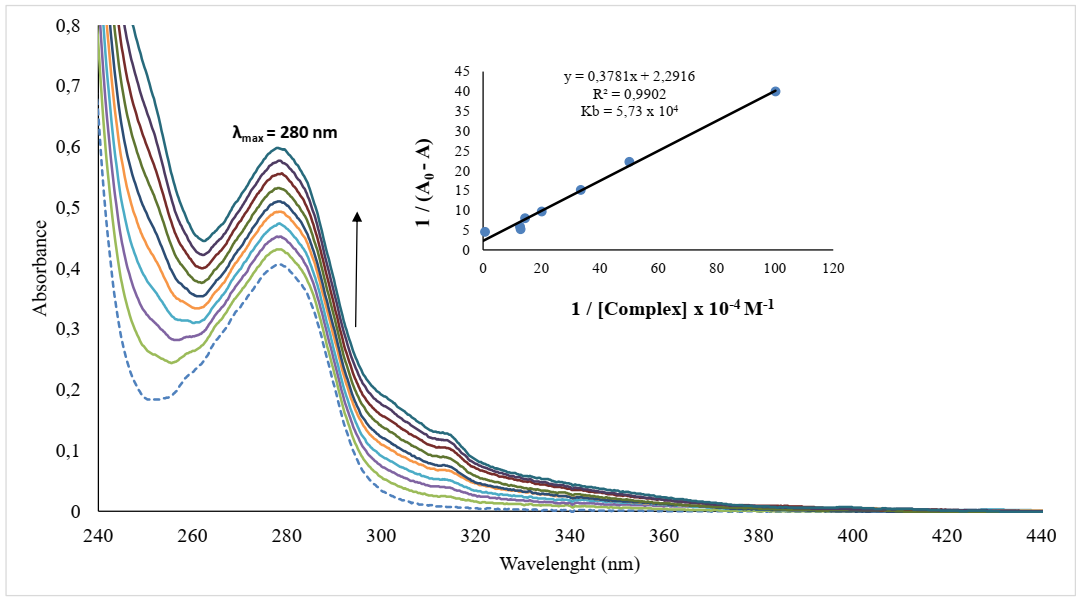


## Figure S62: Electronic Absorption Spectra of BSA in the absence (dashed line) and the presence of different concentrations of complexes **Q6**. (inset) Plot of 1/(Aₒ ‒ A) vs. 1/[Complex] x 10^-4^ M^-1^


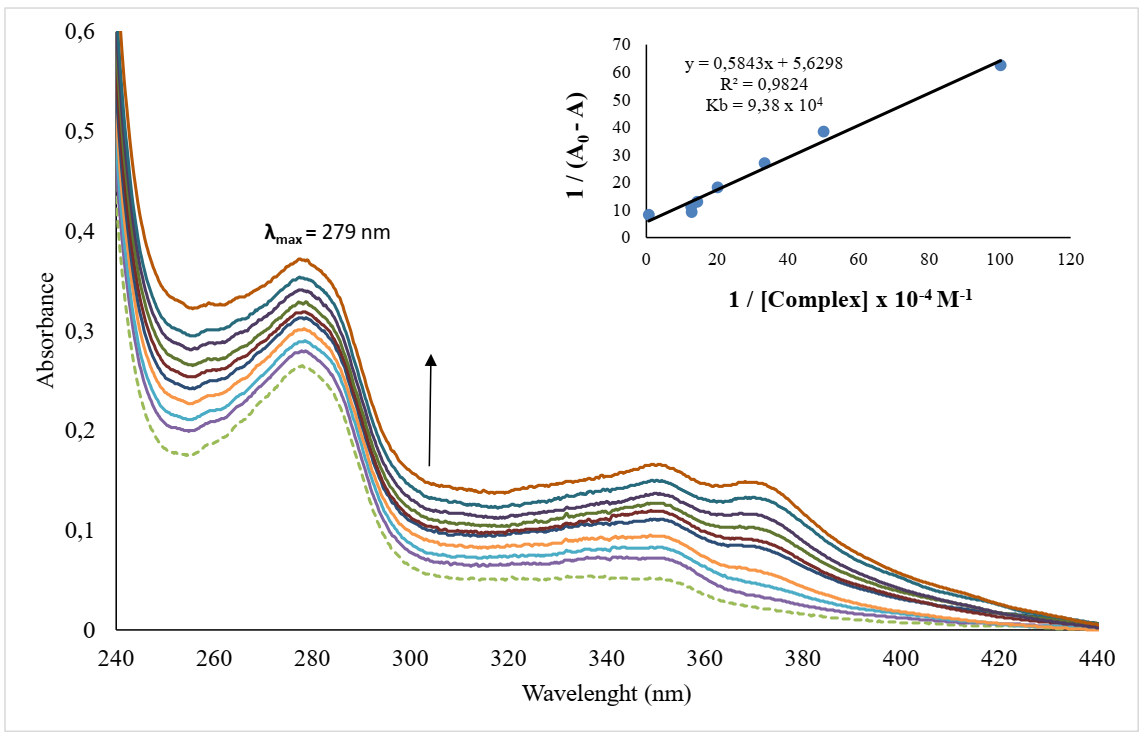


## Figure S63: Electronic Absorption Spectra of BSA in the absence (dashed line) and the presence of different concentrations of complexes **Q7**. (inset) Plot of 1/(Aₒ ‒ A) vs. 1/[Complex] x 10^-4^ M^-1^


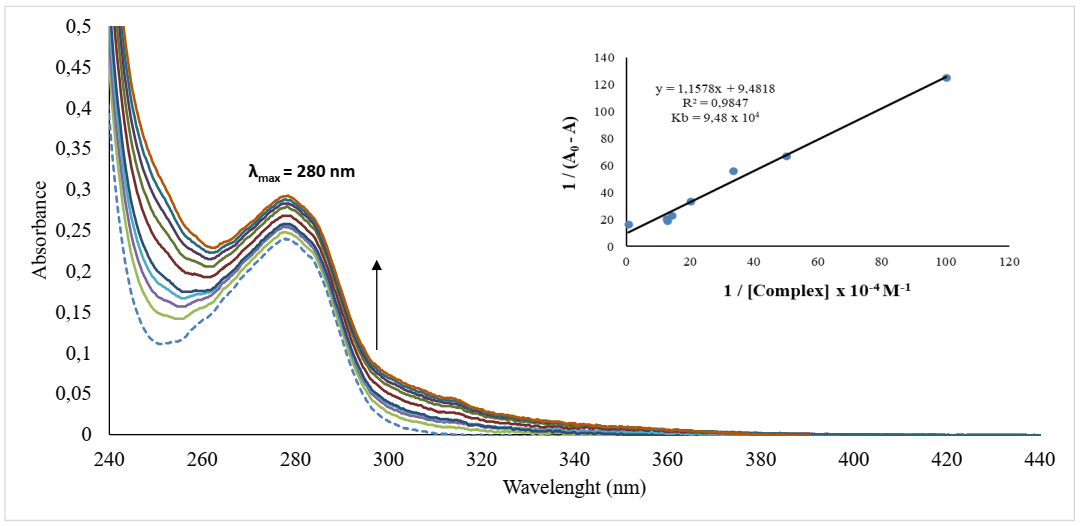


## Figure S64: Electronic Absorption Spectra of BSA in the absence (dashed line) and the presence of different concentrations of complexes **Q8**. (inset) Plot of 1/(Aₒ ‒ A) vs. 1/[Complex] x 10^-4^ M^-1^


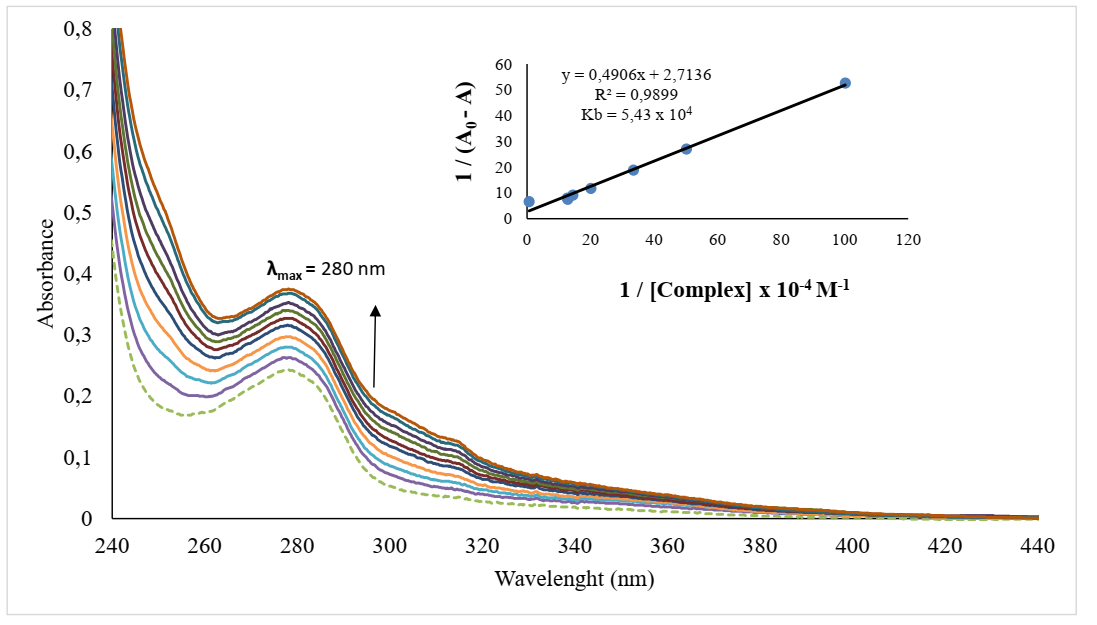


## Figure S65: Electronic Absorption Spectra of BSA in the absence (dashed line) and the presence of different concentrations of complexes **Q9**. (inset) Plot of 1/(Aₒ ‒ A) vs. 1/[Complex] x 10^-4^ M^-1^


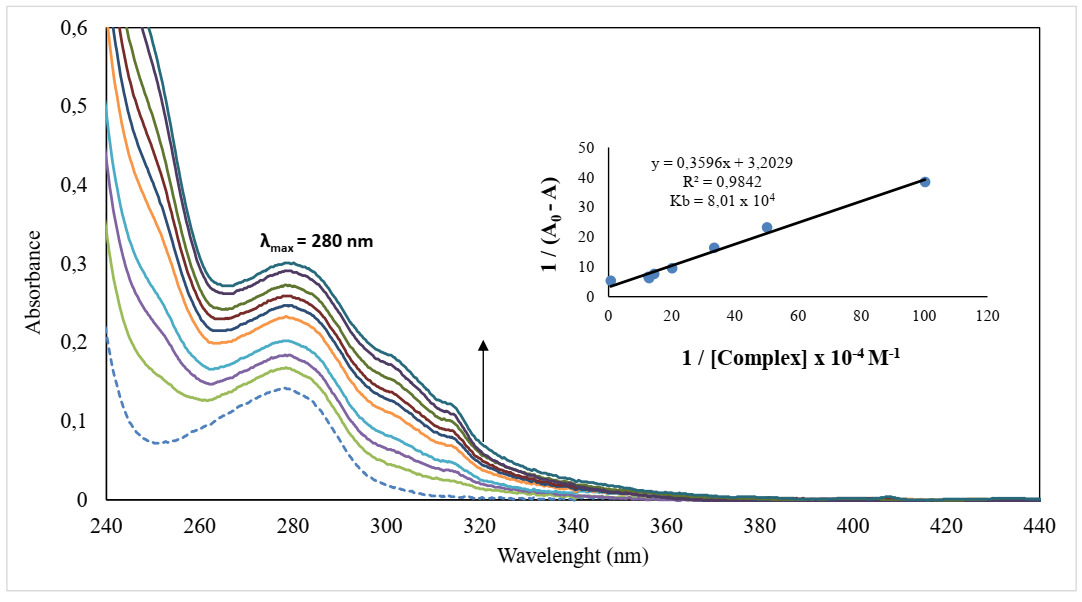


## Figure S66: Electronic Absorption Spectra of BSA in the absence (dashed line) and the presence of different concentrations of complexes **Q10**. (inset) Plot of 1/(Aₒ ‒ A) vs. 1/[Complex] x 10^-4^ M^-1^


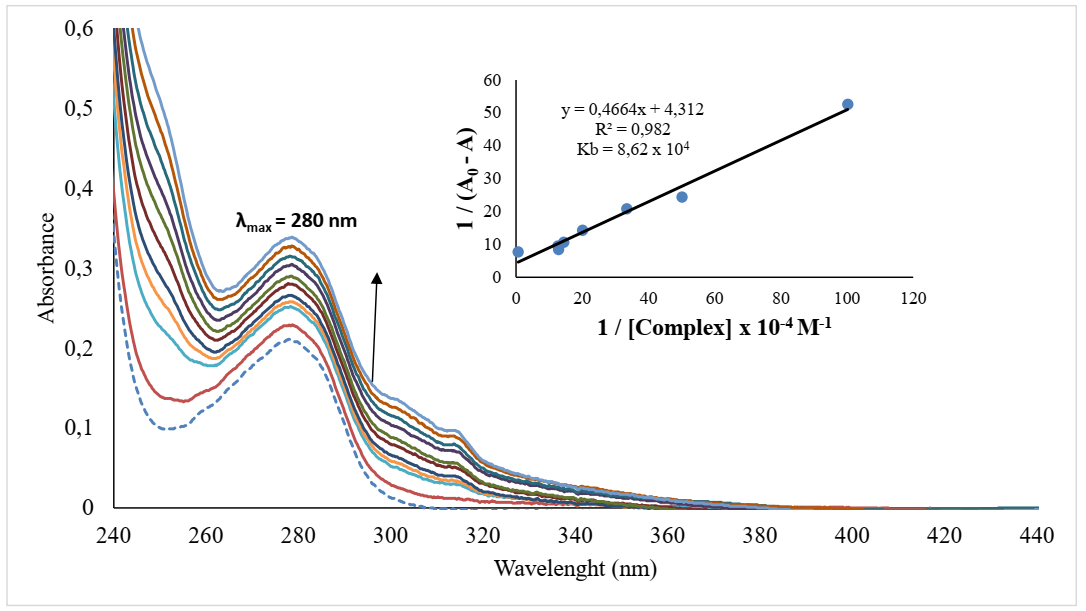


## Figure S67: Electronic Absorption Spectra of BSA in the absence (dashed line) and the presence of different concentrations of complexes **Q11**. (inset) Plot of 1/(Aₒ ‒ A) vs. 1/[Complex] x 10^-4^ M^-1^


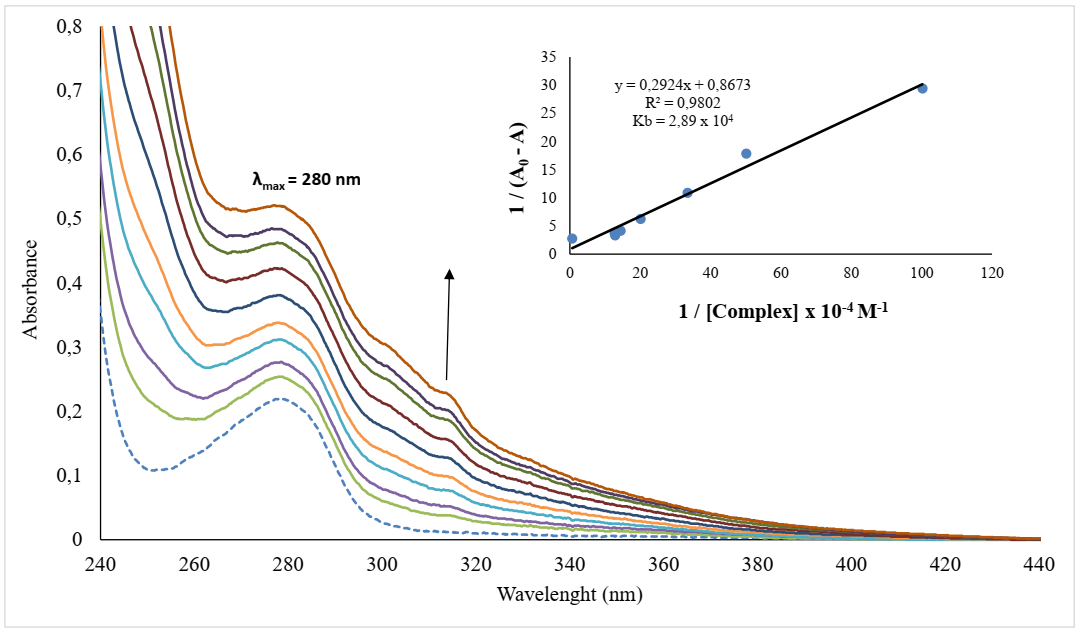


## Figure S68: Electronic Absorption Spectra of BSA in the absence (dashed line) and the presence of different concentrations of complexes **Q13**. (inset) Plot of 1/(Aₒ ‒ A) vs. 1/[Complex] x 10^-4^ M^-1^


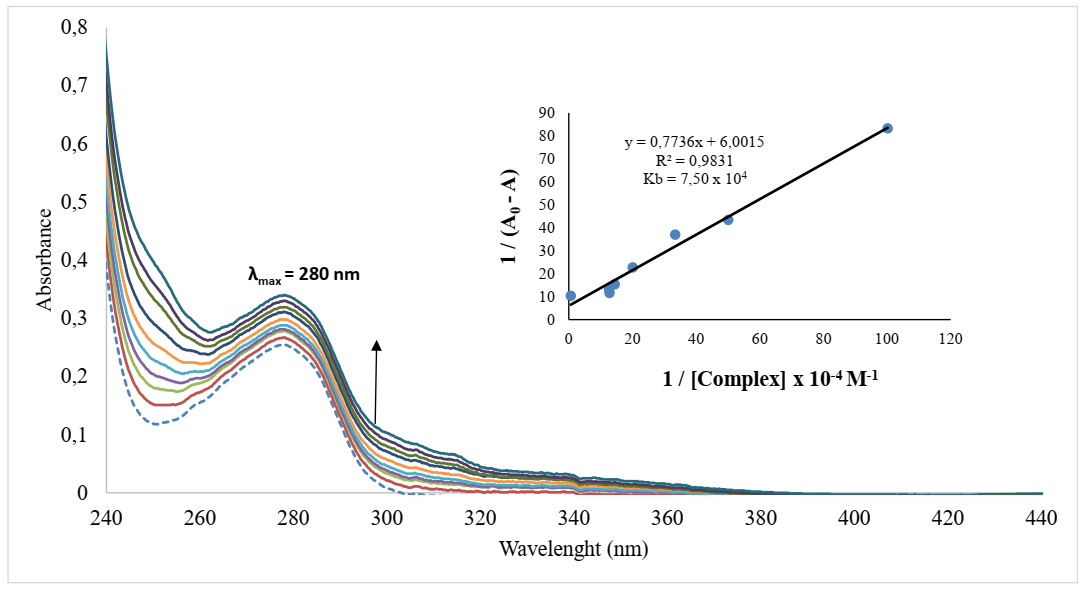


## Figure S69: Electronic Absorption Spectra of BSA in the absence (dashed line) and the presence of different concentrations of complexes **Q14**. (inset) Plot of 1/(Aₒ ‒ A) vs. 1/[Complex] x 10^-4^ M^-1^


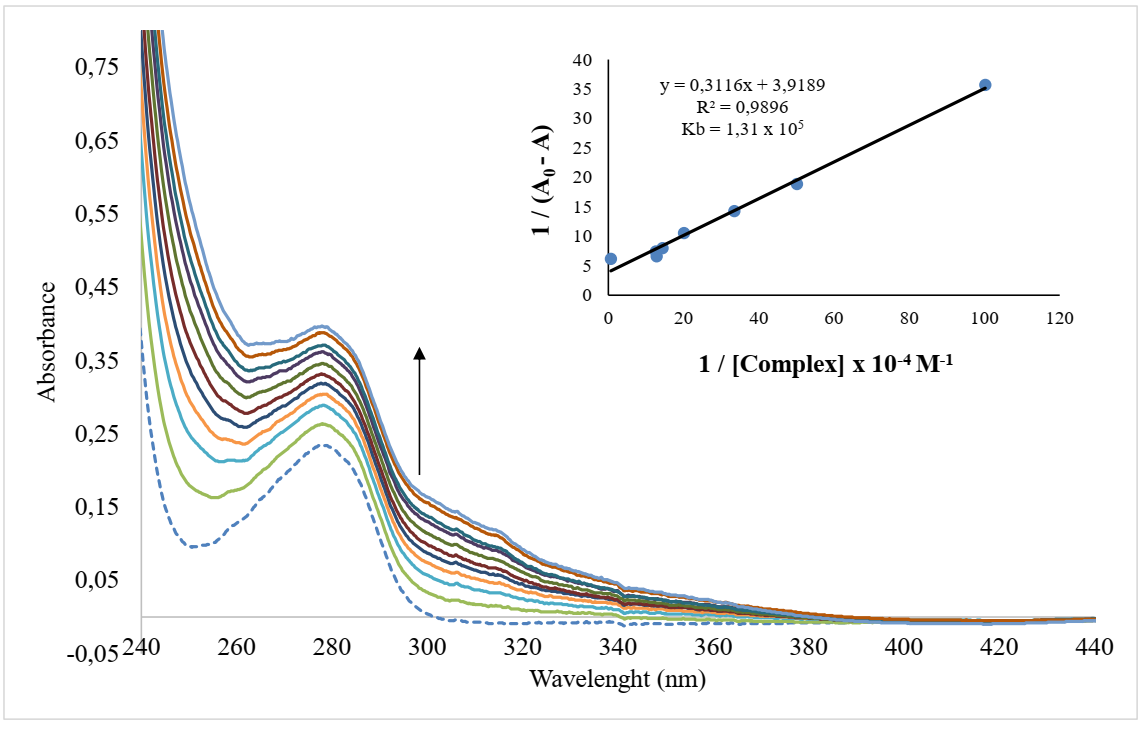


## Figure S70: Electronic Absorption Spectra of BSA in the absence (dashed line) and the presence of different concentrations of complexes **Q15**. (inset) Plot of 1/(Aₒ ‒ A) vs. 1/[Complex] x 10^-4^ M^-1^


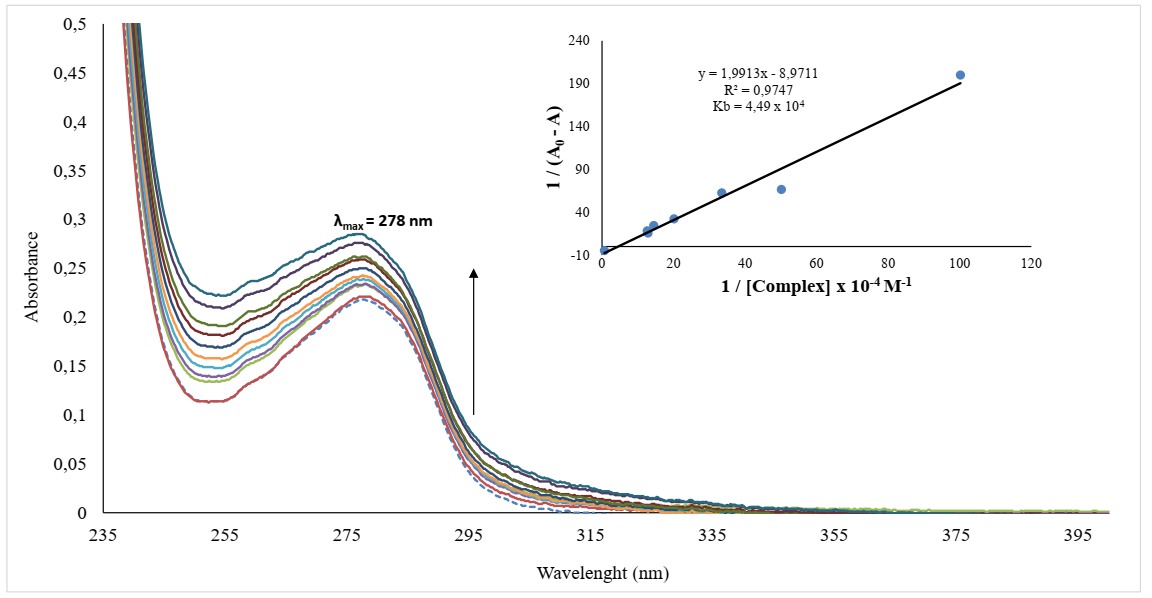


## Figure S71: Electronic Absorption Spectra of BSA in the absence (dashed line) and the presence of different concentrations of complexes silver nitrate. (inset) Plot of 1/(Aₒ ‒ A) vs. 1/[Complex] x 10^-4^ M^-1^


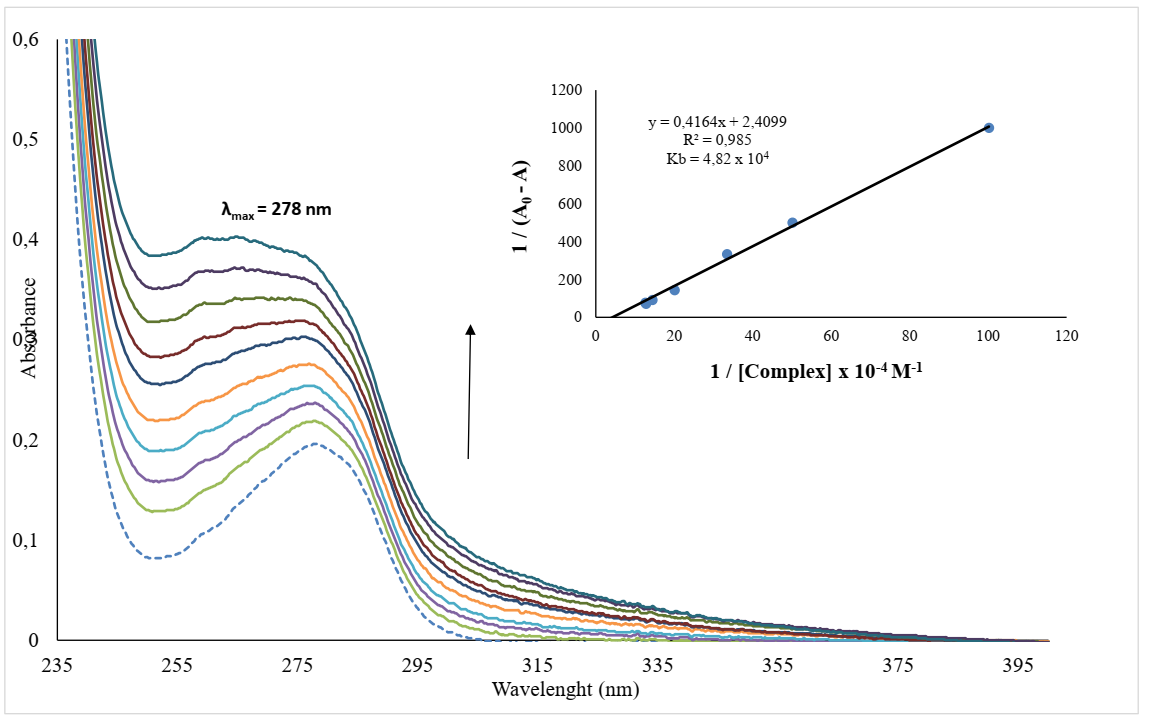


## Figure S72: Electronic Absorption Spectra of BSA in the absence (dashed line) and the presence of different concentrations of complexes silver perchlorate. (inset) Plot of 1/(Aₒ ‒ A) vs. 1/[Complex] x 10^-4^ M^-1^


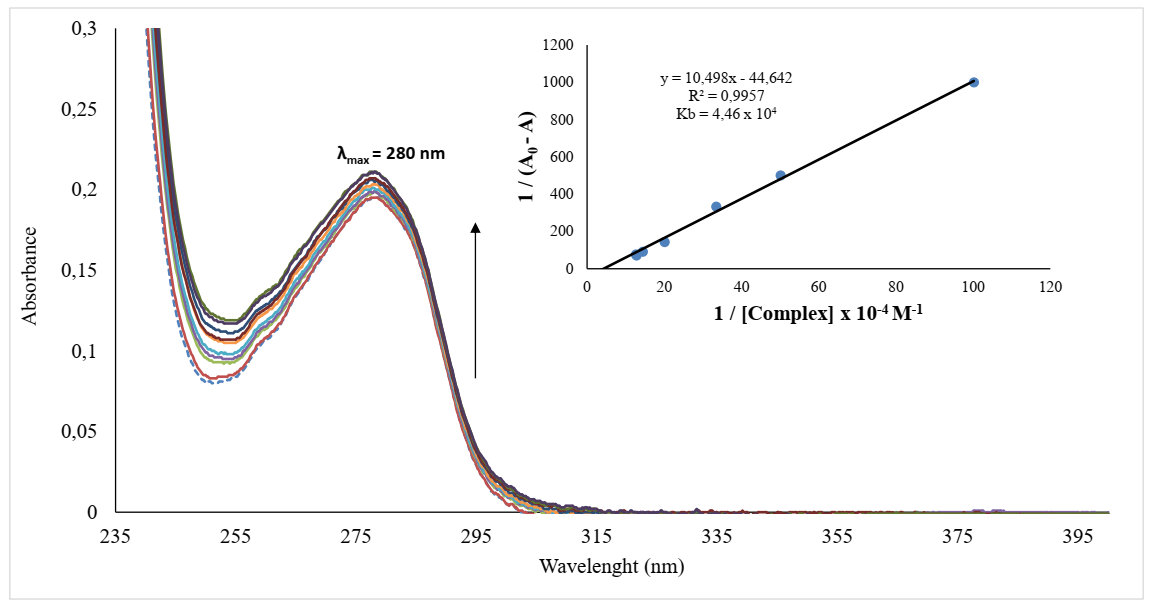


## Figure S73: Electronic Absorption Spectra of BSA in the absence (dashed line) and the presence of different concentrations of complexes silver trifluoromethanesulfonate. (inset) Plot of 1/(Aₒ ‒ A) vs. 1/[Complex] x 10^-4^ M^-1^

# **BSA Binding studies using the Fluorescence method**


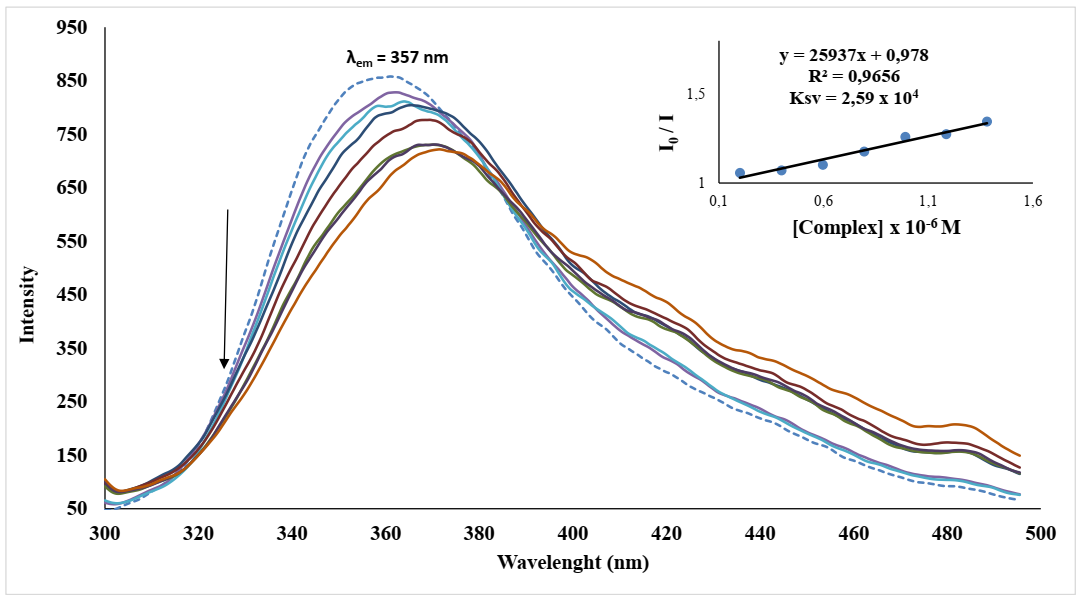


## **Figure S74:** Fluorescence emission spectra of BSA in the absence(dashed line) and the presence of different concentration of complex **Q1**. (inset) Stern-Volmer plot of complex **Q1** interaction with BSA


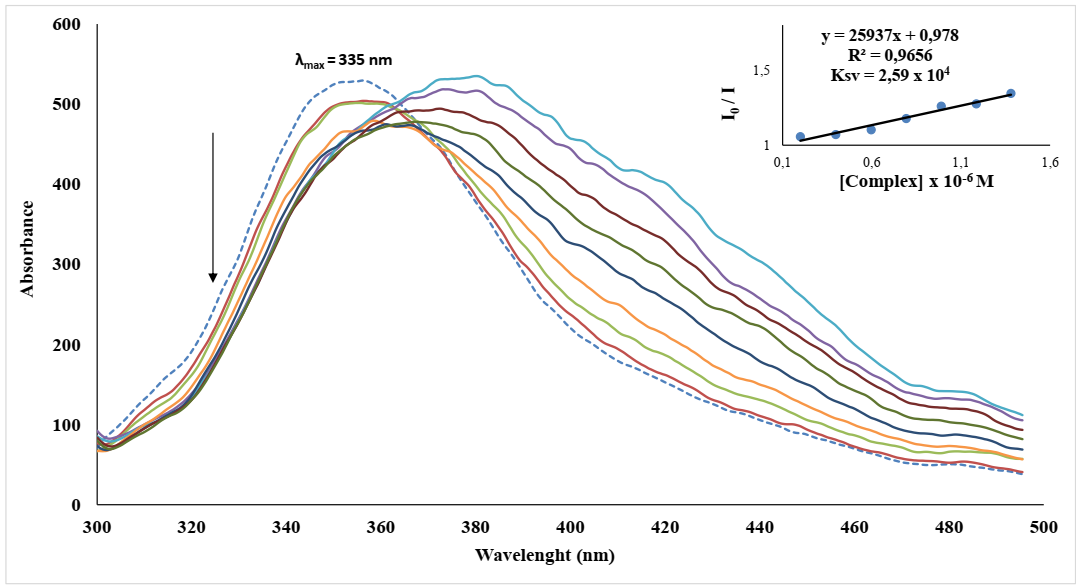


## **Figure S75:**Fluorescence emission spectra of BSA in the absence(dashed line) and the presence of different concentration of complex **Q6**. (inset) Stern-Volmer plot of complex **Q6** interaction with BSA

# **The double-logarithmic plot of BSA–Complexes interactions at room temperature.**


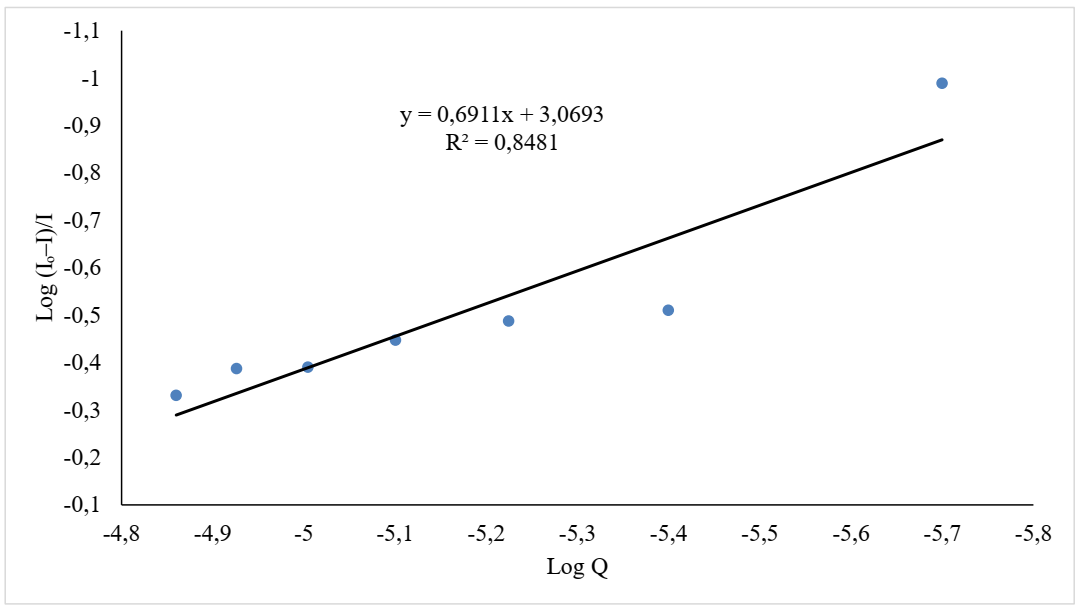


## **Figure S76:** The double-logarithmic plot of BSA–Complex **Q1** interactions.


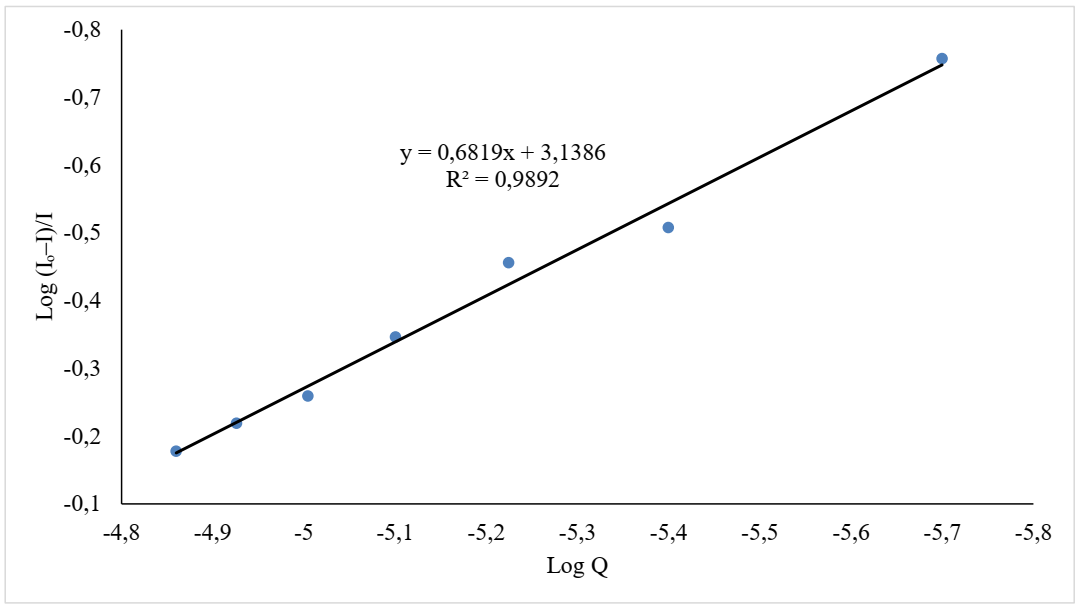


## **Figure S77:** The double-logarithmic plot of BSA–Complex **E2** interactions.

# **^1^H-NMR Spectra of Ligands L1-L5**


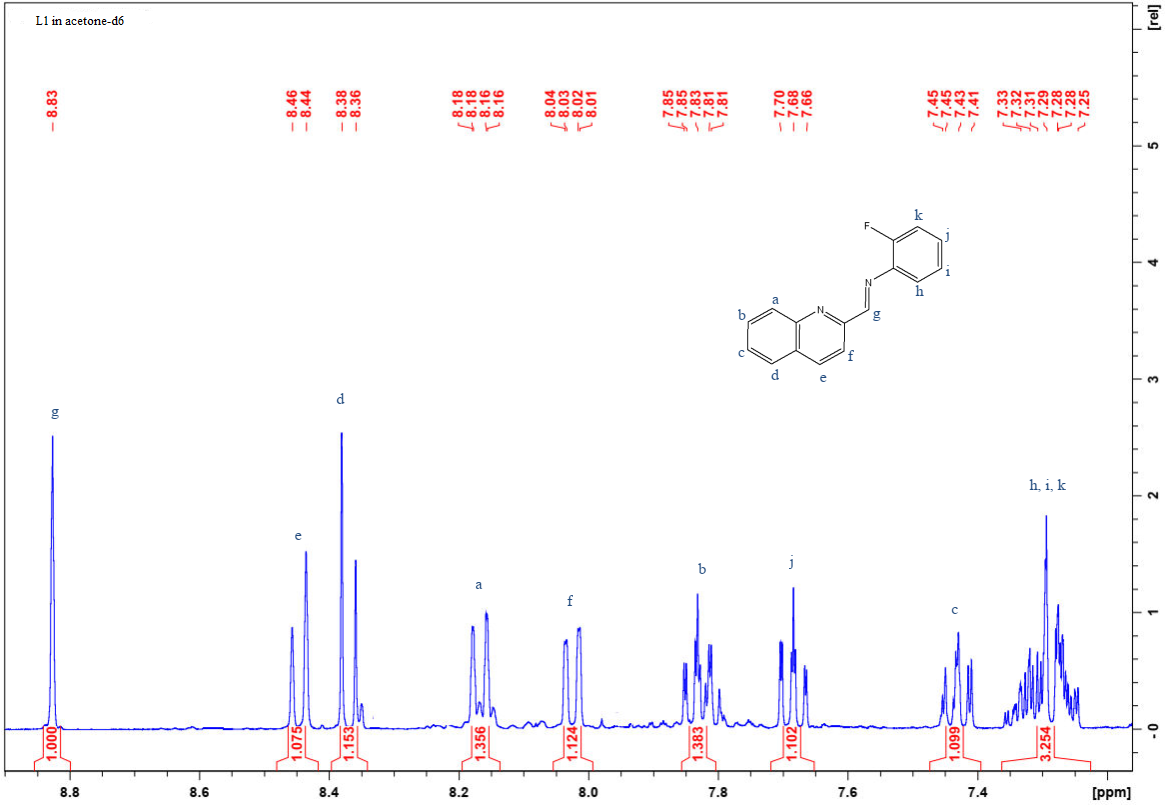


## Figure S78: (E)-N-(2-fluorophenyl)-1-(quinolin-2-yl)methanimine L1


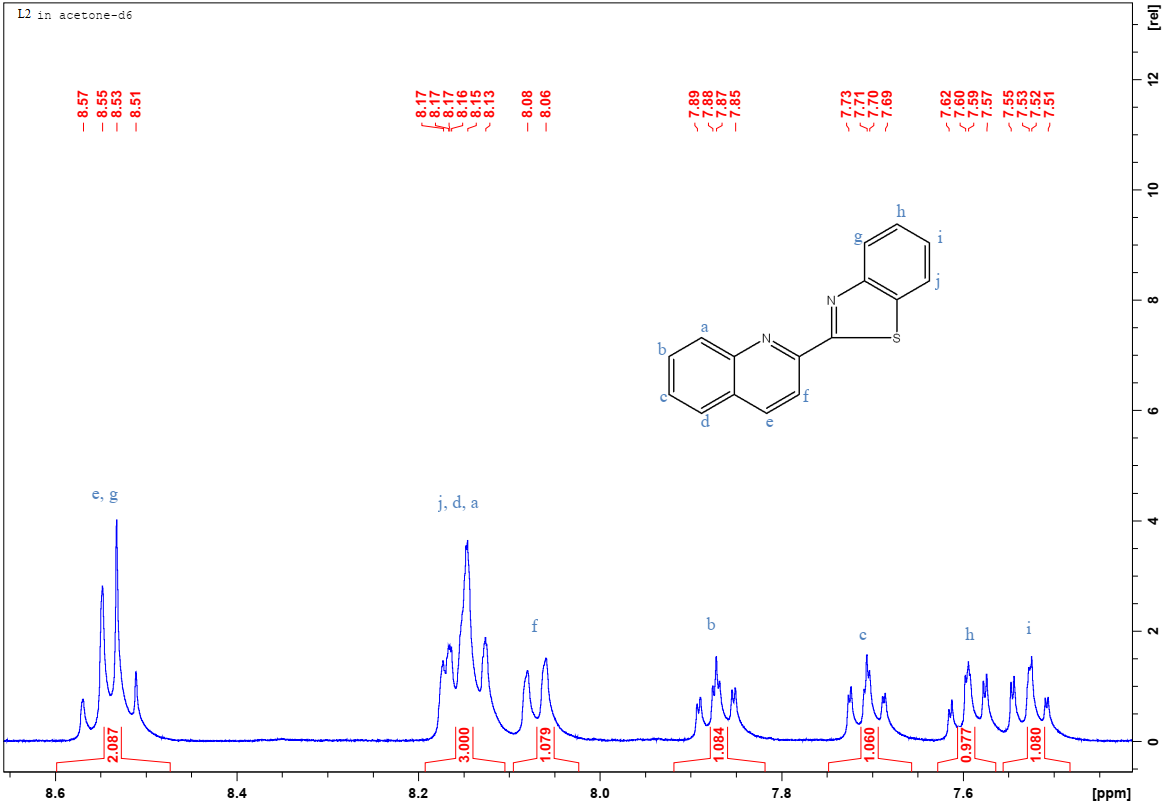


## Figure S79: 2-(quinolin-2-yl)benzo[d]thiazole L2


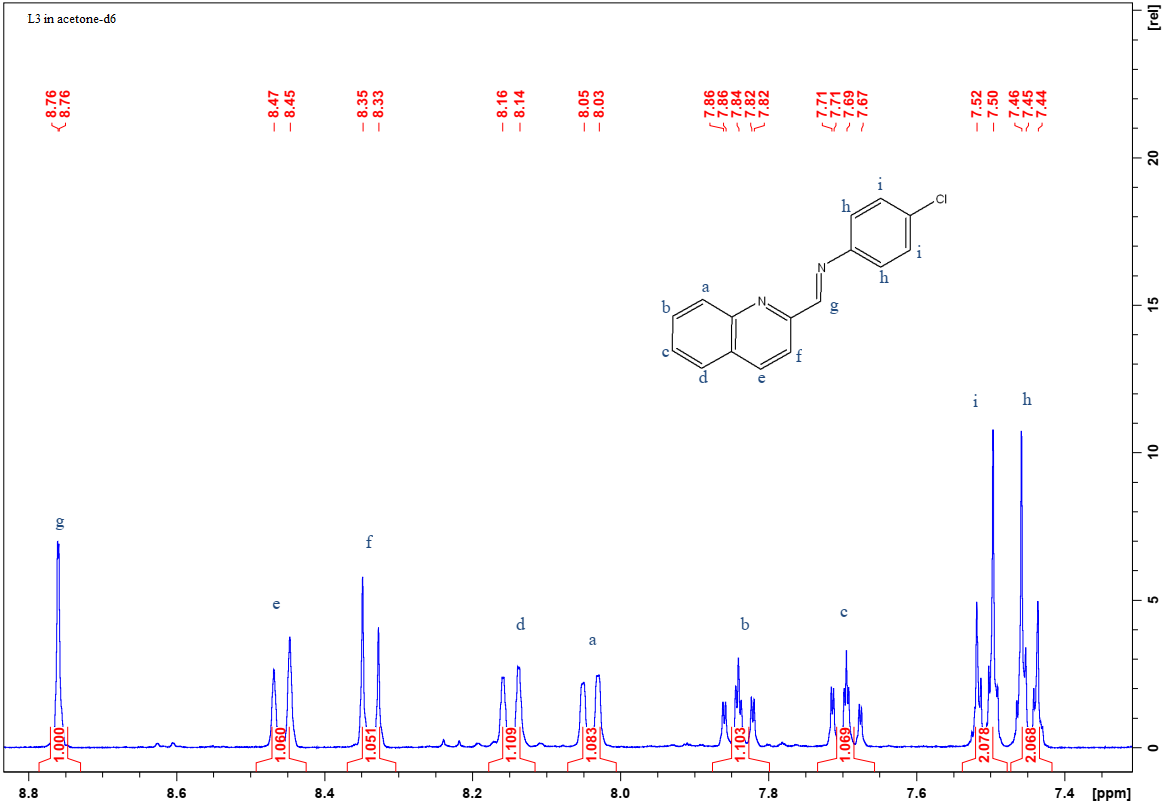


## Figure S80: (E)-N-(4-chlorophenyl)-1-(quinolin-2-yl)methanimine L3


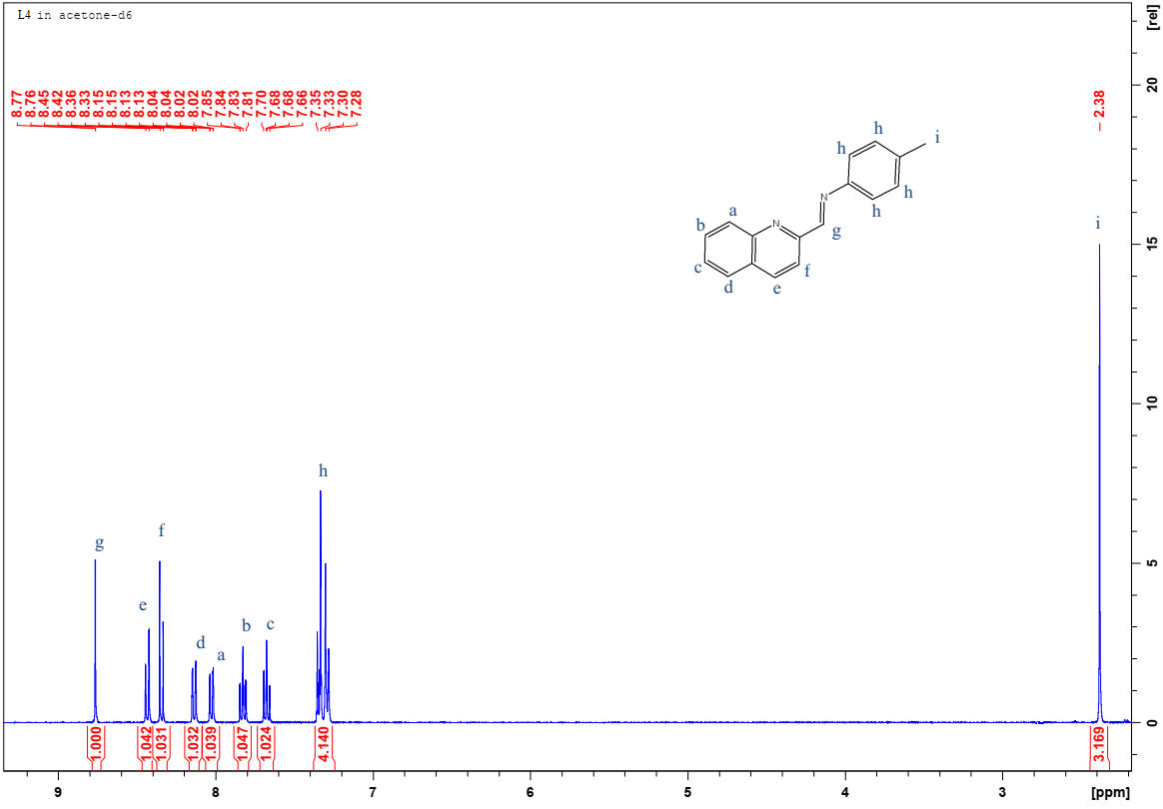


## Figure S81: (E)-1-(quinolin-2-yl)-N-(p-tolyl)methanimine L4


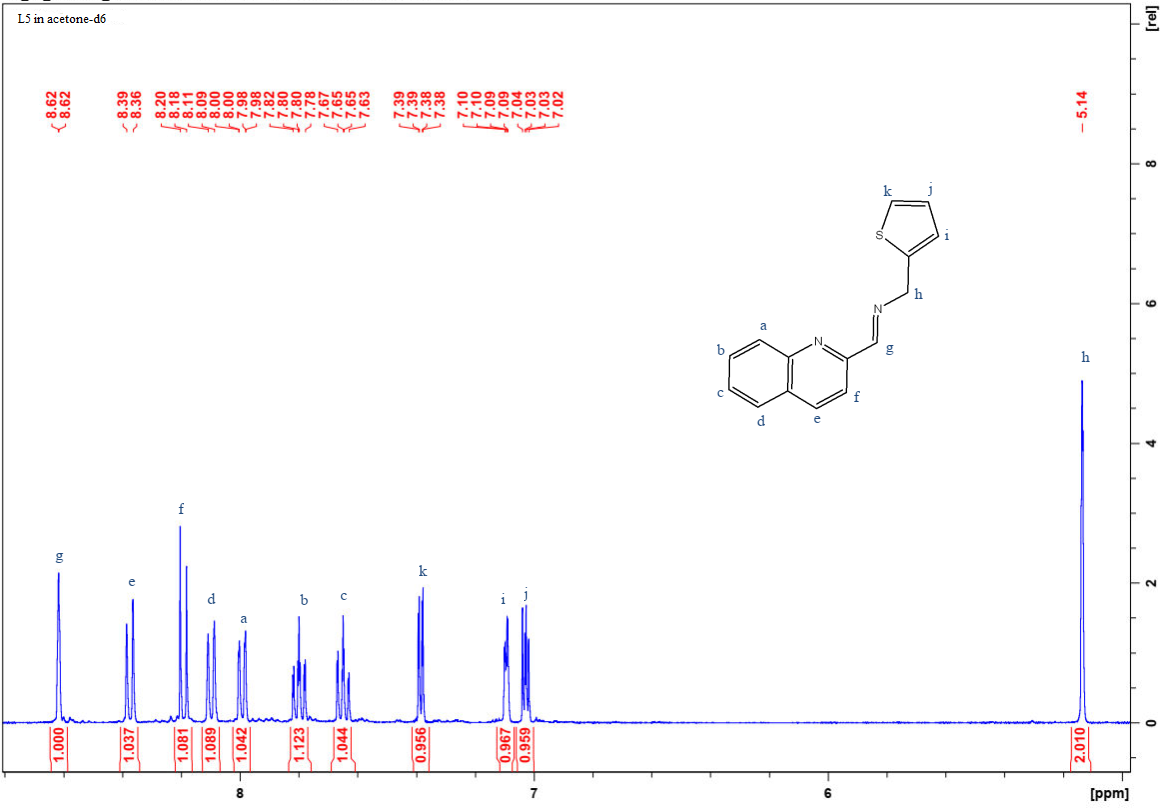


## Figure S82: (E)-1-(quinolin-2-yl)-N-(thiophen-2-ylmethyl)methanimine L5

# **^1^H-NMR Spectra of complexes Q1-Q5**

**
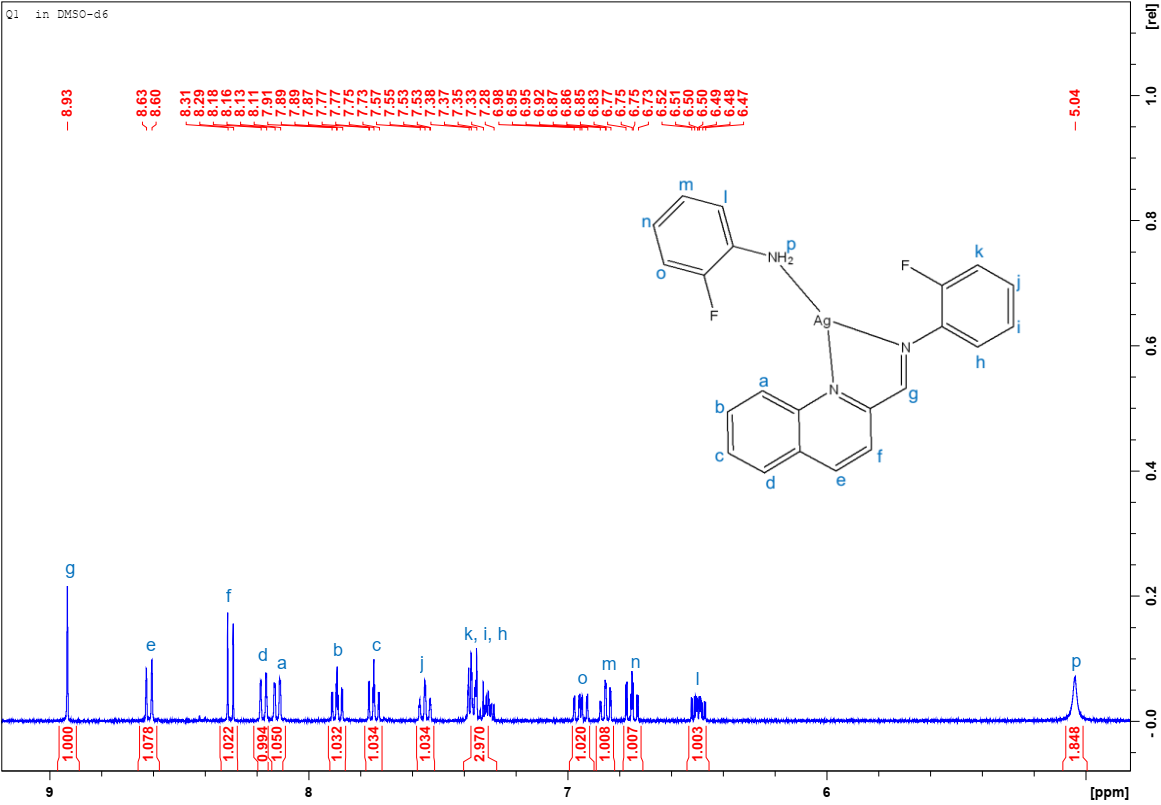
**

## Figure S83: ***[Ag(L1)_2_]NO_3_ Q1***


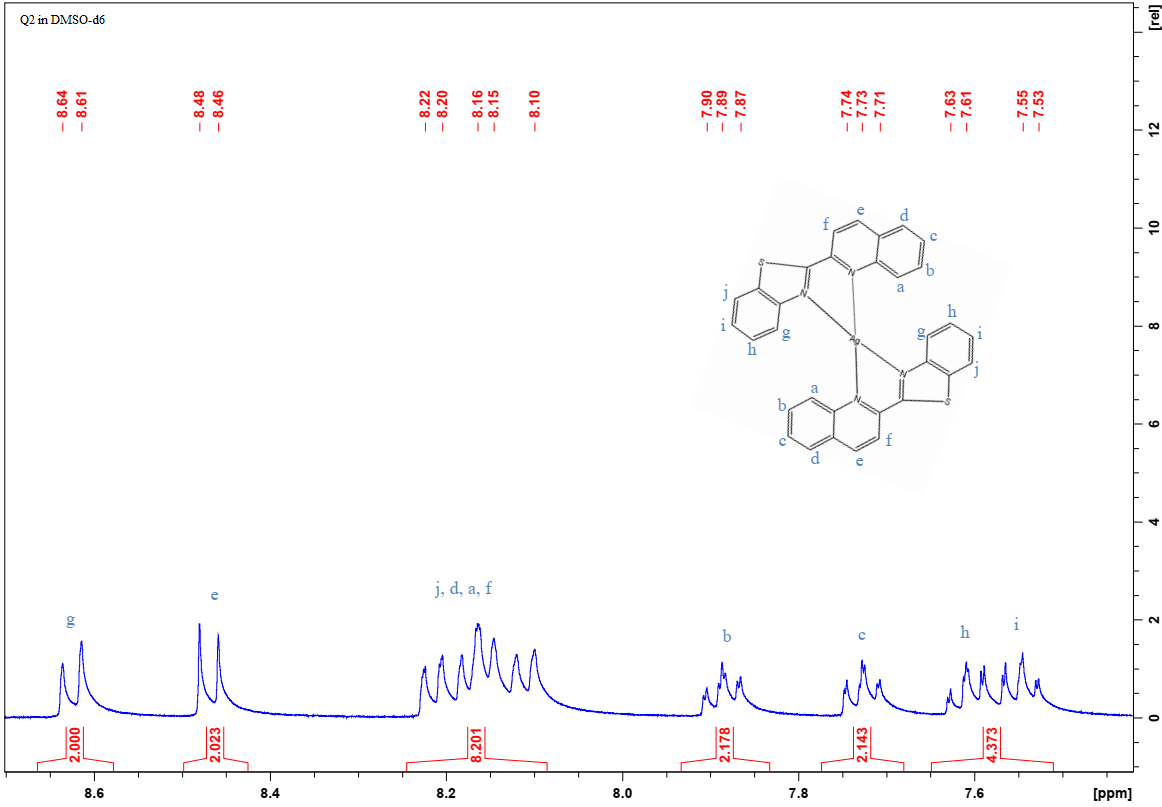


## Figure S84: ***[Ag(L2)_2_]NO_3_ Q2***


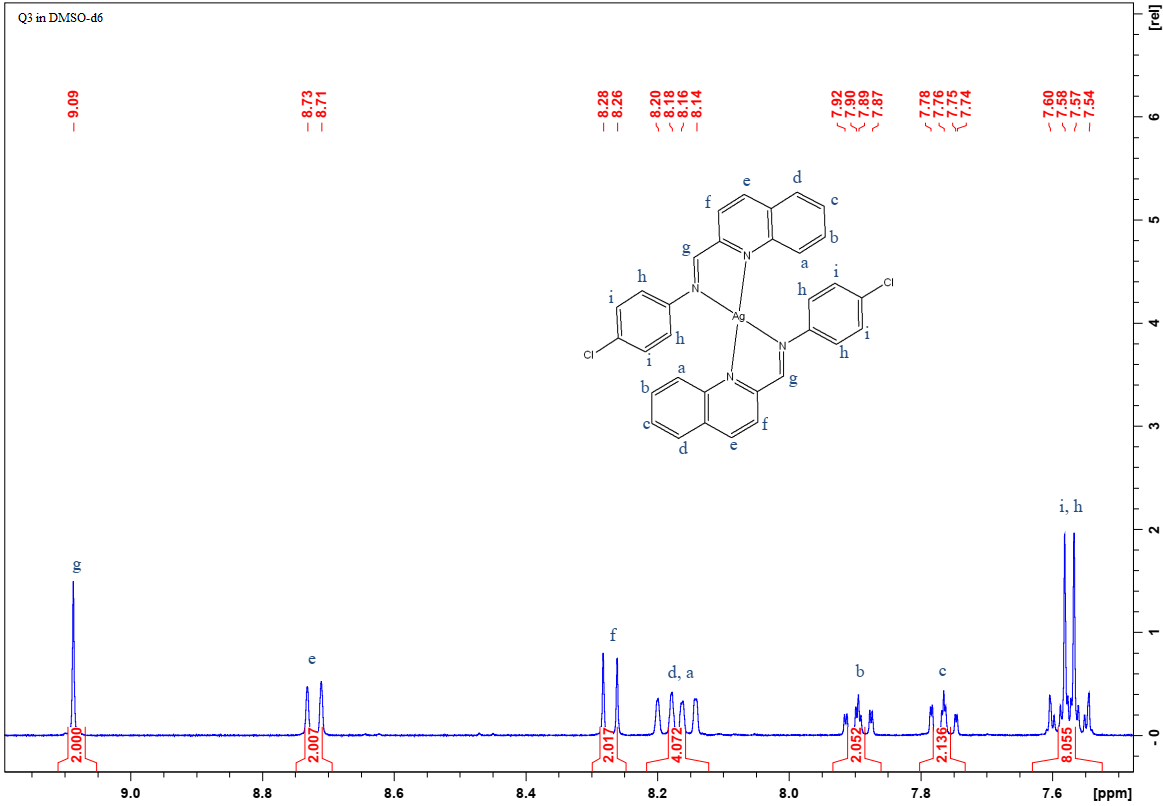


## Figure S85: ***[Ag(L3)_2_]NO_3_ Q3***


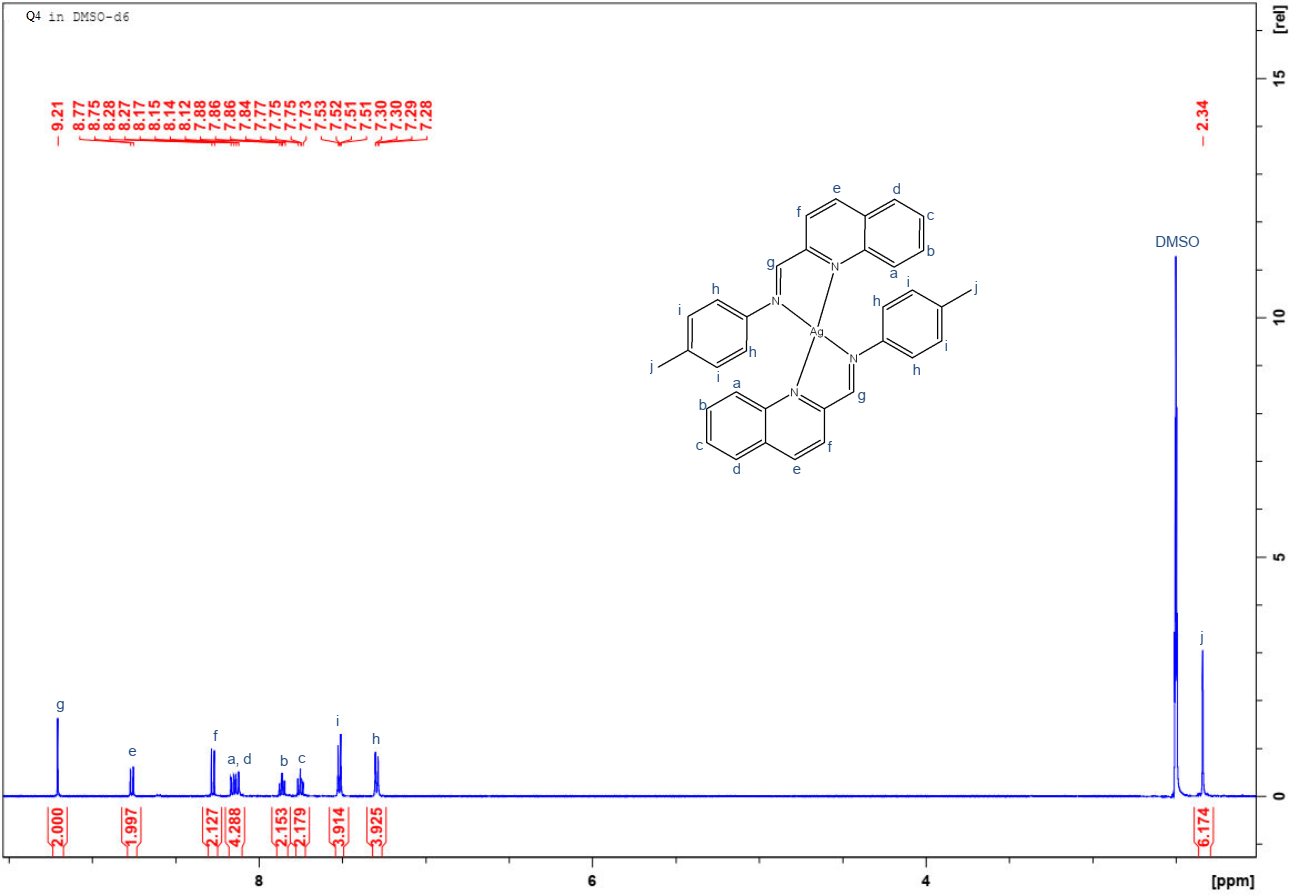


## Figure S86: ***[Ag(L4)_2_]NO_3_ Q4***


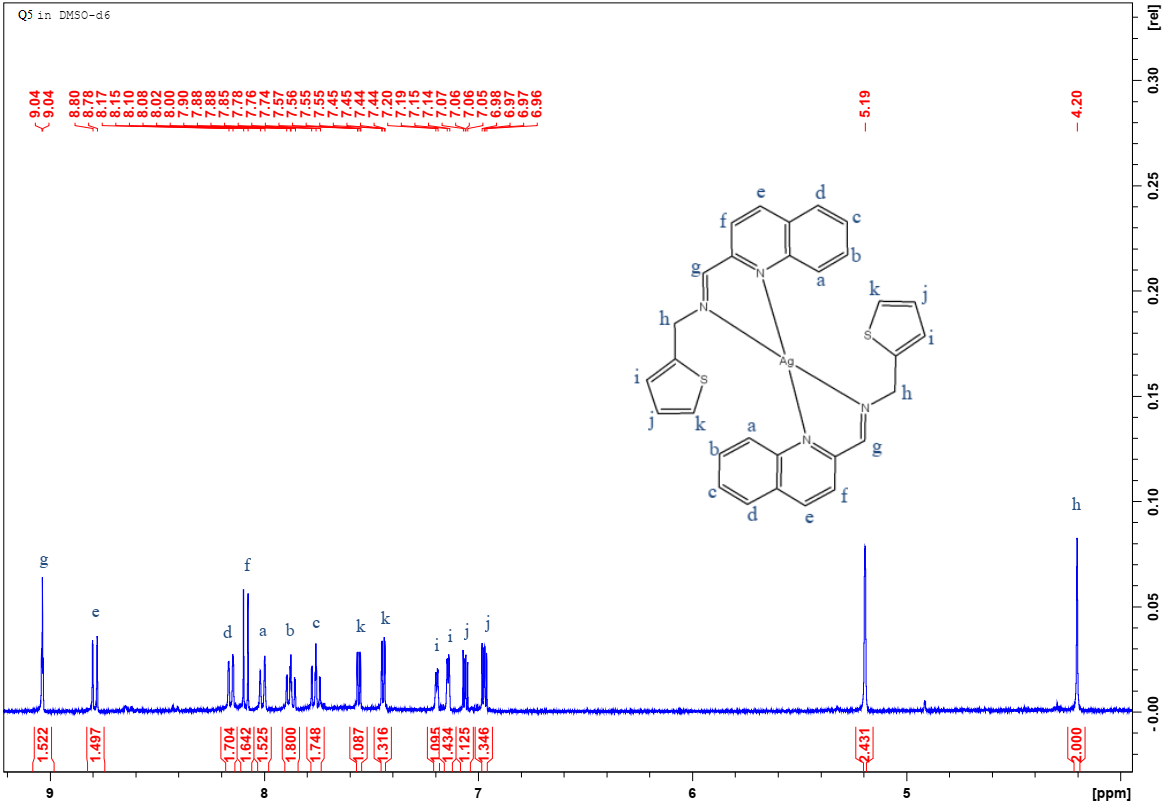


## Figure S87: ***[Ag(L5)_2_]NO_3_ Q5***


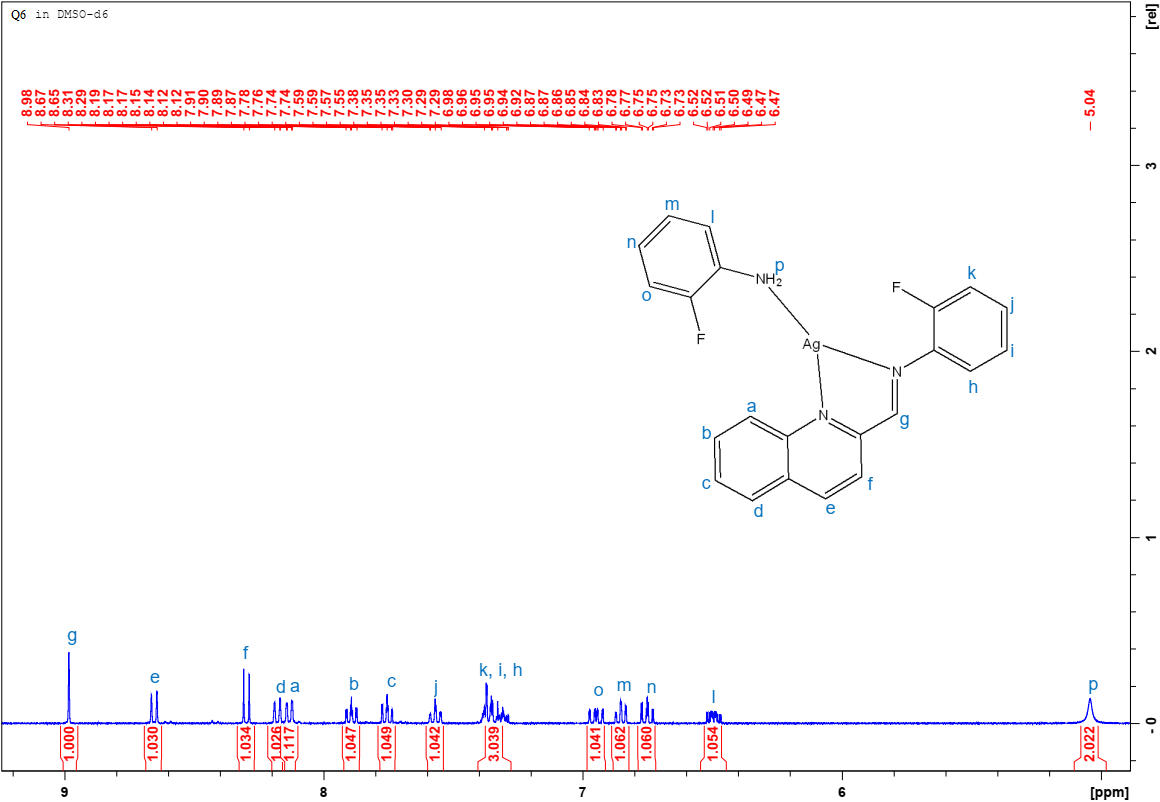


## Figure S88: ***[Ag(L1)_2_]ClO_4_ Q6***


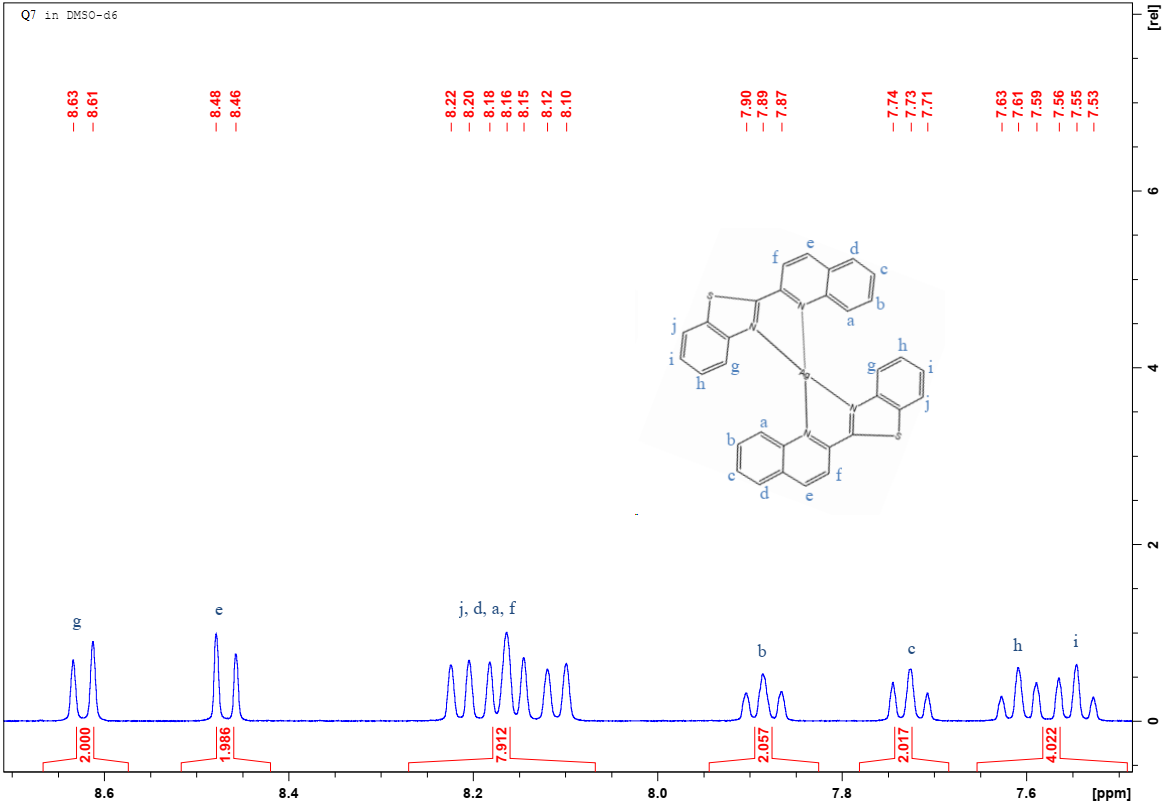


## Figure S89: ***[Ag(L2)_2_]ClO_4_ Q7***


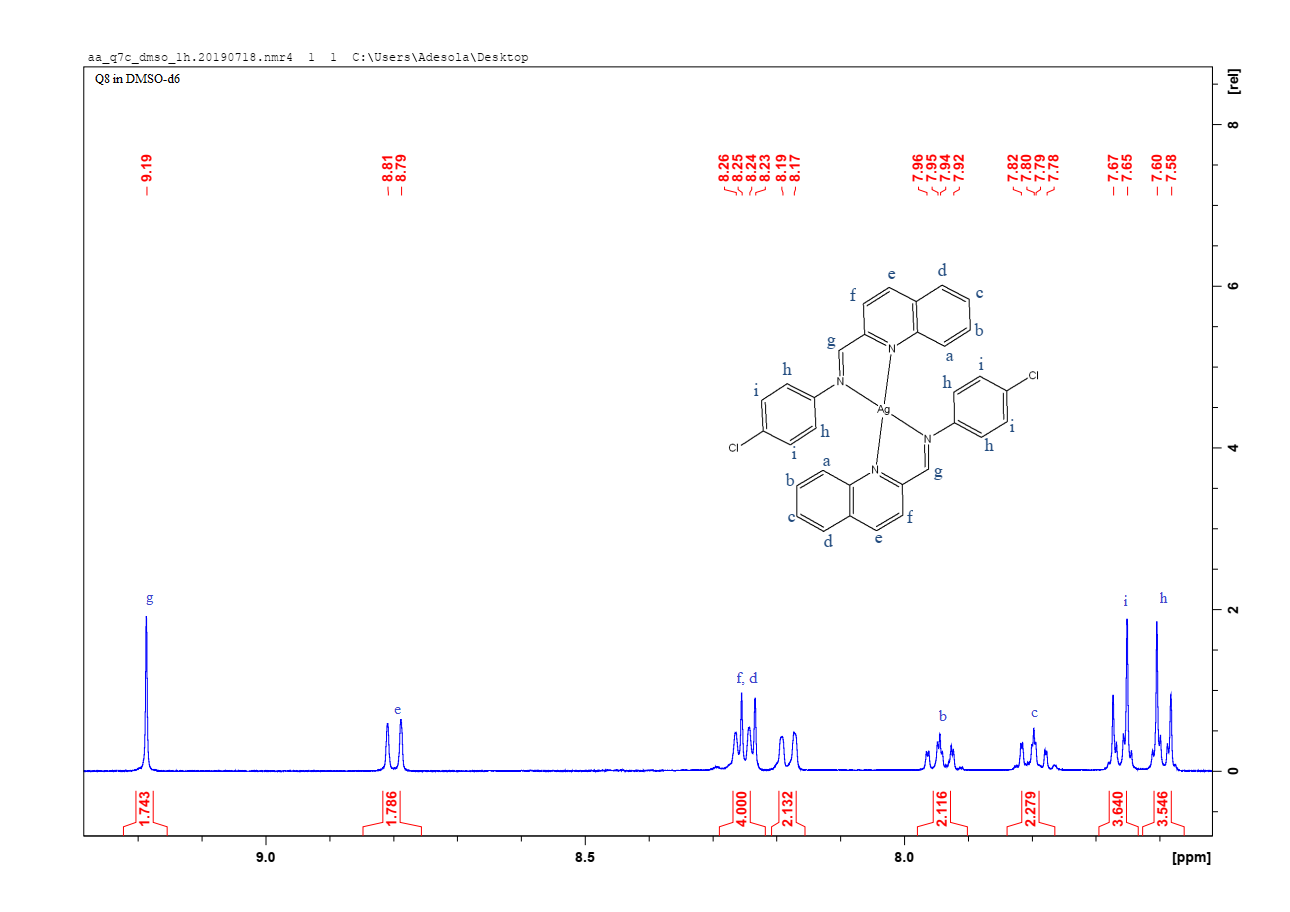


## Figure S90: ***[Ag(L3)_2_]ClO_4_ Q8***


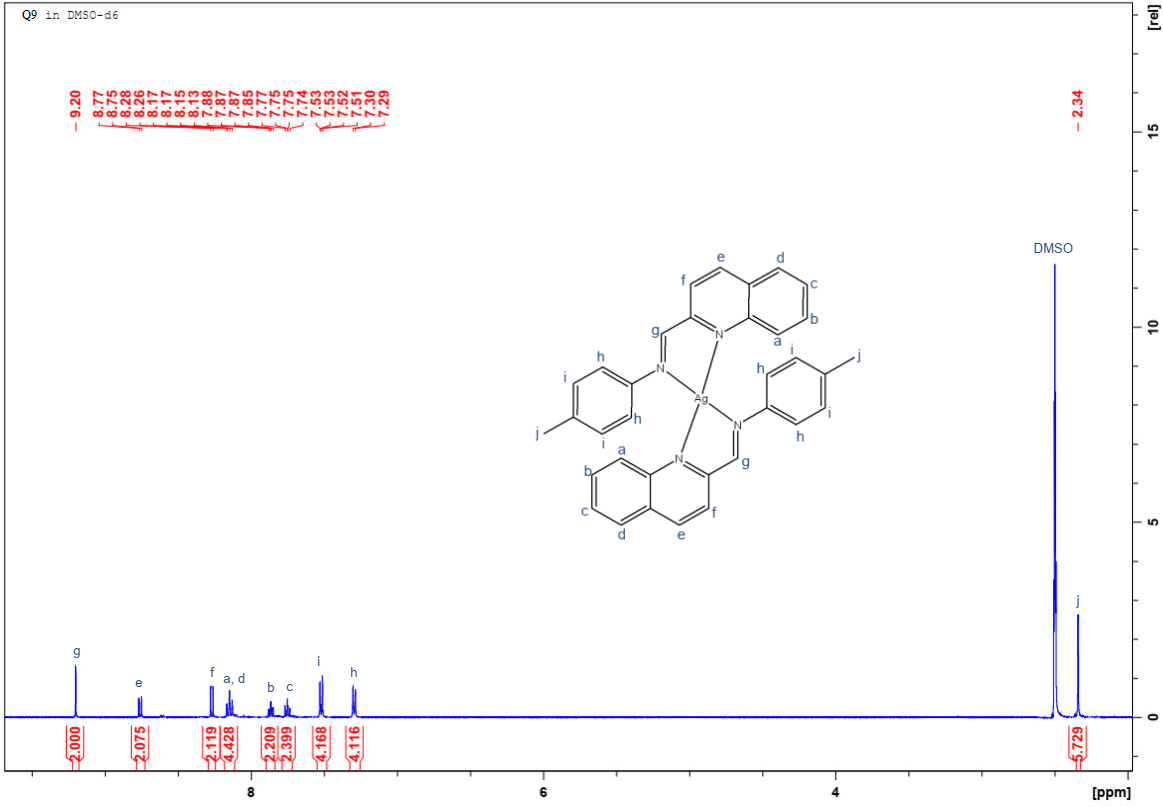


## Figure S91: ***[Ag(L4)_2_]ClO_4_ Q9***


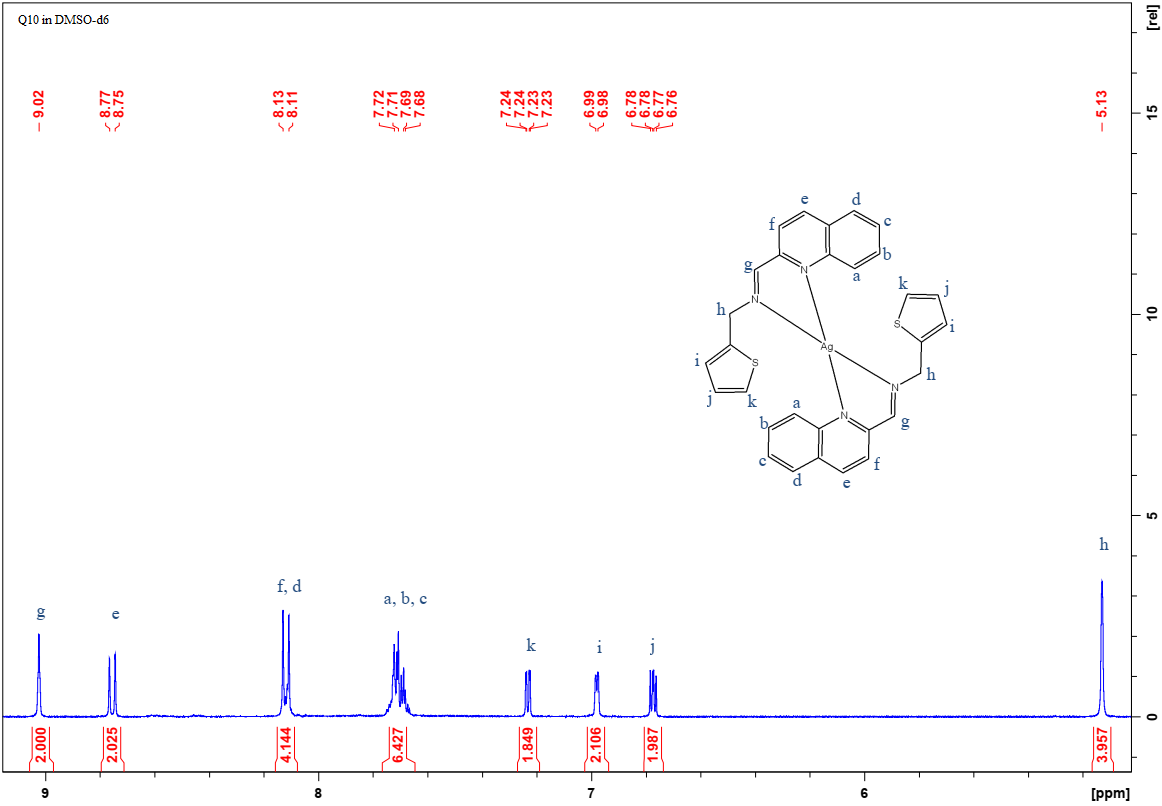


## Figure S92: ***[Ag(L5)_2_]ClO_4_ Q10***


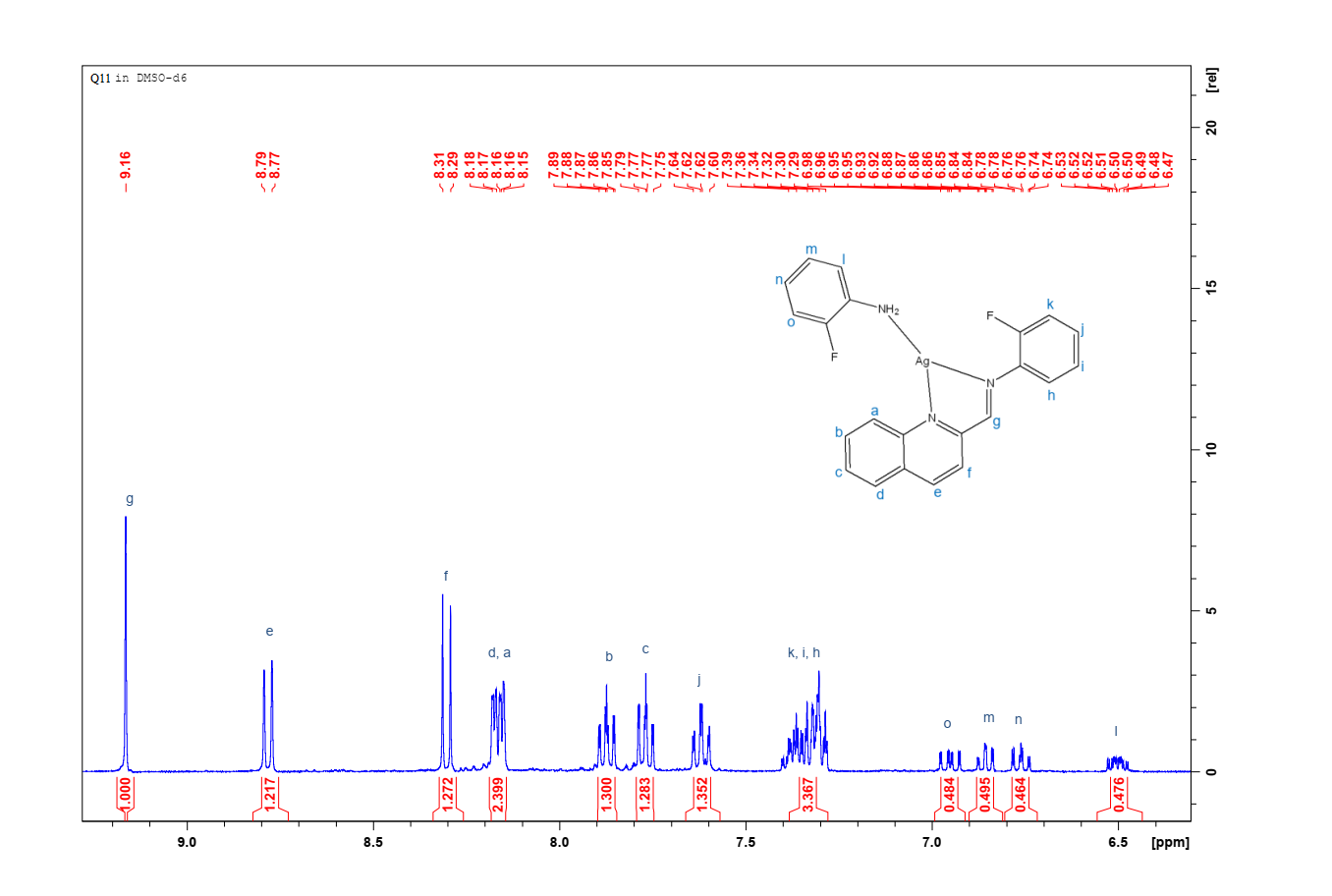


## Figure S93: ***[Ag(L1)_2_]CF_3_SO_3_ Q11***


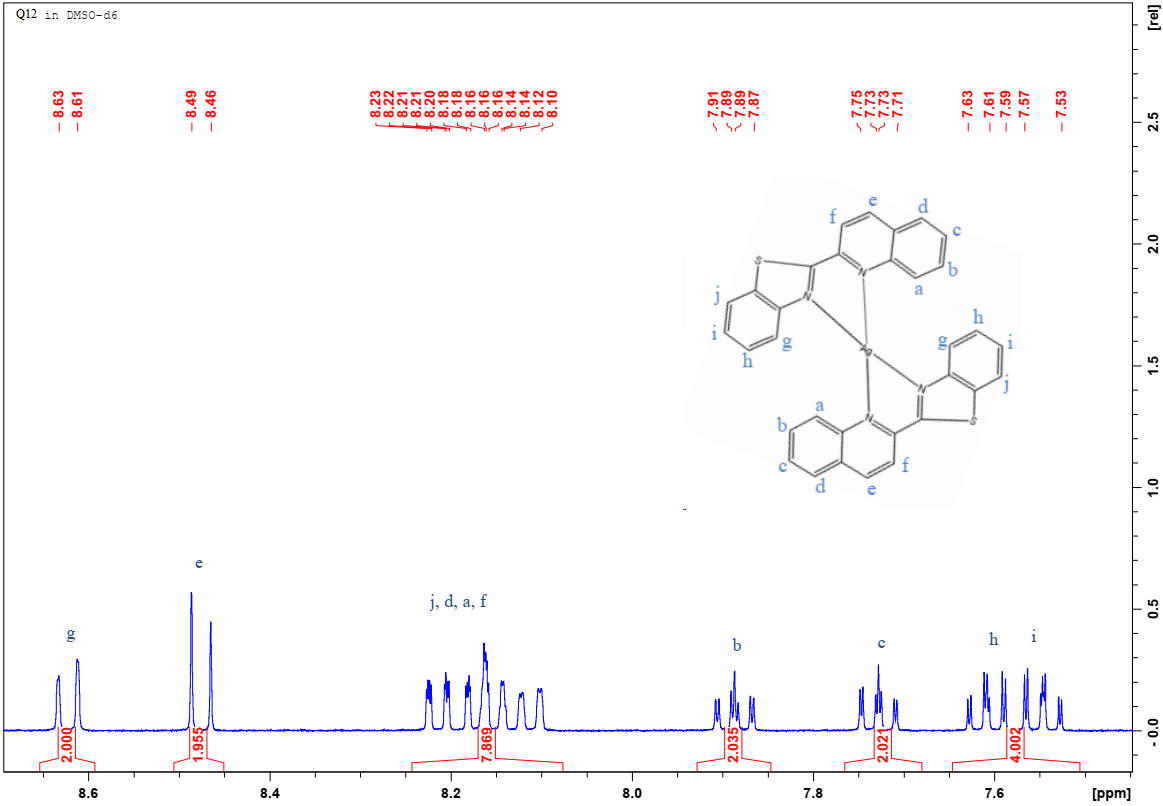


## Figure S94: ***[Ag(L2)_2_]CF_3_SO_3_ Q12***


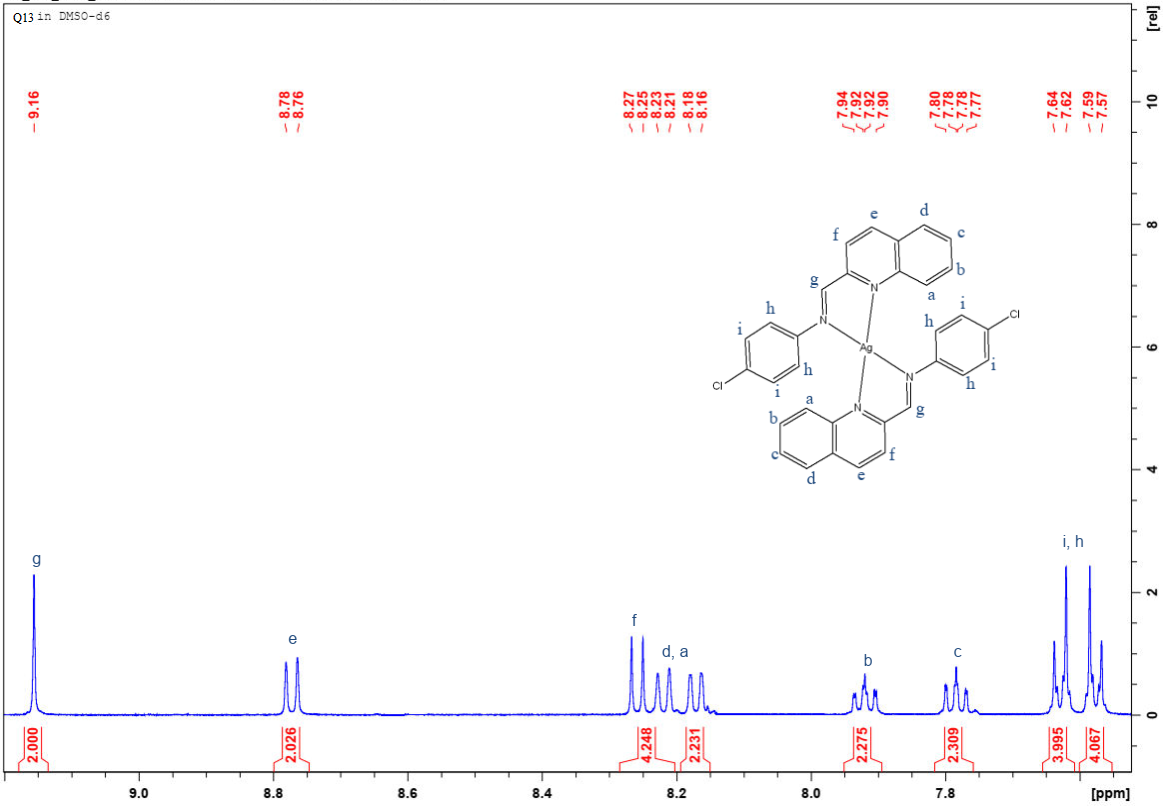


## Figure S95: ***[Ag(L3)_2_]CF_3_SO_3_ Q13***


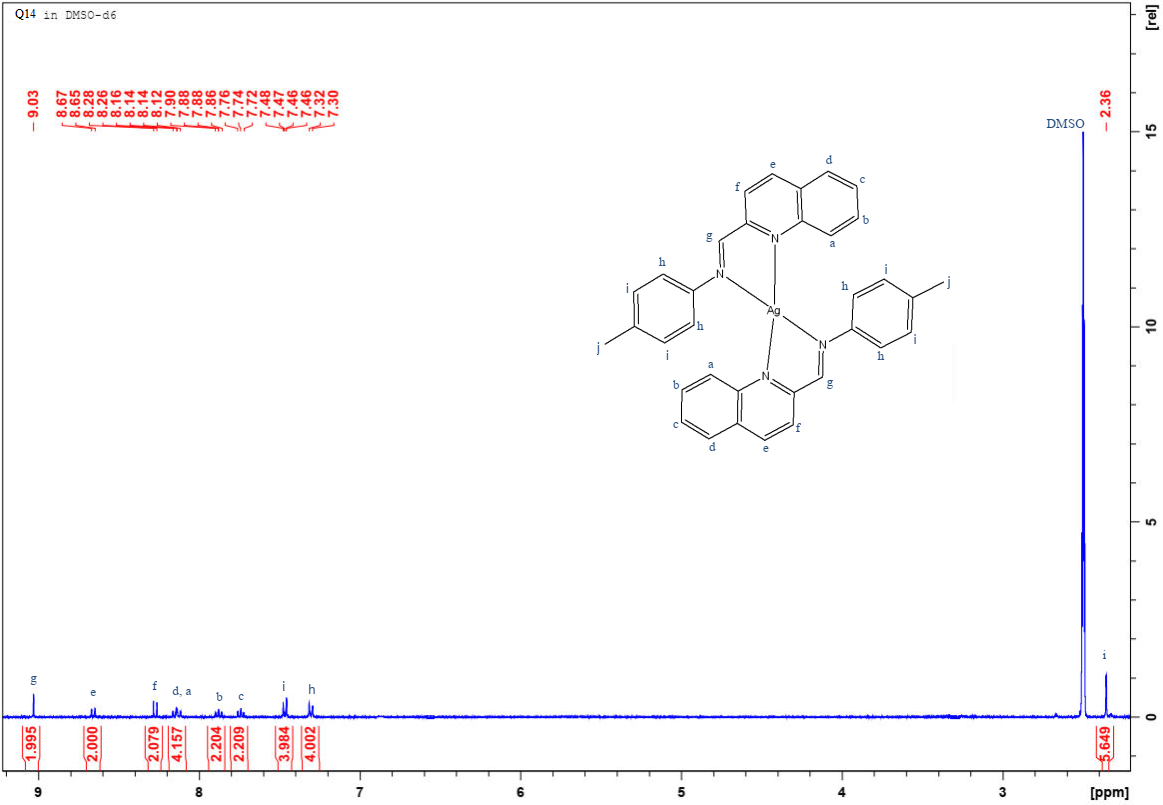


## Figure S96: ***[Ag(L4)_2_]CF_3_SO_3_ Q14***


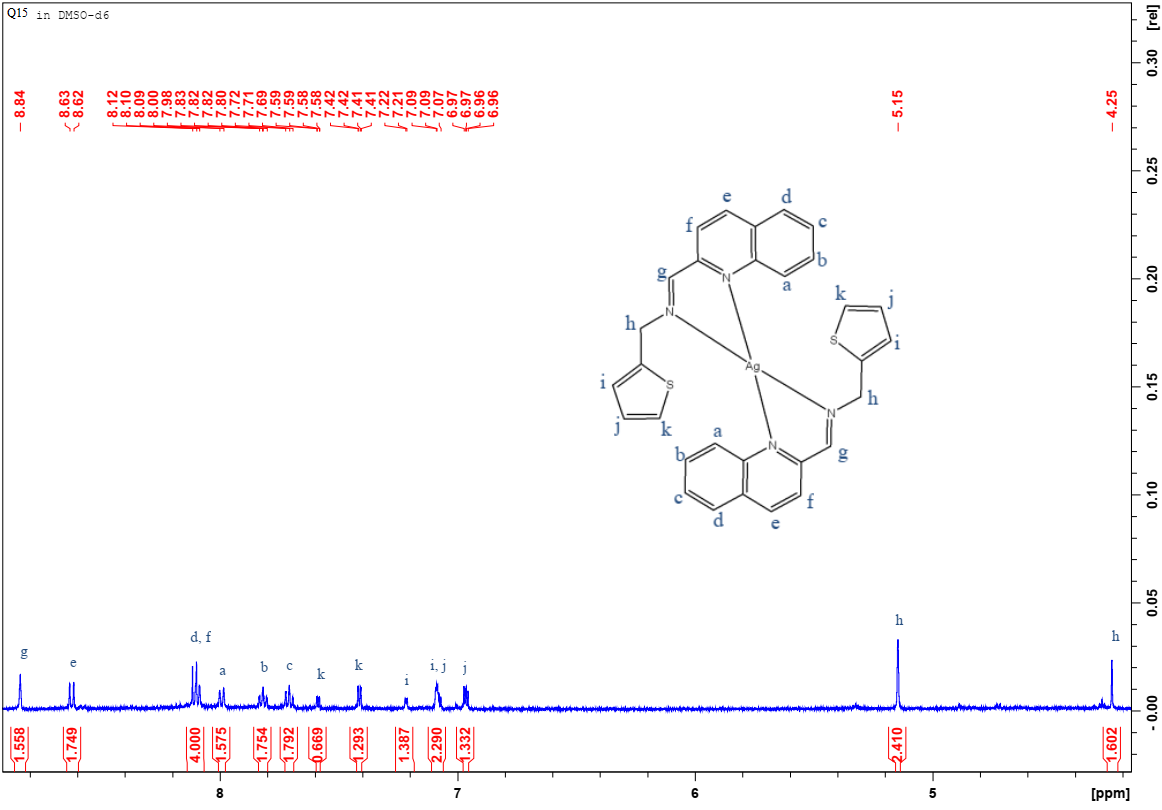


## Figure S97: ***[Ag(L5)_2_]CF_3_SO_3_ Q15***

# **^13^C-NMR SPECTRA OF L1-L5**


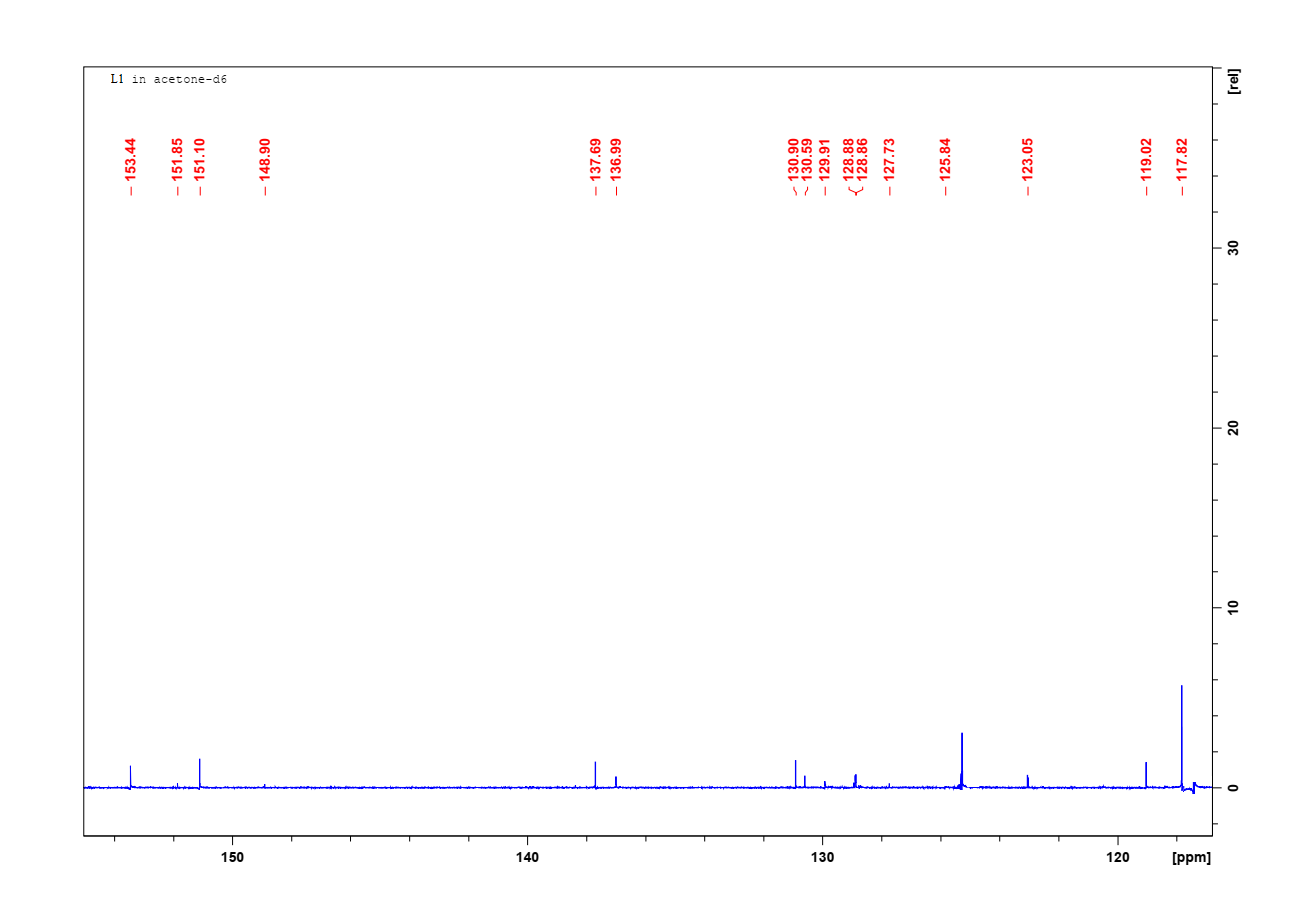


## Figure S98: (E)-N-(2-fluorophenyl)-1-(quinolin-2-yl)methanimine L1


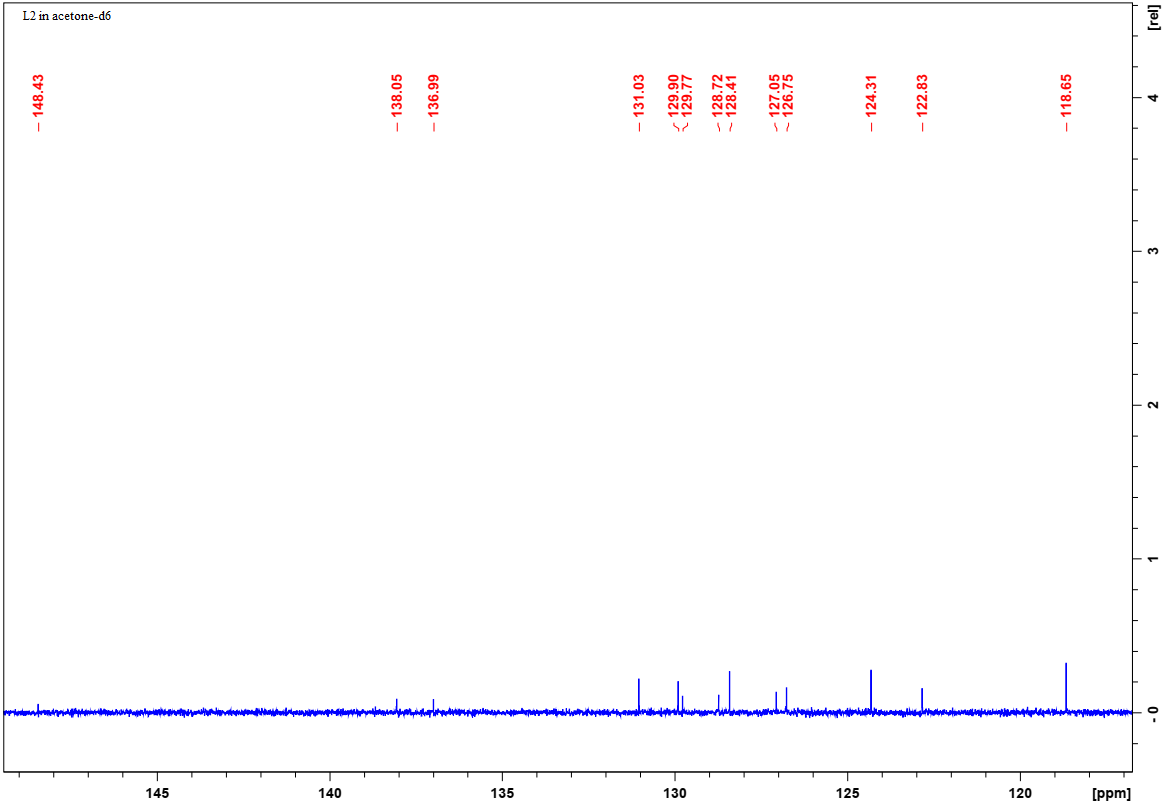


## Figure S99: 2-(quinolin-2-yl)benzo[d]thiazole L2


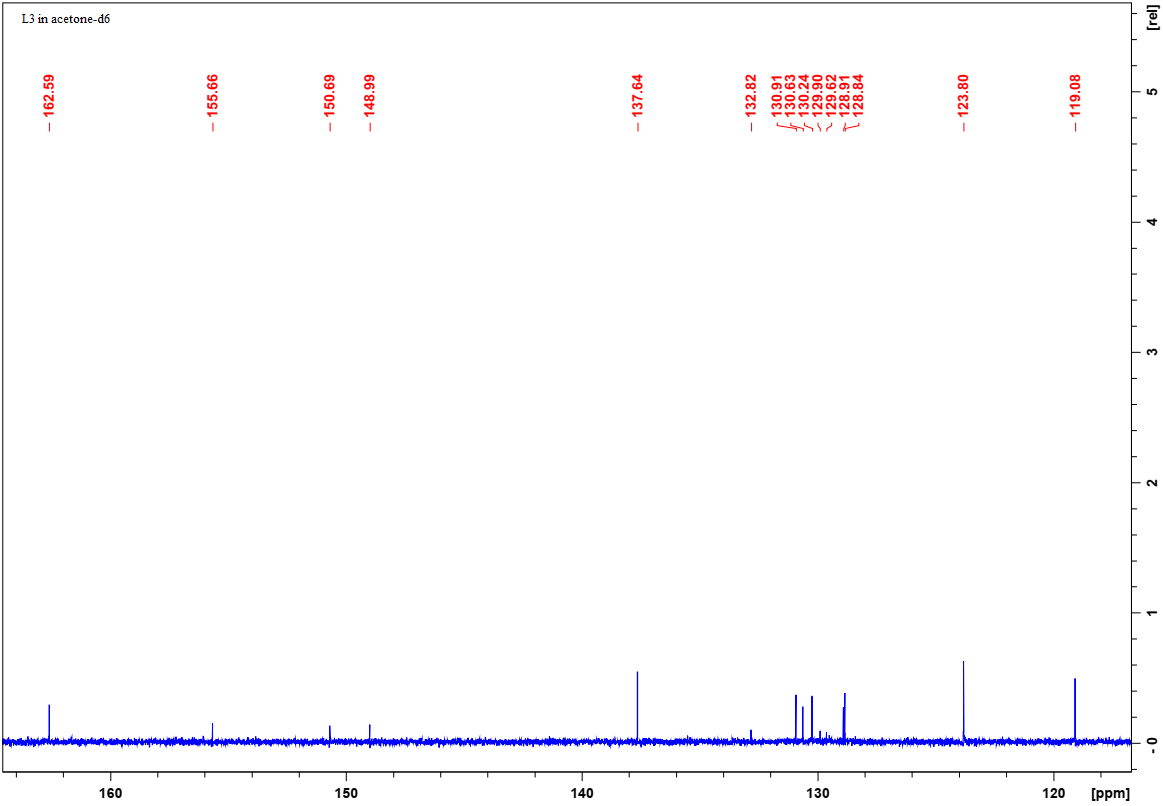


## Figure S100: (E)-N-(4-chlorophenyl)-1-(quinolin-2-yl)methanimine L3

## Figure S101: (E)-1-(quinolin-2-yl)-N-(p-tolyl)methanimine L4

## Figure S102: (E)-1-(quinolin-2-yl)-N-(thiophen-2-ylmethyl)methanimine L5

# **^13^C-NMR SPECTRA OF COMPLEXES Q1-Q15**

## Figure S103: ***[Ag(L1)_2_]NO_3_ Q1***

## Figure S104: ***[Ag(L2)_2_]NO_3_ Q2***

## Figure S105: ***[Ag(L3)_2_]NO_3_ Q3***

## Figure S106: ***[Ag(L4)_2_]NO_3_ Q4***

## Figure S107: ***[Ag(L5)_2_]NO_3_ Q5***

## Figure S108: ***[Ag(L1)_2_]ClO_4_ Q6***

## Figure S109: ***[Ag(L2)_2_]ClO_4_ Q7***

## Figure S110: ***[Ag(L3)_2_]ClO_4_ Q8***

## Figure S111: ***[Ag(L4)_2_]ClO_4_ Q9***

## Figure S112: ***[Ag(L5)_2_]ClO_4_ Q10***

## Figure S113: ***[Ag(L1)_2_]CF_3_SO_3_ Q11***

## Figure S114: ***[Ag(L2)_2_]CF_3_SO_3_ Q12***

## Figure S115: ***[Ag(L3)_2_]CF_3_SO_3_ Q13***

## Figure S116: ***[Ag(L4)_2_]CF_3_SO_3_ Q14***

## Figure S117: ***[Ag(L5)_2_]CF_3_SO_3_ Q15***

# IR SPECTRA OF LIGANDS L1-L5

## Figure S118: (E)-N-(2-fluorophenyl)-1-(quinolin-2-yl)methanimine L1

## Figure S119: 2-(quinolin-2-yl)benzo[d]thiazole L2

## Figure S120: (E)-N-(4-chlorophenyl)-1-(quinolin-2-yl)methanimine L3

## Figure S121: (E)-1-(quinolin-2-yl)-N-(p-tolyl)methanimine L4

## Figure S122: (E)-1-(quinolin-2-yl)-N-(thiophen-2-ylmethyl)methanimine ***L5***

# IR SPECTRA OF COMPLEXES Q1-Q15

## Figure S123: ***[Ag(L1)_2_]NO_3_ Q1***

## Figure S124: ***[Ag(L2)_2_]NO_3_ Q2***

## Figure S125: ***[Ag(L3)_2_]NO_3_ Q3***

## Figure S126: ***[Ag(L4)_2_]NO_3_ Q4***

## Figure S127: ***[Ag(L5)_2_]NO_3_ Q5***

## Figure S128: ***[Ag(L1)_2_]ClO_4_ Q6***

## Figure S129: ***[Ag(L2)_2_]ClO_4_ Q7***

## Figure S130: ***[Ag(L3)_2_]ClO_4_ Q8***

## Figure S131: ***[Ag(L4)_2_]ClO_4_ Q9***

## Figure S132: ***[Ag(L5)_2_]ClO_4_ Q10***

## Figure S133: ***[Ag(L1)_2_]CF_3_SO_3_ Q11***

## Figure S134: ***[Ag(L2)_2_]CF_3_SO_3_ Q12***

## Figure S135: ***[Ag(L3)_2_]CF_3_SO_3_ Q13***

## Figure S136: ***[Ag(L4)_2_]CF_3_SO_3_ Q14***

## Figure S137: ***[Ag(L5)_2_]CF_3_SO_3_ Q15***

# **Mass Spectra of Ligands L1-L5**

## Figure S138: (E)-N-(2-fluorophenyl)-1-(quinolin-2-yl)methanimine L1

## Figure S139: 2-(quinolin-2-yl)benzo[d]thiazole L2

## Figure S140: (E)-N-(4-chlorophenyl)-1-(quinolin-2-yl)methanimine L3

Figure S141: (E)-1-(quinolin-2-yl)-N-(p-tolyl)methanimine L4

## Figure S142: (E)-1-(quinolin-2-yl)-N-(thiophen-2-ylmethyl)methanimine L5

# **MASS SPECTRA OF COMPLEXES Q1-Q15**

## Figure S143: ***[Ag(L1)_2_]NO_3_ Q1***

## Figure S144: ***[Ag(L2)_2_]NO_3_ Q2***

## Figure S145: ***[Ag(L3)_2_]NO_3_ Q3***

## Figure S146: ***[Ag(L4)_2_]NO_3_ Q4***

## Figure S147: ***[Ag(L5)_2_]NO_3_ Q5***

## Figure S148: ***[Ag(L1)_2_]ClO_4_ Q6***

## Figure S149: ***[Ag(L2)_2_]ClO_4_ Q7***

## Figure S150: ***[Ag(L3)_2_]ClO_4_ Q8***

## Figure S151: ***[Ag(L4)_2_]ClO_4_ Q9***

## Figure S152: ***[Ag(L5)_2_]ClO_4_ Q10***

## Figure S153: ***[Ag(L1)_2_]CF_3_SO_3_ Q11***

## Figure S154: ***[Ag(L2)_2_]CF_3_SO_3_ Q12***

## Figure S155: ***[Ag(L3)_2_]CF_3_SO_3_ Q13***

## Figure S156: ***[Ag(L4)_2_]CF_3_SO_3_ Q14***

## Figure S157: ***[Ag(L5)_2_]CF_3_SO_3_ Q15***
